# Atezolizumab versus chemotherapy in advanced or metastatic NSCLC with high blood-based tumor mutational burden: primary analysis of BFAST cohort C randomized phase 3 trial

**DOI:** 10.1038/s41591-022-01933-w

**Published:** 2022-08-22

**Authors:** Solange Peters, Rafal Dziadziuszko, Alessandro Morabito, Enriqueta Felip, Shirish M. Gadgeel, Parneet Cheema, Manuel Cobo, Zoran Andric, Carlos H. Barrios, Masafumi Yamaguchi, Eric Dansin, Pongwut Danchaivijitr, Melissa Johnson, Silvia Novello, Michael S. Mathisen, Sarah M. Shagan, Erica Schleifman, Jin Wang, Mark Yan, Simonetta Mocci, David Voong, David A. Fabrizio, David S. Shames, Todd Riehl, David R. Gandara, Tony Mok

**Affiliations:** 1 Centre Hospitalier Universitaire Vaudois, Lausanne University, Lausanne, Switzerland; 2 Medical University of Gdańsk, Gdańsk, Poland; 3 Istituto Nazionale Tumori ‘Fondazione G Pascale’, IRCCS, Naples, Italy; 4 Vall d’Hebron University Hospital, Vall d’Hebron Institute of Oncology, Barcelona, Spain; 5 Henry Ford Cancer Institute/Henry Ford Health System, Detroit, MI USA; 6 William Osler Health System, University of Toronto, Brampton, Ontario Canada; 7 Unidad de Gestión Clínica Intercentros de Oncología Médica, Hospitales Universitarios Regional y Virgen de la Victoria, IBIMA, Málaga, Spain; 8 University Hospital Medical Center Bezanijska Kosa, Belgrade, Serbia; 9 Oncology Research Center HSL/PUCRS, Porto Alegre, Brazil; 10 National Hospital Organization Kyushu Cancer Center, Fukuoka, Japan; 11 Center Oscar Lambret, Lille, France; 12 Faculty of Medicine, Siriraj Hospital, Bangkok, Thailand; 13 Sarah Cannon Research Institute, Nashville, TN USA; 14 University of Turin, S. Luigi Gonzaga Hospital, Orbassano, Italy; 15 Genentech, Inc, South San Francisco, CA USA; 16 F. Hoffmann La-Roche, Ltd, Basel, Switzerland; 17 Foundation Medicine, Inc, Cambridge, MA USA; 18 UC Davis Comprehensive Cancer Center, Sacramento, CA USA; 19 State Key Laboratory of Translational Oncology, Department of Clinical Oncology, The Chinese University of Hong Kong, Hong Kong, China; 20 Present Address: Gilead Sciences, Inc, Foster City, CA USA

**Keywords:** Predictive markers, Non-small-cell lung cancer

## Abstract

Tumor mutational burden (TMB) is being explored as a predictive biomarker for cancer immunotherapy outcomes in non-small cell lung cancer. BFAST (NCT03178552)—an open-label, global, multicohort trial—evaluated the safety and efficacy of first-line targeted therapies or immunotherapy in patients with unresectable Stage IIIB or IV advanced or metastatic non-small cell lung cancer who were selected for biomarker status using blood-based targeted next-generation sequencing. In the Phase 3 cohort C evaluating blood-based (b)TMB as a biomarker of atezolizumab efficacy, patients with bTMB of ≥10 (*N* = 471) were randomized 1:1 to receive atezolizumab or platinum-based chemotherapy per local standard of care. Cohort C did not meet its primary endpoint of investigator-assessed progression-free survival in the population with bTMB of ≥16 (hazard ratio, 0.77; 95% confidence interval: 0.59, 1.00; *P* = 0.053). Adverse events leading to treatment withdrawal occurred in 10% of patients in the atezolizumab arm and 20% in the chemotherapy arm. Adverse events of special interest occurred in 42% of patients in the atezolizumab arm and 26% in the chemotherapy arm. A prespecified exploratory analysis compared the bTMB clinical trial assay with the FoundationOne Liquid Companion Diagnostic assay and showed high concordance between assays. Additional exploration of bTMB to identify optimal cutoffs, confounding factors, assay improvements or cooperative biomarkers is warranted.

## Abstract (web summary)

In a phase 3 trial, selection of non-small cell lung cancer patients based on high blood-based tumor mutation burden did not improve clinical response to the checkpoint blockade inhibitor atezolizumab, as compared with standard of care platinum-based chemotherapy.

## Main

TMB has emerged as a biomarker for cancer immunotherapy as demonstrated by the approval of pembrolizumab for the treatment of selective solid tumors that are tissue-based (t)TMB high^1,2^. TMB in both tissue and blood is currently being explored in non-small cell lung cancer (NSCLC)^3^. tTMB, determined by whole-exome sequencing, is associated with clinical benefit from multiple checkpoint inhibitors at various cutoffs^1,4–10^. Assessing TMB using large comprehensive gene panels to target a portion of the genome has been shown to correlate with whole-exome sequencing^11–15^. TMB is associated with predicted neoantigen load and also predicts clinical benefit of anti-programmed death-ligand 1 (PD-L1)/programmed death 1 (PD-1) treatments in NSCLC^5,16^. Evidence from mostly retrospective analyses of clinical trials in the first- (1L) or second-line treatment of NSCLC suggests that TMB predicts the efficacy of PD-L1/PD-1 inhibitor monotherapy, but not in combination with chemotherapy^3^, and that tTMB is positively correlated with bTMB^17^. In NSCLC, retrospective analyses have shown TMB is independent of PD-L1 expression and seems to be potentially predictive of progression-free survival (PFS) benefit, but not of overall survival (OS), with checkpoint inhibitor monotherapy^4,18–20^. However, prospective Phase 3 trials using TMB as a predictive biomarker are lacking.

Atezolizumab monotherapy is effective for the 1L treatment of patients with squamous or nonsquamous advanced or metastatic NSCLC without *EGFR/ALK* alterations whose tumors have high PD-L1 expression. The Phase 3 IMpower110 trial (*N* = 572) enrolled patients with NSCLC with PD-L1-positive tumors. Patients with high PD-L1 expression, defined as ≥50% of tumor cells or ≥10% of tumor-infiltrating immune cells assessed by the SP142 immunohistochemistry (IHC) assay (Ventana), showed a median OS of 20.2 months with atezolizumab monotherapy versus 13.1 months with platinum-based chemotherapy (hazard ratio (HR), 0.59; 95% confidence interval (CI): 0.40, 0.89; *P* = 0.0106)^18^. However, OS benefit was not observed in the patient subgroup consisting of high or intermediate PD-L1 expression (≥5% of tumor or tumor-infiltrating immune cells). Additionally, among patients with any tumor PD-L1 expression and bTMB of ≥16 (bTMB clinical trial assay (CTA); Foundation Medicine, Inc, Cambridge, MA), patients given atezolizumab had longer PFS than patients given chemotherapy (HR, 0.55; 95% CI, 0.33, 0.92), suggesting a cooperative predictive value between these independent biomarkers^18^. Therefore, a biomarker such as bTMB that is independent of PD-L1 and can select patients who benefit from PD-L1/PD-1 inhibitors could help broaden access to 1L chemotherapy-free treatment options. Furthermore, given that up to 30% of patients have insufficient tissue at diagnosis for comprehensive biomarker testing^21^, a blood-based assay for TMB that would obviate the need for tTMB analysis could identify patients who benefit from atezolizumab therapy^22^.

B-F1RST (*N* = 152) was the first prospective Phase 2 study evaluating bTMB as a biomarker for 1L atezolizumab monotherapy in locally advanced or metastatic NSCLC^23^. The primary efficacy objective of this single-arm open-label trial was objective response rate (ORR); the primary biomarker objective was the relationship of investigator-assessed (INV) PFS in patients with bTMB of ≥16. The trial met its primary efficacy endpoint but not the primary PFS-related biomarker objective. However, incremental increases in ORR were observed with increasing bTMB cutoffs.

**Extended Data Fig. 1:**
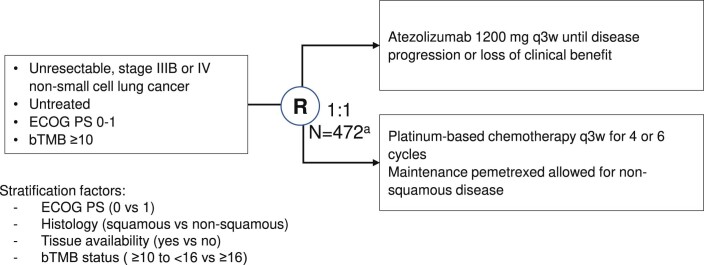
BFAST study design. bTMB, blood-based tumor mutational burden; ECOG PS, Eastern Cooperative Oncology Group performance status; eCRF, electronic case report form; q3w, every 3 weeks; R, randomized. ^a^One patient was excluded from the analysis population due to randomization by error.

The Blood First Assay Screening Trial (BFAST, NCT03178552, ClinicalTrials.gov) is a global, open-label, multicohort trial evaluating the safety and efficacy of targeted therapies or immunotherapy in patients with previously untreated unresectable Stage IIIB–IV NSCLC selected for several different biomarkers, including driver mutations and TMB, using a blood-based next-generation sequencing assay (Extended Data Fig. 1). BFAST cohort C is the first prospective randomized study to assess bTMB as a predictive biomarker for immunotherapy. Patients were selected by bTMB status, and atezolizumab monotherapy was compared with platinum-based chemotherapy. bTMB status was determined by the Foundation Medicine bTMB CTA, which was developed to evaluate TMB status in blood samples using a hybridization capture-based method targeting 1.1 Mb (394 genes) of genomic sequence. The CTA requires circulating free DNA to be present at a maximum somatic allele frequency (MSAF) of ≥1% to produce reliable results^17^. The bTMB CTA reports bTMB as the number of mutations detected in the targeted 1.1 Mb of sequence. The blood-based FoundationOne Liquid (F1L) Companion Diagnostic (CDx) next-generation sequencing assay is a United States Food and Drug Administration-approved assay that targets 0.8 Mb and reports bTMB as the number of mutations per megabase. Here, we report primary results from BFAST cohort C and the exploratory outcomes when using the F1L CDx assay results.

## Results

### Baseline characteristics

**Fig. 1:**
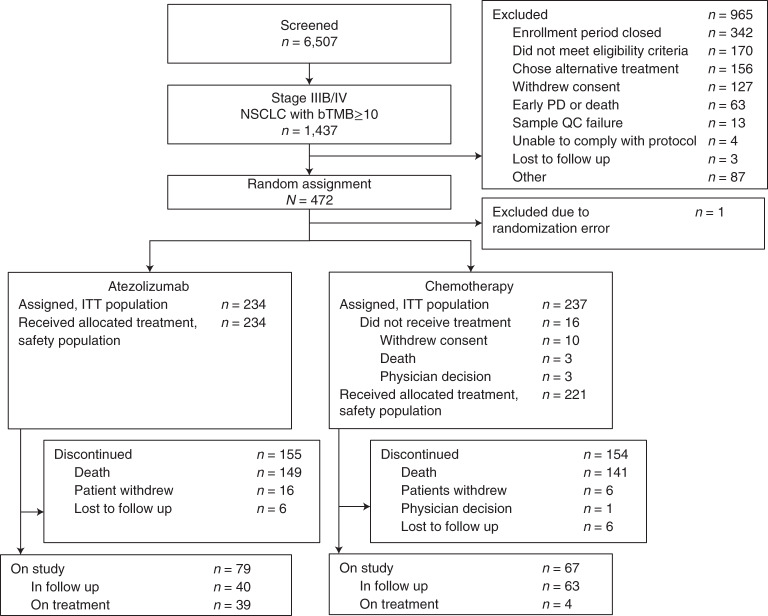
Patient flow diagram for the patient-reported outcome analyses from BFAST study cohort C. PD, progressive disease; QC, quality check.

Patients were enrolled at 120 centers across 25 countries. From 22 September 2017 to 21 November 2019, 6,507 patients were screened, 1,437 of whom (22.1%) had a score of bTMB of ≥10; 472 patients were enrolled in the intent-to-treat (ITT) population and randomized to atezolizumab (*n* = 234) or chemotherapy (*n* = 237; Fig. 1). One patient randomized in error was subsequently excluded from the analysis. The bTMB of ≥16 population included 145 patients in the atezolizumab arm and 146 in the chemotherapy arm. The baseline demographics and characteristics were generally balanced in both arms for the ITT and bTMB ≥16 populations (Table 1). Although patients whose tumors had *ALK* fusions, *EGFR* L858R mutations or *EGFR* exon 19 deletions were excluded, other known driver alterations were potentially eligible, and a small number of patients enrolled across the study (*BRAF* V600E (*n* = 2), *ROS1* (*n* = 1), *RET* (*n* = 1), *HER2* exon 20 insertion (*n* = 1) and *EGFR* exon 20 insertion (*n* = 1).

**Table 1:** Baseline demographics and characteristics

Characteristic	bTMB ≥10 (ITT)		bTMB ≥16	
	Atezolizumab *N* = 234	Chemotherapy *N* = 237	Atezolizumab *n* = 145	Chemotherapy *n* = 146
Age, median (min–max), years	66 (39–89)	66 (33–86)	65 (39–89)	66 (40–86)
Age <65 years, *n* (%)	108 (46)	102 (43)	72 (50)	65 (45)
Male, *n* (%)	170 (73)	176 (74)	106 (73)	108 (74)
Race, *n* (%)				
White	167 (71)	173 (73)	103 (71)	106 (73)
Asian	38 (16)	43 (18)	20 (14)	32 (22)
Black	4 (2)	2 (<1)	4 (3)	1 (<1)
American Indian/Alaska Native	3 (1)	5 (2)	3 (2)	2 (1)
Native Hawaiian/Pacific Islander	1 (<1)	0	1 (<1)	0
Unknown	21 (9)	14 (6)	14 (10)	5 (3)
Baseline ECOG PS per eCRF, *n* (%)				
0	66 (28)	65 (27)	40 (28)	41 (28)
1	166 (71)	171 (72)	104 (72)	105 (72)
3^a^	1 (<1)	0	0	0
Unknown	1 (<1)	1 (<1)	1 (<1)	0
Tissue available (eCRF), *n* (%)	173 (74)	186 (78)	106 (73)	115 (79)
Tobacco history, *n* (%)				
Never	4 (2)	4 (2)	4 (3)	2 (1)
Current	81 (35)	84 (35)	61 (42)	53 (36)
Previous	149 (64)	148 (62)	80 (55)	91 (62)
Unknown	0	1 (<1)		
Stage at diagnosis, *n* (%)				
I	4 (2)	5 (2)	3 (2)	2 (1)
II	7 (3)	9 (4)	4 (3)	4 (3)
III	34 (15)	41 (17)	24 (17)	23 (16)
IV	189 (81)	182 (77)	114 (79)	117 (80)
Nonsquamous histology per eCRF, *n* (%)	168 (72)	171 (72)	113 (78)	112 (77)
Metastatic sites, median (min–max)	3 (1–11)	3 (1–9)	3 (1–11)	3 (1–9)
Sum of longest diameters, median (min–max)	102 (10–253)	105 (10–246)	105 (11–253)	110 (12–246)
Liver metastases, *n* (%)	50 (21)	54 (23)	30 (21)	26 (18)
Brain metastases, *n* (%)	33 (14)	46 (19)	21 (14)	28 (19)

eCRF, electronic case report form.

^a^One patient with ECOG PS 3 was inappropriately enrolled and was considered a key protocol deviation.

### Efficacy in the bTMB ≥16 population

**Fig. 2:**
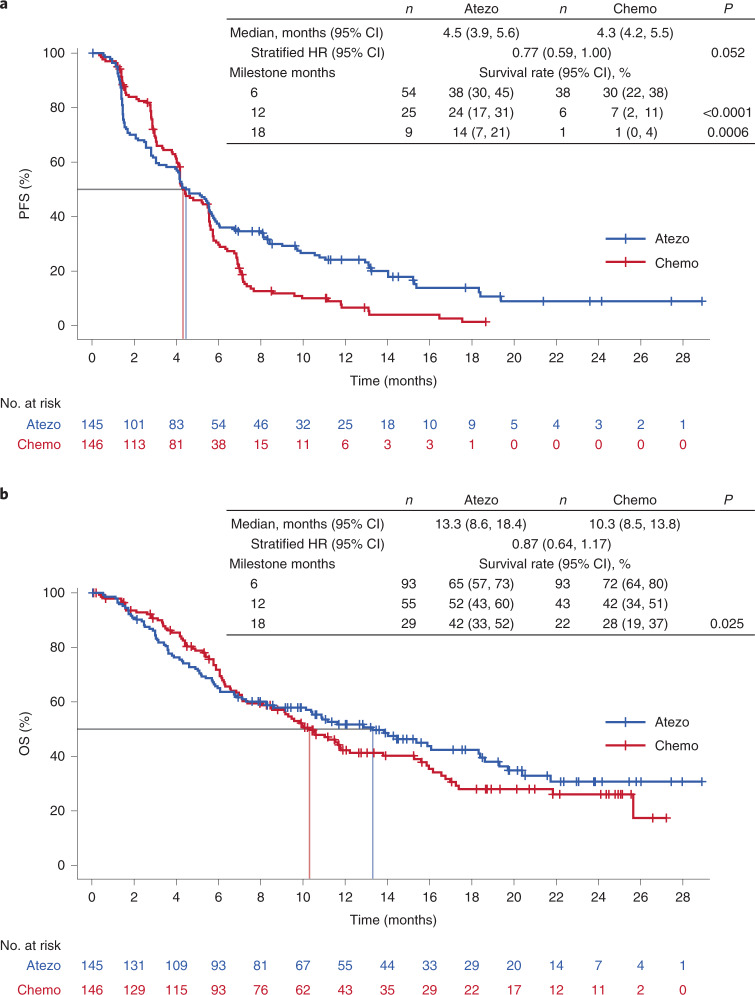
Kaplan–Meier curve for the population of patients with bTMB ≥16. **a**, Primary endpoint: investigator-assessed PFS. Statistical analysis used a stratified log-rank test at the two-sided 0.05 level and was not adjusted for multiple comparisons. **b**, Key secondary endpoint: OS. Statistical analysis used a stratified log-rank test at the two-sided 0.05 level and was not adjusted for multiple comparisons. Atezo, atezolizumab; Chemo, chemotherapy. *P* values for milestone PFS are descriptive.

**Fig. 3:**
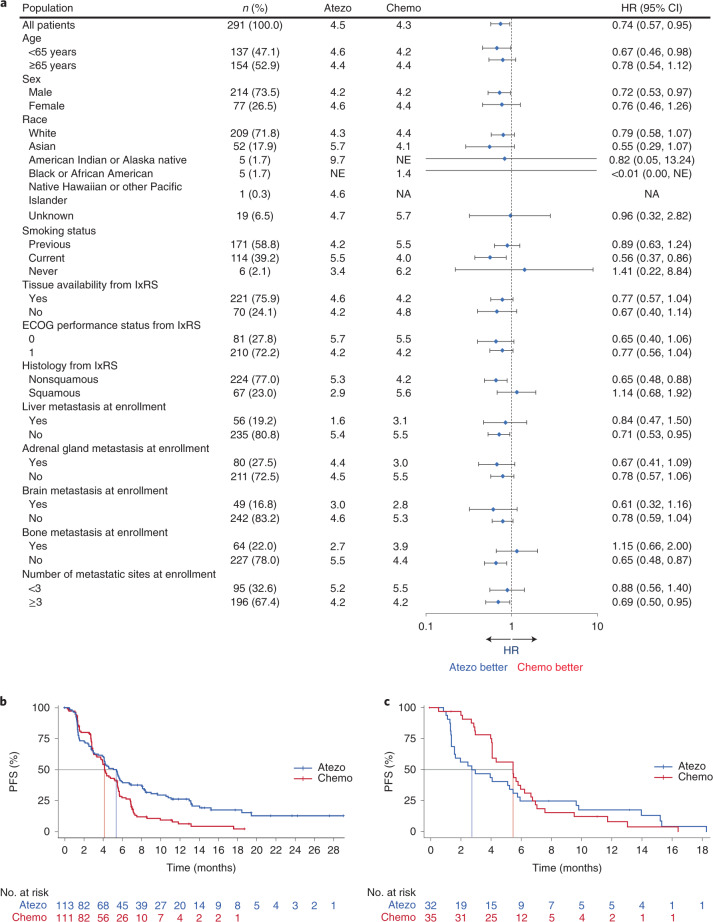
Subgroup analysis of PFS in patients with a bTMB ≥16. **a**, Forest plot of subgroups. Error bars, 95% CI. **b**,**c**, PFS Kaplan–Meier curves for the nonsquamous (**b**) and squamous (**c**) subgroups. Atezo, atezolizumab; Chemo, chemotherapy; lxRS, interactive voice/web response system; NA, not analyzed; NE, not estimable.

**Extended Data Fig. 2:**
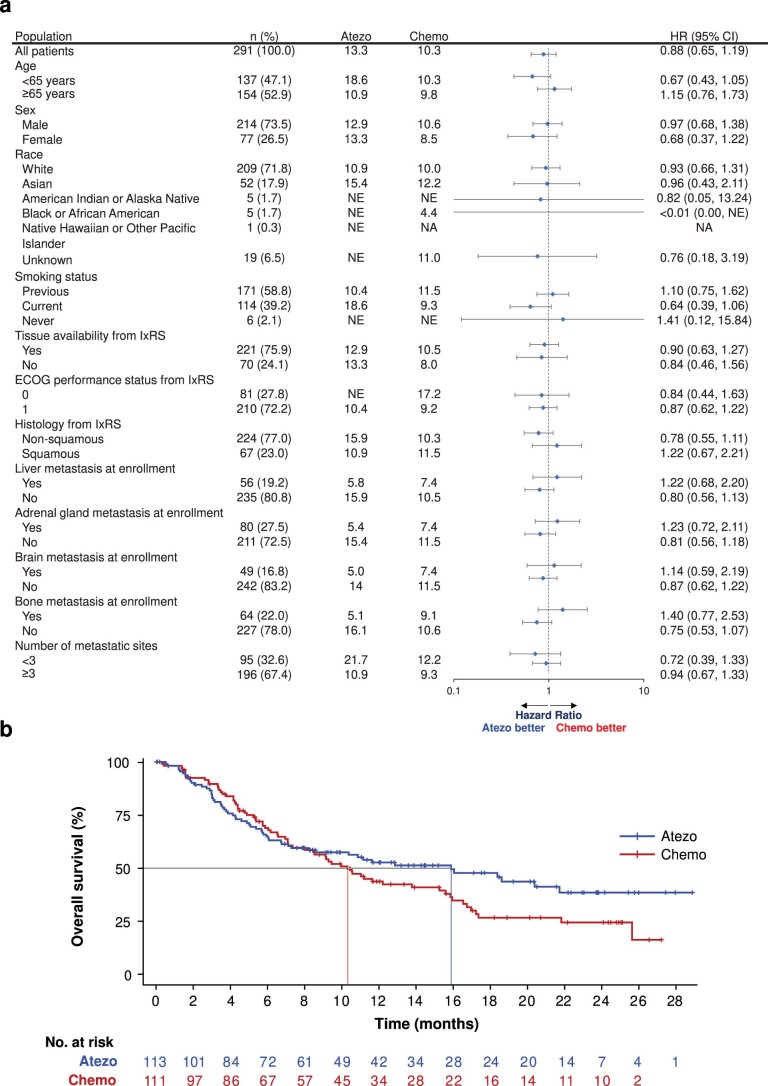
Subgroup analysis of overall survival in patients with a blood-based tumor mutational burden ≥16. (A) Forest plot of subgroups. Error bars indicate 95% confidence intervals. (B) Kaplan-Meier curves for the non-squamous subgroup. Atezo, atezolizumab; Chemo, chemotherapy; ECOG, Eastern Cooperative Oncology Group; IxRS, interactive voice/web response system; NA, not analyzed; NE, not estimable.

At clinical cutoff (21 May 2020), the minimum follow up in the bTMB ≥16 population was 6.0 months (median, 18.2 months). For the primary efficacy endpoint, INV-PFS, there was no significant difference in PFS between arms, with 243 (84%) of events having occurred (stratified HR, 0.77; 95% CI: 0.59, 1.00; *P* = 0.053; Fig. 2a and Table 2). The median PFS was 4.5 months (95% CI: 3.9, 5.6) for the atezolizumab arm and 4.3 months (95% CI: 4.2, 5.5) for the chemotherapy arm. The PFS rate was 24% (95% CI: 17, 31) in the atezolizumab arm versus 7% (95% CI: 2, 11; descriptive *P* < 0.0001) in the chemotherapy arm at 12 months and 14% (95% CI: 7, 21) versus 1% (95% CI: 0, 4; descriptive *P* = 0.0006), respectively, at 18 months (Fig. 2a). As the INV-PFS did not cross the 0.05 significance boundary, secondary endpoints were not formally tested. Subgroup analysis of PFS in the bTMB ≥16 population was generally similar across groups, except in patients with nonsquamous histology who showed a benefit with atezolizumab versus chemotherapy (unstratified HR, 0.65; 95% CI: 0.48, 0.88; Fig. 3); those with squamous histology did not (unstratified HR, 1.14; 95% CI: 0.68, 1.92). In the bTMB ≥10 population, the PFS HRs for nonsquamous (unstratified HR, 0.82; 95% CI: 0.65, 1.05) and squamous histology (unstratified HR, 1.00; 95% CI: 0.70, 1.44) had overlapping 95% CIs. OS analysis of the nonsquamous population for atezolizumab versus chemotherapy resulted in an unstratified HR of 0.78 (95% CI: 0.55, 1.11; Extended Data Fig. 2).

**Table 2:** Efficacy endpoints in the bTMB ≥16 and ITT populations

Endpoint	bTMB ≥10		bTMB ≥16	
	Atezo *n* = 234	Chemo *n* = 237	Atezo *n* = 145	Chemo *n* = 146
Median INV-PFS (95% CI), months	4.1 (2.9, 5.2)	4.4 (4.3, 5.6)	4.5 (3.9, 5.6)	4.3 (4.2, 5.5)
HR (95% CI)	0.91 (0.74, 1.11)		0.77 (0.59, 1.00)	
Median IRF-PFS (95% CI), months	2.8 (2.7, 4.2)	5.5 (4.4, 5.9)	3.6 (2.7, 5.2)	4.9 (4.2, 5.7)
HR (95% CI)	1.22 (0.99, 1.50)		1.11 (0.85, 1.45)	
Median OS (95% CI), months	10.8 (8.2, 14.0)	10.4 (9.2, 12.2)	13.3 (8.6, 18.4)	10.3 (8.5, 13.8)
HR (95% CI)	0.99 (0.79, 1.25)		0.87 (0.64, 1.17)	
Confirmed INV-ORR (95% CI), %	22 (17, 28)	23 (18, 29)	26 (19, 33)	18 (12, 25)
OR (95% CI)	0.94 (0.61, 1.45)		1.57 (0.89, 2.77)	
Confirmed IRF-ORR (95% CI), %	23 (18, 29)^a^	25 (19, 31)^a^	24 (17, 32)	20 (14, 28)
OR (95% CI)	0.91 (0.59, 1.39)		1.23 (0.70, 2.19)	
Median INV-DOR (95% CI), months	14.0 (11.0, 20.8) *n* = 52^b^	5.6 (4.9, 5.7) *n* = 55^b^	11.9 (9.5, 17.0) *n* = 37^b^	5.7 (4.4, 10.6) *n* = 26^b^
HR (95% CI)	0.28 (0.16, 0.50)		0.33 (0.16, 0.68)	
Median IRF-DOR (95% CI), months	12.7 (9.5, 17.0) *n* = 53^a^	5.7 (5.5, 7.1) *n* = 57^a^	12.7 (9.1, 14.8) *n* = 34^a^	5.6 (4.2, 11.9) *n* = 29^a^
HR (95% CI)	0.57 (0.34, 0.97)		0.73 (0.36, 1.46)	

Atezo, atezolizumab; Chemo, chemotherapy; IRF, independent review facility–assessed; OR, odds ratio.

^a^ Atezo, *n* = 232; chemo, *n* = 232.

^b^ *n* is the number of patients with complete or partial response.

### Secondary efficacy endpoints

In the bTMB ≥16 population, there was no statistically significant improvement in OS with a median of 13.3 months (95% CI: 8.6, 18.4) in the atezolizumab arm versus 10.3 months (95% CI: 8.5, 13.8) in the chemotherapy arm (stratified HR, 0.87; 95% CI: 0.64, 1.17; descriptive *P* = 0.129; Fig. 2b and Table 2). The OS probability was 52% (95% CI: 43, 60) in the atezolizumab arm versus 42% (95% CI: 34, 51) in the chemotherapy arm at 12 months and 42% (95% CI: 33, 52) versus 28% (95% CI: 19, 37; descriptive *P* = 0.025), respectively, at 18 months (Fig. 2b). Response rates and duration of response were improved numerically in the atezolizumab arm. Confirmed ORR was 26% (95% CI: 19, 33; Table 2) versus 18% (95% CI: 12, 25). Median duration of response (DOR) was 11.9 months (95% CI: 9.5, 17.0) versus 5.7 months (95% CI: 4.4, 10.6).

In the ITT population (bTMB ≥10), there was no difference between treatment arms in either PFS (stratified HR, 0.91; 95% CI: 0.74, 1.11; Table 2) or OS (stratified HR, 0.99; 95% CI: 0.79, 1.25). Median INV-PFS was 4.1 months (95% CI: 2.9, 5.2) for the atezolizumab arm versus 4.4 months (95% CI: 4.3, 5.6) in the chemotherapy arm. Median OS was 10.8 months (95% CI: 8.2, 14.0) versus 10.4 months (95% CI: 9.2, 12.2). Confirmed INV-ORR (95% CI) was 22% (95% CI: 17, 28) versus 23% (95% CI: 18, 29). The median DOR was 14.0 months (95% CI: 11.0, 20.8) versus 5.6 months (95% CI: 4.9, 5.7). In the ITT population, 3.4% of patients in the atezolizumab arm and 42.2% in the chemotherapy arm received follow up immunotherapy (Supplementary Table 1).

### Safety data

The median number of doses of atezolizumab administered was 6 (range, 1–45), with a median treatment duration of 3.5 months (Supplementary Table 2). The incidence of all-grade treatment-related adverse events was 59% with atezolizumab versus 88% with chemotherapy; the incidence of grade 3/4 events was 18% versus 46%, respectively. Adverse events leading to treatment withdrawal occurred in 10% of patients in the atezolizumab arm and 20% in the chemotherapy arm. Adverse events of special interest occurred in 42% of patients in the atezolizumab arm and 26% in the chemotherapy arm. The most common (≥5%) were rash (14% versus 10%), hypothyroidism (9% versus 2%) and pneumonitis (6% versus 1%; Table 3 and Supplementary Table 3).

**Table 3:** Summary of safety for all treated patients

Adverse events, *n* (%)	Atezo *n* = 234	Chemo *n* = 221
All-grade adverse events due to any cause	216 (92)	216 (98)
Treatment-related adverse events	138 (59)	194 (88)
Grade 3/4 adverse events	107 (46)	127 (58)
Treatment-related grade 3/4 adverse events	43 (18)	102 (46)
Serious adverse events	104 (44)	82 (37)
Treatment-related serious adverse events	27 (12)	32 (14)
Grade 5 adverse events	13 (6)	12 (5)
Treatment-related grade 5 adverse events	0	3 (1)
Adverse events leading to any treatment withdrawal	23 (10)	44 (20)
**Adverse events of special interest**		
All-grade adverse events of special interest	95 (42)	58 (26)
Grade 3/4 adverse events of special interest	29 (12)	10 (5)
All-grade adverse events of special interest requiring use of corticosteroids	41 (18)	20 (9)

Atezo, atezolizumab; Chemo, chemotherapy.

### Exploratory analysis of early disease progression

In general, this exploratory analysis suggested that early progression (≤4 months) in the bTMB ≥16 population was associated with greater disease burden at baseline, as shown by the presence of more metastatic sites or higher sum of the longest diameters (SLD) compared with late progression (Supplementary Table 4). In the atezolizumab arm, several characteristics were associated with early versus late progression (*P* < 0.1), including liver or bone metastasis, higher baseline SLD, squamous histology and presence of *KEAP1* mutations. In the chemotherapy arm, early progression was associated with liver, adrenal gland or brain metastasis, or greater number of metastatic sites overall, higher SLD or tumor mutations in *SMARCA*, *ASXL1* or *CDKN2A* (*P* < 0.05).

### Concordance of the F1L CDx assay with the bTMB CTA

**Extended Data Fig. 3:**
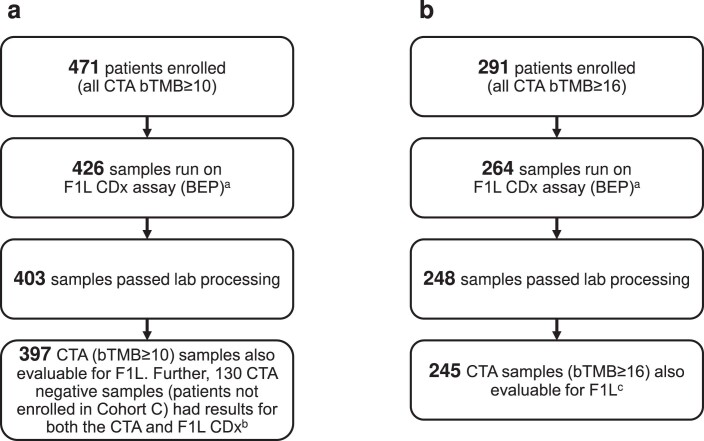
Sample flow for the concordance study comparing the bTMB CTA and F1L CDx assay for bTMB ≥10 and for bTMB ≥16. Sample flow for the concordance study comparing the bTMB CTA and F1L CDx assay for (A) bTMB ≥10 and for (B) bTMB ≥16. The bTMB ≥16 samples are a subset of the bTMB ≥10 samples. BEP, biomarker-evaluable population; bTMB, blood-based tumor mutational burden; CTA, clinical trial assay; F1L CDx, FoundationOne Liquid Companion Diagnostic. ^a^Excludes patients who withdrew consent or with unavailable samples. ^b^6 samples failed quality check. ^c^3 samples failed quality check.

**Extended Data Fig. 4:**
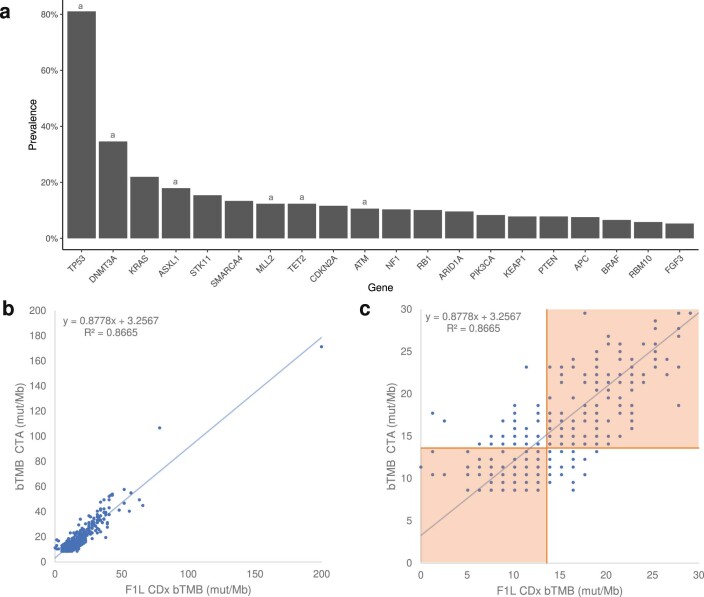
Prevalence of the 20 most commonly mutated genes in the F1L CDx-evaluable patient population and comparison of the corrected bTMB CTA and F1L CDx bTMB values. (A) Prevalence of the 20 most commonly mutated genes in the F1L CDx-evaluable patient population (n = 398). Known and likely alterations in genes with allele frequencies ≤35% are presented. (B) Comparison of the corrected bTMB CTA and F1L CDx bTMB values. (C) An expanded graph showing cases near the cutoff. Concordant cases are shown in the shaded region. ^a^ These genes are known to accumulate CHIP alterations, which is noted in the F1L CDx report, and are filtered from the bTMB calculation.

Of the 471 patients enrolled in cohort C, 426 had samples available for analysis with the F1L CDx assay (Extended Data Fig. 3). Of these, 403 samples passed laboratory processing and 6 failed quality control, leaving 397 samples for inclusion in the analysis. In addition to the 397 cohort C samples, 130 CTA-negative samples (patients screened for BFAST but not eligible for cohort C) were tested on the F1L CDx assay to evaluate assay concordance. The bTMB CTA ≥16 cutoff was determined to be equivalent to a F1L CDx value of 13.6 mutations per megabase and ten mutations was equivalent to 8.3 mutations per megabase (Methods). The 20 most commonly mutated genes tested by the F1L CDx assay are shown in Extended Data Fig. 4a. This exploratory analysis showed that the two assays were highly concordant at their respective cutoffs (Extended Data Fig. 4b,c). Among the 245 cases that were bTMB positive according to the CTA bTMB ≥16 cutoff (Supplementary Table 5), 203 were also positive according to the F1L CDx assay at the bTMB ≥13.6 mutations per megabase cutoff, resulting in a positive percentage agreement of 82.9% (203/245; 95% CI: 77.6, 87.1; Supplementary Table 6). Among the 282 cases that were bTMB CTA negative at the bTMB ≥16 cutoff, 258 were also F1L CDx negative, resulting in a negative percentage agreement of 91.5% (258/282; 95% CI: 87.7, 94.2). The positive predictive value for F1L CDx was 89.4% (95% CI: 84.8, 92.8) and the overall percentage agreement was 87.5% (95% CI: 84.4, 90.0).

**Extended Data Fig. 5:**
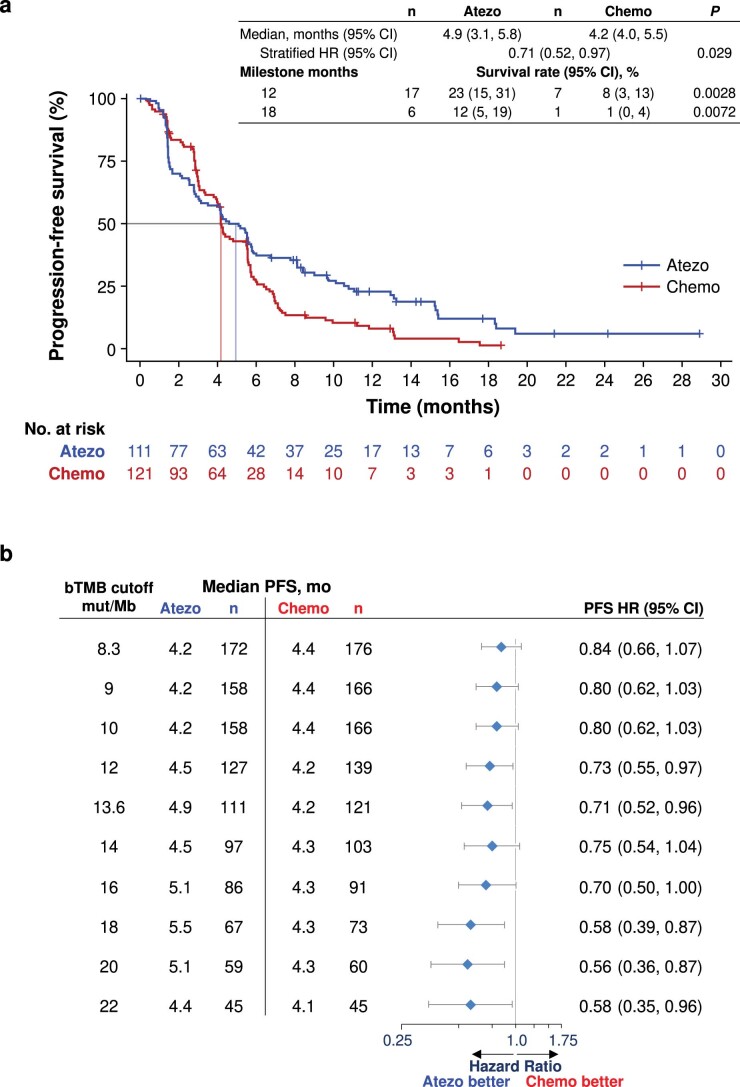
Exploratory analysis of PFS in subgroups defined by the F1L CDx assay PFS survival curves for the bTMB ≥13.6 mut/Mb population atezolizumab arm vs chemotherapy arm and PFS HRs at various cutoffs. Exploratory analysis of PFS in subgroups defined by the F1L CDx assay (A) PFS survival curves for the bTMB ≥13.6 mut/Mb population atezolizumab arm vs chemotherapy arm. Statistical analysis used a stratified log-rank test at the two-sided 0.05 level and was not adjusted for multiple comparisons. (B) Forest plot of PFS HRs at various bTMB cutoffs. Atezo, atezolizumab; bTMB, blood-based tumor mutational burden; Chemo, chemotherapy; F1L CDx, FoundationOne Liquid Companion Diagnostic assay; HR, hazard ratio; PFS, progression-free survival. *P* values are descriptive.

An exploratory analysis compared PFS and OS between subgroups above versus below the assay-determined cutoffs, as well as additional cutoff points at intervals of two mutations per megabase. bTMB determined by the F1L CDx assay showed clinical concordance for PFS, with the medians and HRs for atezolizumab benefit similar to those of the bTMB CTA ≥16 cutoff. PFS in patients above the prespecified bTMB ≥13.6 mutations per megabase cutoff according to F1L CDx was a median of 4.9 months (95% CI: 3.1, 5.8) for atezolizumab versus 4.2 months (95% CI: 4.0, 5.5) for chemotherapy (HR 0.71; 95% CI: 0.52, 0.97; descriptive *P* = 0.029; Extended Data Fig. 5a). The PFS rate was 23% (95% CI: 15, 31) in the atezolizumab arm versus 8% (95% CI: 3, 13; descriptive *P* = 0.0028) in the chemotherapy arm at 12 months, and 12% (95% CI: 5, 19) versus 1% (95% CI: 0, 4; descriptive *P* = 0.0072), respectively, at 18 months (Extended Data Fig. 5a). The PFS benefit for atezolizumab versus chemotherapy at different bTMB cutoffs as determined by F1L CDx generally increased up to the bTMB ≥18 mutations per megabase cutoff and then plateaued (Extended Data Fig. 5b). PFS HRs ranged from 0.84 (95% CI: 0.66, 1.07) to 0.56 (95% CI: 0.36, 0.87) when examined as a continuous variable across increasing bTMB cutoffs from 8.3 to 22 mutations per megabase. The optimal HR of 0.56 was achieved at bTMB ≥20 mutations per megabase.

**Extended Data Fig. 6:**
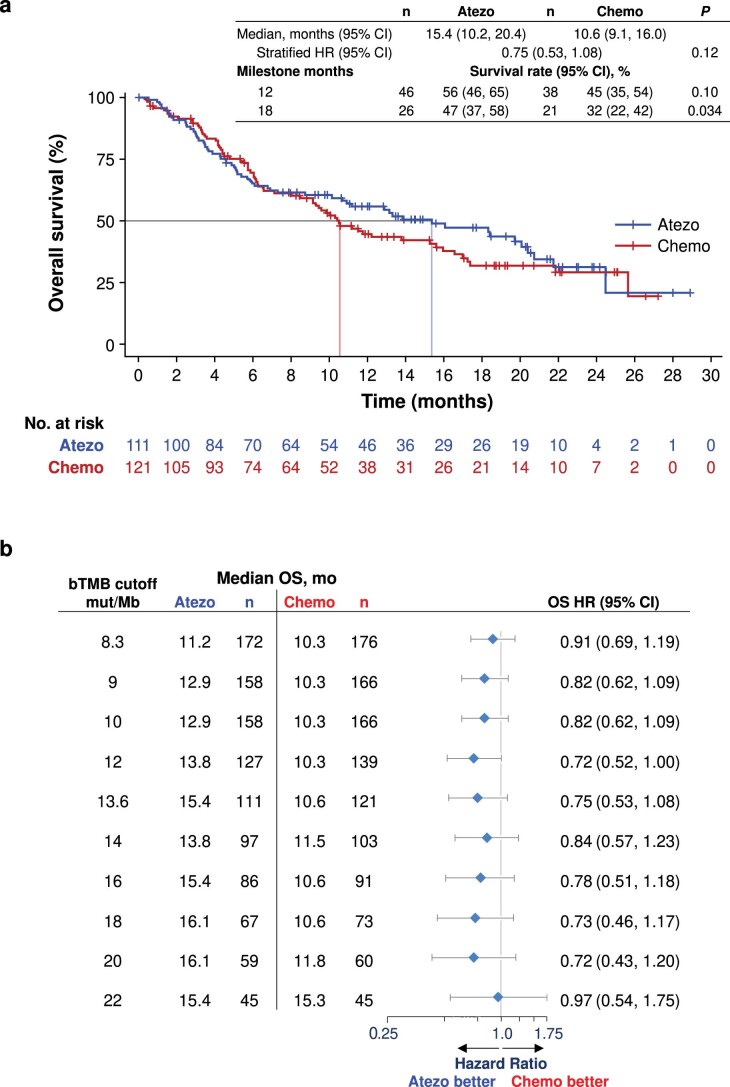
Exploratory analysis of OS in subgroups defined by the F1L CDx assay OS survival curves for the bTMB ≥13.6 mut/Mb population atezolizumab arm vs chemotherapy arm and OS HRs at various bTMB cutoffs. Exploratory analysis of OS in subgroups defined by the F1L CDx assay (A) OS survival curves for the bTMB ≥13.6 mut/Mb population atezolizumab arm vs chemotherapy arm. Statistical analysis used a stratified log-rank test at the two-sided 0.05 level and was not adjusted for multiple comparisons. (B) Forest plot of OS HRs at various bTMB cutoffs. Atezo, atezolizumab; bTMB, blood-based tumor mutational burden; Chemo, chemotherapy; F1L CDx, FoundationOne Liquid Companion Diagnostic assay; HR, hazard ratio; OS, overall survival; PFS, progression-free survival. *P* values are descriptive.

Median OS in patients with bTMB above the ≥13.6 mutations per megabase cutoff according to F1L CDx was 15.4 months (95% CI: 10.2, 20.4) with atezolizumab versus 10.6 months (95% CI: 9.1, 16.0) with chemotherapy (HR, 0.75; 95% CI: 0.53, 1.08; Extended Data Fig. 6a). The OS rate was 56% (95% CI: 46, 65) in the atezolizumab arm versus 45% (95% CI: 35, 54) in the chemotherapy arm at 12 months and 47% (95% CI: 37, 58) versus 32% (95% CI: 22, 42; *P* = 0.034), respectively, at 18 months (Extended Data Fig. 6a). The OS benefits with atezolizumab versus chemotherapy at different bTMB cutoffs seemed to numerically increase up to bTMB ≥12 mutations per megabase and then showed no discernible trend at bTMB ≥13.6 mutations per megabase and above (Extended Data Fig. 6b). OS HRs ranged from 0.97 (95% CI: 0.54, 1.75) to 0.72 (95% CI: 0.52, 1.00) when examined as a continuous variable across increasing bTMB cutoffs from 8.3 mutations per megabase to 22 mutations per megabase. The optimal HR of 0.72 was achieved at bTMB ≥12 mutations per megabase.

## Discussion

Although checkpoint inhibitor therapy has been demonstrated to improve OS versus chemotherapy in several Phase 3 trials in the first-line treatment of advanced stage NSCLC, only a minority of patients sustain long-term survival benefit, regardless of PD-L1 status. Thus, there is a continuing unmet need to define additional predictive biomarkers of the efficacy of checkpoint inhibitors. In this regard, TMB has been shown to be largely nonoverlapping and complementary to PD-L1 status in multiple retrospective analyses^4,18,24,25^. BFAST is the first prospective Phase 3 trial to investigate bTMB in this clinical setting, employing an analytically validated assay with encouraging preliminary data in the proof-of-principle B-F1RST study^23^. In B-F1RST, ORR in the ITT population was 17.1%, and there was a trend toward longer-term benefit in the bTMB ≥16 subgroup than in the bTMB <16 subgroup, with a median PFS of 5.0 versus 3.5 months (HR, 0.80; 90% CI: 0.54, 1.18; *P* = 0.35) and a median OS of 23.9 versus 13.4 months (HR, 0.66; 90% CI: 0.40, 1.10; *P* = 0.18). Although there was no significant difference in PFS observed in BFAST between atezolizumab and chemotherapy in the population with high bTMB, progression-free rates were 24% (95% CI: 17, 32) in the atezolizumab arm versus 7% (95% CI: 2, 11) in the chemotherapy arm at 12 months and 14% (95% CI: 7, 21) versus 1% (95% CI: 0.0, 4), respectively, at 18 months. Therefore, the later timepoints for PFS may better represent the outcomes of the study than the HR, as shown by the shape of the Kaplan–Meier curves, suggesting that the proportional hazards assumption may not have been met. Progression rates were initially higher in the atezolizumab arm than in the chemotherapy arm. However, the curves crossed at approximately 4 months and eventually favored atezolizumab. This Kaplan–Meier ‘crossover gap’ in PFS has been observed in multiple other Phase 3 trials of anti-PD-L1/PD-1 monotherapy versus chemotherapy and may reflect a subset of patients who respond better to cytotoxic chemotherapy and are refractory to an anti-PD-L1/PD-1 agent^4,9,18,26,27^. Similarly, although secondary endpoints were not formally tested, patients in the atezolizumab arm had a numerically longer OS than the chemotherapy arm. Furthermore, a greater percentage of patients achieved longer-term survival at 12 and 18 months, with the Kaplan–Meier curves crossing. Moreover, the overall safety profile of atezolizumab monotherapy was consistent with that seen previously across indications^18,28^.

In the PFS analysis for atezolizumab versus chemotherapy in subgroups defined by baseline disease characteristics, the nonsquamous histological group with bTMB ≥10 had an HR of 0.82 (95% CI: 0.65, 1.05), while those with bTMB ≥16 had an HR of 0.68 (95% CI: 0.48, 0.88). In the squamous group, those with bTMB ≥10 had an HR of 1.00 (95% CI: 0.70, 1.44), and those with bTMB ≥16 had an HR of 1.14 (95% CI: 0.68, 1.92). Smoking-related mutational burden, a large contributor to overall mutation burden, is reported to differ both quantitatively and qualitatively between squamous lung cancers and lung adenocarcinoma. Those with squamous histology have a higher TMB and, most importantly, a more homogeneous TMB distribution than those with nonsquamous histology^29^. Lung adenocarcinoma in particular displays a bimodal distribution consisting of high mutation (smokers) and low mutation burden (never/light-smokers and oncogene-driven cancers)^30^. Furthermore, there are also qualitative differences related to highly neoantigenic tobacco-related transversion mutations, which are more common in squamous lung cancers^29^. Among patients with nonsquamous histology in BFAST, those with bTMB ≥16 showed a greater benefit of atezolizumab versus chemotherapy. In contrast, bTMB level was not effective in predicting immunotherapy activity in those with squamous histology, characterized by a more homogeneous smoking status^29^ and a higher TMB^31^ than with nonsquamous histology. In this disease context, a distinct nonantigenic pattern of mutations, including subclonal smoking-related alterations, might explain the heterogenous pattern of clinical outcomes from atezolizumab monotherapy when patients are selected based on high bTMB.

Compared with the patient populations in other studies of 1L checkpoint inhibitor therapy, including IMpower110 (ref. ^18^), CheckMate 026 (ref. ^4^) and 227 (ref. ^25^) and MYSTIC^9^, as well as in real-world settings^32^, the population enrolled in this study, albeit bTMB-selected, seemed to have more indicators of a poor prognosis: SLD at baseline was higher and the proportion of steroid use required for adverse events was higher. However, the prevalence of ever-smokers was higher, which may have contributed to greater benefit of atezolizumab versus chemotherapy^33^. The rate of patients receiving subsequent therapy (Supplementary Table 1), which may be a measure of the disease status of the enrolled population, did not seem to be lower than has been reported for atezolizumab monotherapy in this setting^18^.

Analysis of early progression at a 4-month cutoff revealed treatment-independent differences such as higher tumor burden and more metastatic sites in early versus late progressors. Exploratory analyses showed PFS point estimates of HRs at bTMB cutoffs ﻿above 16 mutations per megabase according to the F1L CDx ranged from 0.56 to 0.70. OS showed only a weak trend with cutoffs above 13.6 mutations per megabase using F1L CDx.

A formal clinical bridging study was planned if the trial was positive to establish clinical concordance between bTMB cutoffs from the bTMB CTA and the F1L CDx assay. However, because the trial was negative, the analytical concordance and corresponding efficacy analyses were exploratory. Interestingly, PFS in cohort C using F1L CDx was longer with atezolizumab than with chemotherapy, with a PFS HR of 0.71 (95% CI: 0.52, 0.96; descriptive *P* = 0.028). bTMB determined by F1L CDx also showed improving PFS HRs at higher cutoffs, suggesting that the predictive power of bTMB may be improved by the selection of a different cutoff. This trend also suggests that bTMB is a continuous biomarker of outcomes. Selecting a single cutoff for a biomarker of clinical response, while perhaps more practical for clinicians to identify patients, has the disadvantage of making it more difficult to achieve statistical significance with a given sample size. Trial designs that dichotomize the clinical outcomes around a single cutoff can lose as much as a one-third of their statistical power^34^. In contrast, adaptive trial designs that can accommodate continuous and even nonlinear predictive effects (that is, that increase and decrease across the bTMB range) may more accurately model the characteristics of bTMB and be able to categorize patients who benefit from treatment^35^. Furthermore, differences in the computational pipeline between the bTMB CTA and F1L CDx may also play a role in the differences observed. Insertion and deletion mutations (indels) were not included in the bTMB calculation for the bTMB CTA but were included in the calculation for F1L CDx. This might have contributed to the slight differences in clinical outcomes, due potentially to the greater association of indel mutations with antitumor antigenicity compared with SNVs, for example^36^. Therefore, further study is required to validate bTMB as a standalone biomarker for the benefit of immunotherapy.

Additional factors that can impact either the bTMB score or the predictive power of bTMB include the overall fraction of tumor DNA measured in the blood, and variants derived from clonal hematopoiesis of indeterminant potential (CHIP). Patients with very low or undetectable tumor content, as measured by MSAF, have shown better responses and PFS with atezolizumab compared with patients who have high MSAF^37^. However, additional analysis demonstrated that the favorable prognostic factors associated with MSAF seem to be the drivers for the benefit, rather than MSAF itself^37^. BFAST Cohort C did not enroll low (<1%) MSAF patients.

CHIP mutations have the potential to inflate the bTMB score, although we estimate their impact on this assay to be mitigated for the following reasons. First, it has been shown that CHIP variants tend occur at low allele frequencies^38,39^, and the bTMB calculation uses an allele frequency cutoff of ≥0.5%. Second, many CHIP alterations are also cancer driver mutations that are filtered from the final bTMB score^39^. Therefore, low MSAF and CHIP mutations likely did not contribute to the negative BFAST results.

Loss of heterozygosity (LOH) at the human leukocyte antigen class I (HLA-I) locus could be a method to improve the utility of TMB. High TMB is assumed to increase production of tumor neoantigens and hence lead to increased specific T cell response^40^. Cancer immune surveillance can be abrogated not only by exhaustion of the T cell response, but also by impaired neoantigen presentation because of somatic HLA-I LOH. TMB and HLA-I LOH are independent predictors of OS^41^. However, refinements of TMB that can account for HLA-I LOH are under investigation^42^.

Other factors have also been shown to induce antitumor activity via type 1 interferon and T cell recruitment, which have not been evaluated in BFAST. For example, analyses have suggested that cytosolic DNA fragments derived from defective DNA damage response and repair mechanisms influence the response to immune checkpoint inhibitors by triggering the stimulator of interferon genes (STING) signaling pathway^43,44^. Notably, *STK11* loss might lead to immune evasion through methylation-induced suppression of STING.

Although the current results based on the bTMB CTA did not support bTMB ≥16 as a standalone predictive biomarker in 1L immunotherapy of NSCLC, the utility of bTMB as a predictive biomarker may be improved when combined with other relevant biomarkers, such as genes for immune infiltration (*CXCL9*, *CD8A*, *CD274* and *CXCL13*)^10^. A recent analysis suggests that the prediction of clinical benefit using the bTMB assay may be further optimized by incorporating it into multiparameter models^45^.

A main limitation of this study is that PD-L1 status was not known because tissue collection was not mandated and PD-L1 testing information was not collected from local sites. Additionally, during the enrollment period, pembrolizumab monotherapy was the preferred option for the 1L treatment of patients with a PD-L1-staining tumor proportion score ≥50% and was available in many countries in combination with pemetrexed and cisplatin or carboplatin, regardless of PD-L1 expression. Therefore, enrollment in the trial was potentially biased considering that eligible patients had the choice between these two pembrolizumab-based regimens or participating in BFAST, in which the control arm was chemotherapy only. This bias potentially contributed to approximately 20% of patients eligible by bTMB level either choosing an alternative treatment or withdrawing consent before randomization—a substantial proportion that could have affected the external validity of the primary results. Furthermore, approximately 7% of patients in the chemotherapy arm did not receive the planned treatment. Although we and others have shown that TMB is independent of PD-L1 IHC in predicting the benefit of single-agent anti-PD-L1 therapy, we have also shown that patients who are positive for both PD-L1 expression and TMB derive the most benefit^17,18^. However, this raises another limitation, namely that this incremental information provided by bTMB adds limited benefit in the current treatment landscape where most of the patients are already receiving anti-PD-L1 therapy. Although we cannot be certain there was a bias against enrolling patients with high PD-L1 expression, we believe that this likely contributed to shorter OS in the bTMB ≥16 population relative to the same population of B-F1RST (median 13.3 versus 23.9 months)^23^ and, consequently, to the lack of predictive value for bTMB in this study.

In summary, although using the bTMB CTA at a cutoff of ≥16 was not a predictive biomarker for atezolizumab outcome as tested in this study, the 18-month PFS and OS both numerically favored atezolizumab in the bTMB ≥16 group. By using the F1L CDx assay at an equivalent cutoff of bTMB ≥13.6 mutations per megabase, atezolizumab showed improved PFS versus chemotherapy. Additional exploration of bTMB to identify and validate optimal cutoffs or employ new adaptive trial designs to accommodate the continuous and complex nature of bTMB are warranted, as are studies to account for confounding factors such as HLA-I LOH. bTMB may have utility in combination with other relevant biomarkers (for example, PD-L1 status) to identify subgroups that may be responsive to 1L treatment with checkpoint inhibitors in NSCLC^17,18^. Further studies are needed to explore such associations.

## Methods

### Study design and patients

BFAST is an ongoing global, open-label, multicohort trial. Cohort C used a randomized, Phase 3 trial design. The study protocol is available as a Supplementary file. Eligible patients were aged ≥18 years, had previously untreated histologically or cytologically confirmed unresectable Stage IIIB or IV NSCLC according to the American Joint Committee on Cancer Staging version 7, Eastern Cooperative Oncology Group (ECOG) Performance Status (PS) of 0 or 1, measurable disease per Response Evaluation Criteria in Solid Tumors (RECIST) v.1.1, bTMB ≥10 mutations (8.3 mut Mb^–1^) as detected via the bTMB CTA and a treatment-free interval of ≥6 months if they had received previous neoadjuvant or adjuvant treatment. Key exclusion criteria included untreated brain metastasis, history of malignancy other than NSCLC in the past 5 years and presence of an oncogenic *EGFR* mutation or *ALK* fusion. As the goal of this study was to evaluate bTMB as a predictive biomarker to identify treatment for most of the patients in the 1L setting, including those who are not able to submit adequate tissue for molecular profiling, tissue was not required for enrollment. However, patients were stratified by investigator-reported tissue availability. Due to the umbrella design of the trial, in which numerous therapies were tested in biomarker-selected populations, results of local biomarker tests were not collected because of the number of tests available. Patients were also stratified by bTMB cutoff (bTMB ≥16 (13.6 mut Mb^–1^) versus between bTMB ≥10 and bTMB <16), ECOG PS (1 versus 0) and histology (nonsquamous versus squamous). Cancer driver genes were defined based on their status in the Catalog of Somatic Mutations in Cancer^46^ or evidence from literature supporting their pathogenicity status. This literature evidence must demonstrate that the mutations in the tumor suppressor gene results in loss of function. The cancer driver genes are maintained in a database at Foundation Medicine Inc. (FMI) that is updated over time as new clinical evidence emerges. Pathogenic mutations in CHIP-related genes were filtered from the bTMB calculation. Single nucleotide substitutions detected at an allele frequency of ≥0.5% were filtered for germline and driver mutations and were included in the bTMB score^17^. Samples with low tumor content within a circulating free DNA sample as measured by MSAF < 1%, were below the limit required to make reproducible bTMB determinations. bTMB was shown to be a continuous variable, and a cutoff of bTMB ≥16 was selected for further investigation based on a retrospective analysis of PFS in patients with NSCLC treated with second-line or later atezolizumab monotherapy^17,47^. Validation of the assay using Phase 3 data in the second-line or later NSCLC treatment setting^48^ showed PFS benefit at bTMB ≥16 (HR, 0.65; 95% CI: 0.47, 0.92), and the lowest cutoff with PFS benefit was bTMB ≥10 (HR, 0.73; 95% CI: 0.56, 0.95)^17^. Based on these results, BFAST cohort C defined the bTMB CTA enrollment criterion as bTMB ≥10 mutations, and the primary endpoint was evaluated at the bTMB ≥16 cutoff. Enrolling at the bTMB ≥10 cutoff while stratifying at the bTMB ≥16 cutoff enabled clinical evaluation of multiple cutoffs within cohort C.

The study was conducted in accordance with the guidelines for Good Clinical Practice and the Declaration of Helsinki, and all patients provided written informed consent. The study protocol was approved by institutional review boards of participating institutions, including the Ontario Cancer Research Ethics Board (OCREB) (Princess Margaret Cancer Center, William Osler Health System Brampton Civic Hospital, and Sunnybrook Health Sciences Center) and the University of Saskatchewan Biomedical Research Ethics Board (Saskatoon Cancer Centre).

### Study treatment

Patients were randomized 1:1 by a stratified permuted-block randomization procedure via interactive voice or Web-based response system to receive atezolizumab 1,200 mg intravenously (IV) every 3 weeks until disease progression or loss of clinical benefit, or platinum-based chemotherapy every 3 weeks for four or six cycles per local standard of care. Patients with nonsquamous disease received pemetrexed 500 mg m^–2^ in combination with cisplatin (75 mg m^–2^) or carboplatin (area under the concentration curve 5 or 6) IV with optional maintenance pemetrexed allowed. Patients with squamous disease received gemcitabine 1,250 mg m^–2^ plus cisplatin 75 mg m^–2^ or gemcitabine 1,000 mg m^–2^ plus carboplatin area under the concentration curve 5 IV for four or six cycles per local standard of care.

### Assessments

The primary endpoint evaluated in the bTMB ≥16 population, as determined by the bTMB CTA, was INV-PFS, defined as time from randomization to disease progression according to RECIST 1.1 or death. Secondary endpoints included OS, defined as time from randomization to death from any cause, in the bTMB ≥16 population, INV-PFS and OS in the bTMB ≥10 (ITT) population, PFS by independent review, ORR and DOR by INV and independent review. Safety and tolerability were assessed by incidence, type and severity of adverse events according to the National Cancer Institute Common Terminology Criteria for Adverse events v.4.0. A post hoc exploratory analysis compared the bTMB CTA used to select patients in BFAST with the F1L CDx assay, and clinical outcomes were analyzed in patients who were bTMB positive (13.6 mut Mb^–1^) by F1L CDx.

### Statistical analysis

Approximately 440 patients were planned to be enrolled in the bTMB ≥10 population, including 280 in the bTMB ≥16 population based on a one-sided significance level of 0.025 each for the comparison in the bTMB ≥10 and bTMB ≥16 populations and 95% power to detect an HR of 0.6 in the bTMB ≥16 population and 0.65 in the bTMB ≥10 population. The primary PFS endpoint in the bTMB ≥16 population was statistically tested at the *α* = 0.05 two-sided significance level using a stratified Cox regression model to estimate effect size and a log-rank test to calculate *P* values. Stratification factors for the bTMB ≥16 population were ECOG PS (0 versus 1), histology (nonsquamous versus squamous), and tissue availability (yes versus no). In the bTMB ≥10 population, the stratification factors were ECOG PS (0 versus 1), histology (nonsquamous versus squamous), bTMB cutoff (bTMB ≥10 to <16 versus bTMB ≥16), and tissue availability (yes versus no). If significant, the secondary endpoints were hierarchically tested at the two-sided *α* = 0.05 significance level (for each endpoint) in the order of OS in the bTMB ≥16 population, PFS in the bTMB ≥10 population then OS in the bTMB ≥10 population. Clinical analyses were performed using SAS (v.9.4). Biomarker exploratory analyses were performed using R (v.3.6.1).

### Exploratory analyses

#### Early disease progression

To explore factors associated with early disease progression, patients whose disease had progressed within 4 months were analyzed for baseline characteristics and genomic alterations. The analysis included the bTMB ≥16 population and was performed in each treatment arm, and in both arms combined, with a significance cutoff of *P* < 0.1. Fisher’s exact test was used to assess the association between early disease progression and binary clinical variables. For continuous clinical variables, a *t*-test was used.

#### Concordance between the bTMB CTA and F1L CDx assays

The F1L CDx assay was intended to be the assay for CDx registration but was not available at the time of study start. Per the prespecified statistical analysis plan, a clinical bridging study would have been executed to bridge the efficacy of the study from the bTMB CTA to the CDx. However, due to the negative outcome of the primary endpoint, only an analytical concordance was performed with the bTMB CTA, and efficacy analyses were exploratory.

The analysis set for the concordance study comprised available samples from all enrolled patients, excluding those who withdrew consent, for a F1L CDx biomarker-evaluable population of 426 (Extended Data Fig. 3). CTA-negative (bTMB <10) samples from patients screened for BFAST who were ineligible for enrollment in cohort C were also tested with the F1L CDx assay. The bTMB CTA–determined bTMB scores were converted to mutations per megabase (the metric used in the F1L CDx assay) by factoring the size of the T7 bait set (FMI) targets (~1.14 Mb) and then adjusting for ambiguity related to rounding using a −0.5 correction factor. Cutoffs for the prespecified analysis at bTMB ≥10 and bTMB ≥16 were calculated according to the following equations:

$$(16\;{\mathrm{mut}}-0.5)/1.14\;{\mathrm{Mb}} = 13.60\;{\mathrm{mut}}\; {\mathrm{Mb}}^{-1}$$

$$(10\;{\mathrm{mut}}-0.5)/1.14\;{\mathrm{Mb}}= 8.33\;{\mathrm{mut}}\; {\mathrm{Mb}}^{-1}$$

The overall, positive and negative percentage agreements were calculated, as well as the naive estimates of positive and negative predictive values^49^, using prevalence counts reported in Supplementary Table 5; 95% CIs were calculated using the Wilson method. PFS and OS were compared between atezolizumab and chemotherapy at the prespecified cutoffs of bTMB ≥13.6 mutations per megabase and bTMB ≥8.3 mutations per megabase and then by various additional cutoffs.

### Assessments

Tumor assessments per RECIST v.1.1 were conducted using computed tomography scans with contrast or magnetic resonance imaging at baseline, every 6 weeks (±1 week) for the first 48 weeks following treatment initiation and then every 9 weeks thereafter, regardless of dose delays, until disease progression per RECIST v.1.1 or loss of clinical benefit for patients receiving atezolizumab past disease progression. Whole blood for laboratory assessments was collected at screening, Day 1 (±3 days) of each treatment cycle and discontinuation. The incidence and severity of adverse events were assessed using the National Cancer Institute Common Terminology Criteria for Adverse Events v.4.0 at each patient contact.

### Reporting summary

Further information on research design is available in the Nature Research Reporting Summary linked to this article.

## Online content

Any methods, additional references, Nature Research reporting summaries, source data, extended data, supplementary information, acknowledgements, peer review information; details of author contributions and competing interests; and statements of data and code availability are available at 10.1038/s41591-022-01933-w.

## Supplementary information

Supplementary Information Supplementary Tables 1–7.

Reporting Summary

Supplementary Data BFAST study protocol v.6.

## Data Availability

As this study is ongoing, access to patient-level data from this trial will not be available until at least 18 months after the last patient visit and a clinical study report has been completed. After that time, requests for data will be assessed by an independent review panel, which decides whether or not the data will be provided. Once approved, the data are available for up to 24 months. At the time of writing this request platform is Vivli: https://vivli.org/ourmember/roche/. For up-to-date details on Roche’s Global Policy on the Sharing of Clinical Information and how to request access to related clinical study documents, see: https://go.roche.com/data_sharing. Anonymized records for individual patients across more than one data source external to Roche cannot be linked due to a potential increase in risk of patient reidentification. The FMI database of known driver mutations can be requested for access by contacting the FMI study review committee at src@foundationmedicine.com, where proposals are reviewed monthly and subject to data sharing agreements imposed by FMI until further notice. Figures with associated raw data include main text Figs. 1–3, main Tables 1–3, Extended Data Fig. 2–6 and Supplementary Tables 1–6.
