# Supplementary material for: Atezolizumab versus chemotherapy in advanced or metastatic NSCLC with high blood-based tumor mutational burden: primary analysis of BFAST cohort C randomized phase 3 trial
Source: Nat Med. 2022 Aug 22;28(9):1831–9. doi: 10.1038/s41591-022-01933-w (PMC9499854; doi:10.1038/s41591-022-01933-w)
Supplement: Supplementary file 1 — Supplementary Tables 1–7. [file 41591_2022_1933_MOESM1_ESM.pdf]

nature portfolio

https://doi.org/10.1038/s41591-022-01933-w

Supplementary information

Atezolizumab versus chemotherapy in advanced or metastatic NSCLC with high blood-based tumor mutational burden: primary analysis of BFAST cohort C randomized phase 3 trial

In the format provided by the authors and unedited

Atezolizumab vs chemotherapy in high blood tumor mutational burden advanced or metastatic NSCLC: Primary analysis of BFAST Cohort C randomized Phase 3 trial

Supplementary tables

Supplementary Table 1. Follow-up cancer therapies

	bTMB ≥ 10		bTMB ≥16	
**Patients receiving follow up therapy, n (%)**	**Atezo (n=234)**	**Chemo (n=237)**	**Atezo (n=145)**	**Chemo (n=146)**
**≥1 therapy**	94 (40.2)	124 (52.3)	51 (35.2)	73 (50.0)
**Chemotherapy**	93 (39.7)	58 (24.5)	50 (34.5)	37 (25.3)
**Immunotherapy**	8 (3.4)	100 (42.2)	5 (3.4)	54 (37.0)
**Targeted Therapy**	13 (5.6)	10 (4.2)	8 (5.5)	6 (4.1)

Atezo, atezolizumab; bTMB, blood-based tumor mutational burden; Chemo, chemotherapy.

Supplementary Table 2. Treatment exposure

	Atezo (n=234)	Chemo (n=221)			
		Pem	Gem	Carbo	Cis
Median treatment duration, months	3.5	3.5	2.6	2.1	2.1
Number of doses, median (range)	6 (1–45)	6 (1–31)	7 (1–12)	4 (1–6)	4 (1–7)

Atezo, atezolizumab; Carbo, carboplatin; Chemo, chemotherapy; Cis, cisplatin; Gem, gemcitabine; Pem, pemetrexed.

Supplementary Table 3. Adverse events of special interest

Medical concept	Atezo (n=234)		Chemo (n=221)	
	All grade	Grade 3/4	All grade	Grade 3/4
Rash	33 (14)	1 (<1)	21 (10)	4 (2)
Hypothyroidism	20 (9)	1 (<1)	4 (2)	0
Pneumonitis	14 (6)	8 (3)	3 (1)	1 (<1)
Hepatitis diagnosis	8 (3)	5 (2)	1 (<1)	0
Hyperthyroidism	5 (2)	0	3 (1)	0
Infusion-related reactions	5 (2)	1 (<1)	1 (<1)	0
Colitis	2 (<1)	0	1 (<1)	0
Diabetes mellitus	2 (<1)	0	2 (<1)	1 (<1)
Adrenal insufficiency	2 (<1)	1 (<1)	0	0
Severe cutaneous adverse reaction	1 (<1)	0	0	0
Myocarditis	1 (<1)	1 (<1)	0	0
Vasculitis	1 (<1)	0	1 (<1)	0
Meningoencephalitis	3 (1)	2 (<1)	1 (<1)	0
Pancreatitis	2 (<1)	1 (<1)	1 (<1)	0
Guillain-Barré syndrome	1 (<1)	0	0	0
Hypophysitis	0	0	1 (<1)	0
Myositis	1 (<1)	1<1)	0	0

Atezo, atezolizumab; Chemo, chemotherapy.

Supplementary Table 4. Clinical and biomarker variables associated with progression within 4 months

Clinical variable	Atezo bTMB ≥16 (n=145)				Chemo bTMB ≥16 (n=146)			
	Early (≤4 mo) (n=61), n (%)	Late (>4 mo) (n=84), n (%)	Odds ratio (95% CI)	*P*	Early (≤4 mo) (n=52), n (%)	Late (>4 mo) (n=94), n (%)	Odds ratio (95% CI)	*P*
Liver metastasis	19 (31)	11 (13)	0.34 (0.13, 0.83)	0.012[b]	15 (29)	11 (12)	0.33 (0.12, 0.85)	0.013[b]
Bone metastasis	20 (33)	14 (17)	0.41 (0.17, 0.96)	0.029[b]	13 (25)	17 (18)	0.66 (0.27, 1.7)	0.393
Squamous histology	18 (30)	14 (17)	0.48 (0.2, 1.1)	0.072[a]	6 (12)	28 (30)	3.2 (1.2, 10)	0.014[b]
SLD ≥86 mm	46 (75)	51 (61)	0.51 (0.23, 1.1)	0.075[a]	41 (79)	59 (63)	0.45 (0.19, 1)	0.062[a]
Adrenal gland metastasis	16 (26)	20 (24)	0.88 (0.39, 2.0)	0.846	25 (48)	19 (20)	0.28 (0.12, 0.61)	0.001[c]
Brain metastasis	11 (18)	10 (12)	0.62 (0.22, 1.7)	0.344	17 (33)	11 (12)	0.28 (0.1, 0.7)	0.004[b]
≥3 Metastatic sites	47 (77)	53 (63)	0.51 (0.22, 1.1)	0.101	41 (79)	55 (59)	0.38 (0.16, 0.87)	0.018[b]
Baseline SLD by INV, mm, mean (SD)	n=51 125 (59)	n=71 104 (49)	NA	0.034[b]	n=45 131 (45)	n=79 104 (55)	NA	0.004[b]
KEAP1	9 (15)	5 (6)	0.37 (0.092, 1.3)	0.092[a]	3 (6)	7 (7)	1.3 (0.28, 8.2)	1.000
SMARCA	7 (11)	11 (13)	1.2 (0.38, 3.8)	0.805	12 (23)	7 (7)	0.27 (0.084, 0.81)	0.01[b]
ASXL1	8 (13)	11 (13)	1 (0.34, 3.1)	1.000	3 (6)	20 (21)	4.4 (1.2, 24)	0.017[b]
CDKN2A	8 (13)	9 (11)	0.8 (0.25, 2.5)	0.795	10 (19)	6 (6)	0.29 (0.081, 0.95)	0.026[b]
STK11	9 (15)	10 (12)	0.78 (0.26, 2.3)	0.627	9 (17)	7 (7)	0.39 (0.11, 1.3)	0.096[a]

AE, adverse event; Atezo, atezolizumab; bTMB, blood-based tumor mutational burden; Chemo, chemotherapy; INV, investigator assessment; NA, not analyzed; SAE, serious adverse event; SLD, sum of longest diameters.

[a] *P*<0.1 and ≥0.05

[b] *P*<0.05 and ≥0.001

Supplementary Table 5. Comparison of bTMB assessed by CTA vs F1L CDx

F1L CDx	bTMB CTA		
	bTMB ≥16	bTMB <16	Total
bTMB ≥13.6 mut/Mb	203	24	227
bTMB <13.6 mut/Mb	42	258	300
Non-evaluable	3	3	6
Total	248	285	533

bTMB, blood-based tumor mutational burden, CTA, clinical trial assay; F1L CDx, FoundationOne Liquid Companion Diagnostic assay.

Supplementary Table 6. Agreement between the F1L CDx assay and bTMB CTA assay

	Calculation	Agreement, % (95% CI)
**bTMB CTA (bTMB≥16)**	PPA	82.9 (77.6, 87.1)
**bTMB CTA (bTMB<16)**	NPA	91.5 (87.7, 94.2)
**F1L CDx+ (bTMB≥13.6 mut/Mb)**	PPV	89.4 (84.8, 92.8)
**F1L CDx− (bTMB<13.6 mut/Mb)**	NPV	86.0 (81.6, 89.5)
**F1L CDx**	OPA	87.5 (84.4,90.0)

bTMB, blood-based tumor mutational burden, CTA, clinical trial assay; F1L CDx, FoundationOne Liquid Companion Diagnostic assay; NPA, negative percentage agreement; NPV; negative predictive value; OPA, overall percentage agreement; PPA, positive percentage agreement; PPV, positive predictive value.

Supplementary Table 7. Investigators who participated in the Phase 3 BFAST study

Country	Investigators
Argentina	D. Kaen, L. Lupinacci
Australia	B. Hughes, P. Mitchell, N. Pavlakis, N. Singhal
Belgium	L. Decoster, C. Dooms
Brazil	C. H. Barrios, G. Castro, Jr, J. G. Dutra, F. A. Franke
Canada	P. Cheema, S. Cheng, R. El-Maraghi, C. Labbe, N. Leighl, J. Rothenstein, R. Sangha, M. Vincent, S. Yadav
Chile	M. Burotto
Costa Rica	L. Corrales
France	E. Dansin, J. Dutilh, E. Fabre Guillevin, R. Gervais, J. Mazieres, J. Perrin, C. Ricordel
Germany	H. Bischoff, M. Faehling
Hong Kong	C. M. J. Ho
Israel	J. Bar, M. Gottfried, O. Merimsky, M. Moskovitz, N. Peled, A. Zer
Italy	A. Bearz, L. Bonomi, M. Brighenti, D. Cortinovis, M. R. Migliorino, A. Morabito, S. Novello
Japan	K. Chikamori, D. Harada, H. Hayashi, Y. Hosomi, T. Kondo, M. Kondo, M. Nishio, M. Okada, I. Okamoto, A. Ono, R. Saito, S. Sugawara, K. Tajima, S. Takata, K. Takayama, H. Takeoka, H. Tanaka, N. Tokudome, S. Watanabe, M. Yamaguchi
Mexico	J. A. Alatorre Alexander, J. Ramirez Puente, J. Reyes Contreras, J. Saenz
New Zealand	L. Cameron
Peru	L. Mas
Poland	M. Bryl, G. Czyzewicz, R. Dziadziuszko, A. Kazarnowicz, D. Kowalski, A. Szczesna
Republic of Korea	J.- Y. Han, T. M. Kim, H. R. Kim
Russian Federation	M. Dvorkin, V. Moiseenko, A. Smolin, D. Stroyakovskii
Serbia	Z. Andric, M. Rancic, N. Samardzic, B. Zaric
Spain	R. Alvarez, R. Bernabe Caro, M. Cobo-Dols, E. Felip, G. García Ledo, P. Garrido Lopez, I. Gil-Bazo, A. Insa Molla, R. Lopez, T. Moran Bueno, E. Nadal, L. Paz-Ares Rodriguez, M. Provencio Pulla, N. Reguart Aransay, B. Rubio Viqueira
Taiwan	C. Yu
Thailand	P. Danchaivijitr, S. Geater, T. Reungwetwattana
Turkey	C. Arslan, S. Kilickap, O. F. Olmez, A. Sumbul
United States	N. Belman, H. Cheng, D. Daniel, K. Dragnev, M. Johnson, G. Macvicar, J. Reeves, Jr, C. Reynolds, B. Vicuna
