# Supplementary material for: Atezolizumab versus chemotherapy in advanced or metastatic NSCLC with high blood-based tumor mutational burden: primary analysis of BFAST cohort C randomized phase 3 trial
Source: Nat Med. 2022 Aug 22;28(9):1831–9. doi: 10.1038/s41591-022-01933-w (PMC9499854; doi:10.1038/s41591-022-01933-w)
Supplement: Supplementary file 2 — Reporting Summary [file 41591_2022_1933_MOESM2_ESM.pdf]

nature research

Corresponding author(s): Solange Peters

Last updated by author(s): Jun 15, 2022

Reporting Summary

Nature Research wishes to improve the reproducibility of the work that we publish. This form provides structure for consistency and transparency in reporting. For further information on Nature Research policies, see our Editorial Policies and the Editorial Policy Checklist.

Statistics

For all statistical analyses, confirm that the following items are present in the figure legend, table legend, main text, or Methods section.

n/a	Confirmed	
☐	☒	The exact sample size (*n*) for each experimental group/condition, given as a discrete number and unit of measurement
☐	☒	A statement on whether measurements were taken from distinct samples or whether the same sample was measured repeatedly
☐	☒	The statistical test(s) used AND whether they are one- or two-sided<br>*Only common tests should be described solely by name; describe more complex techniques in the Methods section.*
☐	☒	A description of all covariates tested
☐	☒	A description of any assumptions or corrections, such as tests of normality and adjustment for multiple comparisons
☐	☒	A full description of the statistical parameters including central tendency (e.g. means) or other basic estimates (e.g. regression coefficient) AND variation (e.g. standard deviation) or associated estimates of uncertainty (e.g. confidence intervals)
☐	☒	For null hypothesis testing, the test statistic (e.g. *F*, *t*, *r*) with confidence intervals, effect sizes, degrees of freedom and *P* value noted<br>*Give P values as exact values whenever suitable.*
☒	☐	For Bayesian analysis, information on the choice of priors and Markov chain Monte Carlo settings
☐	☒	For hierarchical and complex designs, identification of the appropriate level for tests and full reporting of outcomes
☐	☒	Estimates of effect sizes (e.g. Cohen's *d*, Pearson's *r*), indicating how they were calculated

Our web collection on statistics for biologists contains articles on many of the points above.

Software and code

Policy information about availability of computer code

Data collection	No software was used for the collection of data.
Data analysis	Biomarker exploratory analyses were performed using R (v3.6.1). Clinical analyses were performed using SAS (v9.4).

For manuscripts utilizing custom algorithms or software that are central to the research but not yet described in published literature, software must be made available to editors and reviewers. We strongly encourage code deposition in a community repository (e.g. GitHub). See the Nature Research guidelines for submitting code & software for further information.

Data

Policy information about availability of data

All manuscripts must include a data availability statement. This statement should provide the following information, where applicable:

- Accession codes, unique identifiers, or web links for publicly available datasets
- A list of figures that have associated raw data
- A description of any restrictions on data availability

As this study is ongoing, access to patient-level data from this trial will not be available until at least 18 months after the last patient visit and a clinical study report has been completed. After that time, requests for data will be assessed by an independent review panel, which decides whether or not the data will be provided. Once approved, the data are available for up to 24 months. At the time of writing this request platform is Vivli. https://vivli.org/ourmember/roche/. For up to date details on Roche's Global Policy on the Sharing of Clinical Information and how to request access to related clinical study documents, see here: https://go.roche.com/data_sharing. Anonymized records for individual patients across more than one data source external to Roche cannot be linked due to a potential increase in risk of patient re-identification. The FMI database of known driver mutations can be requested for access by contacting the Foundation Medicine study review committee at src@foundationmedicine.com, where proposals are reviewed monthly and subject to data sharing agreements imposed by Foundation

Medicine until further notice. Figures with associated raw data include main text figures 1-3, main tables 1-3, extended data figures 1-5 and supplementary tables 1-6.

Field-specific reporting

Please select the one below that is the best fit for your research. If you are not sure, read the appropriate sections before making your selection.

☒ Life sciences ☐ Behavioural & social sciences ☐ Ecological, evolutionary & environmental sciences

For a reference copy of the document with all sections, see nature.com/documents/nr-reporting-summary-flat.pdf

Life sciences study design

All studies must disclose on these points even when the disclosure is negative.

Sample size	Approximately 440 patients were planned to be enrolled in the bTMB ≥10 population, including 280 in the bTMB ≥16 population based on a 1-sided significance level of 0.025 each for the comparison in the bTMB ≥10 and bTMB ≥16 populations and 95% power to detect an HR of 0.6 in the bTMB ≥16 population and 0.65 in the bTMB ≥10 population.
Data exclusions	No data were excluded.
Replication	The bTMB assay used was analytically validated previously. No biomarker experiments were repeated or replicated.
Randomization	Patient's randomization and treatment was assigned from the interactive voice or Web-based response system (IxRS). Randomization to one of two treatment cohorts occured in a 1:1 ratio using permuted-block randomization to ensure a balanced assignment to each treatment cohort. Randomization was stratified by the following criteria: biomarker cutoff (bTMB high vs. moderate); ECOG Performance Status (0 vs. 1); tissue availability (yes vs. no); and histology (non-squamous vs. squamous).
Blinding	This is an open-label study in regard to drug administration, though the Sponsor remained blinded to treatment assignment and the associated data until primary analysis.

Reporting for specific materials, systems and methods

We require information from authors about some types of materials, experimental systems and methods used in many studies. Here, indicate whether each material, system or method listed is relevant to your study. If you are not sure if a list item applies to your research, read the appropriate section before selecting a response.

Materials & experimental systems

n/a	Involved in the study	
☒	☐	Antibodies
☒	☐	Eukaryotic cell lines
☒	☐	Palaeontology and archaeology
☒	☐	Animals and other organisms
☐	☒	Human research participants
☐	☒	Clinical data
☒	☐	Dual use research of concern

Methods

n/a	Involved in the study	
☒	☐	ChIP-seq
☒	☐	Flow cytometry
☒	☐	MRI-based neuroimaging

Human research participants

Policy information about studies involving human research participants

Population characteristics	Eligible patients were aged ≥18 years, had previously untreated histologically or cytologically confirmed unresectable Stage IIIB or IV NSCLC according to the American Joint Committee on Cancer Staging version 7, Eastern Cooperative Oncology Group performance status of 0 or 1, measurable disease per Response Evaluation Criteria in Solid Tumors (RECIST) version 1.1, bTMB ≥10 (8.3 mut/Mb) as detected via the bTMB CTA and a treatment-free interval of ≥6 months if they had received prior neoadjuvant or adjuvant treatment. In the ITT patient population (bTMB 10 or greater), as well as the population for primary analysis (bTMB 16 or greater), baseline characteristics were generally well balanced. In the ITT, patients had a median age of 66 years in both arms, were 73%/74% (atezolizumab/chemotherapy) male, 71%/73% White, 2%/<1% Black, 16%/18% Asian, 1%/2% American Indian or Alaska Native, <1%/0% Native Hawaiian or Pacific Islander, and 9%/6% unknown (enrolled in countries that do not report). 72% of patients in both arms had non-squamous histology, 28%/27% had ECOG PS 0, and 74%/78% had tumor tissue available for biomarker testing.
Recruitment	Patients were enrolled at 120 centers across 25 countries, including Argentina, Australia, Belgium, Brazil, Canada, Chile, Costa Rica, France, Germany, Hong Kong, Israel, Italy, Japan, Mexico, New Zealand, Peru, Poland, Republic of Korea, Russian Federation, Serbia, Spain, Taiwan, Thailand, Turkey, and the United States. These sites were selected based on factors such as patient population availability and site staff expertise in lung cancer and the conduct of clinical trials. Patients were recruited

	to be screened and enrolled at the discretion of the Primary Investigator. All patients had central blood-based genomic screening that then informed investigators as to whether their patient was potentially eligible for a treatment cohort. Investigators were responsible for assessing a patient's eligibility to participate in a treatment cohort on the study. Our protocol design allowed for the screening of a representative sample of patients with advanced or metastatic NSCLC.
Ethics oversight	The study protocol was approved by institutional review boards of participating institutions, including the Ontario Cancer Research Ethics Board (OCREB) (Princess Margaret Cancer Center, William Osler Health System Brampton Civic Hospital, and Sunnybrook Health Sciences Center) and the University of Saskatchewan Biomedical Research Ethics Board (Saskatoon Cancer Centre).

Note that full information on the approval of the study protocol must also be provided in the manuscript.

Clinical data

Policy information about clinical studies

All manuscripts should comply with the ICMJE guidelines for publication of clinical research and a completed CONSORT checklist must be included with all submissions.

Clinical trial registration	NCT03178552
Study protocol	The protocol will be provided on an appropriate sharing site if the manuscript is accepted.
Data collection	Data were collected between September 22, 2017 and May 21, 2020 from 472 patients who were enrolled from 120 centers in 25 countries (countries specified above in the "Recruitment" field). The trial and enrollment was considered global and included North America, South/Latin America, Europe, Russia, Asia, Australia/New Zealand, and the Middle East. Academic and community oncology practices participated in the study. From September 22, 2017, to November 21, 2019, 6507 patients were screened, 1437 of whom (22.1%) had a score of bTMB ≥10; 472 patients were enrolled in the intent-to-treat (ITT) population and randomized to atezolizumab (n=234) or chemotherapy (n=237). One patient randomized in error was subsequently excluded from the analysis
Outcomes	The primary endpoint evaluated in the blood-based tumor mutational burden (bTMB) ≥16 population, as determined by the bTMB clinical trial assay, was investigator-assessed (INV) progression-free survival (PFS) defined as time from randomization to disease progression according to RECIST 1.1 or death. Secondary endpoints included overall survival (OS), defined as time from randomization to death from any cause, in the bTMB ≥16 population, INV PFS and OS in the bTMB ≥10 (ITT) population, PFS by independent review, objective response rate and duration of response by INV and independent review.
