# Supplementary material for: Atezolizumab versus chemotherapy in advanced or metastatic NSCLC with high blood-based tumor mutational burden: primary analysis of BFAST cohort C randomized phase 3 trial
Source: Nat Med. 2022 Aug 22;28(9):1831–9. doi: 10.1038/s41591-022-01933-w (PMC9499854; doi:10.1038/s41591-022-01933-w)
Supplement: Supplementary file 3 — BFAST study protocol v.6. [file 41591_2022_1933_MOESM3_ESM.pdf]

PLEASE READ CAREFULLY:

By opening the attached document you agree to the following terms of use:

- You may not use this document or the information contained herein to a regulatory authority in connection with an application for a marketing authorization or any other regulatory submission without the express written consent of Roche. Please contact global.data_sharing@roche.com

- You may not use this document or the information contained herein to identify clinical trial patients.

- You may not copy, reproduce, or make this document available in any manner that would permit a third-party to review or use the document without first agreeing to these terms of use.

PROTOCOL

**TITLE:**	**A PHASE II/III MULTICENTER STUDY EVALUATING THE EFFICACY AND SAFETY OF MULTIPLE TARGETED THERAPIES AS TREATMENTS FOR PATIENTS WITH ADVANCED OR METASTATIC NON–SMALL CELL LUNG CANCER (NSCLC) HARBORING ACTIONABLE SOMATIC MUTATIONS DETECTED IN BLOOD (BFAST: BLOOD FIRST ASSAY SCREENING TRIAL)**
**PROTOCOL NUMBER:**	BO29554
**VERSION NUMBER:**	6
**EUDRACT NUMBER:**	2017-000076-28
**IND NUMBER:**	130232
**TEST PRODUCTS:**	Alectinib (RO5424802), Atezolizumab (RO5541267), Entrectinib (RO7102122), Cobimetinib (RO5514041), Vemurafenib (RO5185426)
**MEDICAL MONITOR:**	[redacted], *MBBS*., Ph.D., *MRCP*.
**SPONSOR:**	F. Hoffmann-La Roche Ltd
**APPROVAL DATE:**	See electronic date stamp below.

FINAL PROTOCOL AMENDMENT APPROVAL

**Date and Time (UTC)**	**Title**	**Approver's Name**
04-Jan-2021 09:54:48	Company Signatory	[redacted]

CONFIDENTIAL

This clinical study is being sponsored globally by F. Hoffmann-La Roche Ltd of Basel, Switzerland. However, it may be implemented in individual countries by Roche's local affiliates, including Genentech, Inc. in the United States. The information contained in this document, especially any unpublished data, is the property of F. Hoffmann-La Roche Ltd (or under its control) and therefore is provided to you in confidence as an investigator, potential investigator, or consultant, for review by you, your staff, and an applicable Ethics Committee or Institutional Review Board. It is understood that this information will not be disclosed to others without written authorization from Roche except to the extent necessary to obtain informed consent from persons to whom the drug may be administered.

"Use of this document is governed by the terms of use on the first page of this document."

PROTOCOL HISTORY

Protocol	
**Version**	**Date Final**
6	See electronic date stamp on title page
5	6 November 2019
4	17 September 2018
3	3 July 2018
2	6 March 2018
1	17 March 2017

"Use of this document is governed by the terms of use on the first page of this document."

PROTOCOL AMENDMENT, VERSION 6: RATIONALE

Protocol BO29554 has been amended primarily to allow for enrollment of a cohort (Cohort F) of approximately 80 patients with epidermal growth factor receptor exon 20 mutation-positive non–small cell lung cancer (NSCLC) to be treated with atezolizumab, bevacizumab, carboplatin, and pemetrexed. Changes to the protocol, along with a rationale for each change, are summarized below:

- Appendix 12, Section 16 (Cohort F: Atezolizumab, Bevacizumab, Carboplatin, and Pemetrexed in Patients with EGFR Exon 20+ NSCLC) has been added to include all of the specific study details for this cohort. Appropriate references to Cohort F have been added throughout the protocol, and subsequent appendices have been renumbered accordingly.
- Notes have been added to the overall Study Design section to reflect the current status of cohorts where enrollment has been completed or paused to provide clarity for investigators and sites when considering whether to screen patients for the trial. In addition, language has been added to the Study Design to note that BFAST blood screening results can be used to identify patients who may potentially qualify for a separate clinical trial. This is intented to optimize clinical trial access for patients who do not have an actionable alteration corresponding to an open cohort in BFAST (i.e., screen fails) who could potentially be directed, with consent, to another clinical trial suited to their genomic screening results (Section 3.1.1).
- The approximated number of patients in the study has been updated from 9000 to 11000. In addition, Table 1 (Current Enrollment Projections by Cohort) has been updated to reflect the adjusted number of patients (Section 3.1.2).
- Language has been added to specify that prior PD-L1 test results, if available, will also be recorded as part of patient medical history and demographic data (Section 4.5.2 and Section 15.8).
- Language has been modified to reflect that the FoundationOne® Liquid Companion Diagnostic (F1LCDx) assay is now FDA-approved and CE-marked (Section 4.5.6).
- The Medical Monitor and contact information has been updated (Section 5.4.1).
- The Bevacizumab Investigator's Brochure has been added to the list of documents for study drug reference (Section 5.7).
- Text has been added to clarify that selected laboratory assessments as part of blood-based NGS ctDNA assay screening could have been collected previously according to the standard of care (Appendix 1).
- Contraception requirements have been added for the IMP used in the new cohort (Cohort F) (Appendix 2).
- Appendix 13 has been revised to indicate that caution should be used when considering atezolizumab for patients who have previously experienced a severe or life-threatening skin adverse reaction while receiving another immunostimulatory anti-cancer agent.

"Use of this document is governed by the terms of use on the first page of this document."

- The protocol has been amended to reflect the most recent safety updates for atezolizumab, with added safety information and/or management guidelines related endocrine events, immune-mediated myocarditis, cytokine-release syndrome, infusion-related reactions, pancreatic events, dermatologic events, immune-mediated myositis, hemophagocytic lymphohistiocytosis (HLH), and/or macrophage activation syndrome (MAS) (Section 13.5.1.1, Section 15.5.1, and Appendix 15).
- For Cohort A (ALK-positive), Appendix 7:
  - Background information has been updated to reflect current clinical trial and approval information (Section 11.1).
  - Text has been added to clarify that patients may continue study treatment with alectinib after disease progression if the patient is perceived to be deriving clinical benefit (Section 11.3).
  - Adverse Events Relating to ALK Inhibitors have been updated to reflect current safety information and to include dysgeusia and alkaline phosphatase increase (Section 11.5.1).
  - Guidelines for Management of Specific Adverse Events with Alectinib have been updated to clarify the management of specific events and to align with the current Alectinib Investigator's Brochure (Section 11.5.2).
  - Text has been modified to clarify assessment timing in the schedule of activities footnotes (Section 11.8).
- For Cohort B (RET-positive), Appendix 8, and Cohort C (blood tumor mutational burden [bTMB]-positive), Appendix 9, text has been modified to clarify assessment timing in the schedule of activities footnotes (Section 12.8 and Section 13.8).
- For Cohort D (ROS1-positive), Appendix 10:
  - Investigator-assessed intracranial tumor response rate by Response Evaluation Criteria In Solid Tumors, Version 1.1 (RECIST v1.1) in patients with measurable CNS disease at baseline has been moved from secondary endpoints to exploratory endpoints to correct its initial misplacement (Section 14.2 and Section 14.6.2.4).
  - Independent review facility (IRF)-assessed intracranial tumor response rate by response assessment in neuro-oncology brain metastases RANO-BM in patients with measurable CNS disease at baseline has been added as an exploratory endpoint to correct its initial misplacement (Section 14.2).
  - Text has been added to clarify that patients may continue study treatment with entrectinib after disease progression if the patient is perceived to be deriving clinical benefit (Section 14.3).
  - Fractures have been added as an identified risk with entrectinib to align with current safety information (Section 14.5.1).
  - Text has been added to clarify that for adverse events of weight gain, dietary adjustments are recommended prior to consideration of entrectinib dose reduction (Section 14.5.3).

"Use of this document is governed by the terms of use on the first page of this document."

- Language has been added to clarify that measureable disease at baseline is assessed by the investigator (Section 4.6.1 and Section 4.6.2.1).
- The timing of the primary analysis has been changed from 8 months after last patient in (LPI) to approximately 14 months from LPI to have a data set consistent with other entrectinib regulatory approvals. The updated timing now allows for 12 months follow-up from the LPI's first post-treatment tumor assessments (Section 14.6.2.1).
- Language has been added to define the time to CNS progression definition and to add that the analysis of CNS progression or response will be performed by both the investigators. All treated patients with measurable disease at baseline will be included in the analysis regardless of their baseline status of CNS metastases (Section 14.6.2.4).
- The Schedule of Activities has been updated to include a "Fall risk factor evaluation and monitoring" assessment. In addition, text has been modified to clarify assessment timing in the schedule of activities footnotes, and a table note has been added to specify that on treatment days, all assessments should be performed prior to dosing, unless otherwise specified (Section 14.9).

- For Cohort E (BRAF V600-positive), Appendix 11:
  - Language has been added to clarify that patients who initially screen fail may qualify for re-screening. In addition, text has been added to clarify that at the discretion of the Investigator and with the Sponsor's approval, patients may continue study treatment after disease progression if the patient is perceived to be deriving clinical benefit (Section 15.3).
  - Additional eligibility criteria regarding systemic immunostimulatory agents have been clarified for sites. In addition, text has been added to exclude patients who have received prior immune checkpoint inhibitors to correct an inadvertent omission from the previous version (Section 15.4.1.2).
  - Language has been added to clarify that evaluations or examinations are performed according to local standard of care (Section 15.4.4.2 and Section 15.4.4.7).
  - The risks associated with cobimetinib plus vemurafenib have been updated with current safety information to include diarrhea (Section 15.5.2.7).
  - The Schedule of Activities has been updated to remove the Run-in Period, Day 21 visit, the primary purpose of the Run-in Period, Day 21 visit was to dispense vemurafenib to patients, and disbursement has been adjusted to provide enough vemurafenib at the Run-in Period Day 15 visit for the entire Run-in Period. Text has also been modified to clarify assessment timing in the schedule of activities footnotes, and a table note has been added to specify that on treatment days, all assessments should be performed prior to dosing, unless otherwise specified. In addition, starting at Cycle 6, the Day 15 visit is no longer required because all relevant activities and assessments can instead be performed on Day 1 of cycles ≥Cycle 6 (Section 15.8).

"Use of this document is governed by the terms of use on the first page of this document."

Additional minor changes have been made to improve clarity and consistency. Substantive new information appears in italics. This amendment represents cumulative changes to the original protocol.

"Use of this document is governed by the terms of use on the first page of this document."

TABLE OF CONTENTS

PROTOCOL AMENDMENT ACCEPTANCE FORM 23

PROTOCOL SYNOPSIS 24

1. BACKGROUND 68
 1.1 Background on Non–Small Cell Lung Cancer 68
 1.2 Driver Mutations and Targeted Therapy in NSCLC 69
 1.3 Blood Tumor Mutational Burden and Immunotherapy in NSCLC 70
 1.4 Background on Study Treatment 71
 1.5 Study Rationale and Benefit–Risk Assessment 71

2. OBJECTIVES AND ENDPOINTS 74

3. STUDY DESIGN 74
 3.1 Description of the Study 74
 3.1.1 Overview of Study Design 74
 3.1.2 Number of Patients 77
 3.2 End of Study and Length of Study 78
 3.3 Rationale for Study Design 78
 3.3.1 Rationale for Biomarker Assessments 80
 3.3.2 Rationale for Patient-Reported Outcome Assessments 80
 3.3.3 Rationale for Natural History Follow-Up 81

4. MATERIALS AND METHODS 81
 4.1 Patients 81
 4.1.1 General Inclusion Criteria 81
 4.1.2 General Exclusion Criteria 83
 4.2 Method of Treatment Assignment 84
 4.3 Study Treatment and Other Treatments Relevant to the Study Design 84
 4.3.1 Study Treatment Formulation, Packaging, and Handling 84
 4.3.2 Study Treatment Dosage, Administration, and Compliance 84

"Use of this document is governed by the terms of use on the first page of this document."

4.3.3 Investigational Medicinal Product Accountability 85
4.3.4 Continued Access to Study Drugs 85
4.4 Concomitant Therapy 86
4.5 Study Assessments 86
4.5.1 Informed Consent Forms and Screening Log 86
4.5.2 Medical History and Demographic Data 87
4.5.3 Physical Examinations 87
4.5.4 Vital Signs 88
4.5.5 Tumor and Response Evaluations 88
4.5.6 Laboratory, Biomarker, and Other Biological Samples 89
4.5.7 Electrocardiograms 93
4.5.8 Left Ventricular Ejection Fraction 93
4.5.9 Patient-Reported Outcomes 93
4.5.9.1 SILC 94
4.5.9.2 EORTC QLQ-C30 94
4.5.9.3 EORTC QLQ-BN20 94
4.5.9.4 EQ-5D-5L 94
4.5.10 Optional Samples for Research Biosample Repository 95
4.5.10.1 Overview of the Research Biosample Repository 95
4.5.10.2 Approval by the Institutional Review Board or Ethics Committee 95
4.5.10.3 Sample Collection 95
4.5.10.4 Confidentiality 96
4.5.10.5 Consent to Participate in the Research Biosample Repository 96
4.5.10.6 Withdrawal from the Research Biosample Repository 97
4.5.10.7 Monitoring and Oversight 97
4.6 Treatment, Patient, Study, and Site Discontinuation 97
4.6.1 Study Treatment Discontinuation 97
4.6.2 Patient Discontinuation from the Study 98
4.6.3 Study Discontinuation 99

"Use of this document is governed by the terms of use on the first page of this document."

4.6.4 Site Discontinuation 99

5. ASSESSMENT OF SAFETY 99

5.1 Safety Plan 99

5.2 Safety Parameters and Definitions 99

5.2.1 Adverse Events 100

5.2.2 Serious Adverse Events (Immediately Reportable to the Sponsor) 100

5.2.3 Adverse Events of Special Interest (Immediately Reportable to the Sponsor) 101

5.2.4 Selected Adverse Events 101

5.3 Methods and Timing for Capturing and Assessing Safety Parameters 101

5.3.1 Adverse Event Reporting Period 102

5.3.2 Eliciting Adverse Event Information 102

5.3.3 Assessment of Severity of Adverse Events 103

5.3.4 Assessment of Causality of Adverse Events 103

5.3.5 Procedures for Recording Adverse Events 104

5.3.5.1 Infusion-Related Reactions (to Parenteral Study Treatment) 104

5.3.5.2 Diagnosis versus Signs and Symptoms 104

5.3.5.3 Adverse Events That Are Secondary to Other Events 104

5.3.5.4 Persistent or Recurrent Adverse Events 105

5.3.5.5 Abnormal Laboratory Values 105

5.3.5.6 Abnormal Vital Sign Values 106

5.3.5.7 Abnormal Liver Function Tests 106

5.3.5.8 Deaths 107

5.3.5.9 Preexisting Medical Conditions 107

5.3.5.10 Lack of Efficacy or Worsening of NSCLC 108

5.3.5.11 Hospitalization or Prolonged Hospitalization 108

5.3.5.12 Adverse Events Associated with an Overdose or Error in Drug Administration 108

5.3.5.13 Patient-Reported Outcome Data 109

5.4 Immediate Reporting Requirements from Investigator to Sponsor 109

"Use of this document is governed by the terms of use on the first page of this document."

5.4.1 Emergency Medical Contacts 110
5.4.2 Reporting Requirements for Serious Adverse Events and Adverse Events of Special Interest 110
5.4.2.1 Events That Occur Prior to Study Drug Initiation 110
5.4.2.2 Events That Occur After Study Drug Initiation 110
5.4.3 Dose-Limiting Toxicities (Immediately Reportable to the Sponsor) 111
5.4.4 Reporting Requirements for Pregnancies 111
5.4.4.1 Pregnancies in Female Patients 111
5.4.4.2 Pregnancies in Female Partners of Male Patients 111
5.4.4.3 Abortions 112
5.4.4.4 Congenital Anomalies/Birth Defects 112
5.5 Follow-Up of Patients after Adverse Events 112
5.5.1 Investigator Follow-Up 112
5.5.2 Sponsor Follow-Up 113
5.6 Adverse Events That Occur after the Adverse Event Reporting Period 113
5.7 Expedited Reporting to Health Authorities, Investigators, Institutional Review Boards, and Ethics Committees 113

6. STATISTICAL CONSIDERATIONS AND ANALYSIS PLAN 114
6.1 Analysis Populations 114
6.2 Determination of Sample Size 114
6.3 Summaries of Conduct of Study 114
6.4 Summaries of Treatment Group Comparability and/or Demographic and Baseline Characteristics 115
6.5 Efficacy Analyses 115
6.6 Patient-Reported Outcomes Analyses 115
6.7 Safety Analyses 116
6.8 Pharmacokinetic Analyses 116
6.9 Immunogenicity analyses 117
6.10 Biomarker Analyses 117
6.11 Natural History Follow-Up Analyses 117
6.12 Interim Analyses 117

"Use of this document is governed by the terms of use on the first page of this document."

7. DATA COLLECTION AND MANAGEMENT 118
7.1 Data Quality Assurance 118
7.2 Electronic Case Report Forms 118
7.3 Electronic Patient-Reported Outcome Data (if applicable) 118
7.4 Source Data Documentation 119
7.5 Use of Computerized Systems 119
7.6 Retention of Records 120

8. ETHICAL CONSIDERATIONS 120
8.1 Compliance with Laws and Regulations 120
8.2 Informed Consent 120
8.3 Institutional Review Board or Ethics Committee 121
8.4 Confidentiality 122
8.5 Financial Disclosure 123

9. STUDY DOCUMENTATION, MONITORING, AND ADMINISTRATION 123
9.1 Study Documentation 123
9.2 Protocol Deviations 123
9.3 Management of Study Quality 123
9.4 Site Inspections 123
9.5 Administrative Structure 124
9.6 Dissemination of Data and Protection of Trade Secrets 124
9.7 Protocol Amendments 125

10. REFERENCES 126

11. COHORT A: ALECTINIB IN PATIENTS WITH ALK+ NSCLC 154
11.1 Background and Rationale: ALK+ Cohort 155
11.2 Objectives and Corresponding Endpoints: ALK+ Cohort 158
11.3 Cohort Design: ALK+ Cohort 159
11.4 Materials and Methods: ALK+ Cohort 160
11.4.1 Patients 160
11.4.1.1 Additional Inclusion Criteria 160

"Use of this document is governed by the terms of use on the first page of this document."

11.4.1.2 Additional Exclusion Criteria 161
11.4.2 Study Treatment 161
11.4.2.1 Formulation, Packaging, and Handling 161
11.4.2.2 Dosage, Administration, and Compliance 162
11.4.3 Concomitant Therapy, Prohibited Food, and Additional Restrictions 162
11.4.3.1 Permitted Therapy 162
11.4.3.2 Prohibited Therapy 163
11.5 Safety Plan: ALK+ Cohort 163
11.5.1 Adverse Events Relating to ALK Inhibitors 163
11.5.2 Guidelines for Management of Specific Adverse Events with Alectinib 167
11.5.3 Adverse Events of Special Interest (Immediately Reportable to the Sponsor) 170
11.5.4 Adverse Event Reporting Period 171
11.6 Statistical Methods: ALK+ Cohort 171
11.6.1 Determination of Sample Size 171
11.6.2 Efficacy Analyses 172
11.6.2.1 Primary Efficacy Endpoint 172
11.6.2.2 Secondary Efficacy Endpoints 172
11.6.2.3 Patient-Reported Outcome Analyses 173
11.6.2.4 Exploratory Analysis 174
11.6.2.5 Interim Analyses 174
11.7 References: ALK+ Cohort 175
11.8 Schedule of Activities: ALK+ Cohort 177

12. COHORT B: ALECTINIB IN PATIENTS WITH RET+ NSCLC 181
12.1 Background and Rationale: RET+ Cohort 182
12.2 Objectives and Corresponding Endpoints: RET+ Cohort 183
12.3 Cohort Design: RET+ Cohort 185
12.4 Materials and Methods: RET+ Cohort 187
12.4.1 Study Treatment 187
12.4.1.1 Formulation, Packaging, and Handling 187
12.4.1.2 Dosage, Administration, and Compliance 187

"Use of this document is governed by the terms of use on the first page of this document."

12.4.2 Concomitant Therapy, Prohibited Food, and Additional Restrictions 190
12.4.2.1 Permitted Therapy 190
12.4.2.2 Prohibited Therapy 191
12.4.2.3 Prohibited Food 192
12.5 Safety Plan: RET+ Cohort 192
12.5.1 Definition of Dose-Limiting Toxicity 192
12.5.2 Dose Modifications 193
12.5.3 Adverse Events Relating to Alectinib 194
12.5.4 Guidelines for Management of Specific Adverse Events with Alectinib 198
12.5.5 Adverse Events of Special Interest (Immediately Reportable to the Sponsor) 200
12.5.6 Adverse Event Reporting Period 201
12.6 Statistical Methods: RET+ Cohort 201
12.6.1 Determination of Sample Size 201
12.6.2 Efficacy Analyses 202
12.6.2.1 Primary Efficacy Endpoint 202
12.6.2.2 Secondary Efficacy Endpoints 202
12.6.2.3 Patient-Reported Outcome Analyses 203
12.6.2.4 Exploratory Analysis 204
12.6.3 Pharmacokinetic Analyses 204
12.6.4 Interim Analysis 205
12.7 References: RET+ Cohort 206
12.8 Schedule of Activities: RET+ Cohort 207

13. COHORT C: ATEZOLIZUMAB VERSUS CHEMOTHERAPY IN PATIENTS WITH BTMB+ NSCLC 212
13.1 Background and Rationale: bTMB+ Cohort 214
13.2 Objectives and Corresponding Endpoints: bTMB+ Cohort 217
13.3 Cohort Design: bTMB+ Cohort 218
13.4 Materials and Methods: bTMB+ Cohort 221
13.4.1 Patients 221
13.4.1.1 Additional Inclusion Criteria 221

"Use of this document is governed by the terms of use on the first page of this document."

13.4.1.2	Additional Exclusion Criteria	223
13.4.2	Method of Treatment Assignment	226
13.4.3	Study Treatment	227
13.4.3.1	Study Treatment Formulation, Packaging, and Handling	227
13.4.3.2	Study Treatment Dosage, Administration, and Compliance	227
13.4.4	Concomitant Therapy and Additional Restrictions	233
13.4.4.1	Permitted Therapy	233
13.4.4.2	Cautionary Therapy for Atezolizumab-Treated Patients	234
13.4.4.3	Prohibited Therapy	234
13.4.5	Cohort Assessments	235
13.4.6	Study Treatment Discontinuation	236
13.5	Assessment of Safety: bTMB+ Cohort	237
13.5.1	Safety Plan	238
13.5.1.1	Risks Associated with Atezolizumab	238
13.5.1.2	Risks Associated with Pemetrexed	238
13.5.1.3	Risks Associated with Gemcitabine	239
13.5.1.4	Risks Associated with Cisplatin	239
13.5.1.5	Risks Associated with Carboplatin	239
13.5.1.6	Dose Modifications	239
13.5.1.7	Management of Atezolizumab-Specific Adverse Events	240
13.5.1.8	Pemetrexed Dose Modifications, Treatment Delays, or Treatment Discontinuation and Management of Specific Adverse Events	241
13.5.1.9	Gemcitabine Dose Modifications, Treatment Delays, or Treatment Discontinuation and Management of Specific Adverse Events	243
13.5.1.10	Non-Hematologic Toxicities	244
13.5.1.11	Cisplatin Dose Modifications, Treatment Delays, or Treatment Discontinuation and Management of Specific Adverse Events	245

"Use of this document is governed by the terms of use on the first page of this document."

13.5.1.12 Carboplatin Dose Modifications, Treatment Delays, or Treatment Discontinuation and Management of Specific Adverse Events 247
13.5.2 Safety Parameters and Definitions 249
13.5.2.1 Adverse Events of Special Interest (Immediately Reportable to the Sponsor) 249
13.5.2.2 Adverse Event Reporting Period 250
13.6 Statistical Considerations: bTMB+ Cohort 250
13.6.1 Determination of Sample Size 251
13.6.2 Efficacy Analyses 254
13.6.2.1 Primary Efficacy Endpoint and Hypothesis Testing 254
13.6.2.2 Secondary Efficacy Endpoints 255
13.6.2.3 Patient-Reported Outcome Analyses 257
13.6.2.4 Exploratory Analysis 258
13.6.3 Interim Analysis 259
13.6.3.1 Planned Interim Analysis 259
13.6.3.2 Optional Interim Analysis 260
13.7 References: bTMB+ Cohort 261
13.8 Schedule of Activities: bTMB+ Cohort 264

14. COHORT D: ENTRECTINIB IN PATIENTS WITH ROS1+ NSCLC 268
14.1 Background and Rationale: ROS1+ Cohort 269
14.2 Objectives and Corresponding Endpoints: ROS1+ Cohort 271
14.3 Cohort Design: ROS1+ Cohort 272
14.4 Materials and Methods 273
14.4.1 Patients 273
14.4.1.1 Additional Inclusion Criteria 274
14.4.1.2 Additional Exclusion Criteria 274
14.4.2 Study Treatment 275
14.4.2.1 Formulation, Packaging, and Handling 275
14.4.2.2 Dosage, Administration, and Compliance 276
14.4.3 Concomitant Therapy, Prohibited Food, and Additional Restrictions 277

"Use of this document is governed by the terms of use on the first page of this document."

14.4.3.1 Permitted Therapy 277
14.4.3.2 Prohibited Therapy 279
14.4.3.3 Prohibited Food 281
14.5 Safety Plan: ROS1+ Cohort 281
14.5.1 Identified Risks with Entrectinib 281
14.5.2 Guidelines for Management of Specific Adverse Events 282
14.5.3 Dose Modifications 283
14.5.4 Adverse Events of Special Interest (Immediately Reportable to the Sponsor) 286
14.5.5 Adverse Event Reporting Period 286
14.6 Statistical Methods: ROS1+ Cohort 287
14.6.1 Determination of Sample Size 287
14.6.2 Efficacy Analysis 287
14.6.2.1 Primary Efficacy Endpoint: Objective Response Rate 287
14.6.2.2 Secondary Efficacy Endpoints 288
14.6.2.3 Patient-Reported Outcomes 289
14.6.2.4 Exploratory Analysis 290
14.6.3 Pharmacokinetic Analyses 291
14.7 Interim Analyses 292
14.8 References 293
14.9 Schedule of Activities: ROS1+ Cohort 294
14.10 Schedule of Entrectinib Pharmacokinetic Assessments 298

15. COHORT E: ATEZOLIZUMAB, VEMURAFENIB, AND COBIMETINIB IN PATIENTS WITH BRAF V600+ NSCLC 299
15.1 Background and Rationale: BRAF V600+ Cohort 302
15.2 Objectives and Corresponding Endpoints: BRAF V600+ Cohort 308
15.3 Cohort Design: BRAF V600+ Cohort 310
15.4 Materials and Methods 311
15.4.1 Patients 311
15.4.1.1 Additional Inclusion Criteria 311

"Use of this document is governed by the terms of use on the first page of this document."

15.4.1.2 Additional Exclusion Criteria ... 312
15.4.2 Study Treatment ... 317
15.4.2.1 Formulation, Packaging, and Handling ... 317
15.4.2.2 Dosage, Administration, and Compliance ... 318
15.4.3 Concomitant Therapy, Prohibited Food, and Additional Restrictions ... 319
15.4.3.1 Permitted Therapy ... 319
15.4.3.2 Prohibited Therapy ... 320
15.4.3.3 Cautionary Therapy ... 321
15.4.3.3.1 Medications Given with Precaution Due to Effects Related to CYP Enzymes ... 322
15.4.3.3.2 Medications Given with Precaution Due to Effects Related to QT Prolongation ... 323
15.4.3.3.3 Herbal Therapies ... 324
15.4.3.4 Prohibited Food ... 324
15.4.4 Cohort-Specific Safety Assessments ... 324
15.4.4.1 Monitoring for New Primary Neoplasms ... 324
15.4.4.2 Dermatologic Evaluation ... 325
15.4.4.3 Head and Neck Evaluation ... 325
15.4.4.4 Lung Examination ... 325
15.4.4.5 Anal Examination ... 325
15.4.4.6 Gynecological Examination ... 326
15.4.4.7 Ophthalmologic Examinations ... 326
15.5 Safety Plan: BRAF+ Cohort ... 326
15.5.1 Risks Associated with Atezolizumab ... 327
15.5.2 Risks Associated with Cobimetinib ... 327
15.5.2.1 Serous Retinopathy ... 327
15.5.2.2 Left Ventricular Dysfunction ... 328
15.5.2.3 Photosensitivity (When Administered with Vemurafenib) ... 328
15.5.2.4 Pneumonitis ... 328
15.5.2.5 Rhabdomyolysis and CPK Elevations ... 328
15.5.2.6 Hemorrhage ... 329
15.5.2.7 Diarrhea ... 329

"Use of this document is governed by the terms of use on the first page of this document."

15.5.2.8 Severe Hepatotoxicity (Grade 3 or Higher).......................... 329
15.5.2.9 Impaired Female Fertility 329
15.5.2.10 Teratogenicity and Developmental Toxicity 330
15.5.3 Risks Associated with Vemurafenib.................................... 330
15.5.3.1 Cutaneous Squamous Cell Carcinoma and New Primary Melanoma 330
15.5.3.2 Non-Cutaneous Squamous Cell Carcinoma 331
15.5.3.3 RAS-Mutant Malignancies 331
15.5.3.4 Photosensitivity and Sunburn 331
15.5.3.5 Liver Injury 331
15.5.3.6 QT Prolongation 331
15.5.3.7 Hypersensitivity and Severe Cutaneous Reactions... 332
15.5.3.8 Uveitis and Retinal Vein Occlusion 332
15.5.3.9 VIIth Nerve Paralysis 332
15.5.3.10 Radiation Recall and Radiation Sensitization 333
15.5.3.11 Acute Kidney Injury and Renal Function Alterations.. 333
15.5.3.12 Drug–Drug Interaction 333
15.5.3.13 Gastrointestinal Polyps 333
15.5.3.14 Neutropenia 333
15.5.3.15 Pancreatitis.. 333
15.5.3.16 Dupuytren Contracture and Plantar Fascial Fibromatosis 334
15.5.4 Potential Risks Associated with Combination Use of Atezolizumab, Cobimetinib, and Vemurafenib 334
15.5.4.1 Gastrointestinal Toxicity... 334
15.5.4.2 Hepatic Toxicity 335
15.5.4.3 Dermatologic Toxicity 335
15.5.4.4 Pancreatic Events.. 335
15.5.4.5 Renal Events 336
15.5.4.6 Hyperglycemia 336
15.5.4.7 Ocular Toxicity 336
15.5.4.8 Pulmonary Toxicity 336

"Use of this document is governed by the terms of use on the first page of this document."

15.5.5 Management of Patients Who Experience Specific Adverse Events ... 337
15.5.5.1 Dose Modifications during the Run-In Period ... 337
15.5.5.2 Dose Modifications During the Triplet Treatment Period ... 337
15.5.5.3 Treatment Interruption or Discontinuation ... 338
15.5.5.4 Management Guidelines ... 338
15.5.6 Adverse Events of Special Interest (Immediately Reportable to the Sponsor) ... 363
15.5.7 Adverse Event Reporting Period ... 364
15.6 Statistical Methods: BRAF V600+ Cohort ... 364
15.6.1 Determination of Sample Size ... 364
15.6.2 Efficacy Analysis ... 365
15.6.2.1 Primary Efficacy Endpoint: 12-Month Time in Response ... 365
15.6.2.2 Secondary Efficacy Endpoints ... 365
15.6.2.3 Exploratory Efficacy Endpoint ... 367
15.6.2.4 Patient-Reported Outcomes ... 367
15.6.3 Pharmacokinetic Analyses ... 368
15.6.4 Immunogenicity Analyses ... 368
15.6.5 Interim Analyses ... 368
15.7 References ... 370
15.8 Schedule of Activities: BRAF V600+ Cohort ... 374
15.9 Schedule of Pharmacokinetic and Immunogenicity Assessments: BRAF V600+ Cohort ... 382
16. COHORT F: ATEZOLIZUMAB, BEVACIZUMAB, CARBOPLATIN, AND PEMETREXED IN PATIENTS WITH EGFR EXON 20+ NSCLC ... 383
16.1 Background and Rationale ... 383
16.2 Objectives and Corresponding Endpoints ... 387
16.3 Cohort Design ... 389
16.4 Materials and Methods ... 391
16.4.1 Patients ... 391
16.4.1.1 Additional Inclusion Criteria ... 391

"Use of this document is governed by the terms of use on the first page of this document."

16.4.1.2 Additional Exclusion Criteria 393
16.4.2 Study Treatment 397
16.4.2.1 Formulation, Packaging, and Handling 397
16.4.2.2 Dosage, Administration, and Compliance 398
16.4.2.3 Atezolizumab 401
16.4.2.4 Bevacizumab, Carboplatin, and Pemetrexed 403
16.4.2.5 Additional Required Medications 405
16.4.3 Concomitant Therapy, Prohibited Food, and Additional Restrictions 405
16.4.3.1 Permitted Therapy 405
16.4.3.2 Prohibited Therapy 406
16.4.3.3 Cautionary Therapy 407
16.4.4 Study Treatment Discontinuation 407
16.5 Safety Plan 408
16.5.1 Risks Associated with Atezolizumab 409
16.5.2 Risks Associated with Bevacizumab 409
16.5.3 Risks Associated with Carboplatin 410
16.5.4 Risks Associated with Pemetrexed 410
16.5.5 Dose Modifications 410
16.5.5.1 General Considerations 410
16.5.5.2 Atezolizumab Dose Modification 411
16.5.6 Management of Atezolizumab-Specific Adverse Events 411
16.5.7 Bevacizumab Dose Modification and Management of Specific Adverse Events 411
16.5.8 Carboplatin and Pemetrexed Dose Modification and Management of Specific Adverse Events 415
16.5.8.1 Pemetrexed Dose Modifications, Treatment Delays, or Treatment Discontinuation and Management of Specific Adverse Events 416
16.5.8.2 Carboplatin Dose Modifications, Treatment Delays, or Treatment Discontinuation and Management of Specific Adverse Events 418
16.5.9 Potential Overlapping Toxicities 420
16.5.10 Safety Parameters and Definitions 420

"Use of this document is governed by the terms of use on the first page of this document."

16.5.10.1 Adverse Events of Special Interest (Immediately Reportable to the Sponsor) ... 420
16.5.11 Adverse Event Reporting Period ... 421
16.6 Statistical Considerations ... 421
16.6.1 Determination of Sample Size ... 421
16.6.2 Efficacy Analyses ... 422
16.6.2.1 Primary Endpoint: Objective Response Rate ... 422
16.6.2.2 Secondary Efficacy Endpoints ... 422
16.6.2.3 Patient-Reported Outcome Analyses ... 423
16.6.2.4 Exploratory Analysis ... 424
16.6.3 Pharmacokinetic Analyses ... 425
16.6.4 Immunogenicity Analyses ... 425
16.7 Interim Analysis ... 425
16.7.1 Planned Interim Analysis ... 425
16.8 References ... 427
16.9 Schedule of Activities ... 431
16.10 Schedule of Pharmacokinetic and Immunogenecity Assessments ... 436

LIST OF TABLES

Table 1 Current Enrollment Projections by Cohort ... 78
Table 2 Adverse Event Severity Grading Scale for Events Not Specifically Listed in NCI CTCAE ... 103

LIST OF FIGURES

Figure 1 Study Schema: Mutation Screening ... 76

LIST OF APPENDICES

Appendix 1 Schedule of Activities: Blood-Based NGS ctDNA Assay Screening ... 129
Appendix 2 Contraception and Pregnancy Reporting Requirements ... 130
Appendix 3 Response Evaluation Criteria in Solid Tumors, Version 1.1 (RECIST v1.1) ... 133

"Use of this document is governed by the terms of use on the first page of this document."

Appendix 4 Eastern Cooperative Oncology Group Performance Status Scale 142
Appendix 5 List of Substrates, Inhibitors, and Inducers of Drug-Metabolizing Enzymes and Transporters 143
Appendix 6 Patient-Reported Outcomes (PRO) Questionnaires 145
Appendix 7 Cohort A: Alectinib in Patients with ALK+ NSCLC 154
Appendix 8 Cohort B: Alectinib in Patients with RET+ NSCLC 181
Appendix 9 Cohort C: Atezolizumab versus Chemotherapy in Patients with bTMB+ NSCLC 212
Appendix 10 Cohort D: Entrectinib in Patients with ROS1+ NSCLC 268
Appendix 11 Cohort E: Atezolizumab, Vemurafenib, and Cobimetinib in Patients with BRAF V600+ NSCLC 299
Appendix 12 Cohort F: Atezolizumab, Bevacizumab, Carboplatin, and Pemetrexed in Patients with EGFR Exon 20+ NSCLC 383
Appendix 13 Preexisting Autoimmune Diseases (Cohorts C, E, and F Only) 437
Appendix 14 Anaphylaxis Precautions (Cohorts C, E, and F Only) 438
Appendix 15 Risks Associated with Atezolizumab and Guidelines for Management of Adverse Events Associated with Atezolizumab 439

"Use of this document is governed by the terms of use on the first page of this document."

PROTOCOL AMENDMENT ACCEPTANCE FORM

**TITLE:**	**A PHASE II/III MULTICENTER STUDY EVALUATING THE EFFICACY AND SAFETY OF MULTIPLE TARGETED THERAPIES AS TREATMENTS FOR PATIENTS WITH ADVANCED OR METASTATIC NON–SMALL CELL LUNG CANCER (NSCLC) HARBORING ACTIONABLE SOMATIC MUTATIONS DETECTED IN BLOOD (BFAST: BLOOD FIRST ASSAY SCREENING TRIAL)**
**PROTOCOL NUMBER:**	BO29554
**VERSION NUMBER:**	6
**EUDRACT NUMBER:**	2017-000076-28
**IND NUMBER:**	130232
**TEST PRODUCTS:**	Alectinib (RO5424802), Atezolizumab (RO5541267), Entrectinib (RO7102122), Cobimetinib (RO5514041), Vemurafenib (RO5185426)
**MEDICAL MONITOR:**	[redacted], *MBBS*., Ph.D., *MRCP*.
**SPONSOR:**	F. Hoffmann-La Roche Ltd

I agree to conduct the study in accordance with the current protocol.

____

Principal Investigator's Name (print)

____ ____________________

Principal Investigator's Signature | Date

Please retain the signed original of this form for your study files. Please return a copy of the signed form as instructed by your local study monitor.

"Use of this document is governed by the terms of use on the first page of this document."

PROTOCOL SYNOPSIS

**TITLE:**	**A PHASE II/III MULTICENTER STUDY EVALUATING THE EFFICACY AND SAFETY OF MULTIPLE TARGETED THERAPIES AS TREATMENTS FOR PATIENTS WITH ADVANCED OR METASTATIC NON–SMALL CELL LUNG CANCER (NSCLC) HARBORING ACTIONABLE SOMATIC MUTATIONS DETECTED IN BLOOD (BFAST: BLOOD FIRST ASSAY SCREENING TRIAL)**
**PROTOCOL NUMBER:**	BO29554
**VERSION NUMBER:**	6
**EUDRACT NUMBER:**	2017-000076-28
**IND NUMBER:**	130232
**TEST PRODUCT:**	Alectinib (RO5424802), Atezolizumab (RO5541267), Entrectinib (RO7102122), Cobimetinib (RO5514041), Vemurafenib (RO5185426)
**PHASE:**	Phase II/III
**INDICATION:**	Non–small cell lung cancer
**SPONSOR:**	F. Hoffmann-La Roche Ltd

Overall Objectives

This study will evaluate the efficacy and safety of multiple therapies that are selected using predictive biomarkers identified via a blood-based next-generation sequencing (NGS) assay in patients with previously untreated advanced non–small cell lung cancer (NSCLC). Specific objectives and corresponding endpoints for the study are outlined for each cohort below.

This study will also characterize the natural history of patients with genomic alteration profiles of interest who do not enroll in any of the above treatment cohorts. Analyses will be exploratory in nature; there is no formal hypothesis testing in the natural history cohort.

Overall Study Design

Description of Study

Study BO29554 (BFAST) is a Phase II/III, global, multicenter, open-label, multi-cohort study designed to evaluate the safety and efficacy of targeted therapies or immunotherapy as single agents or in combination in patients with unresectable, advanced or metastatic NSCLC determined to harbor oncogenic somatic mutations (e.g., ALK, RET) or positive by tumor mutational burden (TMB) assay (above a prespecified cutoff) as identified by a blood-based NGS circulating tumor DNA (ctDNA) assay.

Male and female patients aged $\geq$ 18 years who meet the trial's general inclusion/exclusion criteria will be eligible for blood-based screening. Blood specimens from patients meeting eligibility criteria for the blood-based screening part of the study will be prospectively tested for somatic alterations in a panel of oncogenes, including the targetable oncogenes and TMB.

The overarching structure of the BFAST study is an umbrella screening and interventional study, which will screen patient blood samples for the presence of potentially oncogenic somatic mutations and biomarker positivity by the blood tumor mutational burden (bTMB) assay in NSCLC via blood-based NGS ctDNA assays, and will treat patients with a drug or drug regimen tailored to their results. Initially, three cohorts testing the efficacy and safety of therapy directed at specific mutations or biomarkers (ALK, RET, and biomarker positive for bTMB [bTMB-positive]) were implemented. Additional cohorts will be added to address various

"Use of this document is governed by the terms of use on the first page of this document."

identified somatic mutations or other biomarkers via future protocol amendments. Each cohort may have separate endpoints, screening, and treatment requirements.

Patients will be assigned to the appropriate cohort based on their identified oncogenic somatic mutation(s). If more than one mutation is identified, the priority for cohort assignment will be as follows:

- Line of therapy (cohorts that allow patient enrollment for earlier lines of therapy given preference) where applicable
- Lesser prevalence of mutation or biomarker in the population as follows:

  ROS1 rearrangement (~1%): Cohort D (entrectinib) ***(Note: Cohort D has been closed to further enrollment)***

  BRAF V600 mutation (~1%–2%): Cohort E (vemurafenib, cobimetinib, atezolizumab)

  EGFR exon 20 mutation (~1%–2%): Cohort F (atezolizumab, bevacizumab, carboplatin, pemetrexed)

  RET rearrangement (~1.8%): Cohort B (alectinib) **(Note: Cohort B has been closed to further enrollment.)**

  ALK rearrangement (~5%): Cohort A (alectinib) (**Note: Cohort A has been closed to further enrollment.)**

  bTMB positive (~23%–36%): Cohort C (atezolizumab vs. chemotherapy) ***(Note: Cohort C has been closed to further enrollment)***

- Natural history follow-up ***(Note: as of 26 OCT 2020 , the enrollment into the natural history follow-up cohort has been paused, as the initial goal of enrolling approximately 1,000 patients has been met)***

  Patients who are screened using the ctDNA assay but who do not enroll in a treatment cohort are eligible to begin natural history follow-up (if consent is given) to document their cancer therapies (or therapy types), treatment response, and survival status. *Additional information on other data that were collected as part of initial blood screening process for these patients is available in the body of the protocol.* Patients with an alteration relevant to the BFAST trial or other key alteration profiles of interest (e.g., KRAS+, HER2+, MET+, PIK3CA+, *other EGFR alterations,* other ALK alterations, other RET alterations, other BRAF alterations, or other ROS1 alterations) who pursue therapy or other management options outside of this study for any reason (e.g., failure to meet treatment-specific eligibility criteria, choosing not to enroll, physician decision to pursue alternative therapy, patient experiencing disease progression before they can enroll, etc.) will be the population of interest for this component of the study. Information will be obtained approximately every 3 months during routine doctor or healthcare appointments and/or telephone calls. For patients in the natural history follow-up, a blood sample will be collected at the time of disease progression for blood-based assay testing (if consent is given). These samples may be stored for future exploratory analyses.

Mandatory blood samples at baseline, during therapy (i.e., at each tumor assessment), and at progression of disease, as well as optional tissue (or results from tissue-based testing, when available), will be collected in order to evaluate exploratory prognostic and/or predictive biomarkers, including but not limited to biomarkers related to driver oncogene signaling and NSCLC pathogenesis. For all patients, blood samples will be used as the only means of identifying driver mutations or TMB for inclusion in treatment cohorts, irrespective of tissue availability or tissue testing results. For patients who do not screen positive for an available cohort (or who are enrolled on the study but discontinue study treatment for reasons other than disease progression), or at the time of disease progression on study, treating physicians will have the option to receive access to a report of the FoundationOne® Liquid Companion Diagnostic (F1LCDx) assay results.

To improve patient access to clinical trials and subject to informed consent, central F1LCDx results for patients deemed not eligible for the enrollment in any active BFAST cohort, may be used to determine if patients are potentially eligible for other Sponsor's studies.

The trial is designed to demonstrate clinical efficacy and safety in the target population, though dose-finding may be necessary in certain cohorts. Treatment will be assigned on the basis of

"Use of this document is governed by the terms of use on the first page of this document."

relevant oncogenotype, will have cohort-specific inclusion/exclusion criteria, and unless otherwise specified, will continue until disease progression per Response Evaluation Criteria In Solid Tumors, Version 1.1 (RECIST v1.1), loss of clinical benefit (if applicable), unacceptable toxicity, patient or physician decision to discontinue, or death, whichever occurs first. If the patient discontinues treatment prior to disease progression (because of adverse event, withdrawal of consent to treatment, or other reason), tumor assessment data collection will continue until disease progression per RECIST v1.1, death, withdrawal of consent, or study closure by the Sponsor, whichever occurs first. Follow-up data capture, including survival and subsequent anti-cancer therapies, will continue for each patient until death, loss to follow-up, study discontinuation, or study closure, whichever occurs first. Information regarding the nature and the duration of subsequent therapies will be collected.

Number of Patients

Approximately *11000* patients are anticipated to be screened for driver mutations or bTMB via the blood-based NGS ctDNA assay, and approximately *700* patients are anticipated to be enrolled at approximately 140 sites for treatment within the current *treatment* cohorts of this study.

Cohort	Regimen	Enrollment (n)
Cohort A (ALK+)	Alectinib	87 (actual) [a]
Cohort B (RET+)	Alectinib	8 (actual) [b]
Cohort C (bTMB+)	Atezolizumab or platinum-based chemotherapy	440 (randomized 1:1)
Cohort D (ROS1+)	Entrectinib	*55 (actual)*
Cohort E (BRAF V600+)	Vemurafenib, cobimetinib, atezolizumab	*Approximately* 80
Cohort F (EGFR exon 20+)	*Atezolizumab, Bevacizumab, Carboplatin, Pemetrexed*	*Approximately 80*
Cohort Z	*Natural History*	*Approximately 1000*

[a] Original projected enrollment was 78 patients.

[b] Original projected enrollment was ~52–62 patients, but enrollment has been closed.

The umbrella structure of this study allows for addition of cohorts through protocol amendments to address any of the identified oncogenic targets as assessed by the F1LCDx assay. Additional cohorts may be added as targeted therapies or combinations for additional alterations or other biomarkers become available for clinical testing and/or as cohorts close enrollment for the initially identified target mutations. Any additional cohorts will increase the total sample size and could increase the number of patients screened via the blood-based NGS ctDNA assay.

Target Population: Blood-Based NGS ctDNA Assay Screening

General Inclusion Criteria

Patients must meet the following criteria to be eligible for blood-based NGS ctDNA assay screening:

- Signed informed consent form for the blood-based screening part of the study and willingness to participate in an assigned cohort based on their identified oncogenic somatic mutation(s)
- Histologically or cytologically confirmed diagnosis of unresectable Stage IIIb not amenable to treatment with combined modality chemoradiation (advanced) or Stage IV (metastatic) NSCLC

  Mixed tumors should be categorized according to the predominant cell type.
- No prior systemic treatment for unresectable Stage IIIb or IV NSCLC
- Age $\geq$ 18 years
- Eastern Cooperative Oncology Group (ECOG) Performance Status 0–2
- Measurable disease (as defined by RECIST, v1.1)

"Use of this document is governed by the terms of use on the first page of this document."

- Adequate recovery from most recent systemic or local treatment for cancer
- Adequate organ function, as demonstrated by the following clinical laboratory parameters:

    Hemoglobin $\geq$ 9 g/dL

    ANC $\geq 1.0 \times 10^9$/L

    Platelet count $\geq 75 \times 10^9$/L

    Serum AST and ALT $\leq 2.5 \times$ the upper limit of normal (ULN) or $\leq 5 \times$ ULN for patients with concurrent liver metastases

    Bilirubin $\leq 2 \times$ ULN or $\leq 5 \times$ ULN for patients with concurrent liver metastases

    Serum creatinine $\leq 2 \times$ ULN or creatinine clearance $>$ 45 mL/min

- Life expectancy $\geq$ 12 weeks
- For female patients of childbearing potential and male patients, willingness to use acceptable methods of contraception

Additionally, in order to be enrolled on a treatment cohort of the study, patients must meet the specific criteria for the respective treatment cohort.

<u>General Exclusion Criteria</u>

Patients who meet any of the following criteria will be excluded from participating in the blood-based screening part of the study:

- Inability to swallow oral medication
- Women who are pregnant or lactating
- Symptomatic, untreated CNS metastases
    - Patients with treated and/or asymptomatic brain metastases may still be eligible for treatment on the study depending on individual cohort requirements; see the cohort-specific appendices for details regarding eligibility
- History of malignancy other than NSCLC within 5 years prior to screening, with the exception of malignancies with a negligible risk of metastasis or death (e.g., 5-year OS rate ≥ 90%), such as adequately treated carcinoma in situ of the cervix, non-melanoma skin carcinoma, localized prostate cancer, ductal carcinoma in situ of breast, or Stage I uterine cancer
- Significant cardiovascular disease, such as New York Heart Association cardiac disease (Class II or greater), myocardial infarction, or cerebrovascular accident within 3 months prior to randomization, unstable arrhythmias, or unstable angina

    Patients with known coronary artery disease, congestive heart failure not meeting the above criteria, or known left ventricular ejection fraction $<$ 50% must be on a stable medical regimen that is optimized in the opinion of the treating physician, in consultation with a cardiologist if appropriate.

- Known HIV positivity or AIDS-related illness
- Either a concurrent condition (including medical illness, such as active infection requiring treatment with intravenous antibiotics or the presence of laboratory abnormalities) or history of a prior condition that places the patient at unacceptable risk if he/she were treated with the study drug or confounds the ability to interpret data from the study
- Inability to comply with other requirements of the protocol

Additionally, patients who meet any of the cohort-specific exclusion criteria will be excluded from that respective treatment cohort.

To be enrolled in a treatment cohort, patients must have met and continue to meet all inclusion and exclusion criteria specified above for the blood-based NGS ctDNA assay screening.

End of Study

The end of this study is defined as the date when the last patient, last visit (LPLV) or assessment occurs for the collection of the last data point for the last treatment cohort. Each cohort may end prior to the end of study when all predefined treatment, follow-up, and data collection are completed.

"Use of this document is governed by the terms of use on the first page of this document."

Length of Study

Based on the longitudinal nature of this trial, the projected duration of this study from first patient enrolled to "end of study," including survival follow-up visits conducted by telephone or in the clinic, is expected to be approximately 4–6 years with the current cohorts. However, the umbrella nature of this protocol may provide for an extension of the overall duration of this study to meet the objectives of additional cohorts and/or additional therapies added through protocol amendments, thus changing the assumptions and projections for LPLV.

Investigational and Non-Investigational Medicinal Products

Patients may be assigned to a treatment cohort based on the results of the blood-based screening assay. Because of the differences in study design between the treatment cohorts, investigational and non-investigational medicinal products are summarized separately below.

Consenting patients with a key alteration profile of interest who, for whatever reason, are not enrolled in one of the treatment cohorts will not receive study treatment in this study but will enter natural history follow-up.

Statistical Methods

The statistical considerations are specific to the treatment cohort and are provided separately below.

COHORT A: ALECTINIB IN PATIENTS WITH ALK+ NSCLC

Objectives and Endpoints: Cohort A

EFFICACY OBJECTIVES	
**Primary Efficacy Objective**	**Corresponding Endpoint**
• To evaluate the efficacy of alectinib in patients with ALK+ advanced or metastatic NSCLC as determined by the F1LCDX assay	• Investigator-assessed ORR based on confirmed objective response (indicated by two objective response assessments based on RECIST v1.1 separated by at least 4 weeks)
**Secondary Efficacy Objective**	**Corresponding Endpoints**
• To evaluate the efficacy of alectinib in patients with ALK+ advanced or metastatic NSCLC as determined by the F1LCDX assay	• Investigator-assessed DOR, CBR, and PFS per RECIST v1.1<br>• IRF-assessed ORR, DOR, CBR, and PFS per RECIST v1.1<br>• OS
**SAFETY OBJECTIVES**	
**Safety Objective**	**Corresponding Endpoints**
• To evaluate the safety and tolerability of alectinib	• Incidence, type, and severity of adverse events (based on the NCI CTCAE v4.0), including SAEs and AEs of special interest<br>• Changes in vital signs, physical findings, and clinical laboratory results during and following administration of protocol-specified IMPs

"Use of this document is governed by the terms of use on the first page of this document."

PATIENT-REPORTED OUTCOME OBJECTIVES	
**PRO Objective**	**Corresponding Endpoints**
• To evaluate the impact of alectinib on PROs in patients with ALK+ advanced or metastatic NSCLC as determined by the F1LCDX assay	• Proportion of patients who improved compared with baseline in patient-reported lung cancer symptoms of cough, dyspnea, and chest pain as measured by SILC<br>• TTD in patient-reported lung cancer symptoms of cough, dyspnea, and chest pain, as measured by SILC<br>• Mean change from baseline in HRQoL, patient functioning, and symptoms as measured by the EORTC QLQ-C30 and SILC
• To evaluate and compare patients' health status to generate utility scores for use in economic models for reimbursement	• Health status as assessed by the EQ-5D-5L questionnaire
**BIOMARKER OBJECTIVES**	
**Biomarker Objective**	**Corresponding Endpoint**
• To assess prognostic effect and pharmacodynamics of exploratory biomarkers in blood, and their association with disease status, mechanisms of resistance, and/or response to alectinib	• Relationship between circulating biomarkers related to alectinib efficacy
**EXPLORATORY OBJECTIVES**	
**Exploratory Objectives**	**Corresponding Endpoints**
• To explore the anti-tumor effect of alectinib in patients with CNS disease identified at baseline	• Investigator-assessed ORR per RECIST v1.1 in patients with CNS disease<br>• Investigator-assessed DOR per RECIST v1.1 in patients with CNS disease<br>• Investigator-assessed CBR per RECIST v1.1 in patients with CNS disease
[redacted]	

AE = adverse event; CBR = clinical benefit rate; DOR = duration of response; EORTC = European Organisation for Research and Treatment of Cancer; EQ-5D-5L = EuroQol 5-Dimension, 5-Level questionnaire; F1LCDx = FoundationOne® Liquid Companion Diagnostic (assay); HRQoL = health-related quality of life; IMP = investigational medicinal product; IRF = independent review facility; NCI CTCAE v4.0 = National Cancer Institute Common Terminology Criteria for Adverse Events, Version 4.0; NSCLC = non–small cell lung cancer; ORR = objective response rate; OS = overall survival; PFS = progression-free survival; PRO = patient-reported outcome; QLQ-C30 = Quality of Life Questionnaire Core 30; RECIST v1.1 = Response Evaluation Criteria in Solid Tumors, Version 1.1; SAE = serious adverse event; SILC = Symptoms in Lung Cancer; [redacted] TTD = time to deterioration.

Cohort Design: Cohort A

Description of Cohort

The ALK+ cohort will use a single-arm, open-label study design. Patients will receive alectinib at 600 mg orally BID, taken with food. Patients will be treated until disease progression, unacceptable toxicity, withdrawal of consent, or death. *In general, after* disease progression (as per Response Evaluation Criteria in Solid Tumors, Version 1.1 [RECIST v1.1]), patients should discontinue the study medication and additional treatment decisions will be at the discretion of the investigator according to local practice. *At the discretion of the Investigator and with the Sponsor's approval, patients may continue study treatment with alectinib after disease progression if the patient is perceived to be deriving clinical benefit.*

In case of isolated asymptomatic CNS progression (e.g., new CNS oligometastases), local therapy can be given (e.g., stereotactic radiotherapy or surgery) followed by continuation of alectinib until systemic disease progression and/or symptomatic CNS progression. The decision to continue the treatment beyond isolated, asymptomatic CNS progression is at the investigator's discretion for patients who can continue to benefit from their respective treatment.

"Use of this document is governed by the terms of use on the first page of this document."

Patients who discontinue treatment prior to disease progression (e.g., because of unacceptable toxicity or withdrawal of consent) will continue to be followed until disease progression and for OS regardless of whether they subsequently receive non-study anti-cancer therapy.

Number of Patients

Approximately 78 patients will be enrolled in the ALK+ cohort.

Target Population

To be enrolled in this cohort, patients must have met and continue to meet all eligibility criteria specified above for the assay screening, in addition to the cohort-specific criteria below.

Additional Inclusion Criteria

Patients must meet the following additional criteria for entry in Cohort A:

- Signed cohort-specific informed consent for treatment
- No prior treatment for inoperable Stage IIIB not amenable to treatment with combined modality chemoradiation (advanced) or metastatic non-squamous or squamous NSCLC

    Patients who have received prior neo-adjuvant, adjuvant chemotherapy, radiotherapy, or chemoradiotherapy with curative intent for non-metastatic disease must have experienced a treatment-free interval of at least 6 months from enrollment since the last chemotherapy, radiotherapy, or chemoradiotherapy cycle.

    - ALK-positive, as determined via the F1LCDx assay
- Prior brain metastases or leptomeningeal disease allowed if asymptomatic and diagnosed incidentally at study baseline

    If patients have neurological symptoms or signs due to CNS metastasis, patients need to complete whole brain radiation or stereotactic radiosurgery treatment at least 14 days before enrollment and be clinically stable.
- For women of childbearing potential, a negative serum pregnancy test ≤ 3 days prior to initial cohort treatment

Additional Exclusion Criteria

Patients who meet any of the following additional criteria will be excluded from entry in Cohort A:

- Detection of the following ALK point mutations: I1171N/S, G1202R
- Any gastrointestinal (GI) disorder that may affect absorption of oral medications, such as malabsorption syndrome or status post-major bowel resection
- Liver disease, characterized by any of the following:

    ALT or AST $> 3 \times$ upper limit of normal (ULN) ($\geq 5 \times$ ULN for patients with concurrent liver metastasis) confirmed on two consecutive measurements

    Impaired excretory function (e.g., hyperbilirubinemia) or synthetic function or other conditions of decompensated liver disease such as coagulopathy, hepatic encephalopathy, hypoalbuminemia, ascites, and bleeding from esophageal varices

    Acute viral or active autoimmune, alcoholic, or other types of acute hepatitis
- National Cancer Institute Common Terminology Criteria for Adverse Events (NCI CTCAE) Version 4.0 Grade ≥ 3 toxicities due to any prior therapy (e.g., radiotherapy) (excluding alopecia) that have not shown improvement or are not stable and are considered to interfere with current study medication
- History of organ transplant
- Co-administration of anti-cancer therapy other than that specified for this cohort
- Symptomatic bradycardia
- History of hypersensitivity to any of the additives in the alectinib drug formulation

"Use of this document is governed by the terms of use on the first page of this document."

Investigational Medicinal Products: Cohort A

Test Product (Investigational Drug)

Alectinib comes in a capsule dosage form containing the following active ingredient:

9-ethyl-6,6-dimethyl-8-[4-(morpholin-4-yl) piperidin-1-yl]-11-oxo-6,11-dihydro-5H-benzo[b]carbazole-3-carbonitrile hydrochloride

Each capsule contains 150 mg of alectinib hydrochloride, lactose monohydrate, hydroxypropylcellulose, sodium lauryl sulfate (SLS), magnesium stearate and carboxymethylcellulose calcium, encapsulated in a capsule shell consisting of hypromellose, carrageenan, potassium chloride, titanium dioxide, and carnauba wax.

Alectinib capsules should be stored in accordance with the storage instructions on the label.

Statistical Methods: Cohort A

Primary Analysis

For Cohort A, the primary efficacy endpoint is ORR as assessed by the investigator per RECIST v1.1, defined as proportion of patients with an objective response. An objective response is defined as a complete response (CR) or partial response (PR) per RECIST v1.1. Confirmation of objective response is required (confirmed ≥4 weeks apart in two separate tumor assessments). Patients not meeting this criterion (including patients without a post-baseline tumor assessment) will be considered non-responders. An estimate of ORR and its 95% CI (the Clopper-Pearson method) will be calculated for comparison with the historical ORR for alectinib in ALK inhibitor–naive NSCLC (from Study BO28984 [ALEX trial]). The primary efficacy analysis population is all treated patients with measurable disease at baseline.

For Cohort A, the primary analysis will occur when all treated patients have been followed for at least 8 months; this corresponds to the time window required for four tumor assessments.

Determination of Sample Size

Determination of sample size is based on demonstration of the manner of consistency of data between the two populations selected by two different diagnostics for the same mutations for ALK by observing at least 75% of the known/established ORR.

Assuming the known/established ORR seen with alectinib in Study BO28984 (the ALEX trial, an ongoing study for which the primary analysis has not yet been performed) is 80%, approximately 78 patients are planned to be enrolled in Cohort A to provide an 80% chance that the lower limit of the two-sided 95% CI (using the Clopper-Pearson method) around the point estimate of the ORR in patients selected by the blood-based assay is higher than 60% (75% of the ORR seen with alectinib in the ALEX trial, where patients were selected using tissue-based diagnostic testing).

COHORT B: ALECTINIB IN PATIENTS WITH RET+ NSCLC

Note: Cohort B is closed to further enrollment. Patients already enrolled in Cohort B will continue to undergo treatment and follow-up per protocol.

Objectives and Endpoints: Cohort B

EFFICACY OBJECTIVES	
**Primary Efficacy Objective**	**Corresponding Endpoint**
• To evaluate the efficacy of alectinib in patients with RET+ advanced or metastatic NSCLC as determined by the F1LCDx assay	• Investigator-assessed ORR based on confirmed objective response (indicated by two objective response assessments based on RECIST v1.1 separated by at least 4 weeks)
**Secondary Efficacy Objective**	**Corresponding Endpoints**
• To evaluate the efficacy of alectinib in patients with RET+ advanced or metastatic NSCLC as determined by the F1LCDx assay	• Investigator-assessed DOR, CBR, and PFS per RECIST v1.1<br>• IRF-assessed ORR, DOR, CBR, and PFS per RECIST v1.1<br>• OS

"Use of this document is governed by the terms of use on the first page of this document."

SAFETY OBJECTIVES	
**Safety Objectives**	**Corresponding Endpoints**
• To assess safety and tolerability of alectinib at increasing dose levels in patients with advanced RET+ NSCLC in order to determine the MTD and the RP2D	• DLTs, if any, associated with alectinib at escalating doses
• To assess safety and tolerability of alectinib as a single agent in patients with advanced or metastatic RET+ NSCLC at the RP2D	• Incidence, type, and severity of adverse events (based on the NCI CTCAE v4.0), including SAEs and adverse events of special interest<br>• Changes in vital signs, physical findings, and clinical laboratory results during and following administration of protocol-specified IMPs
**PHARMACOKINETIC OBJECTIVE**	
**Pharmacokinetic Objective**	**Corresponding Endpoints**
• To explore the PK characteristics of alectinib	• PK parameters of alectinib<br>• Population PK analysis for alectinib<br>• Standard PK parameters of alectinib for dose finding
**PATIENT-REPORTED OUTCOME OBJECTIVES**	
**PRO Objectives**	**Corresponding Endpoints**
• To evaluate the impact of alectinib on PROs in patients with RET+ advanced or metastatic NSCLC as determined by the F1LCDx assay	• Proportion of patients who improved compared with baseline in patient-reported lung cancer symptoms of cough, dyspnea, and chest pain as measured by SILC<br>• TTD in patient-reported lung cancer symptoms of cough, dyspnea, and chest pain, as measured by SILC<br>• Mean change from baseline in HRQoL, patient functioning, and symptoms as measured by the EORTC QLQ-C30 and SILC
• To evaluate and compare patients' health status to generate utility scores for use in economic models for reimbursement	• Health status as assessed by the EQ-5D-5L questionnaire
**BIOMARKER OBJECTIVE**	
**Biomarker Objective**	**Corresponding Endpoint**
• To assess prognostic effect and pharmacodynamics of exploratory biomarkers in blood, and their association with disease status, mechanisms of resistance, and/or response to alectinib	• Relationship between circulating biomarkers related to alectinib exposure and efficacy
**EXPLORATORY OBJECTIVES**	
**Exploratory Objectives**	**Corresponding Endpoints**
• To explore the anti-tumor effect of alectinib in patients with CNS disease identified at baseline	• Investigator-assessed ORR per RECIST v1.1 in patients with CNS disease<br>• Investigator-assessed CBR per RECIST v1.1 in patients with CNS disease<br>• Investigator-assessed DOR per RECIST v1.1 in patients with CNS disease

[redacted]

"Use of this document is governed by the terms of use on the first page of this document."

CBR = clinical benefit rate; DLT = dose-limiting toxicity; DOR = duration of response; EORTC = European Organisation for Research and Treatment of Cancer; EQ-5D-5L = EuroQol 5-Dimension, 5-Level questionnaire; F1LCDx = FoundationOne® Liquid Companion Diagnostic (assay); HRQoL= health related quality of life; IMP = investigational medicinal product; IRF = independent review facility; MTD= maximum tolerated dose; NCI CTCAE v4.0 = National Cancer Institute Common Terminology Criteria for Adverse Events, Version 4.0; NSCLC = non–small cell lung cancer; ORR = objective response rate; OS = overall survival; PFS = progression-free survival; PK = pharmacokinetic; PRO = patient-reported outcome; QLQ-C30 = Quality of Life Questionnaire Core 30; RECIST v1.1 = Response Evaluation Criteria in Solid Tumors, Version 1.1; RET = rearranged during transfection; RP2D = recommended Phase II dose; SAE = serious adverse event; SILC = Symptoms in Lung Cancer; [redacted] TTD = time to deterioration.

Cohort Design: Cohort B

Description of Cohort

Cohort B of Study BO29554 (BFAST) will consist of two parts: a Phase I dose-escalation study followed by a Phase II dose-expansion study at the recommended Phase II dose (RP2D).

For the Phase I dose-escalation portion of the study, patients will receive alectinib according to the dose-escalation scheme outlined below. Patients will receive their assigned dose twice daily with food.

Patients will receive alectinib twice daily, taken with food, at the RP2D found in the dose-finding phase of this cohort.

Patients will be treated until disease progression, unacceptable toxicity, withdrawal of consent, or death. After disease progression (as per Response Evaluation Criteria in Solid Tumors, Version 1.1 [RECIST v1.1]), patients should discontinue the study medication and will be treated at the discretion of the investigator according to local practice. Information regarding the nature and the duration of subsequent therapies will be collected.

In case of isolated asymptomatic CNS progression (e.g., new CNS oligometastases), local therapy can be given (e.g., stereotactic radiotherapy or surgery) followed by continuation of either alectinib until systemic disease progression and/or symptomatic CNS progression. The decision to continue the treatment beyond isolated, asymptomatic CNS progression is at the investigator's discretion for patients who can continue to benefit from their respective treatment.

Patients who discontinue treatment prior to disease progression (e.g., due to unacceptable toxicity or withdrawal of consent) will continue to be followed until disease progression and for overall survival (OS) regardless of whether they subsequently receive non-study anti-cancer therapy. Data for subsequent therapy will be collected for the analysis of OS.

If the RP2D is established in a separate study (IND 132210; [redacted], M.D., Ph.D.; alectinib [AF 802; RO5424802]; RET-rearranged NSCLC or RET-mutated thyroid cancer), the Phase I dose-escalation portion of BFAST will potentially be omitted or abbreviated, and patients will be enrolled in the Phase II expansion cohort from that point.

Number of Patients

Enrollment of Cohort B has been closed by the Sponsor; 8 patients have been enrolled. (Originally, up to 12 patients were to be enrolled in the Phase I dose-escalation portion of the study to determine the RP2D, and approximately 50 patients were to be enrolled in the Phase II dose-expansion portion of the study at the RP2D).

Target Population

To be enrolled in this cohort, patients must have met and continue to meet all eligibility criteria specified above for the assay screening, in addition to the cohort-specific criteria below.

Additional Inclusion Criteria

Patients must meet the following additional criteria for entry in Cohort B:

- Signed cohort-specific informed consent for treatment
- No prior treatment for inoperable Stage IIIB not amenable to treatment with combined modality chemoradiation (advanced) or metastatic non-squamous or squamous NSCLC

    Patients who have received prior neo-adjuvant, adjuvant chemotherapy, radiotherapy, or chemoradiotherapy with curative intent for non-metastatic disease must have

"Use of this document is governed by the terms of use on the first page of this document."

experienced a treatment-free interval of at least 6 months from enrollment since the last chemotherapy, radiotherapy, or chemoradiotherapy cycle.

- RET-positive, as determined via the F1LCDx assay

- Prior brain or leptomeningeal metastases are allowed if asymptomatic and diagnosed incidentally at study baseline

  If patients have neurological symptoms or signs due to CNS metastasis, patients need to complete whole brain radiation or stereotactic radiosurgery treatment at least 14 days before enrollment and be clinically stable.

- For women of childbearing potential, a negative serum pregnancy test ≤ 3 days prior to initial cohort treatment

<u>Additional Exclusion Criteria</u>

Patients who meet any of the following additional criteria will be excluded from entry in Cohort B:

- Any gastrointestinal (GI) disorder that may affect absorption of oral medications, such as malabsorption syndrome or status post-major bowel resection

- Liver disease, characterized by <u>any</u> of the following:

  ALT or AST $> 3 \times$ upper limit of normal (ULN) ($\geq 5 \times$ ULN for patients with concurrent liver metastasis) confirmed on two consecutive measurements

  Impaired excretory function (e.g., hyperbilirubinemia) or synthetic function or other conditions of decompensated liver disease such as coagulopathy, hepatic encephalopathy, hypoalbuminemia, ascites, and bleeding from esophageal varices

  Acute viral or active autoimmune, alcoholic, or other types of acute hepatitis

- National Cancer Institute Common Terminology Criteria for Adverse Events (NCI CTCAE), Version 4.0 Grade ≥ 3 toxicities due to any prior therapy (e.g., radiotherapy) (excluding alopecia) that have not shown improvement or are considered to interfere with current study medication

- History of organ transplant

- Co-administration of anti-cancer therapy other than that specified for this cohort

- Symptomatic bradycardia

- Administration of strong/potent cytochrome P450 (CYP)3A inhibitors or inducers within 14 days prior to the first dose of study treatment and while on treatment with alectinib

  No CYP3A inhibitors or inducers of any potency are permitted during the pharmacokinetic (PK) period for the dose-finding cohort (if needed).

- History of hypersensitivity to any of the additives in the alectinib drug formulation

<u>Investigational Medicinal Products: Cohort B</u>

Test Product (Investigational Drug)

Alectinib comes in a capsule dosage form containing the following active ingredient:

9-ethyl-6,6-dimethyl-8-[4-(morpholin-4-yl) piperidin-1-yl]-11-oxo-6,11-dihydro-5H-benzo[b]carbazole-3-carbonitrile hydrochloride

Each capsule contains 150 mg of alectinib hydrochloride, lactose monohydrate, hydroxypropylcellulose, sodium lauryl sulfate (SLS), magnesium stearate and carboxymethylcellulose calcium, encapsulated in a capsule shell consisting of hypromellose, carrageenan, potassium chloride, titanium dioxide, and carnauba wax.

Alectinib capsules should be stored in accordance with the storage instructions on the label.

<u>Statistical Methods: Cohort B</u>

Primary Analysis

For Cohort B, the primary efficacy endpoint is ORR as assessed by the investigator per RECIST v1.1, defined as proportion of patients with an objective response. An objective response is

"Use of this document is governed by the terms of use on the first page of this document."

defined as a complete response (CR) or partial response (PR) per RECIST v1.1. Confirmation of objective response is required (confirmed ≥ 4 weeks apart in two separate tumor assessments). Patients not meeting this criterion (including patients without a post-baseline tumor assessment) will be considered non-responders. An estimate of ORR and its 95% CI (the Clopper-Pearson method) will be calculated for comparison with the historical ORR with chemotherapy. The primary efficacy analysis population is all-treated patients with measurable disease at baseline.

For Cohort B, the primary analysis will occur when all treated patients have been followed for at least 8 months; this corresponds to the time window required for four tumor assessments.

Determination of Sample Size

In the Phase I dose-escalation portion of Cohort B, 6 patients are planned to be enrolled in each dose-finding cohort, with expansion of the RP2D cohort to a total of approximately 50 patients to ensure sufficient power such that the lower limit of the two-sided 95% CI around the point estimate of the ORR is higher than historical control (first-line chemotherapy in NSCLC).

With 50 patients, an ORR of 50% (25 of 50 patients) would have a lower limit of the two-sided 95% CI of 36%, which would be considered a clinically meaningful improvement over the established standard-of-care therapies in this population. In addition, with 50 patients, there is 60% chance of detecting a 15% increase in ORR from 35% for historical-control chemotherapy to 50% at the 5% two-sided significance level.

Interim Analysis

Limited data are available regarding the natural history of RET+ NSCLC and responsiveness of these patients to kinase inhibitors targeting RET. Therefore, data from the first 20 patients treated at the RP2D will be used for the nonbinding futility interim analysis based on ORR calculated by investigator-assessed radiographs. This interim analysis will be utilized to identify whether the treatment (at RP2D) is ineffective (i.e., futility analysis) due to lack of efficacy so that further accrual to such a cohort may be stopped.

Only patients with at least two tumor assessments will be considered eligible for the ORR analysis. Enrollment should not stop while awaiting the ORR results of the first 20 patients treated at the RP2D. For the interim analysis, CR and PR need to be confirmed. If the ORR is < 30% (i.e., fewer than 6 of 20 patients achieve a CR or PR), the cohort may be considered futile and may be terminated. If ORR is ≥ 30%, the cohort may continue to enroll a total of approximately 50 patients at the RP2D.

An Internal Monitoring Committee (IMC) will conduct the futility analysis. The futility methodology, as well as membership of the IMC, will be described in an IMC Charter.

"Use of this document is governed by the terms of use on the first page of this document."

COHORT C: ATEZOLIZUMAB VERSUS CHEMOTHERAPY IN PATIENTS WITH bTMB+ NSCLC

Objectives and Endpoints: Cohort C

EFFICACY OBJECTIVES	
**Primary Efficacy Objective**	**Corresponding Endpoint**
• To evaluate the efficacy of atezolizumab compared with platinum-based chemotherapy consisting of a platinum agent (cisplatin or carboplatin) in combination with either pemetrexed (non-squamous disease) or gemcitabine (squamous disease) in chemotherapy-naive patients with inoperable Stage IIIB or Stage IV NSCLC in patients who are biomarker positive by the bTMB assay	• Investigator-assessed PFS according to RECIST v1.1 in bTMB PP1
**Secondary Efficacy Objective:**	**Corresponding Endpoints**
• To evaluate the efficacy of atezolizumab compared with platinum-based chemotherapy	• OS in bTMB PP1<br>• Investigator-assessed PFS according to RECIST v1.1 in bTMB PP2<br>• OS in bTMB PP2<br>• IRF-assessed PFS, ORR, and DOR according to RECIST v1.1<br>• Investigator-assessed ORR, and DOR according to RECIST v1.1<br>• Investigator-assessed PFS rates at 6-month and 1-year landmark timepoints
**SAFETY OBJECTIVE**	
**Safety Objective**	**Corresponding Endpoint**
• To evaluate the safety and tolerability of atezolizumab compared with platinum-based chemotherapy	• Incidence, type, and severity of adverse events (based on NCI CTCAE v4.0), including SAEs and adverse events of special interest (as identified for individual cohorts)
**EXPLORATORY OBJECTIVE**	
**Exploratory Objective**	**Corresponding Endpoints**
• To evaluate the efficacy of atezolizumab compared with platinum-based chemotherapy	• Investigator-assessed TTR, [redacted], and DCR according to RECIST v1.1<br>• OS rate at 1 and 2 years in each treatment arm
**BIOMARKER OBJECTIVE**	
**Biomarker Objective**	**Corresponding Endpoints**
• To assess predictive, prognostic, and pharmacodynamic exploratory biomarkers in blood, and their association with disease status, mechanisms of resistance, and/or response to atezolizumab	• Relationship between circulating biomarkers related to atezolizumab efficacy

"Use of this document is governed by the terms of use on the first page of this document."

PATIENT REPORTED OUTCOME OBJECTIVES	
**PRO Objective**	**Corresponding Endpoints**
• To determine the impact of atezolizumab on PROs compared with platinum-based chemotherapy	• TTD and change from baseline in patient-reported lung cancer symptom (cough, dyspnea, chest pain) score as assessed by the SILC<br>• Change from baseline in PROs of HRQoL, symptoms, and functioning as assessed by the EORTC QLQ-C30
• To evaluate and compare patients' health status to generate utility scores for use in economic models for reimbursement	• Health status as assessed by the EQ-5D-5L questionnaire

bTMB = blood tumor mutational burden; bTMB PP1 = the primary population of patients with a bTMB level equal to or greater than the higher validated cutoff; bTMB PP2 = the secondary population of all patients who are bTMB-positive, which is the intent-to-treat (ITT) population in this cohort; DCR = disease control rate; DOR = duration of response; EORTC = European Organisation for Research and Treatment of Cancer; EQ-5D-5L = EuroQol 5-Dimension, 5-Level questionnaire; HRQoL = health related quality of life; IRF = independent review facility; NCI CTCAE v4.0 = National Cancer Institute Common Terminology Criteria for Adverse Events, Version 4.0; NSCLC = non-small cell lung cancer; ORR = objective response rate; OS = overall survival; PFS = progression-free survival; PRO = patient-reported outcome; QLQ-C30 = Quality of Life Questionnaire Core 30; RECIST v1.1 = Response Evaluation Criteria in Solid Tumors, Version 1.1; SAE = serious adverse event; SILC = Symptoms in Lung Cancer; [redacted]; TTD = time to deterioration; TTR = time to response.

Cohort Design: Cohort C

Description of Cohort

This is a randomized, open-label cohort designed to evaluate the safety and efficacy of atezolizumab compared with chemotherapy consisting of a platinum agent (cisplatin or carboplatin per investigator discretion) combined with either pemetrexed (non-squamous disease) or gemcitabine (squamous disease) in patients who are biomarker positive by the bTMB assay.

To assure balance during randomization, a number of stratification factors will be used. Histology and Eastern Cooperative Oncology Group (ECOG) Performance Status will be used, as they are accepted to have potential prognostic implications. Because two distinct cutoffs will be evaluated for bTMB, patients will be stratified according to whether they are considered "bTMB moderate" or "bTMB high". Finally, as the trial has the potential to enroll all patients with NSCLC, including patients without tissue, the availability of tissue will be used as an additional stratification factor to prevent an imbalance between those patients with and without tissue, in the event this is prognostic.

Given the toxicities associated with platinum-based chemotherapies (e.g., neutropenia, anemia) and the requirement for pre-medications, this will be an open-label study. No crossover will be allowed from the control arm (platinum-based chemotherapy) to the experimental arm (atezolizumab).

Atezolizumab (fixed dose of 1200 mg) will be administered intravenously on Day 1 of each 21-day cycle. Atezolizumab treatment may continue as long as patients are experiencing clinical benefit as assessed by the investigator (i.e., in the absence of unacceptable toxicity or symptomatic deterioration attributed to disease progression as determined by the investigator after an integrated assessment of radiographic data, biopsy results [if available], and clinical status) or until unacceptable toxicity or death.

During treatment, patients who are treated with atezolizumab and who show evidence of clinical benefit may be permitted to continue atezolizumab treatment after Response Evaluation Criteria in Solid Tumors, Version 1.1 (RECIST v1.1) criteria for progressive disease are met if they meet all of the following criteria:

- Evidence of clinical benefit as assessed by the investigator
- Absence of symptoms and signs (including worsening of laboratory values [e.g., new or worsening hypercalcemia]) indicating unequivocal progression of disease
- No decline in ECOG Performance Status that can be attributed to disease progression

"Use of this document is governed by the terms of use on the first page of this document."

- Absence of tumor progression at critical anatomical sites (e.g., leptomeningeal disease) that cannot be managed by protocol-allowed medical interventions
- Patients must provide written consent to acknowledge deferring other treatment options in favor of continuing study treatment at the time of initial radiographic progression per RECIST v1.1

Patients randomized to receive pemetrexed in combination with either cisplatin or carboplatin (non-squamous disease) will receive chemotherapy intravenously on Day 1 of each 21-day cycle for four or six cycles as per local standard of care, followed by maintenance therapy with pemetrexed as per local standard of care. Patients randomized to receive gemcitabine in combination with either cisplatin or carboplatin (squamous disease) will receive cisplatin or carboplatin intravenously on Day 1 and gemcitabine intravenously on Days 1 and 8 of each 21-day cycle for four or six cycles, as per local standard of care, followed by best supportive care. The intended number of cycles planned for the platinum-based induction chemotherapy (i.e., four or six cycles) will be specified by the investigator prior to study randomization. Treatment will continue until disease progression, unacceptable toxicity, or death.

All patients will undergo tumor assessment at baseline and every 6 weeks (± 7 days) for 48 weeks following Cycle 1, Day 1 regardless of treatment delays. After the completion of the Week 48 tumor assessment, tumor assessment will be required every 9 weeks (± 7 days) regardless of treatment delays, until radiographic disease progression per RECIST v1.1 (or loss of clinical benefit for atezolizumab-treated patients who continue treatment beyond disease progression according to RECIST v1.1), withdrawal of consent, death, or study termination by the Sponsor, whichever occurs first. Patients who discontinue treatment for reasons other than disease progression (e.g., toxicity) will continue scheduled tumor assessments until disease progression per RECIST v1.1, withdrawal of consent, death, or study termination by Sponsor, whichever occurs first. In the absence of disease progression, tumor assessments should continue regardless of whether patients start a new anti-cancer therapy.

A secondary endpoint of this study is independent review facility (IRF)-assessed PFS according to RECIST v1.1. Therefore, an IRF will conduct an independent review of the responses of all patients, including a review of blinded computed tomography (CT) scans. All primary imaging data used for tumor assessment will be collected by the Sponsor to enable centralized, independent review of response endpoints. These reviews will be performed prior to the final efficacy analyses.

Number of Patients

This study will randomize approximately 280 patients in bTMB PP1 (the primary population of patients with a bTMB level equal to or greater than the higher validated cutoff), which will result in the randomization of approximately 440 patients in bTMB PP2 (the secondary population of all patients who are bTMB+ [ITT population]), including the 280 patients in bTMB PP1).

Target Population

To be enrolled into this cohort, patients must have met and continue to meet all eligibility criteria specified for the blood-based next-generation sequencing ctDNA assay screening, in addition to the cohort-specific criteria below.

<u>Additional Inclusion Criteria</u>

Patients must meet the following additional criteria for entry in Cohort C:

- bTMB+, as detected via the blood-based assay
- Signed cohort-specific informed consent for treatment
- ECOG Performance Status of 0 or 1
- For women of childbearing potential, a negative serum pregnancy test ≤ 7 days prior to initial cohort treatment
- No prior treatment for inoperable Stage IIIB not amenable to treatment with combined modality chemoradiation (advanced) or metastatic non-squamous or squamous NSCLC

    Patients who have received prior neo-adjuvant, adjuvant chemotherapy, radiotherapy, or chemoradiotherapy with curative intent for non-metastatic disease must have

"Use of this document is governed by the terms of use on the first page of this document."

experienced a treatment-free interval of at least 6 months from randomization since the last chemotherapy, radiotherapy, or chemoradiotherapy cycle.

- Patients with a history of treated, asymptomatic CNS metastases are eligible, provided they meet all of the following criteria:

    Only supratentorial and cerebellar metastases allowed (i.e., no metastases to midbrain, pons, medulla, or spinal cord)

    No ongoing requirement for corticosteroids as therapy for CNS disease

    No stereotactic radiation within 7 days or whole-brain radiation within 14 days prior to randomization

    No evidence of interim progression between the completion of CNS-directed therapy and the screening radiographic study

        Patients with new asymptomatic CNS metastases detected at the screening scan must receive radiation therapy and/or surgery for CNS metastases. Following treatment, these patients may then be eligible without the need for an additional brain scan prior to randomization, if all other criteria are met.

- Negative HIV test at screening for cohort
- Negative hepatitis B surface antigen (HBsAg) test at screening for cohort
- Negative total hepatitis B core antibody (HBcAb) test at screening for cohort, or positive total HBcAb test followed by a negative hepatitis B virus (HBV) DNA test at screening
    - The HBV DNA test will be performed only for patients who have a positive total HBcAb test.
- Negative hepatitis C virus (HCV) antibody test at screening, or positive HCV antibody test followed by a negative HCV RNA test at screening
    - The HCV RNA test will be performed only for patients who have a positive HCV antibody test.
- Adequate hematologic and end-organ function, defined by the following laboratory test results obtained within 14 days prior to randomization:

    ANC $\geq$ 1500 cells/μL without granulocyte colony-stimulating factor support

    Lymphocyte count $\geq$ 500 cells/μL

    Platelet count $\geq$ 100,000 cells/μL without transfusion

    INR or aPTT $\leq$ 1.5 $\times$ upper limit of normal (ULN)

        This applies only to patients who are not receiving therapeutic anticoagulation; patients receiving therapeutic anticoagulation must have an INR or aPTT within therapeutic limits for at least 1 week prior to randomization.

    Alkaline phosphatase $\leq$ 2.5 $\times$ ULN with the following exceptions:

        Patients with documented liver metastases: Alkaline phosphatase $\leq$ 5 $\times$ ULN

    Serum bilirubin $\leq$ 1.5 $\times$ ULN

        Patients with known Gilbert disease who have serum bilirubin level $\leq$ 3 $\times$ ULN may be enrolled.

Additional Exclusion Criteria

Patients who meet any of the following additional criteria will be excluded from entry in Cohort C:

CANCER-SPECIFIC EXCLUSIONS

- Presence of activating EGFR or ALK genetic alteration considered exclusionary by the blood-based assay (F1LCDx)
    - The F1LCDx assay reports both known and novel alterations. Only patients with known oncogenic alterations in EGFR or ALK will be excluded from BFAST. Alterations in EGFR or ALK where oncogenic potential is unknown (i.e., variants of unknown significance) are not considered exclusionary.

"Use of this document is governed by the terms of use on the first page of this document."

- Spinal cord compression not definitively treated with surgery and/or radiation, or previously diagnosed and treated spinal cord compression without evidence that disease has been clinically stable for ≥ 2 weeks prior to randomization
- Leptomeningeal disease
- Uncontrolled tumor-related pain

  Patients requiring pain medication must be on a stable regimen at study entry.

  Symptomatic lesions amenable to palliative radiotherapy (e.g., bone metastases or metastases causing nerve impingement) should be treated prior to randomization. Patients should be recovered from the effects of radiation. There is no required minimum recovery period.

  Asymptomatic metastatic lesions whose further growth would likely cause functional deficits or intractable pain (e.g., epidural metastasis that is not currently associated with spinal cord compression) should be considered for loco-regional therapy if appropriate prior to enrollment.
- Uncontrolled pleural effusion, pericardial effusion, or ascites requiring recurrent drainage procedures (once monthly or more frequently)

  Patients with indwelling catheters (e.g., PleurX®) are allowed.
- Uncontrolled or symptomatic hypercalcemia ($>$ 1.5 mmol/L ionized calcium or calcium $>$ 12 mg/dL or corrected serum calcium $>$ ULN)

GENERAL MEDICAL EXCLUSIONS

- History of severe allergic, anaphylactic, or other hypersensitivity reactions to chimeric or humanized antibodies or fusion proteins
- Known hypersensitivity to biopharmaceuticals produced in Chinese hamster ovary cells or any component of the atezolizumab formulation
- History of autoimmune disease, including, but not limited to, myasthenia gravis, myositis, autoimmune hepatitis, systemic lupus erythematosus, rheumatoid arthritis, inflammatory bowel disease, vascular thrombosis associated with antiphospholipid syndrome, Wegener granulomatosis, Sjögren syndrome, Guillain-Barré syndrome, multiple sclerosis, vasculitis, or glomerulonephritis

  Patients with a history of autoimmune-related hypothyroidism on thyroid-replacement therapy are eligible for this study.

  Patients with controlled Type I diabetes mellitus on an insulin regimen are eligible for this study.

  Patients with eczema, psoriasis, lichen simplex chronicus, or vitiligo with dermatologic manifestations only (e.g., patients with psoriatic arthritis would be excluded) are permitted provided that they meet the following conditions:

    Rash must cover less than 10% of body surface area.

    Disease is well controlled at baseline and only requiring low potency topical steroids.

    No acute exacerbations of underlying condition within the last 12 months requiring treatment with PUVA [psoralen plus ultraviolet A radiation], methotrexate, retinoids, biologic agents, oral calcineurin inhibitors, or high-potency or oral steroids.
- History of idiopathic pulmonary fibrosis, organizing pneumonia (e.g., bronchiolitis obliterans), drug-induced pneumonitis, idiopathic pneumonitis, or evidence of active pneumonitis on screening chest CT scan

  History of radiation pneumonitis in the radiation field (fibrosis) is permitted.
- Current treatment with anti-viral therapy for HBV
- Active tuberculosis
- Severe infections within 4 weeks prior to randomization, including, but not limited to, hospitalization for complications of infection, bacteremia, or severe pneumonia

"Use of this document is governed by the terms of use on the first page of this document."

- Major surgical procedure other than for diagnosis within 28 days prior to randomization or anticipation of need for a major surgical procedure during the course of the study
- Prior allogeneic bone marrow transplantation or solid organ transplantation
- Any other diseases, metabolic dysfunction, physical examination finding, or clinical laboratory finding giving reasonable suspicion of a disease or condition that contraindicates the use of an investigational drug or that may affect the interpretation of the results or render the patient at high risk from treatment complications
- Patients with illnesses or conditions that interfere with their capacity to understand, follow, and/or comply with study procedures

EXCLUSION CRITERIA RELATED TO MEDICATIONS

- Any approved anti-cancer therapy, including hormonal therapy, within 3 weeks prior to initiation of study treatment
- Treatment with any other investigational agent with therapeutic intent within 28 days prior to randomization
- Receipt of therapeutic oral or IV antibiotics within 2 weeks prior to randomization

    Patients receiving prophylactic antibiotics (e.g., for prevention of a urinary tract infection or to prevent chronic obstructive pulmonary disease exacerbation) are eligible.

- Administration of a live, attenuated vaccine within 4 weeks before randomization or anticipation of need for such a vaccine during atezolizumab treatment or within 5 months after the last dose of atezolizumab
- Prior treatment with CD137 agonists or immune checkpoint blockade therapies, anti–PD-1, and anti–PD-L1 therapeutic antibodies

    Patients who have had prior anti–cytotoxic T lymphocyte–associated antigen 4 (CTLA-4) treatment may be enrolled, provided the following requirements are met:

        Last dose of anti–CTLA-4 at least 6 weeks prior to randomization

        No history of severe immune related adverse effects from anti–CTLA-4 (National Cancer Institute Common Terminology Criteria for Adverse Events [NCI CTCAE] Grade 3 and 4)

- Treatment with systemic immunostimulatory agents (including, but not limited to, interferons or interleukin-2) within 4 weeks or 5 half-lives of the drug, whichever is longer, prior to randomization

    Prior treatment with cancer vaccines is allowed.

- Treatment with systemic corticosteroids or other systemic immunosuppressive medications (including, but not limited to, corticosteroids, cyclophosphamide, azathioprine, methotrexate, thalidomide, and anti–tumor necrosis factor [anti-TNF] agents) within 2 weeks prior to randomization

    Patients who received acute, low-dose, systemic immunosuppressant medication or a one-time pulse dose of systemic immunosuppressant medication (e.g., 48 hours of corticosteroids for a contrast allergy) are eligible for the study after Medical Monitor approval has been obtained.

    The use of corticosteroids ($\leq$ 10 mg oral prednisone or equivalent) for chronic obstructive pulmonary disease, mineralocorticoids (e.g., fludrocortisone) for patients with orthostatic hypotension, and low-dose supplemental corticosteroids for adrenocortical insufficiency are allowed.

EXCLUSION CRITERIA RELATED TO CHEMOTHERAPY

- Hypersensitivity to the active substance or any of the excipients in pemetrexed, gemcitabine, carboplatin, cisplatin, or other platinum-containing compounds
- Patients with hearing impairment (cisplatin)
- Grade $\geq$ 2 peripheral neuropathy as defined by NCI CTCAE v4.0 criteria (cisplatin)
- CrCl $<$ 60 mL/min (cisplatin)

"Use of this document is governed by the terms of use on the first page of this document."

- History of radiation therapy within 7 days prior to initiating gemcitabine

Investigational Medicinal Products: Cohort C

Test Product (Investigational Drug)

Atezolizumab, at a dose of 1200 mg, will be administered by IV infusion every 21 days.

Comparator

Non-Squamous NSCLC

The comparator arm includes pemetrexed + cisplatin or carboplatin to be administered every 21 days for four or six cycles at the doses and the suggested infusion times indicated in the table below. Pemetrexed will continue to be administered as maintenance regimen every 21 days.

Study Drug	Dose/Route	Induction Period (Four or Six Cycles)	Maintenance Period (Until PD)
Pemetrexed	500 mg/m^2 IV	Over ~10 minutes on Day 1 Q21D	Over approximately 10 minutes on Day 1 Q21D
Carboplatin	AUC 5 or 6 IV	Over ~30–60 minutes on Day 1 Q21D	Not applicable
		OR	
Cisplatin	75 mg/m^2	Over 1–2 hours on Day 1 Q21D	Not applicable

AUC = area under the concentration-time curve; IV = intravenous; PD = progressive disease; Q21D = every 21 days.

Squamous NSCLC

The comparator arm includes gemcitabine + cisplatin or carboplatin to be administered every 21 days for four or six cycles at the doses and the suggested infusion times indicated in the table below.

Chemotherapy	Dose/Route	Treatment (Four or Six Cycles)
Gemcitabine	1250 mg/m^2 IV	Over 30 minutes on Days 1 and 8 Q21D
Cisplatin	75 mg/m^2 IV	Over 1–2 hours on Day 1 Q21D
Gemcitabine	1000 mg/m^2 IV	Over 30 minutes on Days 1 and 8 Q21D
Carboplatin	AUC 5 IV	Over approximately 30–60 minutes on Day 1 Q21D

AUC = area under the concentration-time curve; IV = intravenous; Q21D = every 21 days.

Statistical Methods: Cohort C

Primary Analysis

The primary efficacy endpoint is PFS assessed by the investigator using RECIST v1.1 in the bTMB PP1 population.

PFS is defined as the time from the date of randomization to the date of first documented disease progression or death, whichever occurs earlier. Disease progression will be determined on the basis of investigator assessment with use of RECIST v1.1. Data for patients who have not experienced disease progression or death at the time of analysis will be censored at the time of the last tumor assessment. Patients with no post-baseline tumor assessment will be censored at the date of randomization plus 1 day. Type I error control will be applied to this analysis of PFS.

For U.S. registrational purposes, a sensitivity analysis of PFS will be defined as described above with an additional censoring rule for missed visits. Data for patients with a PFS event who missed two or more scheduled assessments immediately prior to the PFS event will be censored at the last tumor assessment prior to the missed visits.

The following analyses will be performed for both PFS endpoints described above and secondary efficacy endpoints to be hierarchically tested (OS in bTMB PP1, PFS assessed by the investigator in bTMB PP2, OS in bTMB PP2). PFS and OS will be compared between

"Use of this document is governed by the terms of use on the first page of this document."

treatment arms with the use of the stratified log-rank test. The HR for PFS and OS will be estimated using a stratified Cox regression model. The 95% CIs for the HRs will be provided.

The hypothesis testing will be performed in the order described below:

- First, PFS will be tested at a one-sided significance level of 0.025 for bTMB PP1. If the estimate of the HR is <1 and the one-sided p-value corresponding to the stratified log-rank test is <0.025, the null hypothesis will be rejected, and it will be concluded that atezolizumab prolongs the duration of PFS relative to the control arm in bTMB PP1.
- If the hypothesis in (1) is rejected, then OS will be tested at a one-sided significance level of 0.025 in bTMB PP2. If the estimate of the HR is <1 and the one-sided p-value corresponding to the stratified log-rank test is <0.025, the null hypothesis will be rejected, and it will be concluded that atezolizumab prolongs the duration of OS relative to the control arm in bTMB PP1.
- If the hypotheses in (2) is rejected, then PFS will be tested at a one-sided significance level of 0.025 in bTMB PP2. If the estimate of the HR is <1 and the one-sided p-value corresponding to the stratified log-rank test is <0.025, the null hypothesis will be rejected, and it will be concluded that atezolizumab prolongs the duration of PFS relative to the control arm in bTMB PP2.
- If the hypothesis in (3) is rejected, then OS will be tested at a one-sided significance level of 0.025 in bTMB PP2. If the estimate of the HR is <1 and the one-sided p-value corresponding to the stratified log-rank test is less than the corresponding type I error rate, the null hypothesis will be rejected, and it will be concluded that atezolizumab prolongs the duration of OS relative to the control arm in bTMB PP2.

In stratified analyses, the stratification factors will be those used during randomization: specifically, for PP1 population, ECOG Performance Status (0 vs. 1), histology (non-squamous vs. squamous), and tissue availability (yes vs. no) as recorded in IxRS; for PP2 population, bTMB cutoff (high vs. moderate), ECOG Performance Status (0 vs. 1), histology (non-squamous vs. squamous), and tissue availability (yes vs. no) as recorded in IxRS. Both stratified and unstratified analysis will be performed. In case the number of patients is too small in some strata, patients in these strata may be pooled for analysis according to a pre-specified method to be presented in the Statistical Analysis Plan (SAP). Kaplan-Meier methodology will be used to estimate the median PFS for each treatment arm and to construct survival curves for the visual description of the difference between the treatment arms. The Brookmeyer-Crowley methodology will be used to construct the 95% CI for the median PFS for each treatment arm.

Determination of Sample Size

The sample size determination for bTMB PP1 and bTMB PP2 is based on the number of events required to demonstrate efficacy with regard to PFS. The estimate of the number of PFS events required is based on the following assumptions:

- One-sided significance level of 0.025 for the comparison in bTMB PP1
- One-sided significance level of 0.025 for the comparison in bTMB PP2
- Approximately 95% power to detect an HR of 0.6, corresponding to an improvement in median PFS from 6 months to 10 months in bTMB PP1.
- Approximately 98% power to detect an HR of 0.65, corresponding to an improvement in median PFS from 6 months to 9.2 months in bTMB PP2.
- No interim analysis of PFS
- Event times exponentially distributed
- Accrual duration is approximately 25 months
- Dropout rate is 5% per 12 months for each treatment arm

With these assumptions, approximately 440 patients in total will be randomized into the bTMB+ cohort, with approximately 280 patients in bTMB PP1. A total of approximately 198 PFS events are required for the PFS primary comparison for bTMB PP1. This number of events corresponds to a minimum detectable difference in HR of approximately 0.756. The primary analysis of PFS will occur when approximately 198 investigator-assessed PFS events have occurred in patients in bTMB PP1 or at least 6 months after the last patient in bTMB PP1 has

"Use of this document is governed by the terms of use on the first page of this document."

been enrolled, whichever occurs later. This number of events is expected to be reached approximately 32 months after the first patient is randomized in bTMB PP1. At the time of the PFS primary analysis in bTMB PP1, approximately 338 PFS events will have occurred in bTMB PP2, with a corresponding minimal detectable HR of approximately 0.808.

Interim Analysis

There will be no interim analysis planned for PFS in this cohort. No iDMC or IMC will be set up for this cohort.

If PFS in bTMB PP1 shows superiority of atezolizumab over chemotherapy, OS in bTMB PP1 will be tested at $\alpha = 0.025$ (one-sided). One interim analysis of OS in bTMB PP1 for comparison of atezolizumab versus chemotherapy will be conducted at the time of the final PFS analysis (with approximately 128 OS events). The final OS analysis will be conducted after approximately 190 total deaths have occurred in both the atezolizumab and chemotherapy arms in bTMB PP1. This is expected to occur approximately 46 months after the first patient is randomized.

If OS in bTMB PP1 and PFS in bTMB PP2 also show superiority of atezolizumab over chemotherapy, OS in bTMB PP2 will be tested at $\alpha = 0.025$ (one-sided). The OS analyses in bTMB PP2 will be performed at the same time as the OS test in bTMB PP1. The stopping boundaries for OS interim and final analyses in bTMB PP1 and bTMB PP2 population are computed using the Lan-DeMets approximation to the Pocock boundary. The actual stopping boundaries will depend on the actual number of OS events in bTMB PP1 and bTMB PP2 populations.

"Use of this document is governed by the terms of use on the first page of this document."

COHORT D: ENTRECTINIB IN PATIENTS WITH ROS1+ NSCLC

Objectives and Endpoints: Cohort D

EFFICACY OBJECTIVES	
**Primary Efficacy Objective**	**Corresponding Endpoint**
• To evaluate the efficacy of entrectinib in patients with ROS1+ advanced or metastatic NSCLC, as determined by the F1LCDx assay	• Investigator-assessed ORR based on confirmed objective response (indicated by two objective response assessments based on RECIST v1.1 separated by at least 4 weeks)
**Secondary Efficacy Objective**	**Corresponding Endpoints**
• To evaluate the efficacy of entrectinib in patients with ROS1+ advanced or metastatic NSCLC, as determined by the F1LCDx assay	• Investigator-assessed DOR, CBR, and PFS per RECIST v1.1<br>• IRF-assessed ORR, DOR, CBR, and PFS per RECIST v1.1<br>• Investigator- and IRF-assessed time to CNS progression according to RECIST v1.1<br>• Investigator-assessed intracranial tumor response rate by RECIST 1.1 in patients with measurable CNS disease at baseline<br>• OS
**SAFETY OBJECTIVE**	
**Safety Objective**	**Corresponding Endpoints**
• To evaluate the safety and tolerability of entrectinib	• Incidence, type, and severity of adverse events (based on the NCI CTCAE v4.0), including serious adverse events<br>• Changes in vital signs, physical findings, and clinical laboratory results during and following administration of protocol-specified IMP
**PHARMACOKINETIC OBJECTIVE**	
**Pharmacokinetic Objective**	**Corresponding Endpoints**
• To characterize the pharmacokinetics of entrectinib and its major metabolite(s)	• Population PK analysis for entrectinib
**PATIENT-REPORTED OUTCOME OBJECTIVES**	
**PRO Objective**	**Corresponding Endpoints**
• To evaluate the impact of entrectinib on PROs in patients with ROS1+ advanced or metastatic NSCLC, as determined by the F1LCDx assay	• Proportion of patients who improve compared with baseline in patient-reported lung cancer symptoms of cough, dyspnea, and chest pain, as measured by the SILC<br>• Proportion of patients presenting with measurable CNS disease at baseline who improve compared with baseline in patient-reported cognitive function, fatigue, HRQoL, headache, and vision disorder per the corresponding scales of the EORTC QLQ-C30 and BN20<br>• TTD in patient-reported lung cancer symptoms of cough, dyspnea, and chest pain, as measured by the SILC<br>• Mean change from baseline in HRQoL, patient functioning, and symptoms, as measured by the EORTC QLQ-C30 and SILC
• To evaluate and compare patients' health status to generate utility scores for use in economic models for reimbursement	• Health status as assessed by the EQ-5D-5L questionnaire

"Use of this document is governed by the terms of use on the first page of this document."

BIOMARKER OBJECTIVE	
**Biomarker Objective**	**Corresponding Endpoint**
• To assess prognostic effect and pharmacodynamics of exploratory biomarkers in blood, and their association with disease status, mechanisms of resistance, and/or response to entrectinib	• Relationship between circulating biomarkers related to entrectinib efficacy
EXPLORATORY OBJECTIVES	
**Exploratory Objectives**	**Corresponding Endpoints**
• To explore the anti-tumor effect of entrectinib in patients with CNS disease identified at baseline	• Investigator-assessed ORR per RECIST v1.1 in patients with CNS disease at baseline<br>• Investigator-assessed intracranial tumor response rate by RANO-BM in patients with measurable CNS disease at baseline<br>• Investigator-assessed time to CNS progression by RANO-BM
[redacted]	

CBR = clinical benefit rate; CNS = central nervous system; DOR = duration of response; EORTC = European Organisation for Research and Treatment of Cancer; EQ-5D-5L = EuroQol 5-Dimension, 5-Level questionnaire; F1LCDx = FoundationOne® Liquid Companion Diagnostic (assay); HRQoL = health-related quality of life; IMP = investigational medicinal product; IRF = independent review facility; NCI CTCAE v4.0 = National Cancer Institute Common Terminology Criteria for Adverse Events, Version 4.0; NSCLC = non–small cell lung cancer; ORR = objective response rate; OS = overall survival; PFS = progression-free survival; PK = pharmacokinetic; PRO = patient-reported outcome; RANO-BM = response assessment in neuro-oncology brain metastases; QLQ-C30 = Quality of Life Questionnaire Core 30; RECIST v1.1 = Response Evaluation Criteria in Solid Tumors, Version 1.1; SILC = Symptoms in Lung Cancer; [redacted] TTD = time to deterioration.

Cohort Design: Cohort D

Description of Cohort

The ROS1+ cohort will use a single-arm, open-label study design. Patients will receive entrectinib at 600 mg orally once a day. Patients will be treated until disease progression (according to Response Evaluation Criteria in Solid Tumors, Version 1.1 (RECIST v1.1), unacceptable toxicity, withdrawal of consent, study termination by sponsor, or death whichever occurs first. After disease progression per RECIST 1.1, patients should discontinue the study drug and additional treatment decisions will be at the discretion of the investigator according to local practice.

Patients who discontinue treatment prior to disease progression (e.g., because of unacceptable toxicity or withdrawal of consent) will continue to be followed until disease progression, unacceptable toxicity, withdrawal of consent, study termination by sponsor or death whichever occurs first, and for overall survival (OS) until withdrawal of consent, loss to follow-up, study termination by sponsor or death whichever occurs first regardless of whether they subsequently receive non–protocol-specified anti-cancer therapy.

Number of Patients

Approximately 50 patients will be enrolled in the ROS1+ cohort.

Target Population

To be enrolled in this cohort, patients must have met and continue to meet all eligibility criteria specified for the F1LCDx assay screening, in addition to the cohort-specific criteria below.

"Use of this document is governed by the terms of use on the first page of this document."

Additional Inclusion Criteria

Patients must meet the following additional criteria for entry in Cohort D:

- Signed cohort-specific informed consent for treatment
- ROS1 positivity, as determined by the F1LCDx assay
- No prior treatment for inoperable Stage IIIB not amenable to treatment with combined modality chemoradiation (advanced) or metastatic non-squamous or squamous NSCLC

  Patients who have received prior neo-adjuvant, adjuvant chemotherapy, radiotherapy, or chemoradiotherapy with curative intent for non-metastatic disease must have experienced a treatment-free interval of at least 6 months from enrollment since the last chemotherapy, radiotherapy, or chemoradiotherapy cycle.

- Prior brain metastases or leptomeningeal disease is allowed if asymptomatic and/or previously treated

  Patients who have received brain irradiation must have completed whole-brain radiotherapy and/or stereotactic radiosurgery at least 14 days prior to the start of entrectinib treatment.

- Adequate liver function, as defined using the following criteria:
  - AST and ALT $\leq 3.0 \times$ the upper limit of normal (ULN); $\leq 5 \times$ the ULN if liver metastases are present
  - Total serum bilirubin $\leq 2.5 \times$ the ULN

    Patients with a known history of Gilbert syndrome and/or isolated elevations of indirect bilirubin are eligible.

- Ability to swallow entrectinib intact, without chewing, crushing, or opening the capsules
- For women of childbearing potential, a negative serum pregnancy test ≤ 3 days prior to initial cohort treatment

Additional Exclusion Criteria

Patients who meet any of the following additional criteria will be excluded from entry in Cohort D:

- Incomplete recovery from any surgery prior to the start of entrectinib treatment that would interfere with the determination of safety or efficacy of entrectinib
- Any condition (within the past 3 months) that would interfere with the determination of safety or efficacy of entrectinib: myocardial infarction, unstable angina, coronary/peripheral artery bypass graft, symptomatic congestive heart failure, cerebrovascular accident or transient ischemic attack, stroke, symptomatic bradycardia, or uncontrolled arrhythmias requiring medication
- History of recent (within the past 3 months) symptomatic congestive heart failure or ejection fraction less than or equal to 50% observed during the screening for the study
- History of prolonged QTc interval (e.g., repeated demonstration of a QTc interval >450 milliseconds from ECGs performed at least 24 hours apart)
- History of additional risk factors for torsade de pointes (e.g., family history of long QT syndrome)
- Grade ≥ 2 peripheral neuropathy
- Known active infections that would interfere with the assessment of safety or efficacy of entrectinib (bacterial, fungal, or viral, including human immunodeficiency virus positive)
- Active gastrointestinal disease (e.g., Crohn's disease, ulcerative colitis, or short gut syndrome) or other malabsorption syndromes that would reasonably affect drug absorption
- Known interstitial lung disease, interstitial fibrosis, or history of tyrosine kinase inhibitor–induced pneumonitis

  Note: Radiation-induced lung disorders are not included in this exclusion criterion.

"Use of this document is governed by the terms of use on the first page of this document."

- Other severe acute or chronic medical or psychiatric condition or laboratory abnormality that may increase the risk associated with study participation or study drug administration or may interfere with the interpretation of study results and, in the judgment of the investigator, would make the patient inappropriate for entry into this study or could compromise protocol objectives in the opinion of the investigator and/or the Sponsor

Investigational Medicinal Product: Cohort D

Test Product (Investigational Drug)

Patients will receive entrectinib at a dose of 600 mg orally once a day. Entrectinib will be supplied as 200-mg capsules.

Statistical Methods: Cohort D

Primary Analysis

For the ROS1+ cohort, the primary efficacy endpoint is objective response rate (ORR), as assessed by the investigator per RECIST v1.1, defined as proportion of patients with an objective response. An objective response is defined as a complete response (CR) or partial response (PR) per RECIST v1.1. Confirmation of objective response is required (confirmed $\geq$4 weeks apart at two separate tumor assessments). Patients not meeting this criterion (including patients without a post-baseline tumor assessment) will be considered non-responders. An estimate of ORR and its 95% CI (using the Clopper-Pearson method) will be calculated for comparison with the historical ORR for entrectinib in a single-arm study of 50 patients. The primary efficacy analysis population is all treated patients with measurable disease at baseline. For the ROS1+ cohort, the final ORR analysis will occur when all treated patients have been followed for at least 8 months; this corresponds to the time window required for four tumor assessments.

Determination of Sample Size

Determination of sample size is based on demonstration of the manner of consistency of data between the two populations selected by two different diagnostics for the same mutations for ROS1 by observing at least 75% of the known/established ORR.

Assuming the known/expected ORR seen with entrectinib in STARTRK-2 (an open-label, multicenter, global Phase II study of entrectinib) is 75%, approximately 50 patients are planned to be enrolled in the ROS1+ cohort to provide a 75% chance that the lower limit of the two-sided 95% CI (using the Clopper-Pearson method) around the point estimate of the ORR in patients selected by the blood-based assay is higher than 57% (75% of the ORR previously seen with entrectinib for which patients were selected using tissue-based diagnostic testing).

"Use of this document is governed by the terms of use on the first page of this document."

COHORT E: ATEZOLIZUMAB, VEMURAFENIB, AND COBIMETINIB IN PATIENTS WITH BRAF V600+ NSCLC

Objectives and Endpoints: Cohort E

EFFICACY OBJECTIVES	
**Primary Efficacy Objective**	**Corresponding Endpoint**
• To evaluate the efficacy of atezo + cobi + vem in patients with BRAF V600+ advanced or metastatic NSCLC, as determined by the F1LCDx assay	• Investigator-assessed 12-month TIR per RECIST v1.1
**Secondary Efficacy Objective**	**Corresponding Endpoints**
• To evaluate the efficacy of atezo + cobi + vem in patients with BRAF V600+ advanced or metastatic NSCLC, as determined by the F1LCDx assay	• Investigator-assessed ORR, 9-month TIR, DOR, and PFS per RECIST v1.1<br>• IRF-assessed ORR, 12-month TIR, DOR, and PFS per RECIST v1.1<br>• OS
**Exploratory Efficacy Objective**	**Corresponding Endpoint**
• To evaluate the efficacy of atezo + cobi + vem in patients with BRAF V600+ advanced or metastatic NSCLC, as determined by the F1LCDx assay	• Investigator-assessed DCR
SAFETY OBJECTIVE	
**Safety Objective**	**Corresponding Endpoints**
• To evaluate the safety and tolerability of atezo + cobi + vem	• Incidence, type, and severity of adverse events (based on the NCI CTCAE v4.0), including serious adverse events<br>• Changes in vital signs, physical findings, and clinical laboratory results during and following administration of protocol-specified IMP
PHARMACOKINETIC OBJECTIVE	
**Pharmacokinetic Objective**	**Corresponding Endpoints**
• To characterize the pharmacokinetics of atezo + cobi + vem when administered together in patients with BRAF V600+ advanced or metastatic NSCLC, as determined by the F1LCDx assay	• Serum concentration of atezolizumab at specified timepoints

"Use of this document is governed by the terms of use on the first page of this document."

PATIENT-REPORTED OUTCOME OBJECTIVES	
**PRO Objective**	**Corresponding Endpoints**
• To evaluate the impact of atezo + cobi + vem on PROs in patients with BRAF V600+ advanced or metastatic NSCLC, as determined by the F1LCDx assay	• Proportion of patients who improve compared with baseline in patient-reported lung cancer symptoms of cough, dyspnea, and chest pain, as measured by the SILC<br>• TTD in patient-reported lung cancer symptoms of cough, dyspnea, and chest pain, as measured by the SILC<br>• Mean change from baseline in HRQoL, patient functioning, and symptoms, as measured by the EORTC QLQ-C30 and SILC
• To evaluate and compare patients' health status to generate utility scores for use in economic models for reimbursement<br>• As such, the analyses from the EQ-5D-5L will not be reported in the CSR.	• Health status as assessed by the EQ-5D-5L questionnaire
**IMMUNOGENICITY OBJECTIVE**	
**Immunogenicity Objective**	**Corresponding Endpoint**
• To evaluate the immune response to atezolizumab when administered in the combination of atezo + cobi + vem in patients with BRAF V600+ advanced or metastatic NSCLC, as determined by the F1LCDx assay	• Presence of ADAs against atezolizumab during the study relative to the presence of ADAs at baseline
**BIOMARKER OBJECTIVES**	
**Biomarker Objective**	**Corresponding Endpoint**
• To assess predictive, prognostic, and pharmacodynamic exploratory biomarkers in blood, and their association with disease status, mechanisms of resistance, and/or response to study treatment	• Relationship between circulating biomarkers and measures of atezo + cobi + vem efficacy

ADA = anti-drug antibody; atezo = atezolizumab; cobi = cobimetinib; CSR = Clinical Study Report; DCR = disease control rate; DOR = duration of response; EORTC = European Organisation for Research and Treatment of Cancer; EQ-5D-5L = EuroQol 5Dimension, 5-Level questionnaire; F1LCDx = FoundationOne® Liquid Companion Diagnostic (assay); HR-QoL = health-related quality of life; IMP = investigational medicinal product; IRF = independent review facility; NCI CTCAE v4.0 = National Cancer Institute Common Terminology Criteria for Adverse Events, Version 4.0; NSCLC = non–small cell lung cancer; ORR = objective response rate; OS = overall survival; PFS = progression-free survival; PRO = patient-reported outcome; QLQ-C30 = Quality of Life Questionnaire Core 30; RECIST v1.1 = Response Evaluation Criteria in Solid Tumors, Version 1.1; SILC = Symptoms in Lung Cancer; TIR = time in response; TTD = time to deterioration; vem = vemurafenib.

Cohort Design: Cohort E

Description of Cohort

The BRAF V600+ cohort will use a single-arm, open-label study design. Patients will receive cobimetinib and vemurafenib during a run-in period of 28 days, followed by treatment with atezolizumab, cobimetinib, and vemurafenib. Patients will be treated until disease progression (according to Response Evaluation Criteria in Solid Tumors, Version 1.1 [RECIST v1.1]), unacceptable toxicity, withdrawal of consent, study termination by Sponsor, or death, whichever occurs first. After disease progression per RECIST v1.1, patients should discontinue the study treatment, and additional treatment decisions will be at the discretion of the investigator according to local practice.

Patients who discontinue treatment prior to disease progression (e.g., because of unacceptable toxicity or withdrawal of consent) will continue to be followed until disease progression,

"Use of this document is governed by the terms of use on the first page of this document."

unacceptable toxicity, withdrawal of consent, study termination by Sponsor, or death, whichever occurs first, and for overall survival (OS) until withdrawal of consent, loss to follow-up, study termination by Sponsor, or death, whichever occurs first, regardless of whether patients subsequently receive non–protocol-specified anti-cancer therapy.

Approximately 80 patients will be enrolled in the BRAF V600+ cohort if enrollment continues beyond futility. There will be a planned futility assessment after 25 patients have a minimum of 13 months of follow-up.

Number of Patients

Approximately 80 patients will be enrolled in the BRAF V600+ cohort.

Target Population

To be enrolled in this cohort, patients must have met and continue to meet all eligibility criteria specified for the FoundationOne® Liquid Companion Diagnostic (F1LCDx) assay screening, in addition to the cohort-specific criteria below.

Additional Inclusion Criteria

Patients must meet the following additional inclusion criteria for entry into Cohort E:

- Signed cohort-specific Informed Consent Form for treatment
- BRAF V600+, as determined by the F1LCDx assay
- No prior treatment for inoperable Stage IIIB advanced (not amenable to treatment with combined modality chemoradiation) or metastatic non-squamous or squamous NSCLC

    Patients who have received prior neo-adjuvant therapy, adjuvant chemotherapy, radiotherapy, or chemoradiotherapy with curative intent for non-metastatic disease must have experienced a treatment-free interval of at least 6 months from enrollment since the last chemotherapy, radiotherapy, or chemoradiotherapy cycle.
- For women of childbearing potential: a negative serum pregnancy test ≤ 3 days prior to initial cohort treatment
- Eastern Cooperative Oncology Group Performance Status of 0 or 1
- Adequate hematologic and end-organ function, defined by the following laboratory test results, obtained within 14 days prior to initiation of study treatment, with the exception of amylase, lipase, and LDH where up to 28 days is acceptable
    - ANC ≥ 1.5 × 10^9/L without granulocyte colony-stimulating factor support
    - WBC count ≥ 2.5 × 10^9/L
    - Lymphocyte count ≥ 0.5 × 10^9/L
    - Platelet count ≥ 100 × 10^9/L without transfusion
    - Hemoglobin ≥ 90 g/L without transfusion
    - Serum albumin ≥ 25 g/L
    - Total bilirubin ≤ 1.5 × upper limit of normal (ULN)
    - AST and ALT ≤ 2.0 × ULN
    - Amylase and lipase ≤ 1.5 × ULN
    - ALP ≤ 2.5 × ULN or, for patients with documented liver or bone metastases, ALP ≤ 5 × ULN
    - Serum creatinine ≤ 1.5 × ULN or creatinine clearance (CrCl) ≥ 40 mL/min on the basis of measured CrCl from a 24-hour urine collection or Cockcroft-Gault glomerular filtration rate estimation:

$$\text{CrCl} = \frac{(140 - \text{age}) \times (\text{weight in kg})}{72 \times (\text{serum creatinine in mg/dL})} \quad (\times\ 0.85 \text{ if female})$$

- For patients not receiving therapeutic anticoagulation: INR or aPTT ≤ 1.5 × ULN within 28 days prior to initiation of study treatment
- For patients receiving therapeutic anticoagulation: stable anticoagulant regimen and stable INR during the 28 days immediately preceding initiation of study treatment

"Use of this document is governed by the terms of use on the first page of this document."

Additional Exclusion Criteria

Patients who meet any of the following additional criteria will be excluded from entry in Cohort E:

CANCER-RELATED EXCLUSION CRITERIA

Patients who meet any of the following cancer-related criteria will be excluded from entry in Cohort E:

- Presence of activating EGFR or ALK genetic alteration detected by the blood-based assay (F1LCDx)

  The F1LCDx assay reports both known and novel alterations. Only patients with known oncogenic alterations in EGFR (exon 19 deletions or L858R) or ALK will be excluded. Alterations in EGFR or ALK where oncogenic potential is unknown (i.e., variants of unknown significance) are not considered exclusionary.

- Major surgical procedure other than for diagnosis within 4 weeks prior to initiation of study treatment, or anticipation of need for a major surgical procedure during the course of the study
- Traumatic injury within 2 weeks prior to initiation of study treatment
- Palliative radiotherapy within 2 weeks prior to initiation of study treatment

OCULAR EXCLUSION CRITERIA

Patients who meet any of the following ocular criteria will be excluded from entry in Cohort E:

- History of or evidence of retinal pathology on ophthalmologic examination that is considered a risk factor for neurosensory retinal detachment, central serous chorioretinopathy, retinal vein occlusion (RVO), or neovascular macular degeneration

  Patients will be excluded from study participation if they currently are known to have any of the following risk factors for RVO:

  - History of serous retinopathy
  - History of retinal vein occlusion
  - Evidence of ongoing serous retinopathy or RVO at baseline

CARDIAC EXCLUSION CRITERIA

Patients who meet any of the following cardiac criteria will be excluded from entry in Cohort E:

- History of clinically significant cardiac dysfunction, including the following:
  - Poorly controlled hypertension, defined as sustained, uncontrolled, nonepisodic baseline hypertension consistently above 159/99 mmHg despite optimal medical management
  - Abnormal ECG that is clinically significant in the investigator's opinion, including complete left bundle branch block, second- or third-degree heart block, or evidence of prior myocardial infarction
  - Unstable angina, or new-onset angina within 3 months prior to initiation of study treatment
  - Symptomatic congestive heart failure, defined as New York Heart Association Class II or higher
  - Myocardial infarction within 3 months prior to initiation of study treatment
  - Unstable arrhythmia
  - History of congenital long QT syndrome
  - Mean (average of triplicate measurements) QT interval corrected through use of Fridericia's method (QTcF) $\geq$ 480 ms at screening or uncorrectable abnormalities in serum electrolytes (sodium, potassium, calcium, magnesium, and phosphorus)
  - Left ventricular ejection fraction (LVEF) below the institutional lower limit of normal of below 50%, whichever is lower

CENTRAL NERVOUS SYSTEM EXCLUSION CRITERIA

"Use of this document is governed by the terms of use on the first page of this document."

Patients who meet any of the following CNS criteria will be excluded from entry in Cohort E:

- Untreated or actively progressing CNS lesions (carcinomatous meningitis)

  Patients with a history of CNS lesions are eligible, provided that all of the following criteria are met:

  - Measurable disease, per RECIST v1.1, must be present outside the CNS.
  - All known CNS lesions have been treated with radiotherapy or surgery.
  - CNS lesions have not been treated with whole-brain radiotherapy, except in patients who underwent definitive resection of or stereotactic therapy for all radiologically detectable parenchymal brain lesions.
  - Absence of interim progression must be confirmed by radiographic study within 4 weeks prior to initiation of study treatment. If new CNS metastases are suspected during the screening period, a confirmatory radiographic study is required prior to initiation of study treatment.
  - Any radiotherapy or surgery must be completed ≥ 4 weeks prior to initiation of study treatment.
  - There is no ongoing requirement for corticosteroids, and any prior corticosteroid treatment must be discontinued ≥ 2 weeks prior to initiation of study treatment. Treatment with an anticonvulsant at a stable dose is allowed.
  - History of intracranial hemorrhage from CNS lesions is not allowed.

- History of metastases to brain stem, midbrain, pons, or medulla, or within 10 mm of the optic apparatus (optic nerves and chiasm)
- History of leptomeningeal metastatic disease

ADDITIONAL EXCLUSION CRITERIA

Patients who meet any of the following additional criteria will be excluded from entry in Cohort E:

- Current severe, uncontrolled systemic disease (including, but not limited to, clinically significant cardiovascular, pulmonary, or renal disease) other than cancer
- Anticipated use of any concomitant medication during or within 7 days prior to initiation of study treatment that is known to cause QT prolongation (which may lead to torsades de pointes)
- Uncontrolled diabetes or symptomatic hyperglycemia
- Any psychological, familial, sociological, or geographical condition that may hamper compliance with the protocol and follow-up after treatment discontinuation
- History of malabsorption or other clinically significant metabolic dysfunction that may interfere with absorption of oral study treatment
- Hereditary galactose intolerance, a congenital lactase deficiency or glucose-galactose malabsorption
- Pregnant or breastfeeding, or intending to become pregnant during the study

  Women of childbearing potential must have a negative serum pregnancy test result within 7 days prior to initiation of study treatment.

- Prior allogeneic stem cell transplant or solid organ transplantation
- History or idiopathic pulmonary fibrosis, organizing pneumonia (e.g., bronchiolitis obliterans), drug-induced pneumonitis, idiopathic pneumonitis, or evidence of active pneumonitis on screening computed tomography (CT) scan

  History of radiation pneumonitis in the radiation field (fibrosis) is permitted.

"Use of this document is governed by the terms of use on the first page of this document."

- Active or history of autoimmune disease or immune deficiency, including, but not limited to, myasthenia gravis, myositis, autoimmune hepatitis, systemic lupus erythematosus, rheumatoid arthritis, inflammatory bowel disease, anti–phospholipid antibody syndrome, Wegener granulomatosis, Sjögren syndrome, Guillain-Barré syndrome, or multiple sclerosis (see the protocol for a more comprehensive list of autoimmune diseases and immune deficiencies), with the following exceptions:

  Patients with a history of autoimmune-related hypothyroidism on a stable dose of thyroid-replacement hormone may be eligible for the study after discussion with the Medical Monitor.

  Patients with controlled Type 1 diabetes mellitus on a stable insulin regimen may be eligible for the study after discussion with the Medical Monitor.

  Patients with eczema, psoriasis, lichen simplex chronicus, or vitiligo with dermatologic manifestations only (e.g., patients with psoriatic arthritis are excluded) are eligible for the study provided all of following conditions are met:

  - Rash must cover < 10% of body surface area.
  - Disease is well controlled at baseline and requires only low-potency topical corticosteroids.
  - There is no occurrence of acute exacerbations of the underlying condition requiring psoralen plus ultraviolet A radiation, methotrexate, retinoids, biologic agents, oral calcineurin inhibitors, or high-potency or oral corticosteroids within the previous 12 months.
- Known clinically significant liver disease, including alcoholism, cirrhosis, fatty liver, and other inherited liver disease as well as active viral disease including:
  - Positive HIV test at screening
  - Active hepatitis B virus (HBV) infection (chronic or acute), defined as having a positive hepatitis B surface antigen (HBsAg) test at screening
    - Patients with a past or resolved HBV infection, defined as having a negative HBsAg test and a positive total hepatitis B core antibody (HBcAb) test at screening, are eligible for the study. Patients with a positive total HBcAb test are required to have a negative HBV DNA test. The HBV DNA test will only be performed in patients with a positive total HBcAb test.
  - Active hepatitis C virus (HCV) infection, defined as having a positive HCV antibody test and a positive HCV RNA test at screening
- Active tuberculosis
- Severe infection within 4 weeks prior to initiation of study treatment, including, but not limited to, hospitalization for complications of infection, bacteremia, or severe pneumonia
- Signs or symptoms of clinically relevant infection within 2 weeks prior to initiation of study treatment
- Any Grade ≥ 3 hemorrhage or bleeding event within 4 weeks prior to initiation of study treatment
- History of stroke, reversible ischemic neurological defect, or transient ischemic attack within 6 months prior to initiation of study treatment
- Any other disease, metabolic dysfunction, physical examination finding, or clinical laboratory finding that contraindicates the use of an investigational drug, may affect the interpretation of the results, or may render the patient at high risk from treatment complications
- Treatment with therapeutic oral or IV antibiotics within 2 weeks prior to initiation of study treatment

  Patients receiving prophylactic antibiotics (e.g., to prevent a urinary tract infection or chronic obstructive pulmonary disease exacerbation) are eligible for the study.
- Treatment with a live, attenuated vaccine within 4 weeks prior to initiation of study treatment, or anticipation of need for such a vaccine during the course of the study

"Use of this document is governed by the terms of use on the first page of this document."

- Treatment with systemic immunosuppressive medication (including, but not limited to, prednisone, cyclophosphamide, azathioprine, methotrexate, thalidomide, and anti–tumor necrosis factor [TNF]-α agents) within 2 weeks prior to initiation of study treatment, or anticipation of need for systemic immunosuppressive medication during the course of the study

  Patients who have received acute, low-dose systemic immunosuppressant medication ($\leq$10 mg/day oral prednisone or equivalent) $\geq$4 weeks prior to initiation of study treatment or a one-time pulse dose of systemic immunosuppressant medication (e.g., 48 hours of corticosteroids for a contrast allergy) are eligible for the study.

  The use of inhaled corticosteroids for chronic obstructive pulmonary disease or asthma, mineralocorticoids (e.g., fludrocortisone), or low-dose corticosteroids for patients with orthostatic hypotension or adrenocortical insufficiency is allowed.
- Known hypersensitivity to biopharmaceutical agents produced in Chinese hamster ovary cells
- Known hypersensitivity to any component of the atezolizumab, cobimetinib, or vemurafenib formulations
- History of severe allergic, anaphylactic, or other hypersensitivity reactions to chimeric or humanized antibodies or fusion proteins
- Treatment with any other investigational agent or participation in another clinical study with therapeutic intent
- Inability or unwillingness to swallow pills
- Requirement for concomitant therapy or food that is prohibited during the study, as described in the protocol

Investigational Medicinal Products: Cohort E

Atezolizumab 1680 mg will be administered by IV infusion Q4W on Day 1 starting on Day 29 (Cycle 1 Day 1, after completion of the 28-day run-in period).

Patients will receive cobimetinib at a dose of 60 mg (three 20-mg tablets) orally (PO) QD on Days 1–21 of each 28-day cycle during the run-in and triple-combination periods.

Patients will receive vemurafenib at a dose of 960 mg (four 240-mg tablets) PO BID on Days 1–21 of the initial run-in period. Patients will then receive vemurafenib at a dose of 720 mg (three 240-mg tablets) PO BID on Days 22–28 of the initial run-in period and on Days 1–28 of each 28-day cycle during the triple-combination period (atezolizumab, cobimetinib, and vemurafenib).

Statistical Methods: Cohort E

Primary Analysis

For the BRAF V600+ cohort, the primary efficacy endpoint is 12-month time in response (TIR), as assessed by the investigator per RECIST v1.1. TIR is defined as the duration of response (DOR) for patients with an objective response as determined by the investigator using RECIST v1.1 or as 1 day for patients without an objective response. An objective response is defined as a complete response (CR) or partial response (PR) per RECIST v1.1. Confirmation of objective response is required (confirmed $\geq$4 weeks apart at two separate tumor assessments). DOR is defined as the time interval from the date of the first occurrence of a confirmed CR or PR, whichever status is recorded first, until the first date that progressive disease or death is documented, whichever occurs first. For patients with a confirmed objective response, ones who have not progressed and who have not died at the time of analysis will be censored at the time of last tumor assessment date; if no tumor assessments were performed after date of the first occurrence of a complete or partial response, the TIR will be censored at the date of the first occurrence of a complete or partial response plus 1 day. Patients without a confirmed objective response will be analyzed as having an event at the first date of treatment plus 1 day. The 12-month TIR is defined as the TIR rate calculated through Kaplan-Meier method at 12 months. The 95% confidence intervals will be calculated based on the normal approximation with standard errors via the Greenwood method.

"Use of this document is governed by the terms of use on the first page of this document."

The primary efficacy analysis population is all treated patients with measurable disease at baseline. The primary analysis will occur approximately 16 months after the last patient is enrolled in BRAF V600+ cohort.

Determination of Sample Size

Approximately 80 patients will be enrolled in the BRAF V600+ cohort. Data from completed and ongoing studies in similar disease settings will be used as historical controls for comparison. Currently available data indicates that the historical 12-month TIR rate is approximately 28% for BRAF V600+ patients. A 12-month TIR of ≥43% (i.e., at least 15% increase compared with historical data representing the current standard of care for patients with BRAF V600+ NSCLC) would indicate a clinically meaningful treatment effect in this population. Eighty patients will provide approximately 80% power (exact binomial test) to detect a 15% increase in 12-month TIR from 28% to 43% at the one-sided 0.025 significance level. With approximately 80 patients, assuming the observed 12-month TIR rate is 43% in BRAF V600+ cohort, the two-sided 95% CI (Clopper-Pearson) will be approximately (32%, 55%).

Interim Analysis

One interim analysis for investigator-assessed ORR and 9-month TIR will be conducted when the first 25 patients have been followed up for at least 13 months. The Internal Monitoring Committee (IMC) will review the data and decide whether to recommend a decision to stop enrollment early in that cohort because of futility. An observed ORR between 64% and 72% with the 9-month TIR lower than 50% or an observed ORR less than 64% will meet the criteria for futility. Further details regarding the rules and guidelines of data review will be provided to the IMC in the IMC Charter.

"Use of this document is governed by the terms of use on the first page of this document."

COHORT F: ATEZOLIZUMAB, BEVACIZUMAB, CARBOPLATIN, AND PEMETREXED IN PATIENTS WITH EGFR EXON 20+ NSCLC

Objectives and Endpoints: Cohort F

EFFICACY OBJECTIVES	
**Primary Efficacy Objective**	**Corresponding Endpoint**
• To evaluate the efficacy of atezo + bev + carboplatin + pemetrexed in patients with EGFR exon 20+ in advanced or metastatic NSCLC, as determined by the F1LCDx assay	• Investigator-assessed ORR based on confirmed objective response (indicated by two objective response assessments based on RECIST v1.1 separated by at least 4 weeks)
**Secondary Efficacy Objective**	**Corresponding Endpoints**
• To evaluate the efficacy of atezo + bev + carboplatin + pemetrexed in patients with EGFR exon 20+ advanced or metastatic NSCLC, as determined by the F1LCDx assay	• Investigator-assessed DOR and PFS per RECIST v1.1<br>• IRF-assessed ORR, DOR, and PFS per RECIST v1.1<br>• OS
**Exploratory Efficacy Objective**	**Corresponding Endpoint**
• To evaluate the efficacy of atezo + bev + carboplatin + pemetrexed in patients with EGFR exon 20+ advanced or metastatic NSCLC, as determined by the F1LCDx assay	• Investigator-assessed DCR<br>• [redacted]<br>• Investigator- and IRF-assessed incidence of new brain metastases according to RECIST v1.1
SAFETY OBJECTIVE	
**Safety Objective**	**Corresponding Endpoints**
• To evaluate the efficacy of atezo + bev + carboplatin + pemetrexed in patients with EGFR exon 20+ advanced or metastatic NSCLC, as determined by the F1LCDx assay	• Incidence, type, and severity of adverse events (based on the NCI CTCAE v4.0), including serious adverse events<br>• Changes in vital signs, physical findings, and clinical laboratory results during and following administration of protocol-specified IMP
PHARMACOKINETIC OBJECTIVE	
**Pharmacokinetic Objective**	**Corresponding Endpoints**
• To characterize the pharmacokinetics of atezo when administered in combination with bev + carboplatin + pemetrexed in patients with EGFR exon 20+ advanced or metastatic NSCLC, as determined by the F1LCDx assay	• Serum concentration of atezo at specified timepoints

"Use of this document is governed by the terms of use on the first page of this document."

PATIENT-REPORTED OUTCOME OBJECTIVES	
PRO Objective	***Corresponding Endpoints***
• *To evaluate the impact of atezo + bev + carboplatin + pemetrexed on PROs in patients with EGFR exon 20+ advanced or metastatic NSCLC, as determined by the F1LCDx assay*	• *Proportion of patients who improve compared with baseline in patient-reported lung cancer symptoms of cough, dyspnea, and chest pain, as measured by the SILC*<br>• *TTD in patient-reported lung cancer symptoms of cough, dyspnea, and chest pain, as measured by the SILC*<br>• *Mean change from baseline in HR-QoL, patient functioning, and symptoms, as measured by the EORTC QLQ-C30 and SILC*
• *To evaluate and compare patients' health status to generate utility scores for use in economic models for reimbursement*<br>*As such, the analyses from the EQ-5D-5L will not be reported in the CSR.*	• *Health status as assessed by the EQ-5D-5L questionnaire*
IMMUNOGENICITY OBJECTIVE	
Immunogenicity Objective	***Corresponding Endpoint***
• *To evaluate the immune response to atezo when administered in the combination of atezo + bev + carboplatin + pemetrexed in patients with EGFR exon 20+ advanced or metastatic NSCLC, as determined by the F1LCDx assay*	• *Incidence of ADAs to atezo following treatment relative to the prevalence of ADAs to atezo at baseline*
BIOMARKER OBJECTIVES	
Biomarker Objective	***Corresponding Endpoint***
• *To assess predictive, prognostic, and pharmacodynamic exploratory biomarkers in blood, and their association with disease status, mechanisms of resistance, and/or response to study treatment*	• *Relationship between circulating biomarkers and measures atezo + bev + carboplatin + pemetrexed efficacy*

ADA = anti-drug antibody; atezo = atezolizumab; bev = bevacizumab; CSR = Clinical Study Report; DCR = disease control rate; DOR = duration of response; EGFR exon 20+ = epidermal growth factor receptor exon 20 mutations; EORTC = European Organisation for Research and Treatment of Cancer; EQ-5D-5L = EuroQol 5Dimension, 5-Level questionnaire; F1LCDx = FoundationOne® Liquid Companion Diagnostic (assay); HR-QoL = health-related quality of life; IMP = investigational medicinal product; IRF = independent review facility; NCI CTCAE v4.0 = National Cancer Institute Common Terminology Criteria for Adverse Events, Version 4.0; NSCLC = non–small cell lung cancer; ORR = objective response rate; OS = overall survival; PFS = progression-free survival; PRO = patient-reported outcome; QLQ-C30 = Quality of Life Questionnaire Core 30; RECIST v1.1 = Response Evaluation Criteria in Solid Tumors, Version 1.1; SILC = Symptoms in Lung Cancer; [redacted]; TTD = time to deterioration; vem = vemurafenib.

Cohort Design: Cohort F

Description of Cohort

The epidermal growth factor receptor exon 20 mutations (EGFR exon 20+) cohort will use a single-arm, open-label study design. Patients will receive the combination of atezolizumab, bevacizumab, carboplatin, and pemetrexed for four or six induction cycles. The decision on the number of indcution cycles, either four or six cycles, will be at the investigators discretion. Following the induction phase, patients will continue treatment with maintenance therapy consisting of a combination of atezolizumab, bevacizumab, and pemetrexed. Patients will be treated until disease progression (as assessed per investigator, according to Response Evaluation Criteria in Solid Tumors, Version 1.1 [RECIST v1.1]), unacceptable toxicity, withdrawal of consent, study termination by Sponsor, or death, whichever occurs first. After

"Use of this document is governed by the terms of use on the first page of this document."

disease progression as assessed per investigator per RECIST v1.1, patients should discontinue the study treatment, and additional treatment decisions will be at the discretion of the investigator according to local practice. Patients may continue treatment with atezolizumab beyond radiographic progression by RECIST v1.1, provided they are experiencing clinical benefit as assessed by the investigator (i.e., in the absence of unacceptable toxicity or symptomatic deterioration attributed to disease progression as determined by the investigator after an integrated assessment of radiographic data and clinical status).

Patients who discontinue study treatment prior to disease progression (e.g., because of unacceptable toxicity) will continue to be followed until disease progression, withdrawal of consent, study termination by Sponsor, or death, whichever occurs first, and for overall survival (OS) until withdrawal of consent, loss to follow-up, study termination by Sponsor, or death, whichever occurs first, regardless of whether patients subsequently receive non-protocol-specified anti-cancer therapy.

Approximately 80 patients will be enrolled in the EGFR exon 20+ cohort if enrollment continues beyond the futility analysis. There will be a planned futility assessment after approximately 25 patients have completed at least 3 months of follow-up.

Number of Patients

Approximately 80 patients will be enrolled in the EGFR exon 20+ cohort.

Target Population

Additional Inclusion Criteria

Patients must meet the following additional inclusion criteria for enrollment into Cohort F:

- *Signed cohort-specific Informed Consent Form for treatment*
- *EGFR exon 20 mutation-positive, as determined by the F1LCDx assay*
  - *The FoundationOne® Liquid Companion Diagnostic (F1LCDx) assay reports EGFR exon 20 mutations of known and unknown (or uncertain) significance. Only patients with known oncogenic mutations in EGFR exon 20 will be considered eligible.*
- *Eastern Cooperative Oncology Group (ECOG) performance status of 0 or 1*
- *Histologically or cytologically confirmed, non-squamous NSCLC*
  - *Patients with tumors of mixed histology (i.e., squamous and non-squamous) are eligible if the major histological component appears to be non-squamous.*
- *No prior treatment for advanced or metastatic non-squamous NSCLC*
- *Patients who have received prior neo-adjuvant, adjuvant chemotherapy, radiotherapy, or chemoradiotherapy with curative intent for non-metastatic disease must have experienced a treatment-free interval of at least 12 months from randomization since the last chemotherapy, radiotherapy, or chemoradiotherapy*
- *Patients with a history of treated asymptomatic CNS metastases are eligible, provided they meet all of the following criteria:*
  - *Only supratentorial and cerebellar metastases allowed (i.e., no metastases to midbrain, pons, medulla or spinal cord)*
  - *No ongoing requirement for corticosteroids as therapy for CNS disease*
  - *No stereotactic radiation within 7 days or whole-brain radiation within 14 days prior to randomization*
  - *No evidence of interim progression between the completion of CNS-directed therapy and the screening radiographic study*
- *Patients with new asymptomatic CNS metastases detected at the screening scan must receive radiation therapy and/or surgery for CNS metastases. Following treatment, these patients may then be eligible without the need for an additional brain scan prior to randomization, if all other criteria are met.*
- *For female patients of childbearing potential, agreement (by patient and/or partner) to use a highly effective form(s) of contraception*

"Use of this document is governed by the terms of use on the first page of this document."

- *Women who are not postmenopausal (at least 12 months of non-therapy induced amenorrhea) or surgically sterile must have a negative serum pregnancy test result within 14 days prior to initiation of study drug*
- *For male patients with female partners of childbearing potential, agreement (by patient and/or partner) to use a highly effective form(s) of contraception*
- *Adequate hematologic and end-organ function, defined by the following laboratory results obtained within 14 days prior to randomization:*
  - *ANC ≥ 1500 cells/μL without granulocyte colony-stimulating factor support*
  - *Lymphocyte count ≥ 500/μL*
  - *Platelet count ≥ 100,000/μL without transfusion*
  - *Hemoglobin ≥ 9.0 g/dL*

    Patients may be transfused to meet this criterion.
  - *INR or aPTT ≤ 1.5 × upper limit of normal (ULN)*

    This applies only to patients who are not receiving therapeutic anticoagulation; patients receiving therapeutic anticoagulation should be on a stable dose.
  - *AST, ALT, and alkaline phosphatase ≤ 2.5 × ULN, with the following exceptions:*

    Patients with documented liver metastases: AST and/or ALT ≤ 5 × ULN

    Patients with documented liver or bone metastases: alkaline phosphatase ≤ 5 × ULN.
  - *Serum bilirubin ≤ 1.25 × ULN*

    Patients with known Gilbert disease who have serum bilirubin level ≤ 3 × ULN may be enrolled.
  - *Serum creatinine ≤ 1.5 × ULN*

Additional Exclusion Criteria

Patients who meet any of the following criteria will be excluded from entry in Cohort F:

CANCER-SPECIFIC EXCLUSIONS

- *Active or untreated CNS metastases as determined by computed tomography (CT) or magnetic resonance imaging (MRI) evaluation during screening and prior radiographic assessments*
- *Spinal cord compression not definitively treated with surgery and/or radiation or previously diagnosed and treated spinal cord compression without evidence that disease has been clinically stable for > 2 weeks prior to randomization*
- *Leptomeningeal disease*
- *Uncontrolled tumor-related pain*

  Patients requiring pain medication must be on a stable regimen at study entry.

  Symptomatic lesions amenable to palliative radiotherapy (e.g., bone metastases or metastases causing nerve impingement) should be treated prior to randomization. Patients should be recovered from the effects of radiation. There is no required minimum recovery period.

  Asymptomatic metastatic lesions whose further growth would likely cause functional deficits or intractable pain (e.g., epidural metastasis that is not currently associated with spinal cord compression) should be considered for locoregional therapy, if appropriate, prior to randomization.
- *Uncontrolled pleural effusion, pericardial effusion, or ascites requiring recurrent drainage procedures (once monthly or more frequently)*

  Patients with indwelling catheters (e.g., PleurX®) are allowed.
- *Uncontrolled or symptomatic hypercalcemia (> 1.5 mmol/L ionized calcium or Ca > 12 mg/dL or corrected serum calcium > ULN)*
- *Mutations in EGFR exon 19 deletions or exon 21 L858R mutations or ALK fusions.*

"Use of this document is governed by the terms of use on the first page of this document."

GENERAL MEDICAL EXCLUSIONS

- *History of severe allergic, anaphylactic, or other hypersensitivity reactions to chimeric or humanized antibodies or fusion proteins*
- *Known hypersensitivity or allergy to biopharmaceuticals produced in Chinese hamster ovary cells or any component of the atezolizumab formulation*
- *History of autoimmune disease, including but not limited to myasthenia gravis, myositis, autoimmune hepatitis, systemic lupus erythematosus, rheumatoid arthritis, inflammatory bowel disease, vascular thrombosis associated with antiphospholipid syndrome, Wegener's granulomatosis, Sjögren's syndrome, Guillain-Barré syndrome, multiple sclerosis, vasculitis, or glomerulonephritis*

  Patients with a history of autoimmune-related hypothyroidism on a stable dose of thyroid replacement hormone are eligible for this study.

  Patients with controlled Type 1 diabetes mellitus on a stable dose of insulin regimen are eligible for this study.

  Patients with eczema, psoriasis, lichen simplex chronicus, or vitiligo with dermatologic manifestations only (e.g., patients with psoriatic arthritis would be excluded) are permitted provided that they meet the following conditions:

    Rash must cover less than 10% of body surface area.

    Disease is well controlled at baseline and only requiring low-potency topical steroids.

    No acute exacerbations of underlying condition within the previous 12 months (not requiring psoralen plus ultraviolet A radiation, methotrexate, retinoids, biologic agents, oral calcineurin inhibitors, high-potency or oral steroids)

- *History of idiopathic pulmonary fibrosis, organizing pneumonia (e.g., bronchiolitis obliterans), drug-induced pneumonitis, idiopathic pneumonitis, or evidence of active pneumonitis on screening chest CT scan*

  History of radiation pneumonitis in the radiation field (fibrosis) is permitted.

- *Positive test for HIV*

  All patients will be tested for HIV prior to inclusion into the study; patients who test positive for HIV will be excluded from the study.

- *Patients with active hepatitis B (chronic or acute; defined as having a positive hepatitis B surface antigen [HBsAg] test at screening) or hepatitis C*

  Patients with past hepatitis B virus (HBV) infection or resolved HBV infection (defined as the presence of hepatitis B core antibody [HBcAb] and absence of HBsAg) are eligible only if they are negative for HBV DNA.

  Patients positive for hepatitis C virus (HCV) antibody are eligible only if polymerase chain reaction (PCR) is negative for HCV RNA.

- *Active tuberculosis*
- *Severe infections within 4 weeks prior to randomization, including, but not limited to, hospitalization for complications of infection, bacteremia, or severe pneumonia*
- *Received therapeutic oral or IV antibiotics within 2 weeks prior to randomization*

  Patients receiving prophylactic antibiotics (e.g., for prevention of a urinary tract infection or to prevent chronic obstructive pulmonary disease exacerbation) are eligible.

- *Significant cardiovascular disease, such as New York Heart Association cardiac disease (Class II or greater), myocardial infarction, or cerebrovascular accident within 3 months prior to randomization, unstable arrhythmias, or unstable angina*

  Patients with known coronary artery disease, congestive heart failure not meeting the above criteria, or left ventricular ejection fraction <50% must be on a stable medical regimen that is optimized in the opinion of the treating physician, in consultation with a cardiologist if appropriate.

"Use of this document is governed by the terms of use on the first page of this document."

- *Major surgical procedure other than for diagnosis within 28 days prior to randomization or anticipation of need for a major surgical procedure during the course of the study*
- *Prior allogeneic bone marrow transplantation or solid organ transplant*
- *Administration of a live, attenuated vaccine within 4 weeks before randomization or anticipation that such a live attenuated vaccine will be required during the study*
- *Any other diseases, metabolic dysfunction, physical examination finding, or clinical laboratory finding giving reasonable suspicion of a disease or condition that contraindicates the use of an investigational drug or that may affect the interpretation of the results or renders the patient at high risk from treatment complications*
- *Patients with illnesses or conditions that interfere with their capacity to understand, follow and/or comply with study procedures*

EXCLUSION CRITERIA RELATED TO MEDICATIONS

- *Treatment with any other investigational agent with therapeutic intent within 28 days prior to randomization*
- *Prior treatment with CD137 agonists or immune checkpoint blockade therapies, anti−PD-1, and anti−PD-L1 therapeutic antibodies*

  Patients who have had prior anti−CTLA-4 treatment may be enrolled, provided the following requirements are met:
  - *Last dose of anti−CTLA-4 at least 6 weeks prior to randomization*
  - *No history of severe immune-mediated adverse effects from anti−CTLA-4 (NCI CTCAE Grade 3 or 4)*
- *Treatment with systemic immunostimulatory agents (including but not limited to interferons, interleukin-2) within 4 weeks or five half-lives of the drug, whichever is longer, prior to randomization*

  Prior treatment with cancer vaccines is allowed.
- *Prior anti-VEGF or anti-VEGFR treatment*
- *Treatment with systemic immunosuppressive medication (including, but not limited to, corticosteroids, cyclophosphamide, azathioprine, methotrexate, thalidomide, and anti−tumor necrosis factor−α [TNF−α] agents) within 2 weeks prior to initiation of study treatment, or anticipation of need for systemic immunosuppressive medication during study treatment, with the following exceptions:*
  - *Patients who received acute, low-dose systemic immunosuppressant medication or a one-time pulse dose of systemic immunosuppressant medication (e.g., 48 hours of corticosteroids for a contrast allergy) may be eligible for the study after Medical Monitor confirmation has been obtained.*
  - *Patients who received mineralocorticoids (e.g., fludrocortisone), inhaled or low-dose corticosteroids for COPD or asthma, or low-dose corticosteroids for orthostatic hypotension or adrenal insufficiency are eligible for the study.*

EXCLUSIONS RELATED TO BEVACIZUMAB

- *Inadequately controlled hypertension (defined as systolic blood pressure >150 mmHg and/or diastolic blood pressure >100 mmHg)*

  Anti-hypertensive therapy to achieve these parameters is allowable.
- *Prior history of hypertensive crisis or hypertensive encephalopathy*
- *Significant vascular disease (e.g., aortic aneurysm requiring surgical repair or recent peripheral arterial thrombosis) within 6 months prior to randomization*
- *History of hemoptysis (≥one-half teaspoon of bright red blood per episode) within 1 month prior to randomization*
- *Evidence of bleeding diathesis or coagulopathy (in the absence of therapeutic anticoagulation)*

"Use of this document is governed by the terms of use on the first page of this document."

- *Current or recent (within 10 days of randomization) use of aspirin (>325 mg/day) or treatment with dipyramidole, ticlopidine, clopidogrel, and cilostazol*
- *Current use of full-dose oral or parenteral anticoagulants or thrombolytic agents for therapeutic purposes that has not been stable for >2 weeks prior to randomization*

  The use of full-dose oral or parenteral anticoagulants is permitted as long as the INR or aPTT is within therapeutic limits (according to the medical standard of the enrolling institution) and the patient has been on a stable dose of anticoagulants for at least 2 weeks prior to randomization.

  Prophylactic anticoagulation for the patency of venous access devices is allowed, provided the activity of the agent results in an INR <1.5 × ULN and aPTT is within normal limits within 14 days prior to randomization.

  Prophylactic use of low-molecular-weight heparin (i.e., enoxaparin 40 mg/day) is permitted.
- *Core biopsy or other minor surgical procedure, excluding placement of a vascular access device, within 7 days prior to the first dose of bevacizumab*
- *History of abdominal or tracheoesophageal fistula or gastrointestinal perforation within 6 months prior to randomization*
- *Clinical signs of gastrointestinal obstruction or requirement for routine parenteral hydration, parenteral nutrition, or tube feeding*
- *Evidence of abdominal free air not explained by paracentesis or recent surgical procedure*
- *Serious, non-healing wound, active ulcer, or untreated bone fracture*
- *Proteinuria, as demonstrated by urine dipstick or >1.0 g of protein in a 24-hour urine collection*

  All patients with ≥ 2 + protein on dipstick urinalysis at baseline must undergo a 24-hour urine collection and must demonstrate ≤1 g of protein in 24 hours.
- *Known sensitivity to any component of bevacizumab*
- *Clear tumor infiltration into the thoracic great vessels is seen on imaging*
- *Clear cavitation of pulmonary lesions is seen on imaging*

EXCLUSIONS RELATED TO CHEMOTHERAPY

- *Known history of severe allergic reactions to platinum-containing compounds or mannitol.*

Investigational Medicinal Products: Cohort F

Atezolizumab 1200 mg will be administered by IV infusion on Day 1 of each 21-day cycle during the induction and maintenance periods.

Bevacizumab (AVASTIN) will be administered 15 mg/kg by IV infusion on Day 1 of each 21-day cycle during the induction and maintenance periods. The initial dose of bevacizumab will be based on the patient's weight at screening and will remain the same throughout the study unless the patient's weight changes by >10%.

Carboplatin will be administered by IV infusion at a target dose of area under the concentration–time curve (AUC) of 5 mg/mL/min (Calvert formula dosing) on Day 1 of each 21-day cycle for four or six cycles during the induction period.

Pemetrexed 500 mg/m² will be administered by IV infusion on Day 1 of each 21-day cycle during the induction and maintenance periods.

Statistical Methods: Cohort F

Primary Analysis

For the EGFR Exon 20+ cohort, the primary efficacy endpoint is objective response rate (ORR) as assessed by the investigator per RECIST v1.1, defined as proportion of patients with an objective response. An objective response is defined as a complete response (CR) or partial response (PR) per RECIST v1.1. Confirmation of objective response is required

"Use of this document is governed by the terms of use on the first page of this document."

(confirmed ≥4 weeks apart in two separate tumor assessments). Patients not meeting this criterion (including patients without a post-baseline tumor assessment) will be considered non-responders. An estimate of ORR and its 95% CI (the Clopper-Pearson method) will be calculated for comparison with the historical ORR for patients with exon 20 activating mutations. The primary efficacy analysis population is all treated-patients with measurable disease at baseline.

For the EGFR Exon 20+ cohort, the primary analysis will occur when all treated-patients have been followed for at least 6 months; this corresponds to the time window required for four tumor assessments.

Determination of Sample Size

Approximately 80 patients will be enrolled in the EGFR exon 20+ cohort.

Currently available data indicates that the historical ORR is approximately 27.5% (95% CI [15.0–40.0]) (Tomaras et al. 2020) in NSCLC patients with EGFR exon 20+ who received platinum-based or other cytotoxic chemotherapy regimen, with or without prior TKI.

In addition, results from IMpower150 showed that in patients with sensitizing EGFR mutations (L858R or exon 19 deletions) who had received prior EGFR TKI therapy treated with ABCP (n=34), the response rate was 70.6% (95% CI 52.5%, 84.9%).

With a sample size of 80 patients, assuming the observed ORR rate is 38.8% (31/80 responders), the two-sided 95% CI (Clopper-Pearson) will be approximately (28%, 50%) and the lower bound would exclude the historical control ORR of 27.5%.

Interim Analysis

Predictive probabilities may be used to guide early stopping for futility by comparing the observed proportion of patients who achieve an objective response with that in historical controls, ORR in NSCLC patients with EGFR exon 20 mutations who received platinum-based or other cytotoxic chemotherapy regimen, with or without prior TKI, 27.5% (95% CI [15.0–40.0]). The design is based on Lee and Liu (2008), with the modification that the uncertainty in the historical-control data is fully taken into account by utilizing a distribution on the control response rate.

Interim analysis decision rules will be based on the predictive probability that this trial will have a positive outcome if carried out to completion and will use the historical-control data available at the time of analysis.

It is anticipated that at least one interim analysis will be conducted for futility, with the earliest interim analysis taking place when approximately 25 patients treated have been followed for at least 3 months and evaluated for objective response. If, at any interim analysis, a low predictive probability suggests that the proportion of patients achieving an objective response is lower than desired, the Internal Monitoring Committee (IMC) will review the data and decide whether to recommend an early decision to stop enrollment. Additional review of safety and/or efficacy data by the IMC may be requested by and carried out at the discretion of the Medical Monitor. Further details regarding the rules and guidelines of data review will be provided in an IMC charter.

"Use of this document is governed by the terms of use on the first page of this document."

LIST OF ABBREVIATIONS AND DEFINITIONS OF TERMS

Abbreviation	Definition
ADA	anti-drug antibody
ALK	anaplastic lymphoma kinase
ASCO	American Society of Clinical Oncology
AUC	area under the concentration-time curve
BCC	basal cell carcinoma
BFAST	Blood First Assay Screening Trial
BID	twice a day
bTMB	blood tumor mutational burden
bTMB PP1	the primary population of patients with a bTMB level equal to or greater than the higher validated cutoff
bTMB PP2	the secondary population of all patients who are bTMB-positive; the ITT population
CAP	College of American Pathologists
CPK	creatine phosphokinase
CR	complete response
CT	computed tomography
CTA	clinical trial assay
cfDNA	cell-free DNA
CrCl	creatine clearance
ctDNA	circulating tumor DNA
cuSCC	cutaneous squamous cell carcinoma
DLT	dose-limiting toxicity
DOR	duration of response
EC	Ethics Committee
ECG	electrocardiogram
ECOG	Eastern Cooperative Oncology Group
eCRF	electronic Case Report Form
EDC	electronic data capture
EGFR	epidermal growth factor receptor
EGFR exon 20+	*EGFR exon 20 mutations*
EKR	extracellular signal-related kinase
EORTC	European Organisation for Research and Treatment of Cancer
EQ-5D-5L	EuroQol 5-Dimension, 5-Level questionnaire
F1LCDx	FoundationOne® Liquid Companion Diagnostic (assay)
FDA	Food and Drug Administration
GLOBOCAN	Global Burden of Cancer Study
HBcAb	hepatitis B core antibody
HBsAg	hepatitis B surface antigen
HBV	hepatitis B virus
HCV	hepatitis C virus
HIPAA	Health Insurance Portability and Accountability Act
HR	hazard ratio
HRQoL	health-related quality of life

"Use of this document is governed by the terms of use on the first page of this document."

Abbreviation	Definition
ICH	International Council for Harmonisation
iDMC	independent Data Monitoring Committee
IMC	Internal Monitoring Committee
IMP	investigational medicinal product
IND	Investigational New Drug (Application)
IRB	Institutional Review Board
IRF	independent review facility
ITT	intent-to-treat
IxRS	interactive voice/web response system
KA	keratoacanthoma
LCMC	Lung Cancer Mutation Consortium
LPLV	last patient, last visit
LVEF	left ventricular ejection fraction
MAD	maximum administered dose
MAF	mutant allele frequency
MAPK	mitogen activated protein kinase
MDD	minimum detectable difference
MHC	major histocompatibility complex
MRI	magnetic resonance imaging
MTD	maximum tolerated dose
NCCN	National Comprehensive Cancer Network
NCI *CTCAE*	National Cancer Institute *Common Terminology Criteria for Adverse Events*
NGS	next-generation sequencing
NSAIDs	non-steroidal anti-inflammatory drugs
NSCLC	non–small cell lung cancer
OCT	optical coherence tomography
ORR	objective response rate
OS	overall survival
PCR	polymerase chain reaction
PD-L1	programmed death ligand-1
PFS	progression-free survival
PK	pharmacokinetic
PO	orally
PR	partial response
PRO	patient-reported outcome
Q2W	every 2 weeks
Q3W	every 3 weeks
Q4W	every 4 weeks
QD	once a day
QLQ-BN20	Quality of Life Questionnaire Brain Cancer Module
QLQ-C30	Quality of Life Questionnaire Core 30
QLQ-LC13	Quality of Life Questionnaire Lung Cancer Module
QTcF	QTc interval corrected using Fridericia's method
RBR	Research Biosample Repository

"Use of this document is governed by the terms of use on the first page of this document."

Abbreviation	Definition
RECIST	Response Evaluation Criteria In Solid Tumors
RET	rearranged during transfection
RP2D	recommended Phase II dose
RVO	retinal vein occlusion
SAP	Statistical Analysis Plan
SCC	squamous cell carcinoma
SEER	Surveillance, Epidemiology and End Results
SILC	Symptoms in Lung Cancer
TIR	time in response
TMB	tumor mutational burden
TNF	tumor necrosis factor
TTD	time to deterioration
TTR	time to response
ULN	upper limit of normal
V600E	valine at codon 600
WES	whole-exome sequencing
WGS	whole genome sequencing

"Use of this document is governed by the terms of use on the first page of this document."

1. BACKGROUND

1.1 BACKGROUND ON NON–SMALL CELL LUNG CANCER

Lung cancer remains the leading cause of cancer death worldwide; it is the most common cancer in both men and women and is estimated to account for approximately 13% of all new cancers in 2016 (Miller et al. 2016). Non–small cell lung cancer (NSCLC), as a whole, is not a rare tumor (224,390 expected new cases in the United States in 2016) (Miller et al. 2016), and several treatments via a number of therapeutic mechanisms are approved for use. However, the disease is heterogeneous and comprises a number of rare or uncommon subgroups of patients, leaving considerable room for improvement in treating patients with a personalized approach.

Patients diagnosed with early-stage NSCLC have reasonable outcomes (5-year survival rate of 55%). However, the remaining 76% of the patient population who are diagnosed with regional or metastatic disease have poorer outcomes (5-year survival rates of 31% and 5% for regional and metastatic disease, respectively [Howlader et al. 2016]). Based on the Global Burden of Cancer Study (GLOBOCAN) 2012 data from the WHO Europe region, the overall age-standardized rate for lung cancer was 449 cases (313 cases in European Union) per 100,000 persons, with 36.5 deaths per 100,000 persons (International Agency for Cancer Research world population standard) (Ferlay et al. 2015). The GLOBOCAN database does not provide information on histology or cancer stage; thus, the proportion of the advanced or metastatic NSCLC could not be specifically determined for this European population. NSCLC accounts for 80%–85% of all lung cancers in the Surveillance, Epidemiology and End Results (SEER) database; assuming a distribution similar to the data provided from SEER can be expected for the European Union, this would produce an expectation of 266 cases of NSCLC per 100,000 persons in the European Union per year.

Patients with advanced or metastatic NSCLC usually receive platinum-based doublet chemotherapy, unless they harbor tumors with activating EGFR, ALK, or ROS1 mutations and are treated with appropriately targeted therapy (based upon diagnostic selection) as first-line treatment (National Comprehensive Cancer Network [NCCN] 2016). These treatment options have produced response rates of up to 35% with chemotherapy (Schiller et al. 2002; Sandler et al. 2006; Scagliotti et al. 2008), 74% with crizotinib (ALK inhibitor; Xalkori®), and 65% with EGFR-targeted therapy (Rosell et al. 2012; Solomon et al. 2014). Additionally, the associated median progression-free survival (PFS) is approximately 6 months for chemotherapy and up to 14 months with targeted therapy, while the median overall survival (OS) is approximately 10 months with chemotherapy and up to 28 months with targeted therapy (Xalkori® and Tarceva® U.S. Prescribing Information). However, most patients will relapse as a result of inherent or acquired resistance and will require subsequent lines of therapy.

"Use of this document is governed by the terms of use on the first page of this document."

The effectiveness of existing therapies declines in patients with advanced or metastatic disease who receive additional therapies beyond the first-line. While there are approved targeted therapies for previously treated patients with ALK- or EGFR-positive NSCLC, available approved treatment options for ALK- or EGFR-negative patients with previously treated advanced or metastatic NSCLC were limited to docetaxel, pemetrexed, and erlotinib prior to 2015. These agents have produced objective response rates (ORRs) of 5.5%–8.9% in pivotal trials in this patient population, with limited benefit in median PFS (range, 2–3 months) and median OS (range, 5.7–8.3 months) (Taxotere®, Alimta®, and Tarceva® U.S. Prescribing Information).

More recently, immunotherapy options such as atezolizumab (Tecentriq®), nivolumab (Opdivo®) and pembrolizumab (Keytruda®) have been approved in the United States, the European Union, and other countries for previously untreated (in the case of pembrolizumab) or previously treated advanced or metastatic NSCLC. In certain settings, patients are treated irrespective of biomarker information, while in others, patients are selected by a protein expression biomarker, largely regardless of driver mutation status (includes some, but not all patients with a specific mutation). The combination of ramucirumab (Cyramza®; VEGF-R2 antagonist) and docetaxel has also been recently approved in an unselected, previously treated NSCLC population. These recently approved agents have resulted in ORRs of ≤23% in unselected, previously treated NSCLC populations. While no single biomarker has, at this time, clearly discerned those patients who benefit from immunotherapies, it has been theorized that patients with high-mutation-load tumors are more likely to benefit than—as well as perhaps represent a distinct population from—those patients who harbor driver mutations (Alexandrov et al. 2013; Rizvi et al. 2015).

1.2 DRIVER MUTATIONS AND TARGETED THERAPY IN NSCLC

Oncogenic driver mutations are distinguished from “passenger” mutations by their critical role in cancer development and maintenance. Driver mutations are typically mutually exclusive and have been identified for the majority of lung adenocarcinomas (including EGFR, BRAF, RAS, ALK, RET, HER2, and ROS1) and squamous cell carcinomas (including PIK3CA and rare cases of other mutations). Therapies that are specifically targeted to “actionable” mutant oncogenic drivers have shown significant efficacy in NSCLC, as outlined below.

Recently, both the Lung Cancer Mutation Consortium (LCMC) and the Biomarkers France project of the French Thoracic Intergroup demonstrated the feasibility of routine genetic analysis in clinical practice (Kris et al. 2014; Barlesi et al. 2016). The LCMC also demonstrated that use of this genetic information could be applied to guide treatment and facilitate studies of targeted agents in NSCLC. The LCMC reported that oncogenic driver(s) were identified in 64% of patients and showed a median survival of 3.5 years for the 260 patients with an oncogenic driver who received targeted therapy, compared with 2.4 years for the 318 patients with any oncogenic driver(s) who did not receive

"Use of this document is governed by the terms of use on the first page of this document."

targeted therapy (propensity score-adjusted hazard ratio [HR] = 0.69, p = 0.006). Together, with randomized trials for targeted therapies such as erlotinib for EGFR-mutant NSCLC and crizotinib for ALK-translocated NSCLC, these data strongly support the incorporation of genetic tumor testing into routine practice to guide clinical care, as recommended in the NCCN guidelines.

Genetic tumor testing generally requires tissue, and because of the location of lesions and co-morbidities often associated with NSCLC, tissue sampling can be difficult and tissue biopsies from lung cancer patients are often very small, rendering testing of multiple important biomarkers a challenge. Current tissue testing rates for common mutations in patients with available tissue have been reported to be between 70% and 90% in the United States and generally below 50% outside the United States (Foundation Medicine Database, Roche Market Research). Recent reports indicate that up to 33% of patients do not have enough accessible tissue for pathologic diagnosis and/or any genetic testing (Kris et al. 2014). Even when tissue is obtained, single assays for common biomarkers require so much tissue material that often little is left for testing for low-prevalence biomarkers such as ALK (~5%), RET (~1.8%), ROS1 (~1%), and others. Thus, optimal medication for a patient's cancer may not be made available to them because of the lack of test results for these low-prevalence markers. A blood assay analyzing driver mutations and other alterations in circulating tumor DNA (ctDNA), which combines testing for both high- and low-prevalence biomarkers, will enable more NSCLC patients to be tested for important biomarkers and ensure access to optimal treatment of their cancer.

Background on specific driver mutations of interest for this protocol, as well their respective targeted therapies in NSCLC, is provided in the following appendices:

- Cohort A (patients who are ALK+): Appendix 7, Section 11.1
- Cohort B (patients who are RET+): Appendix 8, Section 12.1
- Cohort D (patients who are ROS1+): Appendix 10, Section 14.1
- Cohort E (patients who are BRAF V600+): Appendix 11, Section 15.1
- *Cohort F (patients who are EGFR exon 20+): Appendix 12, Section* 16.1

1.3 BLOOD TUMOR MUTATIONAL BURDEN AND IMMUNOTHERAPY IN NSCLC

Emerging data in the field of cancer immunotherapy suggest that tumor mutational burden (TMB) may be a better diagnostic than current biomarkers for the selection of patients for treatment with PD-1/PD-L1 inhibitors. This hypothesis is supported by the notion that tumors with higher rates of somatic mutations, such as in NSCLC, may have increased immunogenicity and are therefore more susceptible to checkpoint inhibition (Chen et al. 2012). In a recent study conducted by Rizvi et al. (2015), investigators reported that nonsynonymous mutational burden, as determined by whole-exome

"Use of this document is governed by the terms of use on the first page of this document."

sequencing (WES) of NSCLC patients treated with pembrolizumab, was associated with improved outcomes such as ORR, durable clinical benefit, and PFS.

Additional considerations in the diagnostic selection of patients include whether an appropriate quality and quantity of tissue samples can be collected to test for the increasing array of oncogenic driver mutations. Advances in diagnostic technologies are enabling newer less-invasive genomic profiling in the blood via detection of ctDNA (Tsui and Berger 2016). Currently, commercial tests of ctDNA are available to guide treatment for targeted therapies. The next advance in this field will be to develop a ctDNA-based diagnostic to guide treatment for immunotherapies through detection of mutational burden in the blood.

Background on tumor immunotherapy, as well their respective targeted therapies in NSCLC, is provided in the following appendices:

- Cohort C (patients who are positive by the blood tumor mutational burden [bTMB] assay, or bTMB+): Appendix 9, Section 13.1

1.4 BACKGROUND ON STUDY TREATMENT

This trial will employ a number of different cohorts, each with a specific study treatment. This design—also referred to as an umbrella trial—allows inclusion of additional cohorts, which may be single-agent or combination therapy. Refer to the cohort-specific appendices of this protocol for background on the respective study treatments, as well as the respective Investigator's Brochures and local prescribing information for details on nonclinical and clinical studies.

1.5 STUDY RATIONALE AND BENEFIT–RISK ASSESSMENT

In the current age of targeted therapies, advances in biomarker identification and the increasing access to biomarker testing have allowed for a more personalized healthcare approach and treatment with appropriately targeted therapies. However, these technological advances have also helped to uncover some limitations to be overcome in order for truly personalized healthcare to become a reality. The first of these limitations is that for many patients with tumors like NSCLC, obtaining enough tissue to test for an ever-increasing number of biomarkers is difficult. This in effect limits diagnostic testing, the associated targeted drug development, and thus, treatment options for patients. Further, retesting patients multiple times throughout the course of therapy to guide treatment is rarely possible, as the benefit–risk ratio of obtaining more tissue is often not favorable.

A second limitation is that some tumors considered common, such as NSCLC, are increasingly being divided into subgroups based on infrequently or rarely occurring biomarkers. A more efficient means to test patients and develop drugs targeted at identified mutations or biomarkers is needed in order to provide these patients with access to effective and innovative treatment options. Use of innovative diagnostics,

"Use of this document is governed by the terms of use on the first page of this document."

such as multiplex panels (in tissue or blood), and studies of small cohorts of biomarker-selected patients (such as basket or umbrella trials) are a plausible means to identify patients and generate a reasonable amount of data to support the selection and treatment of rare tumor populations. Data can then be evaluated relative to active or historical controls (e.g., efficacy and safety data for approved therapeutic entities, or best supportive care in the setting where no treatment options are approved). If, relative to standard of care, the data generated by targeting the identified mutation(s) (or TMB) are deemed to represent a clinically meaningful benefit/risk profile acceptable for approval, this would provide a more efficient and innovative means to develop targeted drugs for these infrequent or rare tumor populations and enable more patients access to beneficial therapeutic options. This study (BO29554; the Blood First Assay Screening Trial [BFAST]) is designed to address these issues through the use of a blood-based next-generation sequencing (NGS) ctDNA diagnostic assay and multiple biomarker-selected cohorts within an umbrella trial.

The BFAST umbrella trial platform will serve to provide clinical validation for diagnostic patient selection for appropriate targeted therapy or immunotherapy via a blood-based NGS ctDNA assay identifying somatic mutations and measuring TMB in the blood. All patients, regardless of the availability of tissue, will be considered eligible for blood-based screening and potential inclusion in the trial. Patients with a driver mutation or whose tumors are biomarker positive for bTMB, as identified by the ctDNA assay, will undergo further clinical screening and be enrolled in the trial. The clinical efficacy and safety data generated will serve as the clinical validation for patient selection via the blood-based NGS ctDNA assay and the basis for benefit–risk analysis to support registration of new drug and diagnostic indications. Within this umbrella design, there will be a number of different treatment cohorts, each with specific efficacy and safety objectives. The umbrella platform design allows inclusion of additional cohorts, which may be single-agent or combination therapy, to address patients with other mutations or biomarkers identified by the assay; any additional cohorts would be added during the course of the trial via protocol amendment.

Rationale for Use of Investigational Diagnostic

NCCN guidelines recommend molecular testing of lung cancer patients for a range of predictive and prognostics biomarkers, including EGFR and ALK, to select patients for targeted therapy, as well as more extensive biomarker testing to identify patients who might benefit from targeted therapies in the context of a clinical trial (NCCN 2016). Further, it is recommended that tumor tissue be screened for PD-L1 expression at the time of progressive metastatic disease if being considered for pembrolizumab treatment. However, molecular testing via tissue biopsy cannot always be performed on NSCLC patients, potentially limiting access of effective treatment to the subset of patients who are able to undergo current testing procedures that utilize tissue biopsies.

Obtaining an adequate tissue biopsy specimen for NSCLC presents a number of challenges. In particular, the method for diagnosis of lung cancer depends on the

"Use of this document is governed by the terms of use on the first page of this document."

location, size, and type of suspected lung cancer, and the presence or absence of metastases (Rivera et al. 2013). Common procedures for centrally located tumors include bronchoscopy and sputum cytology, which frequently yield insufficient tumor tissue for comprehensive mutation testing. In addition, 75% of patients are diagnosed with late-stage disease and often present with multiple comorbidities. A recent report on the prevalence of comorbidities in cancer patients in the United States included patients with lung cancer aged 66 years or older, and revealed that 33.6% have chronic obstructive pulmonary disease as a comorbidity followed by diabetes and sequelae (14.7%), congestive heart failure (12.4%), cerebrovascular disease (7.2%), and peripheral vascular disease (6.8%) (Edwards et al. 2014). Biopsies in these patients can lead to complications such as pneumothorax, hemoptysis, other bleeding complications, and cardiopulmonary decompensation (Tam et al. 2013). As a result of these factors, a physician treating a patient who presents with a significant burden of disease as well as significant comorbidities may rightly consider whether it is in the best interest of the patient to undergo a risky and potentially unsuccessful procedure. According to a recent analysis of the California Cancer Registry, up to 30% of patients diagnosed with lung cancer do not receive a diagnostic procedure that is amenable to molecular analysis, precluding these patients from gaining access to highly active and potentially less toxic targeted therapies.

As described above, there exists a major unmet clinical need for testing procedures that do not require tumor tissue. It follows that the availability of an U.S. Food and Drug Administration (FDA)–approved multiplex diagnostic test based on easily accessible substrate such as cell-free DNA (cfDNA) from blood would allow the testing of more patients to determine their molecular status and would therefore contribute significantly to providing the most appropriate molecularly based treatment option for each patient.

In BFAST, a blood-based NGS ctDNA assay developed by Foundation Medicine to detect mutations in ctDNA isolated from blood will be used for patient screening and selection. The FoundationOne® Liquid Companion Diagnostic (F1LCDx) assay includes 324 cancer-related genes including coding exons of 310 genes, as well as select intronic regions or non-coding regions of 14 genes. The assay detects substitutions and insertions/deletions (indels) in a total of 310 genes and also detects select genomic rearrangements, select copy number alterations, and genomic signatures including microsatellite instability and bTMB. The assay has been launched in a Clinical Laboratory Improvement Act (CLIA)–and College of American Pathologists (CAP)–accredited laboratory.

Elevated TMB in tissue, the total number of coding somatic base substitutions detected in a tumor sample, has been observed in a variety of cancer types including NSCLC (Alexandrov et al. 2013; Lawrence et al. 2013; Vogelstein et al. 2013). Multiple clinical studies have reported that high TMB is associated with response to several immunotherapeutic agents, including anti-CTLA-4 in melanoma (Snyder et al. 2014),

"Use of this document is governed by the terms of use on the first page of this document."

anti-PDL1 therapy in bladder cancer (Rosenberg et al. 2016), and anti-PD-1 therapy in lung and colorectal cancers (Le et al. 2015; Rizvi et al. 2015).

Similarly, the bTMB assay measures the total number of somatic mutations, or mutational burden in ctDNA. Higher levels of TMB in tissue have been shown to be correlated with higher levels of neoantigens capable of eliciting a robust immune response (Brown et al. 2014; Schumacher and Screiber 2015), a requirement for immune-mediated recognition of tumors facilitated by checkpoint inhibitor immunotherapies (Snyder et al. 2014; Rizvi et al. 2015; Rosenberg et al. 2016). The assay identifies mutations (coding base substitutions) in the entire coding sequence of 394 genes, plus introns from 28 genes often rearranged or altered in cancer. Analytical validation has been completed and the assay has been launched in a CLIA- and CAP-accredited laboratory.

2. OBJECTIVES AND ENDPOINTS

This study will evaluate the efficacy and safety of multiple therapies that are selected using predictive biomarkers identified via a blood-based NGS assay in patients with previously untreated advanced NSCLC. Specific objectives and corresponding endpoints for the study are outlined for each cohort in the following cohort-specific appendices:

- Cohort A (patients who are ALK+): Appendix 7, Section 11.2
- Cohort B (patients who are RET+): Appendix 8, Section 12.2
- Cohort C (patients who are bTMB+): Appendix 9, Section 13.2
- Cohort D (patients who are ROS1+): Appendix 10, Section 14.2
- Cohort E (patients who are BRAF V600+): Appendix 11, Section 15.2
- *Cohort F (patients who are EGFR exon 20+): Appendix 12, Section* 16.2

This study will also characterize the natural history of patients with genomic alteration profiles of interest who do not enroll in any of the above treatment cohorts. Analyses will be exploratory in nature; there is no formal hypothesis testing in the natural history cohort.

3. STUDY DESIGN

3.1 DESCRIPTION OF THE STUDY

3.1.1 Overview of Study Design

Study BO29554 (BFAST) is a Phase II/III, global, multicenter, open-label, multi-cohort study designed to evaluate the safety and efficacy of targeted therapies or immunotherapy as single agents or in combination in patients with unresectable, advanced or metastatic NSCLC determined to harbor oncogenic somatic mutations (e.g., ALK, RET) or positive by TMB assay (above a prespecified cutoff) as identified by a blood-based NGS ctDNA assay.

"Use of this document is governed by the terms of use on the first page of this document."

Male and female patients aged ≥ 18 years who meet the trial's general inclusion/exclusion criteria (see Section 4.1) will be eligible for blood-based screening. Blood specimens from patients meeting eligibility criteria for the blood-based screening part of the study will be prospectively tested for somatic alterations in a panel of oncogenes, including the targetable oncogenes and TMB.

The overarching structure of the BFAST study is an umbrella screening and interventional study (see Figure 1), which will screen patient blood samples for the presence of potentially oncogenic somatic mutations and biomarker positivity by the bTMB assay in NSCLC via a blood-based NGS ctDNA assay, and will treat patients with a drug or drug regimen tailored to their results. Initially, three cohorts testing the efficacy and safety of therapy directed at specific mutations or biomarkers (ALK, RET, and biomarker positive for bTMB [bTMB-positive]) were implemented (see Figure 1). Additional cohorts will be added to address various identified somatic mutations or other biomarkers via future protocol amendments. Each cohort may have separate endpoints, screening, and treatment requirements.

Patients will be assigned to the appropriate cohort based on their identified oncogenic somatic mutation(s). If more than one mutation is identified, the priority for cohort assignment will be as follows:

1. Line of therapy (cohorts that allow patient enrollment for earlier lines of therapy given preference) where applicable
2. Lesser prevalence of mutation or biomarker in the population as follows:

   ROS1 rearrangement (~1%): Cohort D (entrectinib) ***(Note: Cohort D has been closed to further enrollment)***

   BRAF V600 mutation (~1%–2%): Cohort E (vemurafenib, cobimetinib, atezolizumab)

   EGFR exon 20 mutation (~1%–2%): Cohort F (atezolizumab, bevacizumab, carboplatin, pemetrexed)

   RET rearrangement (~1.8%): Cohort B (alectinib) **(Note: Cohort B has been closed to further enrollment.)**

   ALK rearrangement (~5%): Cohort A (alectinib) **(Note: Cohort A has been closed to further enrollment.)**

   bTMB-positive (~23%–36%): Cohort C (atezolizumab vs. chemotherapy) ***(Note: Cohort C has been closed to further enrollment)***
3. Natural history follow-up ***(Note: as of 26 OCT 2020 , the enrollment into the natural history follow-up cohort has been paused, as the initial goal of enrolling approximately 1,000 patients has been met)***

   Patients who are screened using the ctDNA assay but who do not enroll in a treatment cohort are eligible to begin natural history follow-up (if consent is given) to document their cancer therapies (or therapy types), treatment

"Use of this document is governed by the terms of use on the first page of this document."

response, and survival status. *See Section* 4.5.2 *and* Appendix 1 *for other data that were collected as part of initial blood screening process for these patients.* Patients with an alteration relevant to the BFAST trial or other key alteration profiles of interest (e.g., KRAS+, HER2+, MET+, PIK3CA+, *other EGFR alterations,* other ALK alterations, other RET alterations, other BRAF alterations, or other ROS1 alterations) who pursue therapy or other management options outside of this study for any reason (e.g., failure to meet treatment-specific eligibility criteria, choosing not to enroll, physician decision to pursue alternative therapy, patient experiencing disease progression before they can enroll, etc.) will be the population of interest for this component of the study. Information will be obtained approximately every 3 months during routine doctor or healthcare appointments and/or telephone calls. For patients in the natural history follow-up, a blood sample will be collected at the time of disease progression for blood-based assay testing (if consent is given). These samples may be stored for future exploratory analyses.

Figure 1 Study Schema: Mutation Screening

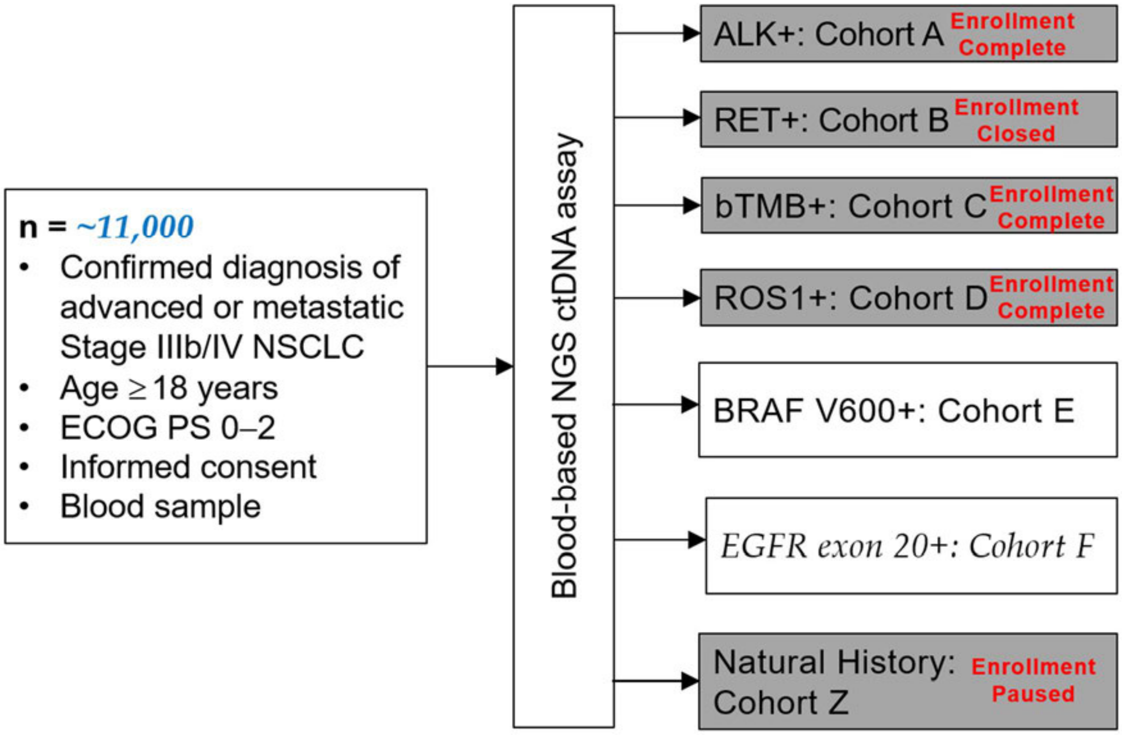

ctDNA = circulating tumor DNA; ECOG PS = Eastern Cooperative Oncology Group Performance Status; NGS = next-generation sequencing; NSCLC = non–small cell lung cancer.

Mandatory blood samples at baseline, during therapy (i.e., at each tumor assessment), and at progression of disease, as well as optional tissue (or results from tissue-based testing, when available), will be collected in order to evaluate exploratory prognostic and/or predictive biomarkers, including but not limited to biomarkers related to driver

"Use of this document is governed by the terms of use on the first page of this document."

oncogene signaling and NSCLC pathogenesis. For all patients, blood samples will be used as the only means of identifying driver mutations or TMB for inclusion in treatment cohorts, irrespective of tissue availability or tissue testing results. For patients who do not screen positive for an available cohort (or who are enrolled on the study but discontinue study treatment for reasons other than disease progression), or at the time of disease progression on study, treating physicians will have the option to receive access to a report of the F1LCDx assay results (see Section 4.5.6).

To improve patient access to clinical trials and subject to informed consent, central F1LCDx results for patients deemed not eligible for the enrollment in any active BFAST cohort, may be used to determine if patients are potentially eligible for other Sponsor's studies.

The trial is designed to demonstrate clinical efficacy and safety in the target population, though dose-finding may be necessary in certain cohorts. Treatment will be assigned on the basis of relevant oncogenotype, will have cohort-specific inclusion/exclusion criteria, and unless otherwise specified, will continue until disease progression per Response Evaluation Criteria In Solid Tumors, Version 1.1 (RECIST v1.1) (Eisenhauer et al. 2009), loss of clinical benefit (if applicable; see cohort-specific appendices), unacceptable toxicity, patient or physician decision to discontinue, or death, whichever occurs first. If the patient discontinues treatment prior to disease progression (because of adverse event, withdrawal of consent to treatment, or other reason), tumor assessment data collection will continue as specified in the cohort-specific appendices until disease progression per RECIST v1.1, death, withdrawal of consent, or study closure by the Sponsor, whichever occurs first. Follow-up data capture, including survival and subsequent anti-cancer therapies, will continue for each patient until death, loss to follow-up, study discontinuation, or study closure, whichever occurs first. Information regarding the nature and the duration of subsequent therapies will be collected.

3.1.2 Number of Patients

Approximately *11000* patients are anticipated to be screened for driver mutations or bTMB via the blood-based NGS ctDNA assay, and approximately *700* patients are anticipated to be enrolled at approximately 140 sites for treatment within the current *treatment* cohorts of this study (see Table 1).

The umbrella structure of this study allows for addition of cohorts through protocol amendments to address any of the identified oncogenic targets as assessed by the F1LCDx assay. Additional cohorts may be added as targeted therapies or combinations for additional alterations or other biomarkers become available for clinical testing and/or as cohorts close enrollment for the initially identified target mutations. Any additional cohorts will increase the total sample size and could increase the number of patients screened via the blood-based NGS ctDNA assay.

"Use of this document is governed by the terms of use on the first page of this document."

Table 1 Current Enrollment Projections by Cohort

Cohort	Regimens	Enrollment (n)
Cohort A (ALK+)	Alectinib	87 (actual) [a]
Cohort B (RET+)	Alectinib	8 (actual) [b]
Cohort C (bTMB+)	Atezolizumab or platinum-based chemotherapy	*471 (actual;* randomized 1:1)
Cohort D (ROS1+)	Entrectinib	*55 (actual)*
Cohort E (BRAF V600+)	Vemurafenib, cobimetinib, atezolizumab	*Approximately* 80
Cohort F (EGFR exon 20+)	*Atezolizumab, Bevacizumab, Carboplatin, Pemetrexed*	*Approximately 80*
Cohort Z	*Natural History*	*Approximately 1000*

[a] Original projected enrollment was 78 patients.

[b] Original projected enrollment was ~52–62 patients, but enrollment has been closed.

3.2 END OF STUDY AND LENGTH OF STUDY

The end of this study is defined as the date when the last patient, last visit (LPLV) or assessment occurs for the collection of the last data point for the last treatment cohort. Each cohort may end prior to the end of study when all predefined treatment, follow-up, and data collection are completed.

Based on the longitudinal nature of this trial, the projected duration of this study from first patient enrolled to "end of study," including survival follow-up visits conducted by telephone or in the clinic, is expected to be approximately 4–6 years with the current cohorts. However, the umbrella nature of this protocol may provide for an extension of the overall duration of this study to meet the objectives of additional cohorts and/or additional therapies added through protocol amendments, thus changing the assumptions and projections for LPLV.

3.3 RATIONALE FOR STUDY DESIGN

For additional rationale beyond what is covered below, please refer to the individual cohort-specific appendices.

The BFAST study has been designed to select treatment strategies for patients with advanced or metastatic NSCLC using predictive blood-based biomarkers. Thus, the treatment cohorts reflect mutations and other biomarkers that are known or expected to be predictive of response and/or outcome in this disease setting. As previously stated, current and potential future cohorts involve rare mutations, and enrolling a large, randomized study is not feasible because of the significant amount of time required to identify a sufficient number of eligible patients.

"Use of this document is governed by the terms of use on the first page of this document."

The patient population selected for this trial is based on the unmet medical (or diagnostic) need for most or all patients with advanced NSCLC. Targeted or biomarker-driven therapy for patients with available tissue and access to drugs that are approved in the first-line has proven to be safer and more effective compared with conventional chemotherapy (Rosell et al. 2012; Solomon et al. 2014). While tissue testing is common for markers such as EGFR and ALK, a multitude of other potential targets have been identified for which testing is not routinely performed. Further, a significant portion of patients never have their tumor tissue molecularly profiled and therefore may be dispositioned to receive cytotoxic chemotherapy or potentially supportive care only, depending on performance status and other clinical factors. This translates to only a subset of patients with actionable mutations or predictive biomarkers (or biosignatures) receiving molecularly optimized treatment. Our goal is to identify biomarker-specific treatments for the majority of patients in the first-line setting, including those patients who are not able to submit adequate tumor tissue for molecular profiling. This is driven by the use of a multiplex blood-based assay with the ability to identify most currently known driver mutations for which drugs have been developed to target. Mutations detected in this trial will be prospectively defined as clinically relevant to patients with advanced NSCLC, and individual cohorts and selection criteria will be designed accordingly. As science and information evolves, the protocol may be amended in the future to include the detection of novel mutations, or different variants of an existing mutation.

Control groups for this trial will vary based on each individual cohort's design. In many cases, a single-arm, open-label design compared with a carefully selected historical control is optimal to be able to offer all patients in the study a personalized, biologically directed treatment in the first-line setting. Many of the mutations of interest are considered rare, making the ability to identify enough patients to conduct an adequate randomized trial very challenging, even within an umbrella trial design. These are also situations where a targeted approach has a plausible chance of achieving substantial clinical improvement over the accepted standard of care. For patients with RET rearrangements, which are rare (~1.8%) in NSCLC, the standard of care is unclear. Therefore, this cohort of the trial will be using a single-arm design, aiming to show that alectinib provides a clinically meaningful improvement in outcome compared with conventional chemotherapy.

In situations where targeted therapy represents the standard of care, it is hypothesized that patients will have similar outcomes when selected using a blood-based assay compared with selection using a tissue sample. This is the case for the ALK cohort (Cohort A) of this trial, who will be compared with a similar group of patients receiving first-line alectinib in a large, Phase III, randomized trial (compared with crizotinib). The statistical methodology for this cohort is to determine whether the treatment effect is consistent between the patient population selected by the blood assay and the patient population selected by tissue and receiving identical treatment.

"Use of this document is governed by the terms of use on the first page of this document."

For treatment cohorts selected for mutations or biomarkers with higher prevalence and/or a more well-defined standard of care, a randomized approach will likely be deemed necessary for comparison to treatment with standard-of-care therapy.

Because of the umbrella nature of this study, the rationale for the study design is individualized on a cohort-specific basis. Please refer to the cohort-specific appendices for additional details.

3.3.1 Rationale for Biomarker Assessments

Advanced solid tumors, including NSCLC, constitute a heterogeneous disease within each patient, and mutations and gene abnormalities have been shown to vary between and within primary and metastatic lesions. A biopsy provides a window into disease that is limited by both site and time. Therefore, blood will be evaluated to look at the level and nature of mutations associated with the relevant study medication, which will represent all the lesions shedding ctDNA. It is also of interest to assess the presence or absence of resistance mutations present in blood and to evaluate how these evolve over the course of treatment of the patient. Pharmacodynamic biomarkers will be assessed to demonstrate evidence of biologic activity of the study medications in patients. Genomic testing is increasingly informing researchers' understanding of disease pathobiology. Targeted NGS provides a multiplex characterization of the genome and, along with clinical data collected in this study, may increase the opportunity for developing new therapeutic approaches. Data will be analyzed in the context of this study but will also be explored in aggregate with data from other studies. The availability of a larger dataset will assist in identification of important pathways, guiding the development of new targeted agents.

3.3.2 Rationale for Patient-Reported Outcome Assessments

A patient-reported outcome (PRO) is "any report on the status of a patient's health condition that comes directly from the patient, without any interpretation of the patient's response by a clinician or anyone else" (FDA 2009). PRO measures are able to contextualize a patient's experience during the course of a clinical trial, in the context of symptom burden, treatment burden, and patient's ability to function.

The efficacy of lung cancer treatment may be evaluated by its contribution to increased survival and symptoms palliation (Hyde and Hyde 1974; Hopwood and Stephens 1995; Sarna et al. 2004). This is especially true for trials that use ORR or PFS as a primary or secondary endpoint, in which it is important to translate changes in tumor progression or a delay in disease progression into an endpoint that is meaningful to patients. Chest pain, dyspnea, and cough have been regarded as the most frequent and clinically relevant disease-related symptoms experienced by NSCLC patients. The BR.21 study (erlotinib versus chemotherapy in second- or third-line NSCLC) demonstrated that a longer time to deterioration (TTD) in the pain, dyspnea, and cough scales of the European Organisation for Research and Treatment of Cancer Quality of Life

"Use of this document is governed by the terms of use on the first page of this document."

Questionnaire Core 30 (EORTC QLQ-C30) and Quality of Life Questionnaire Lung Cancer Module (QLQ-LC13) was consistent with superior PFS, OS, and quality-of-life benefits in the erlotinib arm compared with the placebo arm (Aaronson et al. 1993; Bergman et al. 1994; Bezjak et al. 2006).

In the BFAST study, PRO data will be collected from patients using the Symptoms in Lung Cancer (SILC) and EORTC QLQ-C30 questionnaires in order to assess patient-reported symptoms, global health status, and patient-reported functioning. In addition, in order to evaluate health status utility scores of patients treated with each treatment, generic or non-disease-specific PRO data will be collected using the EQ-5D-5L questionnaire.

Considering that patients might also benefit from treatment effect on metastases located in the brain, select scales from the EORTC QLQ-BN20 brain cancer supplementary module (Taphoorn et al. 2010) will be used for the ROS1 cohort (Cohort D) to translate the patient-relevant impact of CNS activity observed with entrectinib (see Section 14.1). Specifically, Item 34 measuring headache and Items 36–38 measuring visual disorders will be assessed for all patients in the ROS1 cohort in addition to all items from the SILC, EORTC QLQ-C30, and EQ-5D-5L questionnaires.

3.3.3 Rationale for Natural History Follow-Up

Patients with a genomic alteration relevant to the BFAST trial (e.g., bTMB+, ALK+, RET+, ROS1+, BRAF V600+) or with another key alteration profile of interest (including, but not limited to, KRAS+, HER2+, MET+, PIK3CA+, other ALK alterations, other RET alterations, other BRAF alterations, or other ROS1 alterations) who pursue therapy or other management options outside of the study's treatment cohorts for any reason will be the population of interest for this component of the study. Many of these patients are expected to have genomic alterations of interest identified for which the natural history is not adequately characterized. Collection of follow-up cancer therapy and survival information on such patients may help provide information on these sometimes rare subsets of patients with NSCLC.

4. MATERIALS AND METHODS

4.1 PATIENTS

This study will enroll patients with advanced NSCLC.

4.1.1 General Inclusion Criteria

Patients must meet the following criteria to be eligible for blood-based NGS ctDNA assay screening:

- Signed informed consent form for the blood-based screening part of the study and willingness to participate in an assigned cohort based on their identified oncogenic somatic mutation(s)

"Use of this document is governed by the terms of use on the first page of this document."

- Histologically or cytologically confirmed diagnosis of unresectable Stage IIIb not amenable to treatment with combined modality chemoradiation (advanced) or Stage IV (metastatic) NSCLC

  Mixed tumors should be categorized according to the predominant cell type.

- No prior systemic treatment for unresectable Stage IIIb or IV NSCLC
- Age $\geq$ 18 years
- Eastern Cooperative Oncology Group (ECOG) Performance Status 0–2
- Measurable disease (as defined by RECIST v1.1)
- Adequate recovery from most recent systemic or local treatment for cancer
- Adequate organ function, as demonstrated by the following clinical laboratory parameters:
  - Hemoglobin $\geq$ 9 g/dL
  - ANC $\geq 1.0 \times 10^9$/L
  - Platelet count $\geq 75 \times 10^9$/L
  - Serum AST and ALT $\leq 2.5 \times$ the upper limit of normal (ULN) or $\leq 5 \times$ ULN for patients with concurrent liver metastases
  - Bilirubin $\leq 2 \times$ ULN or $\leq 5 \times$ ULN for patients with concurrent liver metastases
  - Serum creatinine $\leq 2 \times$ ULN or creatinine clearance $>$ 45 mL/min
- Life expectancy $\geq$ 12 weeks
- For female patients of childbearing potential and male patients, willingness to use acceptable methods of contraception (see Appendix 2)

Additionally, in order to be enrolled on a treatment cohort of the study, patients must meet the specific criteria for the respective treatment cohorts as provided in the following appendices:

- Cohort A (ALK+): Appendix 7, Section 11.4.1.1
- Cohort B (RET+): Enrollment closed
- Cohort C (bTMB+): Appendix 9, Section 13.4.1.1
- Cohort D (ROS1+): Appendix 10, Section 14.4.1.1
- Cohort E (BRAF V600+*):* Appendix 11, Section 15.4.1.1
- *Cohort F (EGFR exon 20+): Appendix 12, Section* 16.4.1.1

To be enrolled in a treatment cohort, patients must have met and continue to meet all eligibility criteria specified above for the blood-based NGS ctDNA assay screening.

"Use of this document is governed by the terms of use on the first page of this document."

4.1.2 General Exclusion Criteria

Patients who meet any of the following criteria will be excluded from participating in the blood-based screening part of the study:

- Inability to swallow oral medication
- Women who are pregnant or lactating
- Symptomatic, untreated CNS metastases

  Patients with treated and/or asymptomatic brain metastases may still be eligible for treatment on the study depending on individual cohort requirements; see the cohort-specific appendices for details regarding eligibility.

- History of malignancy other than NSCLC within 5 years prior to screening, with the exception of malignancies with a negligible risk of metastasis or death (e.g., 5-year OS rate $\geq$90%), such as adequately treated carcinoma in situ of the cervix, non-melanoma skin carcinoma, localized prostate cancer, ductal carcinoma in situ of breast, or Stage I uterine cancer
- Significant cardiovascular disease, such as New York Heart Association cardiac disease (Class II or greater), myocardial infarction, or cerebrovascular accident within 3 months prior to randomization, unstable arrhythmias, or unstable angina

  Patients with known coronary artery disease, congestive heart failure not meeting the above criteria, or known left ventricular ejection fraction $<$50% must be on a stable medical regimen that is optimized in the opinion of the treating physician, in consultation with a cardiologist if appropriate.

- Known HIV positivity or AIDS-related illness
- Either a concurrent condition (including medical illness, such as active infection requiring treatment with intravenous antibiotics or the presence of laboratory abnormalities) or history of a prior condition that places the patient at unacceptable risk if he/she were treated with the study drug or confounds the ability to interpret data from the study
- Inability to comply with other requirements of the protocol

Additionally, patients who meet any of the cohort-specific exclusion criteria provided in the following appendices will be excluded from that respective treatment cohort:

- Cohort A (ALK+): Appendix 7, Section 11.4.1.2
- Cohort B (RET+): Enrollment closed
- Cohort C (bTMB+): Appendix 9, Section 13.4.1.2
- Cohort D (ROS1+): Appendix 10, Section 14.4.1.2
- Cohort E (BRAF V600+): Appendix 11, Section 15.4.1.2
- *Cohort F (EGFR exon 20+): Appendix 12, Section* 16.4.1.2

To be enrolled in a treatment cohort, patients must have met and continue to meet all eligibility criteria specified above for the blood-based NGS ctDNA assay screening.

"Use of this document is governed by the terms of use on the first page of this document."

4.2 METHOD OF TREATMENT ASSIGNMENT

All patients will be assigned to a specific treatment cohort based on the mutation or biomarker results from the blood-based assay (see Section 3.1.1).

Blood-based screening patients who are diagnostic positive for a treatment cohort must then consent for the applicable cohort after being informed of the cohort-specific details, procedures to be followed, the experimental nature of the treatment, potential benefits, alternatives, side-effects, risks, and discomforts. An interactive voice or Web-based response system (IxRS) vendor will be used to manage patient screening, enrollment and tracking. Patients must be registered via the IxRS prior to the initiation of blood-based screening.

For treatment cohorts that will be randomized, please see treatment assignment details in the respective cohort-specific appendix.

4.3 STUDY TREATMENT AND OTHER TREATMENTS RELEVANT TO THE STUDY DESIGN

Patients may be assigned to a treatment cohort based on the results of the blood-based screening assay (see Section 3.1.1).

Because of the differences in study design between the treatment cohorts, the study designs are provided separately in the appendices as follows:

- Cohort A (ALK+): Appendix 7, Section 11.4.2
- Cohort B (RET+): Appendix 8, Section 12.4.1
- Cohort C (bTMB+): Appendix 9, Section 13.4.3
- Cohort D (ROS1+): Appendix 10, Section 14.4.2
- Cohort E (BRAF V600+): Appendix 11, Section 15.4.2
- *Cohort F (EGFR exon 20+): Appendix 12, Section* 16.4.2

Details of each study treatment, study schema, recommended concomitant medications, and prohibited medications are also contained in the appendices.

Consenting patients with a key alteration profile of interest who, for whatever reason, are not enrolled in one of the treatment cohorts will not receive study treatment in this study but will enter natural history follow-up (see Section 3.1.1).

4.3.1 Study Treatment Formulation, Packaging, and Handling

Please refer to the cohort-specific appendices for details.

4.3.2 Study Treatment Dosage, Administration, and Compliance

The treatment regimens are summarized in the cohort-specific appendices.

"Use of this document is governed by the terms of use on the first page of this document."

Any overdose or incorrect administration of any of the study treatments should be noted on the Study Drug Administration electronic Case Report Form (eCRF). Adverse events associated with an overdose or incorrect administration of any of the study treatments should be recorded on the Adverse Event eCRF. Section 5.3.5.12 provides further details related to overdosing of any study treatment.

4.3.3 Investigational Medicinal Product Accountability

All investigational medicinal products (IMPs) required for completion of this study will be provided by the Sponsor where required by local health authority regulations. The IMPs for this study include the following:

- Cohorts A and B: alectinib
- Cohort C: atezolizumab, cisplatin, carboplatin, gemcitabine, and pemetrexed
- Cohort D: entrectinib
- Cohort E: vemurafenib, cobimetinib, and atezolizumab
- *Cohort F: atezolizumab, bevacizumab, carboplatin, and pemetrexed*

The study site will acknowledge receipt of IMPs using the IxRS to confirm the shipment condition and content. Any damaged shipments will be replaced.

IMPs either will be disposed of at the study site according to the study site's institutional standard operating procedure or will be returned to the Sponsor with the appropriate documentation. The site's method of IMP destruction must be agreed to by the Sponsor. The site must obtain written authorization from the Sponsor before any IMP is destroyed, and IMP destruction must be documented on the appropriate form.

Accurate records of all IMPs received at, dispensed from, returned to, and disposed of by the study site should be recorded on the Drug Inventory Log.

Patient compliance in taking daily doses of cohort-specific oral medicinal product(s) will be assessed by standard pill counts. Bottles and blister packages containing tablets or capsules will be given to patients at regularly scheduled visits. Previously distributed bottles and blister packages will be returned to the clinic and counted; discrepancies will be resolved with the patient at each clinic visit and documented in the patient's medical chart.

4.3.4 Continued Access to Study Drugs

Currently, the Sponsor does not have any plans to provide study treatments or interventions to patients who have completed the study. The Sponsor may evaluate the appropriateness of continuing to provide the drugs used during the study after evaluation of the primary and secondary efficacy outcome measures and safety data gathered in

"Use of this document is governed by the terms of use on the first page of this document."

the study and in accordance with the Roche Global Policy on Continued Access to Investigational Medicinal Product, available at the following Web site:

http://www.roche.com/policy_continued_access_to_investigational_medicines.pdf

These analyses may be conducted prior to completion of the study.

Patients may also continue to receive study treatments as part of an extension study, if applicable.

4.4 CONCOMITANT THERAPY

Concomitant therapy consists of any medication (e.g., prescription drugs, over-the-counter drugs, vaccines, herbal or homeopathic remedies, nutritional supplements) used by a patient from 7 days prior to the cohort-specific treatment consent to the study treatment completion/discontinuation visit. All such medications should be reported to the investigator and recorded on the Concomitant Medications eCRF.

For details on regimen-specific concomitant therapy, refer to the appropriate cohort-specific appendices.

4.5 STUDY ASSESSMENTS

The schedule of activities for initial screening to be performed for all patients is provided in Appendix 1.

For patients who are to be enrolled in one of the treatment cohorts, refer to the cohort-specific appendices for additional requirements. All activities must be performed and documented for each patient. Patients will be closely monitored for safety and tolerability throughout the study. Patients should be assessed for toxicity prior to each dose; dosing will occur only if the clinical assessment and local laboratory test values are acceptable.

For patients who are not enrolled in one of the treatment cohorts and enter natural history follow-up, the following information will be collected approximately every 3 months during routine doctor or healthcare appointments: cancer therapies (or therapy types), treatment response, and survival status. Additionally, a blood sample will be taken at the time of disease progression.

4.5.1 Informed Consent Forms and Screening Log

Written informed consent for participation in the study must be obtained before performing any study-specific procedures (including screening evaluations). Separate Informed Consent Forms will be required for initial blood screening and for enrollment into a specific treatment cohort. Informed Consent Forms for enrolled patients and for patients who are not subsequently enrolled will be maintained at the study site.

"Use of this document is governed by the terms of use on the first page of this document."

All screening evaluations must be completed and reviewed to confirm that patients meet all eligibility criteria before enrollment. The investigator will maintain a screening log to record details of all patients screened and to confirm eligibility or record reasons for screening failure, as applicable.

Patients who are treated with atezolizumab and who show apparent radiographic progression at a tumor-response evaluation must sign an Informed Consent Form at that time to acknowledge deferring other treatment options in favor of continuing treatment with atezolizumab (if that is the decision made at the time based on discussion between the treating physician and patient).

4.5.2 Medical History and Demographic Data

Medical history includes clinically significant diseases, surgeries, non-NSCLC cancer history (including prior cancer therapies and procedures), reproductive status, smoking history, use of alcohol and drugs of abuse, and all medications (e.g., prescription drugs, over-the-counter drugs, herbal or homeopathic remedies, nutritional supplements) used by the patient within 7 days prior to the initial screening visit. NSCLC cancer history will include information about the initial diagnosis, prior cancer therapies, and associated procedures (e.g., radiation, surgery, etc.).

Demographic data will include age, sex, and self-reported race/ethnicity. Additional demographic information regarding tissue availability will be collected for all patients during the screening period. Documentation must be obtained and data will be collected as to whether tissue is available for biomarker testing or if a tissue test for the biomarkers of interest (i.e., corresponding to open cohorts in the BFAST trial) has been performed. If a tissue test report is available, the result of the test, date of collection, and assay methodology (if clearly defined) will be captured. *Prior PD-L1 test results, if available, will also be recorded.*

4.5.3 Physical Examinations

A complete physical examination should be performed at screening for a treatment cohort and should include an evaluation of the head, eyes, ears, nose, and throat, and the cardiovascular, dermatological, musculoskeletal, respiratory, gastrointestinal, genitourinary, and neurological systems. Any abnormality identified at baseline should be recorded on the General Medical History and Baseline Conditions eCRF.

At subsequent visits (or as clinically indicated), limited, symptom-directed physical examinations should be performed. Changes from baseline abnormalities should be recorded in patient notes. New or worsened clinically significant abnormalities should be recorded as adverse events on the Adverse Event eCRF.

For Cohort D (ROS1+ patients receiving entrectinib), ophthalmologic examinations including at least the visual acuity and slit-lamp tests (which may be performed by an

"Use of this document is governed by the terms of use on the first page of this document."

optometrist) will be required. See the cohort-specific appendix and schedule of assessments for more details.

For Cohort E (BRAF V600+ patients receiving atezolizumab, vemurafenib, and cobimetinib), additional safety assessments and assessments to monitor for the development of new primary neoplasms are required. Refer to the cohort-specific appendix and Section 15.4.4 for more details.

4.5.4 Vital Signs

Routine vital signs will include measurements of respiratory rate, pulse rate, and systolic and diastolic blood pressure while the patient is in a seated position, and temperature.

Certain cohorts of this umbrella trial will require additional or more intensive vital sign monitoring (based on the properties and known profiles of the agents in individual cohorts). Please refer to cohort-specific appendices (and the associated schedules of activities) for more detail as necessary.

4.5.5 Tumor and Response Evaluations

Patients will undergo tumor assessments at baseline and periodically according to the cohort-specific schedules of assessment (provided in the cohort-specific appendices), regardless of dose delays, until radiographic disease progression (or loss of clinical benefit for patients in Cohort C *or Cohort F* receiving atezolizumab beyond disease progression per RECIST v1.1) as determined by the investigator, death, withdrawal of consent, or study termination by the Sponsor, whichever occurs first. Thus, tumor assessments are to continue according to the schedule in patients who discontinue treatment for reasons other than disease progression or loss of clinical benefit for patients receiving atezolizumab beyond disease progression per RECIST v1.1, death, withdrawal of consent, or study termination by the Sponsor whichever occurs first, even if they start new anti-cancer therapy. At the investigator’s discretion, tumor assessments may be repeated at any time if progressive disease is suspected.

All measurable and evaluable lesions should be assessed and documented at screening. Tumor assessments performed as standard of care prior to obtaining informed consent and within 28 days prior to initiation of study treatment do not have to be repeated at baseline.

Screening assessments must include computed tomography (CT) scans (with oral or IV contrast) or magnetic resonance imaging (MRI) scans of the chest, abdomen, pelvis, and head. Pelvic imaging beyond screening is required only as clinically indicated or as per local standard of care. A spiral CT scan of the chest may be obtained but is not a requirement. If a CT scan with contrast is contraindicated (i.e., in patients with contrast allergy or impaired renal clearance), a non-contrast CT scan of the chest may be performed and MRI scans of the abdomen, pelvis, and head should be performed.

"Use of this document is governed by the terms of use on the first page of this document."

A CT scan with contrast or MRI scan of the head must be done at screening to evaluate CNS metastasis in all patients (MRI scan must be performed if contrast is contraindicated). An MRI scan of the head is required to confirm or refute the diagnosis of CNS metastases at baseline in the event of an equivocal CT scan. Bone scans and CT scans of the neck should also be performed if clinically indicated.

If a CT scan for tumor assessment is performed in a positron emission tomography/CT scanner, the CT acquisition must be consistent with the standards for a full-contrast diagnostic CT scan.

All measurable and evaluable lesions should be re-assessed at each subsequent tumor evaluation. In Cohort C, patients with a history of irradiated brain metastases at screening are not required to undergo brain scans at subsequent tumor evaluations unless scans are clinically indicated (see Appendix 9 for more details). *In Cohort F, imaging of the brain is required at each tumor assessment (see* Appendix 12 *for more details).*

The same radiographic procedures used to assess disease sites at screening should be used for subsequent tumor assessments (e.g., the same contrast protocol for CT scans).

Response will be assessed by the investigator using RECIST v1.1 (see Appendix 3). Assessments should be performed by the same evaluator, if possible, to ensure internal consistency across visits. Results must be reviewed by the investigator before dosing at the next cycle.

Scans will be submitted to an independent review facility (IRF) for central review.

4.5.6 Laboratory, Biomarker, and Other Biological Samples

Samples for the following laboratory tests will be sent to the study site's local laboratory for analysis:

- Hematology (CBC, including RBC count, hemoglobin, hematocrit, WBC count with differential [neutrophils, eosinophils, lymphocytes, monocytes, basophils, and other cells], and platelet count)
- Serum chemistries (glucose, BUN or urea, creatinine, sodium, potassium, magnesium, chloride, bicarbonate or total CO_2, calcium, phosphorus, total bilirubin, ALT, AST, alkaline phosphatase, LDH, total protein, and albumin)
- Serum pregnancy test at baseline for women of childbearing potential, including women who have had a tubal ligation; during subsequent cycles, urine or serum pregnancy tests will be performed according to the cohort-specific appendices

  Refer to Appendix 2 and cohort-specific appendices for additional details.
- Urinalysis (specific gravity, pH, glucose, protein, ketones, and blood); dipstick permitted

"Use of this document is governed by the terms of use on the first page of this document."

- Cohorts A and B only (alectinib-containing cohorts):

  CPK

- Cohort C only (atezolizumab-containing cohort)
  - Thyroid function testing (thyroid-stimulating hormone, free T3, free T4); total T3 will be tested only at sites where free T3 is not performed.
  - Coagulation (INR or aPTT)
  - HIV serology
  - Hepatitis B virus (HBV) serology: hepatitis B surface antigen (HBsAg), total hepatitis B core antibody (HBcAb), and (if HBsAg test is negative and total HBcAb test is positive) HBV DNA

    If a patient has a negative HBsAg test and a positive total HBcAb test at screening, an HBV DNA test must also be performed to determine if the patient has an HBV infection.
  - Hepatitis C virus (HCV) serology: HCV antibody and (if HCV antibody test is positive) HCV RNA

    If a patient has a positive HCV antibody test at screening, an HCV RNA test must also be performed to determine if the patient has an HCV infection.
- Cohort D only (entrectinib-containing cohort)
  - Uric acid
  - Coagulation (INR, aPTT)
  - Lipids (total cholesterol, LDL, HDL, triglycerides)
- Cohort E only
  - Coagulation (INR, aPTT)
  - Lipids (total cholesterol, LDL, HDL, triglycerides)
  - CPK
  - Amylase
  - Lipase
  - Thyroid function testing (thyroid-stimulating hormone, free T3, free T4); total T3 will be tested only at sites where free T3 is not performed.
  - HIV serology
  - Hepatitis B virus (HBV) serology: hepatitis B surface antigen (HBsAg), total hepatitis B core antibody (HBcAb), and (if HBsAg test is negative and total HBcAb test is positive) HBV DNA

    If a patient has a negative HBsAg test and a positive total HBcAb test at screening, a HBV DNA test must also be performed to determine if the patient has a HBV infection.

"Use of this document is governed by the terms of use on the first page of this document."

- Hepatitis C virus (HCV) serology: HCV antibody and (if HCV antibody test is positive) HCV RNA

  If a patient has a positive HCV antibody test at screening, an HCV RNA test must also be performed to determine if the patient has an HCV infection.

- *Cohort F Only*
  - *Coagulation (aPTT or INR)*
  - *Thyroid function testing (thyroid-stimulating hormone, free T3, free T4); total T3 will be tested only at sites where free T3 is not performed*
  - *HIV testing*
  - *Hepatitis B virus (HBV) serology: HBsAg, HBsAb, and total HBcAb for all patients; HBV DNA for patients with negative HBsAg and HBsAb tests and a positive total HBcAb test*
  - *Hepatitis C virus (HCV) serology: HCV antibody for all patients; HCV RNA for patients with a positive HCV antibody test*

    If a patient has a positive HCV antibody test at screening, an HCV RNA test must also be performed to determine if the patient has an HCV infection

A central laboratory will coordinate the sample collection of blood and/or optional tissue samples for research-related testing at central laboratories or at the Sponsor. Instruction manuals and supply kits will be provided for all central laboratory assessments. Samples for the following laboratory tests will be sent to one or several central laboratories or to the Sponsor for analysis:

- Serum samples for assessment of anti-drug antibodies (ADAs) (if applicable, depending on cohort)
- Pharmacokinetic (PK) assay (if applicable, depending on cohort)

  If applicable, blood samples for PK assessments will be obtained according to the schedule in the cohort. Plasma concentrations for the study drug (and its metabolite[s], if applicable) will be measured by specific and validated methods.

- Biomarker assays

  Blood samples will be obtained for biomarker evaluation (including but not limited to biomarkers related to NSCLC or tumor immune biology) from all eligible patients according to the schedules of activities in the cohort-specific appendices. Samples will be processed to obtain plasma for the determination of changes in blood-based biomarkers (e.g., ctDNA). Whole-blood samples may be processed to obtain their derivatives (e.g., RNA and DNA) and evaluated for immune-related, tumor type−related, and other exploratory biomarkers (e.g., alterations in gene expression or single-nucleotide polymorphisms). For patients who consent to submit their whole-blood samples at screening but are determined to be ineligible for study participation, these

"Use of this document is governed by the terms of use on the first page of this document."

samples and their derivatives (e.g., DNA, RNA, protein) may be used for development of biomarker and/or diagnostic tests within the scope of the trial objectives.

Any remaining samples collected for biomarker assays may be used for exploratory biomarker profiling, identification, and pharmacodynamic assay development purposes as appropriate.

For patients who consent to the optional collection of samples for the Research Biosample Repository (RBR), any leftover material from the above sample collection will be stored and used for exploratory analyses as indicated in Section 4.5.10.

Refer to the laboratory manual for additional details on laboratory assessments and sample handling.

Blood samples will be collected at screening, baseline, at predefined timepoints during treatment (please refer to the cohort-specific schedules of activities) and at the time of disease progression for DNA extraction to enable analysis via NGS to identify somatic mutations or signatures of mutations that are predictive of response to study drug, are associated with disease progression, are associated with acquired resistance to the study medications, or that can increase the knowledge and understanding of disease biology. Methods for exploratory analysis include, but are not limited to, NGS, polymerase chain reaction (PCR), and proteomics-based approaches.

Tissue biopsies (tissue blocks or unstained slides) may be submitted at any time during the study, with fresh or archival tissue being acceptable. These samples are considered optional, and biopsy or tissue is not required; although, if archival tissue is available, it should be submitted if the patient also consented to RBR. Methods for exploratory analysis include, but are not limited to, NGS, PCR, and proteomics-based approaches. NGS may be performed by Foundation Medicine for exploratory purposes at some point during the study.

In this study, NGS will be performed by Foundation Medicine. For all patients who are screened using the blood-based assay, investigators can obtain results from these analyses in the form of *a BFAST-specific* NGS report, which is available via an online portal maintained by Foundation Medicine. For patients enrolled in the trial, at the time of disease progression (or loss of clinical benefit in patients receiving atezolizumab beyond disease progression per RECIST v1.1 in Cohort C *or Cohort F*), a blood sample will be taken for testing using the blood-based assay. The investigator can obtain results from these analyses in the form of *a BFAST-specific* NGS report, which is available via an online portal maintained by Foundation Medicine. If allowed by local laws, the investigator may share and discuss the results with the patient, unless the patient chooses otherwise. The Foundation Medicine NGS *test FoundationOne® Liquid Companion Diagnostic* assay *(F1LCDx) is FDA-*approved *and CE-marked.* The *BFAST-specific* NGS report is generated for research purposes and is not provided for

"Use of this document is governed by the terms of use on the first page of this document."

the purpose of guiding future treatment decisions. Results may not be available for samples that do not meet testing criteria.

4.5.7 Electrocardiograms

A twelve-lead electrocardiogram (ECG) is required at screening for a particular treatment cohort (i.e., not required at time of blood-based screening), and when clinically indicated. ECGs for each patient should be obtained from the same machine wherever possible. Lead placement should be as consistent as possible. ECG recordings must be performed after the patient has been resting in a supine position for at least 10 minutes. All ECGs will to be obtained prior to other procedures scheduled at that same time (e.g., vital sign measurements, blood draws, study drug administration), as well as prior to meals. Circumstances that may induce changes in heart rate, including environmental distractions (e.g., television, radio, conversation), should be avoided during the pre-ECG resting period and during ECG recording.

For safety monitoring purposes, the investigator must review, sign, and date all ECG tracings. Paper copies of ECG tracings will be kept as part of the patient's permanent study file at the site. Any morphologic waveform changes or other ECG abnormalities must be documented on the eCRF.

Refer to cohort-specific appendices for schedule and additional details.

4.5.8 Left Ventricular Ejection Fraction

Patients who are eligible to be enrolled in certain treatment cohorts will undergo evaluation of LVEF, either by ECHO or MUGA, at specified timepoints during the study, and as clinically indicated for new or worsening symptoms. Any patient who develops clinical signs or symptoms suspicious of cardiac failure should undergo an LVEF assessment. Evaluation of LVEF should be performed using the same method for each patient.

Not all treatment cohorts in the study will require LVEF assessment. Currently, LVEF assessment is mandatory in the following cohorts:

- Cohort D (ROS1+ patients receiving entrectinib)
- Cohort E (BRAF V600+ patients receiving atezolizumab, vemurafenib, and cobimetinib)

Please refer to cohort-specific appendices for schedule and additional details.

4.5.9 Patient-Reported Outcomes

PRO questionnaires will be collected at the investigational site to more fully characterize the clinical profile of the study drugs. The questionnaires will be translated as required into the local language. PRO questionnaires scheduled for administration during a clinic visit are required to be completed by the patient at the investigational site at the start of

"Use of this document is governed by the terms of use on the first page of this document."

the clinic visit before discussion of the patient's health state, laboratory results or health record, before administration of study treatment, and/or prior to any other study assessment(s) that could bias patients' responses to ensure that the validity of the instrument is not compromised and that data quality meets regulatory requirements.

Adverse event reports will not be derived from PRO data by the Sponsor. However, any PRO responses suggestive of a possible adverse event that are identified during site review of the PRO data should be reported as outlined in Section 5.3.5.13.

Patients whose native language is not available on the PRO instruments or who are deemed by the investigator incapable of completing their PRO assessments after undergoing appropriate training are exempted from completing all PRO assessments.

The following PRO instruments will be utilized in the BFAST study. The instruments to be used in each cohort are specific to the given cohorts; please refer to the respective appendices for details.

4.5.9.1 SILC

The SILC scale (see Appendix 6) will be used to assess patient-reported severity of lung cancer symptoms (cough, dyspnea, and chest pain). The SILC scale is a nine-item content valid self-report measure of lung cancer symptoms. It measures severity of cough, dyspnea, and chest pain with a total symptom severity score.

4.5.9.2 EORTC QLQ-C30

The EORTC QLQ-C30 (see Appendix 6) is a validated and reliable self-report measure (Aaronson et al. 1993; Fitzsimmons et al. 1999) that consists of 30 questions that assess five aspects of patient functioning (physical, emotional, role, cognitive, and social), three symptom scales (fatigue, nausea and vomiting, pain), global health/quality of life, and six single items (dyspnea, insomnia, appetite loss, constipation, diarrhea, and financial difficulties). Scale scores can be obtained for the multi-item scales. The EORTC QLQ-C30 module takes approximately 10 minutes to complete.

4.5.9.3 EORTC QLQ-BN20

Selected items (Items 34, 36–38) from the EORTC QLQ-BN20 (see Appendix 6) self-reported measure will be used to assess the patient-relevant impact of the CNS activity observed with entrectinib. The EORTC QLQ-BN20 is valid and reliable for use in primary brain tumors (Taphoorn et al. 2010); therefore, the selected items will be used in an exploratory manner.

4.5.9.4 EQ-5D-5L

The EQ-5D-5L is a generic, preference-based health utility measure with questions about mobility, self-care, usual activities, pain/discomfort, and anxiety/depression that is used to build a composite of the patient's health status (see Appendix 6). The EQ-5D-5L will be utilized in this study for economic modeling.

"Use of this document is governed by the terms of use on the first page of this document."

4.5.10 Optional Samples for Research Biosample Repository

4.5.10.1 Overview of the Research Biosample Repository

The RBR is a centrally administered group of facilities used for the long-term storage of human biologic specimens, including body fluids, solid tissues, and derivatives thereof (e.g., DNA, RNA, proteins, peptides). The collection, storage, and analysis of RBR specimens will facilitate the rational design of new pharmaceutical agents and the development of diagnostic tests, which may allow for individualized drug therapy for patients in the future.

Specimens for the RBR will be collected from patients who give specific consent to participate in this optional research. RBR specimens will be used to achieve the following objectives:

- To study the association of biomarkers with efficacy, adverse events, or disease progression
- To increase knowledge and understanding of disease biology
- To study drug response, including drug effects and the processes of drug absorption and disposition
- To develop biomarker or diagnostic assays and establish the performance characteristics of these assays

4.5.10.2 Approval by the Institutional Review Board or Ethics Committee

Collection and submission of biological samples to the RBR is contingent upon the review and approval of the exploratory research and the RBR portion of the Informed Consent Form by each site's Institutional Review Board or Ethics Committee (IRB/EC) and, if applicable, an appropriate regulatory body. If a site has not been granted approval for RBR sampling, this section of the protocol (Section 4.5.10) will not be applicable at that site.

4.5.10.3 Sample Collection

The following samples will be stored in the RBR and used for research purposes, including, but not limited to, research on biomarkers related to NSCLC:

- Leftover blood, plasma, and tumor tissue samples, including, but not limited to, the derivatives of these samples.

The above samples may be sent to one or more laboratories for analysis of germline or somatic mutations via whole genome sequencing (WGS), NGS, or other genomic analysis methods.

Genomics is increasingly informing researchers' understanding of disease pathobiology. WGS and WES provide a comprehensive characterization of the genome and exome, respectively, and, along with clinical data collected in this study, may increase the opportunity for developing new therapeutic approaches. Data will be analyzed in the context of this study but will also be explored in aggregate with data from other studies.

"Use of this document is governed by the terms of use on the first page of this document."

The availability of a larger dataset will assist in identification of important pathways, guiding the development of new targeted agents.

For sampling procedures, storage conditions, and shipment instructions, see the laboratory manual.

RBR specimens are to be stored until they are no longer needed or until they are exhausted. However, the RBR storage period will be in accordance with the IRB/EC–approved Informed Consent Form and applicable laws (e.g., health authority requirements).

4.5.10.4 Confidentiality

Specimens and associated data will be labeled with a unique patient identification number.

Patient medical information associated with RBR specimens is confidential and may be disclosed to third parties only as permitted by the Informed Consent Form (or separate authorization for use and disclosure of personal health information) signed by the patient, unless permitted or required by law.

Given the complexity and exploratory nature of the analyses of RBR specimens, data derived from these analyses will generally not be provided to study investigators or patients unless required by law. The aggregate results of any conducted research will be available in accordance with the effective Sponsor policy on study data publication.

Data generated from RBR specimens must be available for inspection upon request by representatives of national and local health authorities, and Sponsor monitors, representatives, and collaborators, as appropriate.

Any inventions and resulting patents, improvements, and/or know-how originating from the use of the RBR data will become and remain the exclusive and unburdened property of the Sponsor, except where agreed otherwise.

4.5.10.5 Consent to Participate in the Research Biosample Repository

The Informed Consent Form will contain a separate section that addresses participation in the RBR. The investigator or authorized designee will explain to each patient the objectives, methods, and potential hazards of participation in the RBR. Patients will be told that they are free to refuse to participate and may withdraw their specimens at any time and for any reason during the storage period. A separate, specific signature will be required to document a patient's agreement to provide optional RBR specimens. Patients who decline to participate will not provide a separate signature.

The investigator should document whether or not the patient has given consent to participate and (if applicable) the date(s) of consent, by completing the RBR Research Sample Informed Consent eCRF.

"Use of this document is governed by the terms of use on the first page of this document."

In the event of an RBR participant's death or loss of competence, the participant's specimens and data will continue to be used as part of the RBR research.

4.5.10.6 Withdrawal from the Research Biosample Repository

Patients who give consent to provide RBR samples have the right to withdraw their consent at any time for any reason. After withdrawal of consent, any remaining samples will be destroyed or will no longer be linked to the patient. However, if RBR samples have been tested prior to withdrawal of consent, results from those tests will remain as part of the overall research data. If a patient wishes to withdraw consent to the testing of his or her RBR samples during the study, the investigator must inform the Medical Monitor in writing of the patient's wishes through use of the appropriate RBR Subject Withdrawal Form and must enter the date of withdrawal on the RBR Research Sample Withdrawal of Informed Consent eCRF. If a patient wishes to withdraw consent to the testing of his or her RBR samples after closure of the site, the investigator must inform the Sponsor by emailing the study number and patient number to the following email address:

global_rcr-withdrawal@roche.com

A patient's withdrawal from this study does not, by itself, constitute withdrawal of consent for testing of RBR samples. Likewise, a patient's withdrawal of consent for testing of RBR samples does not constitute withdrawal from this study.

4.5.10.7 Monitoring and Oversight

RBR specimens will be tracked in a manner consistent with Good Clinical Practice by a quality-controlled, auditable, and appropriately validated laboratory information management system, to ensure compliance with data confidentiality as well as adherence to authorized use of specimens as specified in this protocol and in the Informed Consent Form. Sponsor monitors and auditors will have direct access to appropriate parts of records relating to patient participation in the RBR for the purposes of verifying the data provided to the Sponsor. The site will permit monitoring, audits, IRB/EC review, and health authority inspections by providing direct access to source data and documents related to the RBR samples.

4.6 TREATMENT, PATIENT, STUDY, AND SITE DISCONTINUATION

4.6.1 Study Treatment Discontinuation

Patients must permanently discontinue study treatment if they experience any of the following:

- Any medical condition that the investigator or Sponsor determines may jeopardize the patient’s safety if he or she continues study treatment
- Investigator or Sponsor determines it is in the best interest of the patient
- No longer continuing to meet eligibility criteria, or unwillingness to comply with study assessments

"Use of this document is governed by the terms of use on the first page of this document."

- Symptomatic deterioration attributed to disease progression as determined by the investigator after integrated assessment of radiographic data, biopsy results (if available), and clinical status
- Intolerable toxicity related to any study treatment
- Use of another non−protocol-specified anti-cancer therapy (see Prohibited Therapy sections in cohort-specific appendices)
- Pregnancy
- Radiographic disease progression per RECIST v1.1 (see cohort-specific appendices for exceptions)

Additional cohort-specific study treatment discontinuation criteria are detailed in the cohort-specific appendices.

The primary reason for study drug discontinuation should be documented on the appropriate eCRF. Patients who discontinue study treatment will not be replaced.

4.6.2 Patient Discontinuation from the Study

Patients have the right to voluntarily withdraw from the study at any time for any reason. In addition, the investigator has the right to withdraw a patient from the study at any time. Reasons for withdrawal from the study may include, but are not limited to, the following:

- Patient withdrawal of consent at any time
- Any medical condition that the investigator or Sponsor determines may jeopardize the patient's safety if he or she continues in the study
- Investigator or Sponsor determines it is in the best interest of the patient
- Patient non-compliance, defined as failure to comply with protocol requirements as determined by the investigator or Sponsor
- Study termination or site closure

Every effort should be made to obtain information on patients who withdraw from the study. The primary reason for withdrawal from the study should be documented in the source documents and on the appropriate eCRF. Patients who withdraw from the study will not be replaced.

Patients who wish to withdraw consent for the study may still be willing to provide long-term follow-up information such as survival status and can do so if they consent to a limited follow-up.

If a patient withdraws from the study, the study staff may use a public information source (e.g., county records) to obtain information about survival status.

"Use of this document is governed by the terms of use on the first page of this document."

4.6.3 Study Discontinuation

The Sponsor has the right to terminate this study at any time. Reasons for terminating the study may include, but are not limited to, the following:

- The incidence or severity of adverse events in this or other studies indicates a potential health hazard to patients.
- Patient enrollment is unsatisfactory.

The Sponsor will notify the investigator if the Sponsor decides to discontinue the study.

4.6.4 Site Discontinuation

The Sponsor has the right to close a site at any time. Reasons for closing a site may include, but are not limited to, the following:

- Excessively slow recruitment
- Poor protocol adherence
- Inaccurate or incomplete data recording
- Non-compliance with the International Council for Harmonisation (ICH) guideline for Good Clinical Practice
- No study activity (i.e., all patients have completed the study and all obligations have been fulfilled)

5. ASSESSMENT OF SAFETY

5.1 SAFETY PLAN

Several measures will be taken to ensure the safety of patients participating in this study. Eligibility criteria have been designed to exclude patients at higher risk for toxicities. Patients will undergo safety monitoring during the study, including assessment of the nature, frequency, and severity of adverse events. In addition, guidelines for managing adverse events, including criteria for dosage modification (if applicable) and treatment interruption or discontinuation, are provided below.

Guidelines for management of specific adverse events are provided in the cohort-specific appendices for each study drug.

5.2 SAFETY PARAMETERS AND DEFINITIONS

Safety assessments will consist of monitoring and recording adverse events, including serious adverse events and adverse events of special interest, performing protocol-specified safety laboratory assessments, measuring protocol-specified vital signs, and conducting other protocol-specified tests that are deemed critical to the safety evaluation of the study.

Certain types of events require immediate reporting to the Sponsor, as outlined in Section 5.4.

"Use of this document is governed by the terms of use on the first page of this document."

5.2.1 Adverse Events

According to the ICH guideline for Good Clinical Practice, an adverse event is any untoward medical occurrence in a clinical investigation subject administered a pharmaceutical product, regardless of causal attribution. An adverse event can therefore be any of the following:

- Any unfavorable and unintended sign (including an abnormal laboratory finding), symptom, or disease temporally associated with the use of a medicinal product, whether or not considered related to the medicinal product
- Any new disease or exacerbation of an existing disease (a worsening in the character, frequency, or severity of a known condition), except as described in Section 5.3.5.10
- Recurrence of an intermittent medical condition (e.g., headache) not present at baseline
- Any deterioration in a laboratory value or other clinical test (e.g., ECG, X-ray) that is associated with symptoms or leads to a change in study treatment or concomitant treatment or discontinuation from study drug
- Adverse events that are related to a protocol-mandated intervention, including those that occur prior to assignment of study treatment (e.g., screening invasive procedures such as biopsies or blood draws)

5.2.2 Serious Adverse Events (Immediately Reportable to the Sponsor)

A serious adverse event is any adverse event that meets any of the following criteria:

- Is fatal (i.e., the adverse event actually causes or leads to death)
- Is life-threatening (i.e., the adverse event, in the view of the investigator, places the patient at immediate risk of death)

  This does not include any adverse event that had it occurred in a more severe form or was allowed to continue might have caused death.
- Requires or prolongs inpatient hospitalization (see Section 5.3.5.11)
- Results in persistent or significant disability/incapacity (i.e., the adverse event results in substantial disruption of the patient's ability to conduct normal life functions)
- Is a congenital anomaly/birth defect in a neonate/infant born to a mother exposed to study drug
- Is a significant medical event in the investigator's judgment (e.g., may jeopardize the patient or may require medical/surgical intervention to prevent one of the outcomes listed above)

The terms "severe" and "serious" are not synonymous. Severity refers to the intensity of an adverse event (e.g., rated as mild, moderate, or severe, or according to National Cancer Institute Common Terminology Criteria for Adverse Events [NCI CTCAE]; see

"Use of this document is governed by the terms of use on the first page of this document."

Section 5.3.3); the event itself may be of relatively minor medical significance (such as severe headache without any further findings).

Severity and seriousness need to be independently assessed for each adverse event recorded on the eCRF.

Serious adverse events are required to be reported by the investigator to the Sponsor immediately (i.e., no more than 24 hours after learning of the event; see Section 5.4.2 for reporting instructions).

5.2.3 Adverse Events of Special Interest (Immediately Reportable to the Sponsor)

Adverse events of special interest are required to be reported by the investigator to the Sponsor immediately (i.e., no more than 24 hours after learning of the event; see Section 5.4.2 for reporting instructions). Adverse events of special interest for any cohort of this study include the following:

- Cases of potential drug-induced liver injury that include an elevated ALT or AST in combination with either an elevated bilirubin or clinical jaundice, as defined by Hy's Law (see Section 5.3.5.7)
- Suspected transmission of an infectious agent by the study treatment, as defined below

  Any organism, virus, or infectious particle (e.g., prion protein transmitting transmissible spongiform encephalopathy), pathogenic or non-pathogenic, is considered an infectious agent. A transmission of an infectious agent may be suspected from clinical symptoms or laboratory findings that indicate an infection in a patient exposed to a medicinal product. This term applies only when a contamination of the study drug is suspected.

Please refer to the following cohort-specific appendices for additional adverse events of special interest relevant to the respective study treatments:

- Cohort C (bTMB+) (Appendix 9; Section 13.5.2.1)
- Cohort E (BRAF V600+) (Appendix 11; Section 15.5.6)
- *Cohort F (EGFR exon 20+): Appendix 12, Section* 16.5.10.1

5.2.4 Selected Adverse Events

Additional data will be collected for selected adverse events. Please refer to the cohort-specific appendices for selected adverse events and their management.

5.3 METHODS AND TIMING FOR CAPTURING AND ASSESSING SAFETY PARAMETERS

The investigator is responsible for ensuring that all adverse events (see Section 5.2.1 for definition) are recorded on the Adverse Event eCRF and reported to the Sponsor in accordance with instructions provided in this section and in Sections 5.4–5.6.

"Use of this document is governed by the terms of use on the first page of this document."

For each adverse event recorded on the Adverse Event eCRF, the investigator will make an assessment of seriousness (see Section 5.2.2 for seriousness criteria), severity (see Section 5.3.3), and causality (see Section 5.3.4).

5.3.1 Adverse Event Reporting Period

Investigators will seek information on adverse events at each patient contact. All adverse events, whether reported by the patient or noted by study personnel, will be recorded in the patient's medical record and on the Adverse Event eCRF.

After informed consent has been obtained **but prior to initiation of study drug**, only serious adverse events caused by a protocol-mandated intervention (e.g., invasive procedures such as biopsies, discontinuation of medications) should be reported (see Section 5.4.2 for instructions for reporting serious adverse events).

For patients in the natural history cohort, only serious adverse events caused by protocol-mandated interventions (blood draws) should be reported, via the paper reporting process described in Section 5.4.2.1.

After initiation of study drug, all serious adverse events and adverse events of special interest, regardless of relationship to study drug, will be reported for a defined period; please refer to the cohort-specific appendices for details.

- Cohort A (alectinib): Appendix 7, Section 11.5.4
- Cohort B (alectinib): Appendix 8, Section 12.5.6
- Cohort C (atezolizumab or chemotherapy): Appendix 9, Section 13.5.2.2
- Cohort D (entrectinib): Appendix 10, Section 14.5.5
- Cohort E (vemurafenib, cobimetinib, atezolizumab): Appendix 11, Section 15.5.7
- *Cohort F (atezolizumab, bevacizumab, carboplatin, pemetrexed): Appendix 12, Section* 16.5.11

All other adverse events, regardless of relationship to study drug, will be reported until 30 days after the last dose of study drug, or initiation of new anti-cancer therapy, whichever occurs first.

Instructions for reporting adverse events that occur after the adverse event reporting period are provided in Section 5.6.

5.3.2 Eliciting Adverse Event Information

A consistent methodology of non-directive questioning should be adopted for eliciting adverse event information at all patient evaluation timepoints. Examples of non-directive questions include the following:

"How have you felt since your last clinic visit?"

"Have you had any new or changed health problems since you were last here?"

"Use of this document is governed by the terms of use on the first page of this document."

5.3.3 Assessment of Severity of Adverse Events

The adverse event severity grading scale for the NCI CTCAE (v4.0) will be used for assessing adverse event severity. Table 2 will be used for assessing severity for adverse events that are not specifically listed in the NCI CTCAE.

Table 2 Adverse Event Severity Grading Scale for Events Not Specifically Listed in NCI CTCAE

Grade	Severity
1	Mild; asymptomatic or mild symptoms; clinical or diagnostic observations only; or intervention not indicated
2	Moderate; minimal, local, or non-invasive intervention indicated; or limiting age-appropriate instrumental activities of daily living [a]
3	Severe or medically significant, but not immediately life-threatening; hospitalization or prolongation of hospitalization indicated; disabling; or limiting self-care activities of daily living [b, c]
4	Life-threatening consequences or urgent intervention indicated [d]
5	Death related to adverse event [d]

NCI CTCAE = National Cancer Institute Common Terminology Criteria for Adverse Events.

Note: Based on the most recent version of NCI CTCAE (v4.0), which can be found at: http://ctep.cancer.gov/protocolDevelopment/electronic_applications/ctc.htm

[a] Instrumental activities of daily living refer to preparing meals, shopping for groceries or clothes, using the telephone, managing money, etc.

[b] Examples of self-care activities of daily living include bathing, dressing and undressing, feeding oneself, using the toilet, and taking medications, as performed by patients who are not bedridden.

[c] If an event is assessed as a "significant medical event," it must be reported as a serious adverse event (see Section 5.4.2 for reporting instructions), per the definition of serious adverse event in Section 5.2.2.

[d] Grade 4 and 5 events must be reported as serious adverse events (see Section 5.4.2 for reporting instructions), per the definition of serious adverse event in Section 5.2.2.

5.3.4 Assessment of Causality of Adverse Events

Investigators should use their knowledge of the patient, the circumstances surrounding the event, and an evaluation of any potential alternative causes to determine whether an adverse event is considered to be related to the study drug, indicating "yes" or "no" accordingly. The following guidance should be taken into consideration:

- Temporal relationship of event onset to the initiation of study drug
- Course of the event, with special consideration of the effects of dose reduction, discontinuation of study drug, or reintroduction of study drug (as applicable)
- Known association of the event with the study drug or with similar treatments
- Known association of the event with the disease under study
- Presence of risk factors in the patient or use of concomitant medications known to increase the occurrence of the event

"Use of this document is governed by the terms of use on the first page of this document."

- Presence of non–treatment-related factors that are known to be associated with the occurrence of the event

For patients receiving combination therapy, causality will be assessed individually for each protocol-mandated therapy.

5.3.5 Procedures for Recording Adverse Events

Investigators should use correct medical terminology/concepts when recording adverse events on the Adverse Event eCRF. Avoid colloquialisms and abbreviations.

Only one adverse event term should be recorded in the event field on the Adverse Event eCRF.

5.3.5.1 Infusion-Related Reactions (to Parenteral Study Treatment)

Adverse events that occur during or within 24 hours after study drug administration should be captured as individual signs and symptoms on the Adverse Event eCRF rather than an overall diagnosis (e.g., record dyspnea and hypotension as separate events rather than a diagnosis of infusion-related reaction).

5.3.5.2 Diagnosis versus Signs and Symptoms

For adverse events other than infusion-related reactions (see Section 5.3.5.1), a diagnosis (if known) should be recorded on the Adverse Event eCRF rather than individual signs and symptoms (e.g., record only liver failure or hepatitis rather than jaundice, asterixis, and elevated transaminases). However, if a constellation of signs and/or symptoms cannot be medically characterized as a single diagnosis or syndrome at the time of reporting, each individual event should be recorded on the Adverse Event eCRF. If a diagnosis is subsequently established, all previously reported adverse events based on signs and symptoms should be nullified and replaced by one adverse event report based on the single diagnosis, with a starting date that corresponds to the starting date of the first symptom of the eventual diagnosis.

5.3.5.3 Adverse Events That Are Secondary to Other Events

In general, adverse events that are secondary to other events (e.g., cascade events or clinical sequelae) should be identified by their primary cause, with the exception of severe or serious secondary events. A medically significant secondary adverse event that is separated in time from the initiating event should be recorded as an independent event on the Adverse Event eCRF. For example:

- If vomiting results in mild dehydration with no additional treatment in a healthy adult, only vomiting should be reported on the eCRF.
- If vomiting results in severe dehydration, both events should be reported separately on the eCRF.
- If a severe gastrointestinal hemorrhage leads to renal failure, both events should be reported separately on the eCRF.

"Use of this document is governed by the terms of use on the first page of this document."

- If dizziness leads to a fall and consequent fracture, all three events should be reported separately on the eCRF.
- If neutropenia is accompanied by an infection, both events should be reported separately on the eCRF.

All adverse events should be recorded separately on the Adverse Event eCRF if it is unclear as to whether the events are associated.

5.3.5.4 Persistent or Recurrent Adverse Events

A persistent adverse event is one that extends continuously, without resolution, between patient evaluation timepoints. Such events should only be recorded once on the Adverse Event eCRF. The initial severity (intensity or grade) of the event will be recorded at the time the event is first reported. If a persistent adverse event becomes more severe, the most extreme severity should also be recorded on the Adverse Event eCRF. If the event becomes serious, it should be reported to the Sponsor immediately (i.e., no more than 24 hours after learning that the event became serious; see Section 5.4.2 for reporting instructions). The Adverse Event eCRF should be updated by changing the event from "non-serious" to "serious," providing the date that the event became serious, and completing all data fields related to serious adverse events.

A recurrent adverse event is one that resolves between patient evaluation timepoints and subsequently recurs. Each recurrence of an adverse event should be recorded as a separate event on the Adverse Event eCRF.

5.3.5.5 Abnormal Laboratory Values

Not every laboratory abnormality qualifies as an adverse event. A laboratory test result must be reported as an adverse event if it is a change from baseline and meets any of the following criteria:

- Is accompanied by clinical symptoms
- Results in a change in study treatment (e.g., dosage modification, treatment interruption, or treatment discontinuation)
- Results in a medical intervention (e.g., potassium supplementation for hypokalemia) or a change in concomitant therapy
- Is clinically significant in the investigator's judgment

    Note: For oncology trials, certain abnormal values may not qualify as adverse events.

It is the investigator's responsibility to review all laboratory findings. Medical and scientific judgment should be exercised in deciding whether an isolated laboratory abnormality should be classified as an adverse event.

"Use of this document is governed by the terms of use on the first page of this document."

If a clinically significant laboratory abnormality is a sign of a disease or syndrome (e.g., alkaline phosphatase and bilirubin 5 × ULN associated with cholestasis), only the diagnosis (i.e., cholestasis) should be recorded on the Adverse Event eCRF.

If a clinically significant laboratory abnormality is not a sign of a disease or syndrome, the abnormality itself should be recorded on the Adverse Event eCRF, along with a descriptor indicating whether the test result is above or below the normal range (e.g., "elevated potassium," as opposed to "abnormal potassium"). If the laboratory abnormality can be characterized by a precise clinical term per standard definitions, the clinical term should be recorded as the adverse event. For example, an elevated serum potassium level of 7.0 mEq/L should be recorded as "hyperkalemia."

Observations of the same clinically significant laboratory abnormality from visit to visit should only be recorded once on the Adverse Event eCRF (see Section 5.3.5.4 for details on recording persistent adverse events).

5.3.5.6 Abnormal Vital Sign Values

Not every vital sign abnormality qualifies as an adverse event. A vital sign result must be reported as an adverse event if it is a change from baseline and meets any of the following criteria:

- Is accompanied by clinical symptoms
- Results in a change in study treatment (e.g., dosage modification, treatment interruption, or treatment discontinuation)
- Results in a medical intervention or a change in concomitant therapy
- Is clinically significant in the investigator's judgment

It is the investigator's responsibility to review all vital sign findings. Medical and scientific judgment should be exercised in deciding whether an isolated vital sign abnormality should be classified as an adverse event.

If a clinically significant vital sign abnormality is a sign of a disease or syndrome (e.g., high blood pressure), only the diagnosis (e.g., hypertension) should be recorded on the Adverse Event eCRF.

Observations of the same clinically significant vital sign abnormality from visit to visit should only be recorded once on the Adverse Event eCRF (see Section 5.3.5.4 for details on recording persistent adverse events).

5.3.5.7 Abnormal Liver Function Tests

The finding of an elevated ALT or AST ($>3\times$ baseline value) in combination with either an elevated total bilirubin ($>2\times$ ULN) or clinical jaundice in the absence of cholestasis or other causes of hyperbilirubinemia is considered to be an indicator of severe liver injury

"Use of this document is governed by the terms of use on the first page of this document."

(as defined by Hy's Law). Therefore, investigators must report as an adverse event the occurrence of either of the following:

- Treatment-emergent ALT or AST $> 3 \times$ baseline value in combination with total bilirubin $> 2 \times$ ULN (of which $\geq 35\%$ is direct bilirubin)
- Treatment-emergent ALT or AST $> 3 \times$ baseline value in combination with clinical jaundice

The most appropriate diagnosis or (if a diagnosis cannot be established) the abnormal laboratory values should be recorded on the Adverse Event eCRF (see Section 5.3.5.2) and reported to the Sponsor immediately (i.e., no more than 24 hours after learning of the event), either as a serious adverse event or an adverse event of special interest (see Section 5.4.2).

5.3.5.8 Deaths

For this protocol, mortality is an efficacy endpoint. Deaths that occur during the protocol-specified adverse event reporting period (see Section 5.3.1) that are attributed by the investigator solely to progression of NSCLC should be recorded on the Death Attributed to Progressive Disease eCRF. All other deaths that occur during the adverse event reporting period, regardless of relationship to study drug, must be recorded on the Adverse Event eCRF and immediately reported to the Sponsor (see Section 5.4.2).

Death should be considered an outcome and not a distinct event. The event or condition that caused or contributed to the fatal outcome should be recorded as the single medical concept on the Adverse Event eCRF. Generally, only one such event should be reported. If the cause of death is unknown and cannot be ascertained at the time of reporting, **"unexplained death"** should be recorded on the Adverse Event eCRF. If the cause of death later becomes available (e.g., after autopsy), "unexplained death" should be replaced by the established cause of death. The term **"sudden death"** should not be used unless combined with the presumed cause of death (e.g., "sudden cardiac death").

Deaths that occur after the adverse event reporting period should be reported as described in Section 5.6.

5.3.5.9 Preexisting Medical Conditions

A preexisting medical condition is one that is present at the screening visit for this study. Such conditions should be recorded on the General Medical History and Baseline Conditions eCRF.

A preexisting medical condition should be recorded as an adverse event <u>only</u> if the frequency, severity, or character of the condition worsens during the study. When recording such events on the Adverse Event eCRF, it is important to convey the concept that the preexisting condition has changed by including applicable descriptors (e.g., "more frequent headaches").

"Use of this document is governed by the terms of use on the first page of this document."

5.3.5.10 Lack of Efficacy or Worsening of NSCLC

Events that are clearly consistent with the expected pattern of progression of the underlying disease should not be recorded as adverse events. These data will be captured as efficacy assessment data only. In most cases, the expected pattern of progression will be based on RECIST v1.1. In rare cases, the determination of clinical progression will be based on symptomatic deterioration. However, every effort should be made to document progression through use of objective criteria. If there is any uncertainty as to whether an event is due to disease progression, it should be reported as an adverse event.

5.3.5.11 Hospitalization or Prolonged Hospitalization

Any adverse event that results in hospitalization (i.e., inpatient admission to a hospital) or prolonged hospitalization should be documented and reported as a serious adverse event (per the definition of serious adverse event in Section 5.2.2), except as outlined below.

An event that leads to hospitalization under the following circumstances should not be reported as an adverse event or a serious adverse event:

- Hospitalization for respite care
- Planned hospitalization required by the protocol
- Hospitalization for a preexisting condition, provided that all of the following criteria are met:
  - The hospitalization was planned prior to the study or was scheduled during the study when elective surgery became necessary because of the expected normal progression of the disease
  - The patient has not experienced an adverse event
- Hospitalization due solely to progression of the underlying cancer

An event that leads to hospitalization under the following circumstances is not considered to be a serious adverse event, but should be reported as an adverse event instead:

- Hospitalization that was necessary because of patient requirement for outpatient care outside of normal outpatient clinic operating hours

5.3.5.12 Adverse Events Associated with an Overdose or Error in Drug Administration

An overdose is the accidental or intentional use of a drug in an amount higher than the dose being studied. An overdose or incorrect administration of study treatment is not itself an adverse event, but it may result in an adverse event. All adverse events associated with an overdose or incorrect administration of study drug should be recorded on the Adverse Event eCRF. If the associated adverse event fulfills

"Use of this document is governed by the terms of use on the first page of this document."

seriousness criteria, the event should be reported to the Sponsor immediately (i.e., no more than 24 hours after learning of the event; see Section 5.4.2).

5.3.5.13 Patient-Reported Outcome Data

Adverse event reports will not be derived from PRO data by the Sponsor, and safety analyses will not be performed using PRO data. However, if any PRO responses suggestive of a possible adverse event are identified during site review of the PRO data, the investigator will determine whether the criteria for an adverse event have been met and, if so, will report the event on the Adverse Event eCRF.

5.4 IMMEDIATE REPORTING REQUIREMENTS FROM INVESTIGATOR TO SPONSOR

Certain events require immediate reporting to allow the Sponsor to take appropriate measures to address potential new risks in a clinical trial. The investigator must report such events to the Sponsor immediately; under no circumstances should reporting take place more than 24 hours after the investigator learns of the event. The following is a list of events that the investigator must report to the Sponsor within 24 hours after learning of the event, regardless of relationship to study drug:

- Serious adverse events (defined in Section 5.2.2; see Section 5.4.2 for details on reporting requirements)
- Adverse events of special interest (defined in Section 5.2.3; see Section 5.4.2 for details on reporting requirements)
- Dose-limiting toxicities (DLTs) during the DLT-assessment window (applicable to the following cohorts):

    Cohort B (RET+) (defined in Section 12.5.1; see Section 5.4.3 for details on reporting requirements)

- Pregnancies (see Section 5.4.4 for details on reporting requirements)

The investigator must report new significant follow-up information for these events to the Sponsor immediately (i.e., no more than 24 hours after becoming aware of the information). New significant information includes the following:

- New signs or symptoms or a change in the diagnosis
- Significant new diagnostic test results
- Change in causality based on new information
- Change in the event’s outcome, including recovery
- Additional narrative information on the clinical course of the event

Investigators must also comply with local requirements for reporting serious adverse events to the local health authority and IRB/EC.

"Use of this document is governed by the terms of use on the first page of this document."

5.4.1 Emergency Medical Contacts

Medical Monitor Contact Information for All Sites

Medical Monitor/Roche Medical Responsible: [redacted], *MBBS*., Ph.D., *MRCP (Primary)*

Telephone No.: [redacted]

Email: [redacted]

Medical Monitor: [redacted], Pharm.D. (Secondary)

Mobile Telephone No.: [redacted]

Email: [redacted]

To ensure the safety of study patients, an Emergency Medical Call Center Help Desk will access the Roche Medical Emergency List, escalate emergency medical calls, provide medical translation service (if necessary), connect the investigator with a Roche Medical Responsible (listed above and/or on the Roche Medical Emergency List), and track all calls. The Emergency Medical Call Center Help Desk will be available 24 hours per day, 7 days per week. Toll-free numbers for the Help Desk, as well as Medical Monitor and Medical Responsible contact information, will be distributed to all investigators.

5.4.2 Reporting Requirements for Serious Adverse Events and Adverse Events of Special Interest

5.4.2.1 Events That Occur Prior to Study Drug Initiation

After informed consent has been obtained but prior to initiation of study drug, only serious adverse events caused by a protocol-mandated intervention should be reported. The paper Clinical Trial Serious Adverse Event/Adverse Event of Special Interest Reporting Form provided to investigators should be completed and submitted to the Sponsor or its designee immediately (i.e., no more than 24 hours after learning of the event), either by faxing or by scanning and emailing the form using the fax number or email address provided to investigators.

5.4.2.2 Events That Occur After Study Drug Initiation

After initiation of study drug, serious adverse events and adverse events of special interest will be reported as in Section 5.3.1. Investigators should record all case details that can be gathered immediately (i.e., within 24 hours after learning of the event) on the Adverse Event eCRF and submit the report via the electronic data capture (EDC) system. A report will be generated and sent to Roche Safety Risk Management by the EDC system.

In the event that the EDC system is unavailable, the paper Clinical Trial Serious Adverse Event/Adverse Event of Special Interest Reporting Form provided to investigators should be completed and submitted to the Sponsor or its designee immediately (i.e., no more than 24 hours after learning of the event), either by faxing or by scanning and emailing the form using the fax number or email address provided to investigators. Once the

"Use of this document is governed by the terms of use on the first page of this document."

EDC system is available, all information will need to be entered and submitted via the EDC system.

Instructions and timing for reporting serious adverse events that occur after the last dose of study treatment are provided in Section 5.6 and in the cohort-specific appendices.

5.4.3 Dose-Limiting Toxicities (Immediately Reportable to the Sponsor)

For the following cohorts, adverse events identified as DLTs during the DLT assessment window are required to be reported by the investigator to the Sponsor immediately (i.e., no more than 24 hours after learning of the event; see Section 5.4.2 for reporting instructions):

- Cohort B (RET+): Appendix 8, Section 12.5.1

5.4.4 Reporting Requirements for Pregnancies

5.4.4.1 Pregnancies in Female Patients

Female patients of childbearing potential will be instructed to immediately inform the investigator if they become pregnant during the study or within the timeframe specified in Appendix 2 after the *final* dose of study drug. A Clinical Trial Pregnancy Reporting Form should be completed and submitted to the Sponsor or its designee immediately (i.e., no more than 24 hours after learning of the pregnancy), either by faxing or by scanning and emailing the form using the fax number or email address provided to investigators. Pregnancy should not be recorded on the Adverse Event eCRF. The investigator should discontinue study drug and counsel the patient, discussing the risks of the pregnancy and the possible effects on the fetus. Monitoring of the patient should continue until conclusion of the pregnancy. Any serious adverse events associated with the pregnancy (e.g., an event in the fetus, an event in the mother during or after the pregnancy, or a congenital anomaly/birth defect in the child) should be reported on the Adverse Event eCRF. In addition, the investigator will submit a Clinical Trial Pregnancy Reporting Form when updated information on the course and outcome of the pregnancy becomes available.

5.4.4.2 Pregnancies in Female Partners of Male Patients

Male patients will be instructed through the Informed Consent Form to immediately inform the investigator if their partner becomes pregnant during the study or within the timeframe specified in Appendix 2 after the *final* dose of study drug. A Clinical Trial Pregnancy Reporting Form should be completed and submitted to the Sponsor or its designee immediately (i.e., no more than 24 hours after learning of the pregnancy), either by faxing or by scanning and emailing the form using the fax number or email address provided to investigators. Attempts should be made to collect and report details of the course and outcome of any pregnancy in the partner of a male patient exposed to study drug. The pregnant partner will need to sign an Authorization for Use and Disclosure of Pregnancy Health Information to allow for follow-up on her pregnancy.

"Use of this document is governed by the terms of use on the first page of this document."

After the authorization has been signed, the investigator will submit a Clinical Trial Pregnancy Reporting Form when updated information on the course and outcome of the pregnancy becomes available. An investigator who is contacted by the male patient or his pregnant partner may provide information on the risks of the pregnancy and the possible effects on the fetus, to support an informed decision in cooperation with the treating physician and/or obstetrician.

5.4.4.3 Abortions

A spontaneous abortion should be classified as a serious adverse event (as the Sponsor considers abortions to be medically significant), recorded on the Adverse Event eCRF, and reported to the Sponsor immediately (i.e., no more than 24 hours after learning of the event; see Section 5.4.2).

If a therapeutic or elective abortion was performed because of an underlying maternal or embryofetal toxicity, the toxicity should be classified as a serious adverse event, recorded on the Adverse Event eCRF, and reported to the Sponsor immediately (i.e., no more than 24 hours after learning of the event; see Section 5.4.2). A therapeutic or elective abortion performed for reasons other than an underlying maternal or embryofetal toxicity is not considered an adverse event.

All abortions should be reported as pregnancy outcomes on the paper Clinical Trial Pregnancy Reporting Form.

5.4.4.4 Congenital Anomalies/Birth Defects

Any congenital anomaly/birth defect in a child born to a female patient exposed to study drug or the female partner of a male patient exposed to study drug should be classified as a serious adverse event, recorded on the Adverse Event eCRF, and reported to the Sponsor immediately (i.e., no more than 24 hours after learning of the event; see Section 5.4.2).

5.5 FOLLOW-UP OF PATIENTS AFTER ADVERSE EVENTS

5.5.1 Investigator Follow-Up

The investigator should follow each adverse event until the event has resolved to baseline grade or better, the event is assessed as stable by the investigator, the patient is lost to follow-up, or the patient withdraws consent. Every effort should be made to follow all serious adverse events considered to be related to study *treatment* or trial-related procedures until a final outcome can be reported.

During the *adverse event reporting* period *(defined in Section* 5.3.1 *),* resolution of adverse events (with dates) should be documented on the Adverse Event eCRF and in the patient's medical record to facilitate source data verification.

All pregnancies reported during the study should be followed until pregnancy outcome.

"Use of this document is governed by the terms of use on the first page of this document."

5.5.2 Sponsor Follow-Up

For serious adverse events, adverse events of special interest, and pregnancies, the Sponsor or a designee may follow up by telephone, fax, electronic mail, and/or a monitoring visit to obtain additional case details and outcome information (e.g., from hospital discharge summaries, consultant reports, autopsy reports) in order to perform an independent medical assessment of the reported case.

5.6 ADVERSE EVENTS THAT OCCUR AFTER THE ADVERSE EVENT REPORTING PERIOD

After the end of the adverse event reporting period (defined as a specific number of days after the last dose of study drug depending on the treatment; see Section 5.3.1), all deaths, regardless of cause, should be reported through use of the Long-Term Survival Follow-Up eCRF.

In addition, if the investigator becomes aware of a serious adverse event that is believed to be related to prior exposure to study drug, the event should be reported through use of the Adverse Event eCRF. However, if the EDC system is not available, the investigator should report these events directly to the Sponsor or its designee, either by faxing or by scanning and emailing the paper Clinical Trial Serious Adverse Event/Adverse Event of Special Interest Reporting Form using the fax number or email address provided to investigators.

5.7 EXPEDITED REPORTING TO HEALTH AUTHORITIES, INVESTIGATORS, INSTITUTIONAL REVIEW BOARDS, AND ETHICS COMMITTEES

The Sponsor will promptly evaluate all serious adverse events and adverse events of special interest against cumulative product experience to identify and expeditiously communicate possible new safety findings to investigators, IRBs, ECs, and applicable health authorities based on applicable legislation.

To determine reporting requirements for single adverse event cases, the Sponsor will assess the expectedness of these events using the following reference documents:

- Investigator's Brochures for the following study drugs:
  - Alectinib
  - Atezolizumab
  - Entrectinib
  - Vemurafenib
  - Cobimetinib
  - *Bevacizumab*
- E.U. Summary of Product Characteristics for the following study drugs:
  - Cisplatin

"Use of this document is governed by the terms of use on the first page of this document."

- Carboplatin
- Pemetrexed
- Gemcitabine

The Sponsor will compare the severity of each event and the cumulative event frequency reported for the study with the severity and frequency reported in the applicable reference document.

Reporting requirements will also be based on the investigator's assessment of causality and seriousness, with allowance for upgrading by the Sponsor as needed.

6. STATISTICAL CONSIDERATIONS AND ANALYSIS PLAN

6.1 ANALYSIS POPULATIONS

Analysis populations are defined as follows:

- The intent-to-treat (ITT) population is defined as all randomized patients (for randomized cohorts) or enrolled patients (for non-randomized cohorts), whether or not the assigned study treatment was received.
- The all-treated population is defined as ITT patients who received at least one dose of study drug. The all-treated population will serve as the safety-evaluable population.
- The duration of response (DOR)–evaluable population is defined as patients with an objective response.

The analysis populations to be used for statistical analysis are specific to the treatment cohorts. See cohort-specific appendices for details.

6.2 DETERMINATION OF SAMPLE SIZE

Sample sizes are specific to the study cohorts; please refer to the following appendices for details:

- Cohort A (ALK+): Appendix 7, Section 11.6.1
- Cohort B (RET+): Appendix 8, Section 12.6.1
- Cohort C (bTMB+): Appendix 9, Section 13.6.1
- Cohort D (ROS1+): Appendix 10, Section 14.6.1
- Cohort E (BRAF V600+): Appendix 11, Section 15.6.1
- *Cohort F (EGFR exon 20+): Appendix 12, Section* 16.6.1

6.3 SUMMARIES OF CONDUCT OF STUDY

Enrollment, study drug administration, and discontinuation from the study will be summarized by cohort and, if applicable, by treatment arm. The incidence of study drug discontinuation for reasons other than disease progression will be tabulated similarly.

"Use of this document is governed by the terms of use on the first page of this document."

Protocol deviations, including major deviations of inclusion/exclusion criteria, will be also be summarized in a similar manner by cohort and, if applicable, treatment arm.

6.4 SUMMARIES OF TREATMENT GROUP COMPARABILITY AND/OR DEMOGRAPHIC AND BASELINE CHARACTERISTICS

For all patients and cohorts, demographic and baseline characteristics (including age, sex, race/ethnicity, baseline disease characteristics, ECOG Performance Status, and number of prior cancer treatments) will be summarized using means, standard deviations, medians, and ranges for continuous variables and proportions for categorical variables, as appropriate. Summaries will be presented by cohort and, if applicable, treatment arm.

6.5 EFFICACY ANALYSES

The endpoints selected for statistical analysis (and whether they are deemed primary or secondary) are specific to the treatment cohorts; please refer to the respective appendices for details as provided in the following appendices:

- Cohort A (ALK+): Appendix 7, Section 11.6.2
- Cohort B (RET+): Appendix 8, Section 12.6.2
- Cohort C (bTMB+): Appendix 9, Section 13.6.2
- Cohort D (ROS1+): Appendix 10, Section 14.6.2
- Cohort E (BRAF V600+*):* Appendix 11, Section 15.6.2
- *Cohort F (EGFR exon 20+): Appendix 12, Section* 16.6.2

6.6 PATIENT-REPORTED OUTCOMES ANALYSES

The EORTC QLQ-C30 and items from the EORTC QLQ-BN20 will be scored according to the EORTC scoring manual, 3rd edition (Fayers et al. 2001), and the EORTC QLQ-BN20 summary sheet (Taphoorn et al. 2010). The EORTC QLQ-C30 is composed of both multi-item scales and single-item measures including functioning scales, symptom scales, and a global health status/health-related quality of life (HRQoL) scale. For multi-item subscales, if $\leq$50% of items within the multi-item subscale are missing at a given timepoint, the multi-item score will be calculated on the basis of the non-missing items. If $\geq$50% of items are missing or if a single-item measure is missing, the subscale is considered missing.

All of the scales and single-item measures will be linearly transformed so that each score will range from 0 to 100. A high score for a functioning scale represents a high/healthy level of functioning, and a high score for the global health status/HRQoL represents a high level of HRQoL; however, a high score for a symptom scale/item represents a high level of symptomatology.

The SILC questionnaire comprises three individual symptoms (dyspnea, cough, and chest pain) and will be scored at the individual symptom level; thus, there will be a

"Use of this document is governed by the terms of use on the first page of this document."

dyspnea score, chest pain score, and cough score. Each individual symptom score will be calculated as the average of responses for the symptom items (e.g., chest pain score = mean [item 1; item 2]). An increase in score is suggestive of a worsening in symptomology (i.e., frequency or severity). A score change of $\geq$0.3 points for the dyspnea and cough symptom scores is considered to be clinically significant, while a score change of $\geq$0.5 points for the chest pain score is considered to be clinically significant.

The EQ-5D-5L will generate utility scores for use in economic models for reimbursement purposes only.

Please refer to the respective appendices for cohort-specific PRO analysis plans.

6.7 SAFETY ANALYSES

All safety analyses will be performed by cohort and, if applicable, treatment arm.

Safety analyses will be performed on the safety-evaluable population (see Section 6.1) and will include all patients who received at least one dose of study drug.

Study drug exposure, including treatment duration, number of doses, and dose intensity, will be summarized using descriptive statistics.

Verbatim description of adverse events will be mapped to thesaurus terms and graded according to NCI CTCAE v4.0. All adverse events occurring during or after the first study drug dose will be summarized by NCI CTCAE grade. In addition, serious adverse events; severe adverse events (Grade 3, 4, and 5); selected adverse events; and adverse events leading to study drug dose reduction, discontinuation, or interruption will be summarized accordingly. Multiple occurrences of the same event will be counted once at the maximum severity. The proportion of patients experiencing at least one adverse event will be reported by preferred term.

Deaths reported during the study treatment period and those reported during the follow-up period after treatment completion/discontinuation will be summarized.

Laboratory values will be summarized, including summary tables for the shifts in grades from baseline to the worst grade observed during treatment. In addition, ECG data and changes in vital signs will be summarized.

Additional details on safety analyses will be provided in cohort-specific Statistical Analysis Plans (SAPs), if deemed necessary.

6.8 PHARMACOKINETIC ANALYSES

Specific PK analyses are currently planned for Cohort B (RET+), Cohort D (ROS1+), Cohort E (BRAF V600+)*, and Cohort F (EGFR exon 20*+). See Appendix 8,

"Use of this document is governed by the terms of use on the first page of this document."

Section 12.6.3, Appendix 10, Section 14.6.3, Appendix 11, Section 15.6.3*, and Appendix 12, Section* 16.6.3 for additional details.

6.9 IMMUNOGENICITY ANALYSES

Specific immunogenicity analyses are currently planned for Cohort E (BRAF V600+*; see* Appendix 11, Section 15.6.4 for additional details*) and Cohort F (EGFR exon 20+; see Appendix 12, Section* 16.6.4 *for additional details).*

6.10 BIOMARKER ANALYSES

Exploratory biomarker analyses will be performed in an effort to understand the association of biomarkers with study drug response, including but not limited to efficacy and/or adverse events, as well as emergence of resistance. Additional pharmacodynamic analyses will be conducted as appropriate. Exploratory biomarker analyses will include assessments of biomarkers in both tumor tissue (when available) and blood. Such assessments will include, but will not be limited to, mutational analysis using NGS, gene expression, and proteomics and may also include other methods that may become available in the future.

6.11 NATURAL HISTORY FOLLOW-UP ANALYSES

This study will also characterize the natural history of patients with genetic alteration profiles of interest who do not enroll in any of the treatment cohorts. Details regarding cancer therapies (or therapy types), treatment response, and survival status may be analyzed. Analyses will be exploratory in nature; there is no formal hypothesis testing in this cohort.

6.12 INTERIM ANALYSES

Interim analyses (planned and/or optional) may be performed during the overall conduct of the study. The nature and planning for interim analyses will be determined at the cohort level, as each cohort has a unique trial design and correspondingly a unique set of efficacy and safety objectives. The statistical considerations also are determined at the individual cohort level. Please refer to the cohort-specific appendices for details regarding interim analyses.

Interim analyses may be conducted by an Internal Monitoring Committee (IMC) or independent Data Monitoring Committee (iDMC), depending on the type of committee deemed necessary for a given cohort. Each cohort's statistical section will clearly state whether an IMC or iDMC will be used (see cohort-specific appendices for this information). Additionally, the roles, responsibilities, and decision-making processes will be detailed in separate IMC or iDMC charters.

"Use of this document is governed by the terms of use on the first page of this document."

7. DATA COLLECTION AND MANAGEMENT

7.1 DATA QUALITY ASSURANCE

The Sponsor will be responsible for data management of this study, including quality checking of the data. Data entered manually will be collected via EDC through use of eCRFs. Sites will be responsible for data entry into the EDC system. In the event of discrepant data, the Sponsor will request data clarification from the sites, which the sites will resolve electronically in the EDC system.

The Sponsor will produce an EDC Study Specification document that describes the quality checking to be performed on the data. Central laboratory data and other electronic data will be sent directly to the Sponsor, using the Sponsor's standard procedures to handle and process the electronic transfer of these data.

eCRFs and correction documentation will be maintained in the EDC system's audit trail. System backups for data stored by the Sponsor and records retention for the study data will be consistent with the Sponsor's standard procedures.

PRO data will be collected through the use of an electronic device provided by a vendor (see Section 7.3 for details).

7.2 ELECTRONIC CASE REPORT FORMS

eCRFs are to be completed through use of a Sponsor-designated EDC system. Sites will receive training and have access to a manual for appropriate eCRF completion. eCRFs will be submitted electronically to the Sponsor and should be handled in accordance with instructions from the Sponsor.

All eCRFs should be completed by designated, trained site staff. eCRFs should be reviewed and electronically signed and dated by the investigator or a designee.

At the end of the study, the investigator will receive patient data for his or her site in a readable format on a compact disc that must be kept with the study records. Acknowledgement of receipt of the compact disc is required.

7.3 ELECTRONIC PATIENT-REPORTED OUTCOME DATA (IF APPLICABLE)

If patients will be using an electronic device to capture PRO data, it will be designed for entry of data in a way that is attributable, secure, and accurate, in compliance with FDA regulations for electronic records (21 CFR Part 11). The data will be transmitted to a centralized database maintained by the electronic device vendor.

The electronic data will be available for view access only, via secure access to a Web portal provided by the ePRO vendor. Only identified and trained users may view the data, and their actions will become part of the audit trail. The Sponsor will have view

"Use of this document is governed by the terms of use on the first page of this document."

access only. System backups for data stored by the Sponsor and records retention for the study data will be consistent with the Sponsor's standard procedures.

Once the study is complete, the data, audit trail, and trial and system documentation will be archived. The investigator will receive patient data for the site in both human- and machine-readable formats on an archival-quality compact disc that must be kept with the study records as source data. Acknowledgement of receipt of the compact disc is required. In addition, the Sponsor will receive all data in a machine-readable format on a compact disc.

7.4 SOURCE DATA DOCUMENTATION

Study monitors will perform ongoing source data verification and review to confirm that critical protocol data (i.e., source data) entered into the eCRFs by authorized site personnel are accurate, complete, and verifiable from source documents.

Source documents (paper or electronic) are those in which patient data are recorded and documented for the first time. They include, but are not limited to, hospital records, clinical and office charts, laboratory notes, memoranda, patient-reported outcomes, evaluation checklists, pharmacy dispensing records, recorded data from automated instruments, copies of transcriptions that are certified after verification as being accurate and complete, microfiche, photographic negatives, microfilm or magnetic media, X-rays, patient files, and records kept at pharmacies, laboratories, and medico-technical departments involved in a clinical trial.

Before study initiation, the types of source documents that are to be generated will be clearly defined in the Trial Monitoring Plan. This includes any protocol data to be entered directly into the eCRFs (i.e., no prior written or electronic record of the data) and considered source data.

Source documents that are required to verify the validity and completeness of data entered into the eCRFs must not be obliterated or destroyed and must be retained per the policy for retention of records described in Section 7.6.

To facilitate source data verification and review, the investigators and institutions must provide the Sponsor direct access to applicable source documents and reports for trial-related monitoring, Sponsor audits, and IRB/EC review. The study site must also allow inspection by applicable health authorities.

7.5 USE OF COMPUTERIZED SYSTEMS

When clinical observations are entered directly into a study site's computerized medical record system (i.e., in lieu of original hardcopy records), the electronic record can serve as the source document if the system has been validated in accordance with health authority requirements pertaining to computerized systems used in clinical research. An acceptable computerized data collection system allows preservation of the original

"Use of this document is governed by the terms of use on the first page of this document."

entry of data. If original data are modified, the system should maintain a viewable audit trail that shows the original data as well as the reason for the change, name of the person making the change, and date of the change.

7.6 RETENTION OF RECORDS

Records and documents pertaining to the conduct of this study and the distribution of IMP, including eCRFs, electronic or paper PRO data (whichever is applicable), Informed Consent Forms, laboratory test results, and medication inventory records, must be retained by the Principal Investigator for 15 years after completion or discontinuation of the study or for the length of time required by relevant national or local health authorities, whichever is longer. After that period of time, the documents may be destroyed, subject to local regulations.

No records may be disposed of without the written approval of the Sponsor. Written notification should be provided to the Sponsor prior to transferring any records to another party or moving them to another location.

Roche will retain study data for 25 years after the final Clinical Study Report has been completed or for the length of time required by relevant national or local health authorities, whichever is longer.

8. ETHICAL CONSIDERATIONS

8.1 COMPLIANCE WITH LAWS AND REGULATIONS

This study will be conducted in full conformance with the ICH E6 guideline for Good Clinical Practice and the principles of the Declaration of Helsinki, or the applicable laws and regulations of the country in which the research is conducted, whichever affords the greater protection to the individual. The study will comply with the requirements of the ICH E2A guideline (Clinical Safety Data Management: Definitions and Standards for Expedited Reporting). Studies conducted in the United States or under a U.S. Investigational New Drug (IND) application will comply with U.S. FDA regulations and applicable local, state, and federal laws. Studies conducted in the European Union or European Economic Area will comply with the E.U. Clinical Trial Directive (2001/20/EC).

8.2 INFORMED CONSENT

The Sponsor’s sample Informed Consent Forms will be provided to each site. If applicable, it will be provided in a certified translation of the local language. The Sponsor or its designee must review and approve any proposed deviations from the Sponsor's sample Informed Consent Forms or any alternate consent forms proposed by the site (collectively, the "Consent Forms") before IRB/EC submission. The final IRB/EC–approved Consent Forms must be provided to the Sponsor for health authority submission purposes according to local requirements.

"Use of this document is governed by the terms of use on the first page of this document."

If applicable, the Informed Consent Form will contain separate sections for any optional procedures. The investigator or authorized designee will explain to each patient the objectives, methods, and potential risks associated with each optional procedure. Patients will be told that they are free to refuse to participate and may withdraw their consent at any time for any reason. A separate, specific signature will be required to document a patient's agreement to participate in optional procedures. Patients who decline to participate will not provide a separate signature.

The Consent Forms must be signed and dated by the patient or the patient's legally authorized representative before his or her participation in the study. The case history or clinical records for each patient shall document the informed consent process and that written informed consent was obtained prior to participation in the study.

The Consent Forms should be revised whenever there are changes to study procedures or when new information becomes available that may affect the willingness of the patient to participate. The final revised IRB/EC–approved Consent Forms must be provided to the Sponsor for health authority submission purposes.

Patients must be re-consented to the most current version of the Consent Forms (or to a significant new information/findings addendum in accordance with applicable laws and IRB/EC policy) during their participation in the study. For any updated or revised Consent Forms, the case history or clinical records for each patient shall document the informed consent process and that written informed consent was obtained using the updated/revised Consent Forms for continued participation in the study.

A copy of each signed Consent Form must be provided to the patient or the patient's legally authorized representative. All signed and dated Consent Forms must remain in each patient's study file or in the site file and must be available for verification by study monitors at any time.

For sites in the United States, each Consent Form may also include patient authorization to allow use and disclosure of personal health information in compliance with the U.S. Health Insurance Portability and Accountability Act (HIPAA) of 1996. If the site utilizes a separate Authorization Form for patient authorization for use and disclosure of personal health information under the HIPAA regulations, the review, approval, and other processes outlined above apply except that IRB review and approval may not be required per study site policies.

8.3 INSTITUTIONAL REVIEW BOARD OR ETHICS COMMITTEE

This protocol, the Informed Consent Forms, any information to be given to the patient, and relevant supporting information must be submitted to the IRB/EC by the Principal Investigator and reviewed and approved by the IRB/EC before the study is initiated. In addition, any patient recruitment materials must be approved by the IRB/EC.

"Use of this document is governed by the terms of use on the first page of this document."

The Principal Investigator is responsible for providing written summaries of the status of the study to the IRB/EC annually or more frequently in accordance with the requirements, policies, and procedures established by the IRB/EC. Investigators are also responsible for promptly informing the IRB/EC of any protocol amendments (see Section 9.7).

In addition to the requirements for reporting all adverse events to the Sponsor, investigators must comply with requirements for reporting serious adverse events to the local health authority and IRB/EC. Investigators may receive written IND safety reports or other safety-related communications from the Sponsor. Investigators are responsible for ensuring that such reports are reviewed and processed in accordance with health authority requirements and the policies and procedures established by their IRB/EC, and archived in the site's study file.

8.4 CONFIDENTIALITY

The Sponsor maintains confidentiality standards by coding each patient enrolled in the study through assignment of a unique patient identification number. This means that patient names are not included in data sets that are transmitted to any Sponsor location.

Patient medical information obtained by this study is confidential and may be disclosed to third parties only as permitted by the Informed Consent Form (or separate authorization for use and disclosure of personal health information) signed by the patient, unless permitted or required by law.

Medical information may be given to a patient's personal physician or other appropriate medical personnel responsible for the patient's welfare, for treatment purposes.

Given the complexity and exploratory nature of the analyses, data derived from exploratory biomarker specimens will generally not be provided to study investigators or patients unless required by law. The aggregate results of any conducted research will be available in accordance with the effective Roche policy on study data publication (see Section 9.6).

Data generated by this study must be available for inspection upon request by representatives of national and local health authorities, Sponsor monitors, representatives, and collaborators, and the IRB/EC for each study site, as appropriate.

Study data may be submitted to government or other health research databases or shared with researchers, government agencies, companies, or other groups that are not participating in this study. These data may be combined with or linked to other data and used for research purposes, to advance science and public health, or for analysis, development, and commercialization of products to treat and diagnose disease. In addition, redacted clinical study reports and other summary reports will be provided upon request.

"Use of this document is governed by the terms of use on the first page of this document."

8.5 FINANCIAL DISCLOSURE

Investigators will provide the Sponsor with sufficient, accurate financial information in accordance with local regulations to allow the Sponsor to submit complete and accurate financial certification or disclosure statements to the appropriate health authorities. Investigators are responsible for providing information on financial interests during the course of the study and for 1 year after completion of the study (i.e., LPLV).

9. STUDY DOCUMENTATION, MONITORING, AND ADMINISTRATION

9.1 STUDY DOCUMENTATION

The investigator must maintain adequate and accurate records to enable the conduct of the study to be fully documented, including, but not limited to, the protocol, protocol amendments, Informed Consent Forms, and documentation of IRB/EC and governmental approval. In addition, at the end of the study, the investigator will receive the patient data, including an audit trail containing a complete record of all changes to data.

9.2 PROTOCOL DEVIATIONS

The investigator should document and explain any protocol deviations. The investigator should promptly report any deviations that might have an impact on patient safety and data integrity to the Sponsor and to the IRB/EC in accordance with established IRB/EC policies and procedures. The Sponsor will review all protocol deviations and assess whether any represent a serious breach of Good Clinical Practice guidelines and require reporting to health authorities. As per the Sponsor's standard operating procedures, prospective requests to deviate from the protocol, including requests to waive protocol eligibility criteria, are not allowed.

9.3 MANAGEMENT OF STUDY QUALITY

The Sponsor will implement a system to manage the quality of the study, focusing on processes and data that are essential to ensuring patient safety and data integrity. The Sponsor will identify potential risks associated with critical trial processes and data and will implement plans for evaluating and controlling these risks. Risk evaluation and control will include the selection of risk-based parameters (e.g., adverse event rate, protocol deviation rate) and the establishment of quality tolerance limits for these parameters prior to study initiation. Detection of deviations from quality tolerance limits will trigger an evaluation to determine if action is needed. Details on the establishment and monitoring of quality tolerance limits will be provided in a Quality Tolerance Limit Management Plan.

9.4 SITE INSPECTIONS

Site visits will be conducted by the Sponsor or an authorized representative for inspection of study data, patients' medical records, and eCRFs. The investigator will

"Use of this document is governed by the terms of use on the first page of this document."

permit national and local health authorities; Sponsor monitors, representatives, and collaborators; and the IRBs/ECs to inspect facilities and records relevant to this study.

9.5 ADMINISTRATIVE STRUCTURE

This trial will be sponsored and managed by the Sponsor.

A Steering Committee is established to provide the study Sponsor with recommendations related to any aspect of the trial, specifically study design, data interpretation, exploratory analyses, or alternate changes to the trial that may assist in patient accrual, data collection, analysis, and interpretation of the study results. The Sponsor is ultimately responsible for all decisions regarding the study.

Depending on the cohort, an iDMC or IMC may be established to monitor the progress of the study and ensure that the safety of patients enrolled in the study is not compromised. Some cohorts may not require either an IMC or iDMC. This is necessary because of the umbrella nature of the study, where each individual cohort has a unique design and set of objectives from an efficacy, safety, and statistical perspective. Details of the composition, roles, and responsibilities, and processes of the iDMC and/or IMC will be documented in separate iDMC and IMC charters. The iDMC or IMC will review safety data and can make recommendations to the Sponsor to stop or amend the study on the basis of safety findings. The frequency of these reviews as well as the data to be reviewed will be agreed with the iDMC or IMC and outlined in the separate iDMC or IMC charters. All iDMC review meetings will be held in a blinded manner to the Sponsor. For further details, refer to the cohort-specific appendices.

Central facilities will be used for certain study assessments throughout the study (e.g., specified laboratory tests, and PK analyses). Accredited local laboratories will be used for routine monitoring; local laboratory ranges will be collected.

Scans will be submitted to an IRF for central review.

9.6 DISSEMINATION OF DATA AND PROTECTION OF TRADE SECRETS

Regardless of the outcome of a trial, the Sponsor is dedicated to openly providing information on the trial to healthcare professionals and to the public, at scientific congresses, in clinical trial registries, and in peer-reviewed journals. The Sponsor will comply with all requirements for publication of study results. Study data may be shared with others who are not participating in this study (see Section 8.4 for details), and redacted Clinical Study Reports and other summary reports will be made available upon request, provided the requirements of Roche's global policy on data sharing have been met. For more information, refer to the Roche Global Policy on Sharing of Clinical Study Information at the following Web site:

http://www.roche.com/roche_global_policy_on_sharing_of_clinical_study_information.pdf

"Use of this document is governed by the terms of use on the first page of this document."

The results of this study may be published or presented at scientific congresses. For all clinical trials in patients involving an IMP for which a marketing authorization application has been filed or approved in any country, the Sponsor aims to submit a journal manuscript reporting primary clinical trial results within 6 months after the availability of the respective Clinical Study Report. In addition, for all clinical trials in patients involving an IMP for which a marketing authorization application has been filed or approved in any country, the Sponsor aims to publish results from analyses of additional endpoints and exploratory data that are clinically meaningful and statistically sound.

The investigator must agree to submit all manuscripts or abstracts to the Sponsor prior to submission for publication or presentation. This allows the Sponsor to protect proprietary information and to provide comments based on information from other studies that may not yet be available to the investigator.

In accordance with standard editorial and ethical practice, the Sponsor will generally support publication of multicenter trials only in their entirety and not as individual center data. In this case, a coordinating investigator will be designated by mutual agreement.

Authorship will be determined by mutual agreement and in line with International Committee of Medical Journal Editors authorship requirements. Any formal publication of the study in which contribution of Sponsor personnel exceeded that of conventional monitoring will be considered as a joint publication by the investigator and the appropriate Sponsor personnel.

Any inventions and resulting patents, improvements, and/or know-how originating from the use of data from this study will become and remain the exclusive and unburdened property of the Sponsor, except where agreed otherwise.

9.7 PROTOCOL AMENDMENTS

Any protocol amendments will be prepared by the Sponsor. Protocol amendments will be submitted to the IRB/EC and to regulatory authorities in accordance with local regulatory requirements.

Approval must be obtained from the IRB/EC and regulatory authorities (as locally required) before implementation of any changes, except for changes necessary to eliminate an immediate hazard to patients or changes that involve logistical or administrative aspects only (e.g., change in Medical Monitor or contact information).

"Use of this document is governed by the terms of use on the first page of this document."

10. REFERENCES

Aaronson NK, Ahmedzai S, Bergman B, et al. The European Organization for Research and Treatment of Cancer QLQ-C30: a quality-of-life instrument for use in international clinical trials in oncology. J Natl Cancer Inst 1993;85:365–76.

Alexandrov LB, Nik-Zainal S, Wedge DC, et al. Signatures of mutational processes in human cancer. Nature 2013;500:415–21.

Alimta® (pemetrexed) U.S. prescribing information. Indianapolis (IN): Lilly USA, 2013.

Barlesi F, Mazieres J, Merlio J-P, et al. Routine molecular profiling of patients with advanced non–small cell lung cancer: results of a 1-year nationwide programme of the French Cooperative Thoracic Intergroup (IFCT). Lancet 2016;387:1415–26.

Bergman B, Aaronson NK, Ahmedzai S, et al. The EORTC QLQ-LC13: a modular supplement to the EORTC Core Quality of Life Questionnaire (QLQ-C30) for use in lung cancer clinical trials. EORTC Study Group on Quality of Life. Eur J Cancer 1994;30A:635–42.

Bezjak A, Tu D, Seymour L, et al. Symptom improvement in lung cancer patients treated with erlotinib: quality of life analysis of the National Cancer Institute of Canada Clinical Trials Group Study BR21. J Clin Oncol 2006;24:3831–7.

Brown SD, Warren RL, Gibb EA, et al. Neo-antigens predicted by tumor genome meta-analysis correlate with increased patient survival. Genome Res 2014;24:743–50.

Chen DS, Irving BA, Hodi FS. Molecular pathways: next-generation immunotherapy—inhibiting programmed death-ligand 1 and programmed death-1. Clin Cancer Res 2012;18:6580–7.

Edwards BK, Noone AM, Mariotto AB, et al. Annual report to the nation on the status of cancer, 1975−2010, featuring prevalence of comorbidity and impact on survival among persons with lung, colorectal, breast, or prostate cancer. Cancer 2014;120:1290–314.

Eisenhauer EA, Therasse P, Bogaerts J, et al. New response evaluation criteria in solid tumors: Revised RECIST guideline (Version 1.1). Eur J Cancer 2009;45:228–47.

Fayers PM, Aaronson NK, Bjordal K, et al. The EORTC QLQ-C30 scoring manual (3rd edition). Brussels: European Organisation for Research and Treatment of Cancer, 2001.

[FDA] U.S. Department of Health and Human Services Food and Drug Administration. Guidance for industry: patient-reported outcome measures: use in medical product development to support labeling claims. December 2009. https://www.fda.gov/downloads/drugs/guidances/ucm193282.pdf. Accessed 26 February 2017.

"Use of this document is governed by the terms of use on the first page of this document."

Ferlay J, Soerjomataram I, Dikshit R, et al. Cancer incidence and mortality worldwide: sources, methods and major patterns in GLOBOCAN 2012. Int J Cancer 2015;136:E359–86.

Fitzsimmons D, Johnson CD, George S, et al. Development of a disease specific quality of life (QoL) questionnaire module to supplement the EORTC core cancer QoL questionnaire, the QLQ-C30 in patients with pancreatic cancer. Eur J Cancer 1999;35:939–41.

Hopwood P, Stephens RJ. Symptoms at presentation for treatment in patients with lung cancer: implications for the evaluation of palliative treatment. The Medical Research Council (MRC) Lung Cancer Working Party. Br J Cancer 1995;71:633–6.

Howlader N, Noone AM, Krapcho M, et al. SEER Cancer Statistics Review, 1975–2013, National Cancer Institute. Bethesda, MD, http://seer.cancer.gov/csr/1975_2013/, based on November 2015 SEER data submission, posted to the SEER Web site, April 2016.

Hyde L, Hyde CI. Clinical manifestations of lung cancer. Chest 1974;65:299–306.

Kris MG, Johnson BE, Berry LD, et al. Using multiplexed assays of oncogenic drivers in lung cancers to select targeted drugs. JAMA 2014;311:1998–2006.

Lawrence MS, Stojanov P, Polak P, et al. Mutational heterogeneity in cancer and the search for new cancer-associated genes. Nature 2013;499:214–8.

Le DT, Uram JN, Wang H, et al. PD-1 blockade in tumors with mismatch-repair deficiency. N Eng J Med 2015;372:2509–20.

Miller KD, Siegel RL, Lin CC, et al. Cancer treatment and survivorship statistics, 2016. CA Cancer J Clin 2016;66:271–89.

[NCCN] National Comprehensive Cancer Network. NCCN clinical practice guidelines in oncology. Non-small cell lung cancer v4.2016. http://www.nccn.org/professionals/physician_gls/pdf/nscl.pdf. Accessed: 14 March 2016.

Rivera MP, Mehta AC, Wahidi MM. Establishing the diagnosis of lung cancer: diagnosis and management of lung cancer: American College of Chest Physicians evidence-based clinical practice guidelines. Chest 2013;143(5 Suppl):e142S–65S.

Rizvi NA, Hellmann MD, Snyder A, et al. Mutational landscape determines sensitivity to PD-1 blockade in non–small cell lung cancer. Science 2015;348:124–8.

Rosell R, Carcereny E, Gervais R, et al. Erlotinib versus standard chemotherapy as first-line treatment for European patients with advanced EGFR mutation-positive non-small-cell lung cancer (EURTAC): a multicentre, open-label, randomised phase 3 trial. Lancet Oncol 2012;13:239–46.

"Use of this document is governed by the terms of use on the first page of this document."

Rosenberg JE, Hoffman-Censits J, Powles T, et al. Atezolizumab in patients with locally advanced and metastatic urothelial carcinoma who have progressed following treatment with platinum-based chemotherapy: a single-arm, multicentre, phase 2 trial. Lancet 2016;387:1909–20.

Sandler A, Gray R, Perry MC, et al. Paclitaxel–carboplatin alone or with bevacizumab for non–small-cell lung cancer. N Engl J Med 2006;355:2542–50.

Sarna L, Evangelista L, Tashkin D, et al. Impact of respiratory symptoms and pulmonary function on quality of life of long-term survivors of non-small cell lung cancer. Chest 2004;125:439–45.

Scagliotti GV, Parikh P, Von Pawel J, et al. Phase III study comparing cisplatin plus gemcitabine with cisplatin plus pemetrexed in chemotherapy-naive patients with advanced-stage non–small-cell lung cancer. J Clin Oncol 2008;26:3543–51.

Schiller JH, Harrington D, Belani CP, et al. Eastern Cooperative Oncology Group. Comparison of four chemotherapy regimens for advanced non–small cell lung cancer. N Engl J Med 2002;346:92–8.

Schumacher TN, Schreiber RD. Neoantigens in cancer immunotherapy. Science 2015; 348:69–74.

Snyder A, Makarov V, Merghoub T, et al. Genetic basis for clinical response to CTLA-4 blockade in melanoma. N Eng J Med 2014;371:2189–99.

Solomon BJ, Mok T, Kim DW, et al. First-line crizotinib versus chemotherapy in ALK-positive lung cancer. N Eng J Med 2014;371:2167–77.

Tam AL, Kim ES, Lee JJ, et al. Feasibility of image-guided transthoracic core-needle biopsy in the BATTLE lung trial. J Thorac Oncol 2013;8:436–42.

Taphoorn MJ, Claasens L, Aaronson NK, et al. An international validation study of the EORTC brain cancer module (EORTC QLQ-BN20) for assessing health-related quality of life and symptoms in brain-cancer patients. Eur J Cancer 2010;46:1033–40.

Tarceva® (erlotinib) U.S. prescribing information. South San Francisco (CA): Genentech, 2016

Taxotere® (docetaxel) U.S. prescribing information. Paris: Sanofi ; 2015.

Tsui DW, Berger MF. Profiling non–small cell lung cancer: from tumor to blood. Clin Cancer Res 2016;22:790–2.

Vogelstein B, Papadopoulos N, Velculescu VE, et al. Cancer genome landscapes. Science 2013;339:1546–58.

Xalkori® (crizotinib) U.S. prescribing information. New York: Pfizer Inc., 2017.

"Use of this document is governed by the terms of use on the first page of this document."

Appendix 1
Schedule of Activities: Blood-Based NGS ctDNA Assay Screening

Note: These activities are to be completed and results known prior to obtaining and submitting a sample for the blood-based NGS ctDNA assay screening. Activities regarding the treatment-specific cohorts are provided in the respective appendices.

	Screening
Blood-based screening Informed consent	x
Demographic data [a]	x
Complete blood count with differential (WBC, hemoglobin, platelets, neutrophils, lymphocytes) [b]	x
Liver function tests (ALT, AST, total bilirubin) [b]	x
Serum creatinine [b]	x
History of NSCLC and related treatment	x
ECOG Performance Status	x
Blood-based NGS ctDNA sample submitted to central laboratory [c]	x

ctDNA = circulating tumor DNA; ECOG = Eastern Cooperative Oncology Group; FMI = Foundation Medicine, Inc.; ICF = informed consent form; NGS = next-generation sequencing; NSCLC = non–small cell lung cancer.

[a] Demographic data will include: age, sex, and self-reported race/ethnicity, information regarding tissue availability and biomarker testing will be collected for all patients during the screening period. Documentation must be obtained and data will be collected as to whether tissue is available for testing or if a tissue test for the biomarkers of interest (e.g., corresponding to open cohorts in the BFAST trial, PD-L1 test results, etc.) has been performed. If a tissue test report is available, the result of the test, date of collection, and assay methodology (if clearly defined) will be captured.

[b] These laboratory assessments could have been collected *previously according to the standard of care* if they are deemed clinically relevant by the treating physician.

[c] Turnaround time for the blood-based NGS ctDNA assays is 10–14 calendar days upon receipt of the blood samples at FMI.

"Use of this document is governed by the terms of use on the first page of this document."

Appendix 2
Contraception and Pregnancy Reporting Requirements

APPLICABLE TO ALL STUDY TREATMENTS/COHORTS

Definition of Woman of Childbearing Potential

A woman is considered to be of childbearing potential if she is postmenarcheal, has not reached a postmenopausal state ($\geq$12 continuous months of amenorrhea with no identified cause other than menopause), and has not undergone surgical sterilization (removal of ovaries and/or uterus). The definition of childbearing potential may be adapted for alignment with local guidelines or requirements.

Contraception Requirements during the Treatment Period for Women of Childbearing Potential

Female patients of childbearing potential must agree to remain abstinent (refrain from heterosexual intercourse) or use contraceptive methods with a failure rate of $<$1% per year during the treatment period. Women must refrain from donating eggs during this same period.

Contraceptive Methods

Examples of highly effective contraceptive methods with a failure rate of $<$1% per year include bilateral tubal ligation, male sterilization, hormonal contraceptives that inhibit ovulation, hormone-releasing intrauterine devices, and copper intrauterine devices.

The reliability of sexual abstinence should be evaluated in relation to the duration of the clinical trial and the preferred and usual lifestyle of the patient. Periodic abstinence (e.g., calendar, ovulation, symptothermal, or postovulation methods) and withdrawal are not acceptable methods of contraception.

APPLICABLE TO ALECTINIB COHORTS (COHORTS A AND B)

Contraception Requirements after the Last Dose of Study Treatment for Women of Childbearing Potential

Female patients of childbearing potential must agree to remain abstinent or use contraceptive methods (see above) for at least 3 months after the last dose of study drug. Women must refrain from donating eggs during this same period.

Contraception Requirements for Men

With female partners of childbearing potential or pregnant female partners, male patients must remain abstinent or use a condom during the treatment period and for at least 3 months after the last dose of study drug to avoid exposing the embryo. Male patients must refrain from donating sperm during this same period.

The reliability of sexual abstinence should be evaluated in relation to the duration of the clinical trial and the preferred and usual lifestyle of the patient. Periodic abstinence (e.g., calendar, ovulation, symptothermal, or postovulation methods) and withdrawal are not acceptable methods of contraception.

"Use of this document is governed by the terms of use on the first page of this document."

Pregnancy Reporting Requirements

Female patients of childbearing potential will be instructed to immediately inform the investigator if they become pregnant during the study or within 3 months after the last dose of study drug.

Male patients will be instructed through the Informed Consent Form to immediately inform the investigator if their partner becomes pregnant during study treatment with chemotherapy or within 3 months after the last dose of study drug.

APPLICABLE TO ATEZOLIZUMAB VERSUS CHEMOTHERAPY COHORT (COHORT C)

Contraception Requirements after the Last Dose of Study Treatment for Women of Childbearing Potential

Female patients of childbearing potential (see above) should continue contraception use for 5 months after the last dose of atezolizumab and for 6 months after the last dose of cisplatin. Women must refrain from donating eggs during this same period.

Contraception Requirements for Men

During the treatment period, male patients must remain abstinent or use a condom. Male patients treated with chemotherapy (cisplatin or carboplatin plus pemetrexed or gemcitabine) should continue contraception use for 6 months after the last dose of chemotherapy to avoid exposing the embryo. Male patients must refrain from donating sperm during this same period.

Pregnancy Reporting Requirements

Female patients of childbearing potential will be instructed to immediately inform the investigator if they become pregnant during the study or within 5 months after the last dose of atezolizumab or within 6 months after the last dose of cisplatin.

Male patients will be instructed through the Informed Consent Form to immediately inform the investigator if their partner becomes pregnant during study treatment with chemotherapy or within 6 months after the last dose of chemotherapy.

APPLICABLE TO ENTRECTINIB COHORT (COHORT D)

Contraception Requirements after the Last Dose of Study Treatment for Women of Childbearing Potential

Female patients of childbearing potential (see above) should remain abstinent or continue to use highly effective contraception use for at least 5 weeks after the last dose of entrectinib. Women must refrain from donating eggs during this same period.

Contraception Requirements for Men

Male patients treated with entrectinib should remain abstinent or continue to use highly effective contraception during the treatment period and continue contraception use for at

"Use of this document is governed by the terms of use on the first page of this document."

least 3 months after the last dose of entrectinib to avoid exposing the embryo. Male patients must refrain from donating sperm during this same period.

Pregnancy Reporting Requirements

Female patients of childbearing potential will be instructed to immediately inform the investigator if they become pregnant during the study or within 5 weeks after the last dose of study drug.

Male patients will be instructed through the Informed Consent Form to immediately inform the investigator if their partner becomes pregnant during study treatment with chemotherapy or within 3 months after the last dose of study drug.

APPLICABLE TO VEMURAFENIB, COBIMETINIB, AND ATEZOLIZUMAB COHORT (COHORT E)

Contraception Requirements after the Last Dose of Study Treatment for Women of Childbearing Potential

Female patients of childbearing potential (see above) should remain abstinent or continue contraception use for at least 6 months after the last dose of study treatment. Women must refrain from donating eggs during this same period.

Contraception Requirements for Men

Male patients treated with vemurafenib, cobimetinib, and atezolizumab who have a pregnant partner should remain abstinent or use a condom during the treatment period and for at least 6 months after the last dose of study treatment. Males with female partners of childbearing potential should remain abstinent or use a condom plus an additional contraceptive method during the treatment period and for at least 6 months after the last dose of study treatment. Male patients must also refrain from donating sperm during this same period.

APPLICABLE TO ATEZOLIZUMAB, BEVACIZUMAB, CARBOPLATIN, AND PEMETREXED COHORT (COHORT F)

Contraception Requirements after the Last Dose of Study Treatment for Women of Childbearing Potential

Female patients of childbearing potential (see above) should remain abstinent or continue contraception use for 5 months after the last dose of atezolizumab and/or 6 months after the last dose of bevacizumab, whichever is later. Women must refrain from donating eggs during this same period.

Contraception Requirements for Men

During the treatment period, male patients must remain abstinent or use a condom. Male patients with female partners of childbearing potential should continue contraception use for 6 months after the last dose of bevacizumab, carboplatin, or pemetrexed. Male patients must refrain from donating sperm during this same period.

"Use of this document is governed by the terms of use on the first page of this document."

Appendix 3
Response Evaluation Criteria in Solid Tumors, Version 1.1 (RECIST v1.1)

Selected sections from the Response Evaluation Criteria in Solid Tumors, Version 1.1 (RECIST v1.1), (Eisenhauer et al. 2009) are presented below, with slight modifications from the original publication and the addition of explanatory text as needed for clarity.[1]

TUMOR MEASURABILITY

At baseline, tumor lesions/lymph nodes will be categorized as measurable or non-measurable as described below. All measurable and non-measurable lesions should be assessed at screening and at subsequent protocol-specified tumor assessment timepoints. Additional assessments may be performed as clinically indicated for suspicion of progression.

DEFINITION OF MEASURABLE LESIONS

Tumor Lesions

Tumor lesions must be accurately measured in at least one dimension (longest diameter in the plane of measurement is to be recorded) with a minimum size as follows:

- 10 mm by computed tomography (CT) or magnetic resonance imaging (MRI) scan (CT/MRI scan slice thickness/interval $\leq$ 5 mm)
- 10-mm caliper measurement by clinical examination (lesions that cannot be accurately measured with calipers should be recorded as non-measurable)
- 20 mm by chest X-ray

Malignant Lymph Nodes

To be considered pathologically enlarged and measurable, a lymph node must be $\geq$ 15 mm in the short axis when assessed by CT scan (CT scan slice thickness recommended to be $\leq$ 5 mm). At baseline and follow-up, only the short axis will be measured and followed. Additional information on lymph node measurement is provided below (see "Identification of Target and Non-Target Lesions" and "Calculation of Sum of Diameters").

[1] For clarity and for consistency within this document, the section numbers and cross-references to other sections within the article have been deleted and minor changes have been made.

"Use of this document is governed by the terms of use on the first page of this document."

DEFINITION OF NON-MEASURABLE LESIONS

Non-measurable tumor lesions encompass small lesions (longest diameter $<$ 10 mm or pathological lymph nodes with short axis $\geq$ 10 mm but $<$ 15 mm) as well as truly non-measurable lesions. Lesions considered truly non-measurable include leptomeningeal disease, ascites, pleural or pericardial effusion, inflammatory breast disease, lymphangitic involvement of skin or lung, peritoneal spread, and abdominal mass/abdominal organomegaly identified by physical examination that is not measurable by reproducible imaging techniques.

SPECIAL CONSIDERATIONS REGARDING LESION MEASURABILITY

Bone lesions, cystic lesions, and lesions previously treated with local therapy require particular comment, as outlined below.

Bone Lesions:

- Technetium-99m bone scans, sodium fluoride positron emission tomography scans, and plain films are not considered adequate imaging techniques for measuring bone lesions. However, these techniques can be used to confirm the presence or disappearance of bone lesions.
- Lytic bone lesions or mixed lytic-blastic lesions with identifiable soft tissue components that can be evaluated by cross-sectional imaging techniques such as CT or MRI can be considered measurable lesions if the soft tissue component meets the definition of measurability described above.
- Blastic bone lesions are non-measurable.

Cystic Lesions:

- Lesions that meet the criteria for radiographically defined simple cysts should not be considered malignant lesions (neither measurable nor non-measurable) since they are, by definition, simple cysts.
- Cystic lesions thought to represent cystic metastases can be considered measurable lesions if they meet the definition of measurability described above. However, if non-cystic lesions are present in the same patient, these are preferred for selection as target lesions.

Lesions with Prior Local Treatment:

- Tumor lesions situated in a previously irradiated area or in an area subjected to other loco-regional therapy are usually not considered measurable unless there has been demonstrated progression in the lesion. Study protocols should detail the conditions under which such lesions would be considered measurable.

"Use of this document is governed by the terms of use on the first page of this document."

METHODS FOR ASSESSING LESIONS

All measurements should be recorded in metric notation, using calipers if clinically assessed. All baseline evaluations should be performed as close as possible to the treatment start and never more than 4 weeks before the beginning of the treatment.

The same method of assessment and the same technique should be used to characterize each identified and reported lesion at baseline and during the study. Imaging-based evaluation should always be the preferred option.

CLINICAL LESIONS

Clinical lesions will only be considered measurable when they are superficial and $\geq$10 mm in diameter as assessed using calipers (e.g., skin nodules). For the case of skin lesions, documentation by color photography, including a ruler to estimate the size of the lesion, is suggested.

CHEST X-RAY

Chest CT is preferred over chest X-ray, particularly when progression is an important endpoint, since CT is more sensitive than X-ray, particularly in identifying new lesions. However, lesions on chest X-ray may be considered measurable if they are clearly defined and surrounded by aerated lung.

CT AND MRI SCANS

CT is the best currently available and reproducible method to measure lesions selected for response assessment. In this guideline, the definition of measurability of lesions on CT scan is based on the assumption that CT slice thickness is $\leq$5 mm. When CT scans have slice thickness of $>$5 mm, the minimum size for a measurable lesion should be twice the slice thickness. MRI is also acceptable.

If prior to enrollment it is known that a patient is unable to undergo CT scans with intravenous (IV) contrast because of allergy or renal insufficiency, the decision as to whether a non-contrast CT or MRI (without IV contrast) will be used to evaluate the patient at baseline and during the study should be guided by the tumor type under investigation and the anatomic location of the disease. For patients who develop contraindications to contrast after baseline contrast CT is done, the decision as to whether non-contrast CT or MRI (enhanced or non-enhanced) will be performed should also be based on the tumor type and the anatomic location of the disease, and should be optimized to allow for comparison with the prior studies if possible. Each case should be discussed with the radiologist to determine if substitution of these other approaches is possible and, <u>if not, the patient should be considered not evaluable from that point forward</u>. Care must be taken in measurement of target lesions and interpretation of

"Use of this document is governed by the terms of use on the first page of this document."

non-target disease or new lesions on a different modality, since the same lesion may appear to have a different size using a new modality.

ENDOSCOPY, LAPAROSCOPY, ULTRASOUND, TUMOR MARKERS, CYTOLOGY, HISTOLOGY

Endoscopy, laparoscopy, ultrasound, tumor markers, cytology, and histology cannot be used for objective tumor evaluation.

ASSESSMENT OF TUMOR BURDEN

To assess objective response or future progression, it is necessary to estimate the overall tumor burden at baseline and use this as a comparator for subsequent measurements.

IDENTIFICATION OF TARGET AND NON-TARGET LESIONS

When more than one measurable lesion is present at baseline, all lesions up to a maximum of five lesions total (and a maximum of two lesions per organ) representative of all involved organs should be identified as target lesions and will be recorded and measured at baseline. This means that, for instances in which patients have only one or two organ sites involved, a maximum of two lesions (one site) and four lesions (two sites), respectively, will be recorded. Other lesions (albeit measurable) in those organs will be considered non-target lesions.

Target lesions should be selected on the basis of their size (lesions with the longest diameter) and should be representative of all involved organs, but in addition should lend themselves to reproducible repeated measurements. It may be the case that, on occasion, the largest lesion does not lend itself to reproducible measurement, in which circumstance the next largest lesion that can be measured reproducibly should be selected.

Lymph nodes merit special mention since they are normal anatomical structures that may be visible by imaging even if not involved by tumor. As noted above, pathological nodes that are defined as measurable and may be identified as target lesions must meet the criterion of a short axis of $\geq$ 15 mm by CT scan. Only the short axis of these nodes will contribute to the baseline sum. The short axis of the node is the diameter normally used by radiologists to judge if a node is involved by solid tumor. Lymph node size is normally reported as two dimensions in the plane in which the image is obtained (for CT, this is almost always the axial plane; for MRI, the plane of acquisition may be axial, sagittal, or coronal). The smaller of these measures is the short axis. For example, an abdominal node that is reported as being 20 mm $\times$ 30 mm has a short axis of 20 mm and qualifies as a malignant, measurable node. In this example, 20 mm should be recorded as the node measurement. All other pathological nodes (those with short axis $\geq$ 10 mm

"Use of this document is governed by the terms of use on the first page of this document."

but < 15 mm) should be considered non-target lesions. Nodes that have a short axis of < 10 mm are considered non-pathological and should not be recorded or followed.

All lesions (or sites of disease) not selected as target lesions (measurable or non-measurable), including pathological lymph nodes, should be identified as non-target lesions and should also be recorded at baseline. Measurements are not required. It is possible to record multiple non-target lesions involving the same organ as a single item on the Case Report Form (CRF) (e.g., "multiple enlarged pelvic lymph nodes" or "multiple liver metastases").

CALCULATION OF SUM OF DIAMETERS

A sum of the diameters (longest diameter for non–lymph node lesions, short axis for lymph node lesions) will be calculated for all target lesions at baseline and at each subsequent tumor assessment as a measure of tumor burden.

Measuring Lymph Nodes

Lymph nodes identified as target lesions should always have the actual short axis measurement recorded (measured in the same anatomical plane as the baseline examination), even if the node regresses to < 10 mm during the study. Thus, when lymph nodes are included as target lesions, the sum of diameters may not be zero even if complete response criteria are met, since a normal lymph node is defined as having a short axis of < 10 mm.

Measuring Lesions That Become Too Small to Measure

During the study, all target lesions (lymph node and non–lymph node) recorded at baseline should have their actual measurements recorded at each subsequent evaluation, even when very small (e.g., 2 mm). However, sometimes lesions or lymph nodes that are recorded as target lesions at baseline become so faint on CT scan that the radiologist may not feel comfortable assigning an exact measurement and may report them as being too small to measure. When this occurs, it is important that a value be recorded on the CRF, as follows:

- If it is the opinion of the radiologist that the lesion has likely disappeared, the measurement should be recorded as 0 mm.
- If the lesion is believed to be present and is faintly seen but too small to measure, a default value of 5 mm should be assigned and "too small to measure" should be ticked. (Note: It is less likely that this rule will be used for lymph nodes since they usually have a definable size when normal and are frequently surrounded by fat such as in the retroperitoneum; however, if a lymph node is believed to be present and is faintly seen but too small to measure, a default value of 5 mm should be assigned in this circumstance as well and "too small to measure" should also be ticked).

"Use of this document is governed by the terms of use on the first page of this document."

To reiterate, however, if the radiologist is able to provide an actual measurement, that should be recorded, even if it is < 5 mm, and in that case "too small to measure" should not be ticked.

Measuring Lesions That Split or Coalesce on Treatment

When non–lymph node lesions fragment, the longest diameters of the fragmented portions should be added together to calculate the sum of diameters. Similarly, as lesions coalesce, a plane between them may be maintained that would aid in obtaining maximal diameter measurements of each individual lesion. If the lesions have truly coalesced such that they are no longer separable, the vector of the longest diameter in this instance should be the maximum longest diameter for the coalesced lesion.

EVALUATION OF NON-TARGET LESIONS

Measurements are not required for non-target lesions, except that malignant lymph node non-target lesions should be monitored for reduction to < 10 mm in short axis. Non-target lesions should be noted at baseline and should be identified as "present" or "absent" and (in rare cases) may be noted as "indicative of progression" at subsequent evaluations. In addition, if a lymph node lesion shrinks to a non-malignant size (short axis < 10 mm), this should be captured on the CRF as part of the assessment of non-target lesions.

RESPONSE CRITERIA

CRITERIA FOR TARGET LESIONS

Definitions of the criteria used to determine objective tumor response for target lesions are provided below:

- Complete response (CR): Disappearance of all target lesions

  Any pathological lymph nodes must have reduction in short axis to < 10 mm.

- Partial response (PR): At least a 30% decrease in the sum of diameters of all target lesions, taking as reference the baseline sum of diameters, in the absence of CR
- Progressive disease (PD): At least a 20% increase in the sum of diameters of target lesions, taking as reference the smallest sum of diameters on study (including baseline)

  In addition to the relative increase of 20%, the sum of diameters must also demonstrate an absolute increase of ≥ 5 mm.

- Stable disease (SD): Neither sufficient shrinkage to qualify for CR or PR nor sufficient increase to qualify for PD

"Use of this document is governed by the terms of use on the first page of this document."

CRITERIA FOR NON-TARGET LESIONS

Definitions of the criteria used to determine the tumor response for the group of non-target lesions are provided below. While some non-target lesions may actually be measurable, they need not be measured and instead should be assessed only qualitatively at the timepoints specified in the schedule of activities.

- CR: Disappearance of all non-target lesions and (if applicable) normalization of tumor marker level

  All lymph nodes must be non-pathological in size ($<$10 mm short axis).

- Non-CR/Non-PD: Persistence of one or more non-target lesions and/or (if applicable) maintenance of tumor marker level above the normal limits
- PD: Unequivocal progression of existing non-target lesions

SPECIAL NOTES ON ASSESSMENT OF PROGRESSION OF NON-TARGET LESIONS

Patients with Measurable and Non-Measurable Disease

For patients with both measurable and non-measurable disease to achieve unequivocal progression on the basis of the non-target lesions, there must be an overall level of substantial worsening in non-target lesions in a magnitude that, even in the presence of SD or PR in target lesions, the overall tumor burden has increased sufficiently to merit discontinuation of therapy. A modest increase in the size of one or more non-target lesions is usually not sufficient to qualify for unequivocal progression status. The designation of overall progression solely on the basis of change in non-target lesions in the face of SD or PR in target lesions will therefore be extremely rare.

NEW LESIONS

The appearance of new malignant lesions denotes disease progression; therefore, some comments on detection of new lesions are important. There are no specific criteria for the identification of new radiographic lesions; however, the finding of a new lesion should be unequivocal, that is, not attributable to differences in scanning technique, change in imaging modality, or findings thought to represent something other than tumor (for example, some "new" bone lesions may be simply healing or flare of preexisting lesions). This is particularly important when the patient’s baseline lesions show PR or CR. For example, necrosis of a liver lesion may be reported on a CT scan report as a "new" cystic lesion, which it is not.

A lesion identified during the study in an anatomical location that was not scanned at baseline is considered a new lesion and will indicate disease progression.

"Use of this document is governed by the terms of use on the first page of this document."

If a new lesion is equivocal, for example because of its small size, continued therapy and follow-up evaluation will clarify if it represents truly new disease. If repeat scans confirm there is definitely a new lesion, progression should be declared using the date of the initial scan.

CRITERIA FOR OVERALL RESPONSE AT A SINGLE TIMEPOINT

Table 1 provides a summary of the overall response status calculation at each response assessment timepoint for patients who have measurable disease at baseline.

Table 1 Criteria for Overall Response at a Single Timepoint: Patients with Target Lesions (with or without Non-Target Lesions)

Target Lesions	Non-Target Lesions	New Lesions	Overall Response
CR	CR	No	CR
CR	Non-CR/non-PD	No	PR
CR	Not all evaluated	No	PR
PR	Non-PD or not all evaluated	No	PR
SD	Non-PD or not all evaluated	No	SD
Not all evaluated	Non-PD	No	NE
PD	Any	Yes or no	PD
Any	PD	Yes or no	PD
Any	Any	Yes	PD

CR = complete response; NE = not evaluable; PD = progressive disease; PR = partial response; SD = stable disease.

MISSING ASSESSMENTS AND NOT-EVALUABLE DESIGNATION

When no imaging/measurement is done at all at a particular timepoint, the patient is not evaluable at that timepoint. If measurements are made on only a subset of target lesions at a timepoint, usually the case is also considered not evaluable at that timepoint, unless a convincing argument can be made that the contribution of the individual missing lesions would not change the assigned timepoint response. This would be most likely to happen in the case of PD. For example, if a patient had a baseline sum of 50 mm with three measured lesions and during the study only two lesions were assessed, but those gave a sum of 80 mm, the patient will have achieved PD status, regardless of the contribution of the missing lesion.

SPECIAL NOTES ON RESPONSE ASSESSMENT

Patients with a global deterioration in health status requiring discontinuation of treatment without objective evidence of disease progression at that time should be reported as "symptomatic deterioration." Every effort should be made to document objective

"Use of this document is governed by the terms of use on the first page of this document."

progression even after discontinuation of treatment. Symptomatic deterioration is not a descriptor of an objective response; it is a reason for stopping study therapy. The objective response status of such patients is to be determined by evaluation of target and non-target lesions as shown in Table 1.

For equivocal findings of progression (e.g., very small and uncertain new lesions; cystic changes or necrosis in existing lesions), treatment may continue until the next scheduled assessment. If at the next scheduled assessment, progression is confirmed, the date of progression should be the earlier date when progression was suspected.

REFERENCES

Eisenhauer EA, Therasse P, Bogaerts J, et al. New response evaluation criteria in solid tumors: revised RECIST guideline (version 1.1). Eur J Cancer 2009;45:228–47.

"Use of this document is governed by the terms of use on the first page of this document."

Appendix 4
Eastern Cooperative Oncology Group Performance Status Scale

Grade	Description
0	Fully active; able to carry on all pre-disease performance without restriction
1	Restricted in physically strenuous activity but ambulatory and able to carry out work of a light or sedentary nature (e.g., light housework or office work)
2	Ambulatory and capable of all self-care but unable to carry out any work activities; up and about $>$ 50% of waking hours
3	Capable of only limited self-care; confined to a bed or chair $>$ 50% of waking hours
4	Completely disabled; cannot carry on any self-care; totally confined to bed or chair
5	Dead

"Use of this document is governed by the terms of use on the first page of this document."

Appendix 5
List of Substrates, Inhibitors, and Inducers of Drug-Metabolizing Enzymes and Transporters

This representative list is not intended to be an exhaustive list. Each patient's concomitant medications should be carefully considered by the investigator with regard to the benefit–risk for the particular patient and appropriate monitoring, including any concomitant medication, dose adjustment, or therapeutic alternatives, which should be determined by the investigator caring for the patient.

CYP3A Potent Inducers	CYP3A Potent Inhibitors
avasimibe, barbiturates, carbamazepine, efavirenz, ethosuximide, garlic supplements, modafinil, nevirapine, oxcarbazepine, phenobarbital, phenytoin, pioglitazone, primidone, rifabutin, rifampin, rifapentine, St. John's wort, troglitazone	aprepitant, atazanavir, boceprevir, ciprofloxacin, clarithromycin, conivaptan, diltiazem, erythromycin, fluconazole, grapefruit juice, indinavir, itraconazole, ketoconazole, lopinavir, mibefradil, nefazodone, nelfinavir, posaconazole, ritonavir, saquinavir, telaprevir, telithromycin, troleandomycin, verapamil, voriconazole

P-gp		
**Substrates**		**Inducers**
aliskiren, ambrisentan, colchicine, dabigatran, digoxin, everolimus, fexofenadine, imatinib, lapatinib, maraviroc, nilotinib, posaconazole, pravastatin, ranolazine, saxagliptin, sirolimus, sitagliptin, talinolol, tolvaptan, topotecan		avasimibe, carbmazepine, phenytoin, rifampin, St John's wort, tipranavir
**Dual UGT1A1/CYP3A**		
**Substrates**	**Inhibitors**	**Inducers**
buprenorphine, raltegravir	atazanavir	rifampin

Levien TL, and Baker DE. Cytochrome P450 Drug Interactions. Therapeutic Research Center Pharmacist's Letter/Prescriber's Letter [resource on the Internet]. 2003. Available from: www.pharmacistsletter.com and www.prescribersletter.com.

Zhang L. Transporter Mediated Drug-Drug Interactions. FDA. Clinical Pharmacology Advisory Committee Meeting Topic 4: Transporter-Mediated Drug-Drug Interactions Atlanta, GA, March 17, 2010.

This information in this appendix is adapted from Levien and Baker 2003, Zhang 2010, and FDA Guidance on Drug-Drug Interactions.

Also see:

- http://www.fda.gov/Drugs/DevelopmentApprovalProcess/DevelopmentResources/DrugInteractionsLabeling/ucm080499.htm
- http://medicine.iupui.edu/clinpharm/ddis/table.aspx

"Use of this document is governed by the terms of use on the first page of this document."

Potent inhibitors of CYP3A are those considered to be “strong CYP3A inhibitors” previously shown to result in a ≥5-fold increase in the area under the curve of a concomitantly administered CYP3A substrate. These are based on the available published literature and, thus, are not considered exhaustive or inclusive.

The above lists of medications are not necessarily comprehensive. Thus, the investigator should consult the prescribing information for any concomitant medication as well as the Internet references provided below when determining whether a certain medication strongly inhibits or induces CYP3A4, or inhibits P-gp. In addition, the investigator should contact the Medical Monitor if questions arise regarding medications not listed above. See FDA Guidance on Drug-Drug Interactions for further detail.

"Use of this document is governed by the terms of use on the first page of this document."

Appendix 6
Patient-Reported Outcomes (PRO) Questionnaires

EORTC QLQ-30

EORTC QLQ-C30 (version 3)

We are interested in some things about you and your health. Please answer all of the questions yourself by circling the number that best applies to you. There are no "right" or "wrong" answers. The information that you provide will remain strictly confidential.

Please fill in your initials: |__|__|__|__|
Your birthdate (Day, Month, Year): |__|__|__|__|__|__|__|__|
Today's date (Day, Month, Year): 31 |__|__|__|__|__|__|__|__|

	Not at All	A Little	Quite a Bit	Very Much
1. Do you have any trouble doing strenuous activities, like carrying a heavy shopping bag or a suitcase?	1	2	3	4
2. Do you have any trouble taking a long walk?	1	2	3	4
3. Do you have any trouble taking a short walk outside of the house?	1	2	3	4
4. Do you need to stay in bed or a chair during the day?	1	2	3	4
5. Do you need help with eating, dressing, washing yourself or using the toilet?	1	2	3	4

During the past week:	Not at All	A Little	Quite a Bit	Very Much
6. Were you limited in doing either your work or other daily activities?	1	2	3	4
7. Were you limited in pursuing your hobbies or other leisure time activities?	1	2	3	4
8. Were you short of breath?	1	2	3	4
9. Have you had pain?	1	2	3	4
10. Did you need to rest?	1	2	3	4
11. Have you had trouble sleeping?	1	2	3	4
12. Have you felt weak?	1	2	3	4
13. Have you lacked appetite?	1	2	3	4
14. Have you felt nauseated?	1	2	3	4
15. Have you vomited?	1	2	3	4
16. Have you been constipated?	1	2	3	4

Please go on to the next page

Specimen

"Use of this document is governed by the terms of use on the first page of this document."

During the past week:	Not at All	A Little	Quite a Bit	Very Much
17. Have you had diarrhea?	1	2	3	4
18. Were you tired?	1	2	3	4
19. Did pain interfere with your daily activities?	1	2	3	4
20. Have you had difficulty in concentrating on things, like reading a newspaper or watching television?	1	2	3	4
21. Did you feel tense?	1	2	3	4
22. Did you worry?	1	2	3	4
23. Did you feel irritable?	1	2	3	4
24. Did you feel depressed?	1	2	3	4
25. Have you had difficulty remembering things?	1	2	3	4
26. Has your physical condition or medical treatment interfered with your family life?	1	2	3	4
27. Has your physical condition or medical treatment interfered with your social activities?	1	2	3	4
28. Has your physical condition or medical treatment caused you financial difficulties?	1	2	3	4

For the following questions please circle the number between 1 and 7 that best applies to you

29. How would you rate your overall health during the past week?

1	2	3	4	5	6	7
Very poor						Excellent

30. How would you rate your overall quality of life during the past week?

1	2	3	4	5	6	7
Very poor						Excellent

© Copyright 1995 EORTC Quality of Life Group. All rights reserved. Version 3.0

Specimen

"Use of this document is governed by the terms of use on the first page of this document."

SYMPTOMS IN LUNG CANCER (SILC)

E01108			–		
Project ID	Site ID			Subject ID	

Symptoms in Lung Cancer (SILC)

Instructions: Please answer the following questions thinking about your lung cancer symptoms over the **past week.**

Item #	Question
1.	Over the past week, how would you rate your chest pain at its worst?
	☐$_0$ No pain
	☐$_1$ Mild pain
	☐$_2$ Moderate pain
	☐$_3$ Severe pain
	☐$_4$ Very severe pain
2.	Over the past week, how often did you have chest pain?
	☐$_0$ Never
	☐$_1$ Rarely
	☐$_2$ Sometimes
	☐$_3$ Often
	☐$_4$ Always
3.	Over the past week, how would you rate your coughing at its worst?
	☐$_0$ No coughing at all
	☐$_1$ Mild coughing
	☐$_2$ Moderate coughing
	☐$_3$ Severe coughing
	☐$_4$ Very severe coughing

"Use of this document is governed by the terms of use on the first page of this document."

EO1108			–		
Project ID	Site ID			Subject ID	

Item #	Question
4.	Over the past week, how often did you cough?

☐$_0$ Never

☐$_1$ Rarely

☐$_2$ Sometimes

☐$_3$ Often

☐$_4$ Always

5.	Over the past week, how often did you feel short of breath when lying down or sitting?

☐$_0$ Never

☐$_1$ Rarely

☐$_2$ Sometimes

☐$_3$ Often

☐$_4$ Always

6.	Over the past week, how often did you feel short of breath when standing for less than 5 minutes?

☐$_0$ Never

☐$_1$ Rarely

☐$_2$ Sometimes

☐$_3$ Often

☐$_4$ Always

☐$_5$ I did not stand because it was too difficult to do

"Use of this document is governed by the terms of use on the first page of this document."

E01108			–		
Project ID	Site ID			Subject ID	

Item #	Question
7.	Over the past week, how often did you feel short of breath when walking for 2-5 minutes?

☐$_0$ Never

☐$_1$ Rarely

☐$_2$ Sometimes

☐$_3$ Often

☐$_4$ Always

☐$_5$ I did not walk 2-5 minutes because it was too difficult to do

8. Over the past week, how often did you feel short of breath when lifting and carrying a light load?

☐$_0$ Never

☐$_1$ Rarely

☐$_2$ Sometimes

☐$_3$ Often

☐$_4$ Always

☐$_5$ I did not lift and carry a light load because it was too difficult to do

9. Over the past week, how often did you feel short of breath when walking up a flight of stairs or hill?

☐$_0$ Never

☐$_1$ Rarely

☐$_2$ Sometimes

☐$_3$ Often

☐$_4$ Always

☐$_5$ I did not walk up a flight of stairs or hill because it was too difficult to do

"Use of this document is governed by the terms of use on the first page of this document."

EQ-5D-5L

Health Questionnaire

English version for the USA

USA (English) © 2009 EuroQol Group EQ-5D™ is a trade mark of the EuroQol Group

"Use of this document is governed by the terms of use on the first page of this document."

Under each heading, please check the ONE box that best describes your health TODAY.

MOBILITY

I have no problems walking ❑

I have slight problems walking ❑

I have moderate problems walking ❑

I have severe problems walking ❑

I am unable to walk ❑

SELF-CARE

I have no problems washing or dressing myself ❑

I have slight problems washing or dressing myself ❑

I have moderate problems washing or dressing myself ❑

I have severe problems washing or dressing myself ❑

I am unable to wash or dress myself ❑

USUAL ACTIVITIES *(e.g. work, study, housework, family or leisure activities)*

I have no problems doing my usual activities ❑

I have slight problems doing my usual activities ❑

I have moderate problems doing my usual activities ❑

I have severe problems doing my usual activities ❑

I am unable to do my usual activities ❑

PAIN / DISCOMFORT

I have no pain or discomfort ❑

I have slight pain or discomfort ❑

I have moderate pain or discomfort ❑

I have severe pain or discomfort ❑

I have extreme pain or discomfort ❑

ANXIETY / DEPRESSION

I am not anxious or depressed ❑

I am slightly anxious or depressed ❑

I am moderately anxious or depressed ❑

I am severely anxious or depressed ❑

I am extremely anxious or depressed ❑

2

USA (English) © 2009 EuroQol Group EQ-5D™ is a trade mark of the EuroQol Group

"Use of this document is governed by the terms of use on the first page of this document."

- We would like to know how good or bad your health is TODAY.
- This scale is numbered from 0 to 100.
- 100 means the best health you can imagine.
  0 means the worst health you can imagine.
- Mark an X on the scale to indicate how your health is TODAY.
- Now, please write the number you marked on the scale in the box below.

YOUR HEALTH TODAY = ☐

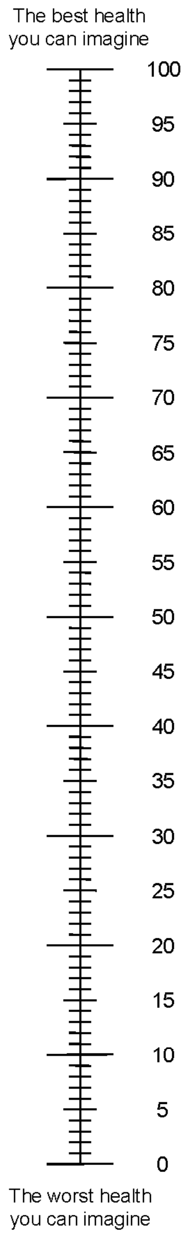

USA (English) © 2009 EuroQol Group EQ-5D™ is a trade mark of the EuroQol Group

"Use of this document is governed by the terms of use on the first page of this document."

EORTC QLQ-BN20

ENGLISH

EORTC QLQ - BN20

Patients sometimes report that they have the following symptoms. Please indicate the extent to which you have experienced these symptoms or problems during the past week.

During the past week:	Not at All	A Little	Quite a Bit	Very Much
31. Did you feel uncertain about the future?	1	2	3	4
32. Did you feel you had setbacks in your condition?	1	2	3	4
33. Were you concerned about disruption of family life?	1	2	3	4
34. Did you have headaches?	1	2	3	4
35. Did your outlook on the future worsen?	1	2	3	4
36. Did you have double vision?	1	2	3	4
37. Was your vision blurred?	1	2	3	4
38. Did you have difficulty reading because of your vision?	1	2	3	4
39. Did you have seizures?	1	2	3	4
40. Did you have weakness on one side of your body?	1	2	3	4
41. Did you have trouble finding the right words to express yourself?	1	2	3	4
42. Did you have difficulty speaking?	1	2	3	4
43. Did you have trouble communicating your thoughts?	1	2	3	4
44. Did you feel drowsy during the daytime?	1	2	3	4
45. Did you have trouble with your coordination?	1	2	3	4
46. Did hair loss bother you?	1	2	3	4
47. Did itching of your skin bother you?	1	2	3	4
48. Did you have weakness of both legs?	1	2	3	4
49. Did you feel unsteady on your feet?	1	2	3	4
50. Did you have trouble controlling your bladder?	1	2	3	4

© Copyright 1994 EORTC Quality of Life Group

SPECIMEN

"Use of this document is governed by the terms of use on the first page of this document."

Appendix 9
Cohort C: Atezolizumab versus Chemotherapy in Patients with bTMB+ NSCLC

13. COHORT C: ATEZOLIZUMAB VERSUS CHEMOTHERAPY IN PATIENTS WITH bTMB+ NSCLC

TABLE OF CONTENTS

13. COHORT C: ATEZOLIZUMAB VERSUS CHEMOTHERAPY IN PATIENTS WITH BTMB+ NSCLC 212

13.1 Background and Rationale: bTMB+ Cohort 214

13.2 Objectives and Corresponding Endpoints: bTMB+ Cohort 217

13.3 Cohort Design: bTMB+ Cohort 218

13.4 Materials and Methods: bTMB+ Cohort 221

13.4.1 Patients 221

13.4.1.1 Additional Inclusion Criteria 221

13.4.1.2 Additional Exclusion Criteria 223

13.4.2 Method of Treatment Assignment 226

13.4.3 Study Treatment 227

13.4.3.1 Study Treatment Formulation, Packaging, and Handling 227

13.4.3.2 Study Treatment Dosage, Administration, and Compliance 227

13.4.4 Concomitant Therapy and Additional Restrictions 233

13.4.4.1 Permitted Therapy 233

13.4.4.2 Cautionary Therapy for Atezolizumab-Treated Patients 234

13.4.4.3 Prohibited Therapy 234

13.4.5 Cohort Assessments 235

13.4.6 Study Treatment Discontinuation 236

13.5 Assessment of Safety: bTMB+ Cohort 237

13.5.1 Safety Plan 238

13.5.1.1 Risks Associated with Atezolizumab 238

13.5.1.2 Risks Associated with Pemetrexed 238

13.5.1.3 Risks Associated with Gemcitabine 239

13.5.1.4 Risks Associated with Cisplatin 239

"Use of this document is governed by the terms of use on the first page of this document."

13.5.1.5 Risks Associated with Carboplatin 239

13.5.1.6 Dose Modifications 239

13.5.1.7 Management of Atezolizumab-Specific Adverse Events 240

13.5.1.8 Pemetrexed Dose Modifications, Treatment Delays, or Treatment Discontinuation and Management of Specific Adverse Events 241

13.5.1.9 Gemcitabine Dose Modifications, Treatment Delays, or Treatment Discontinuation and Management of Specific Adverse Events 243

13.5.1.10 Non-Hematologic Toxicities 244

13.5.1.11 Cisplatin Dose Modifications, Treatment Delays, or Treatment Discontinuation and Management of Specific Adverse Events 245

13.5.1.12 Carboplatin Dose Modifications, Treatment Delays, or Treatment Discontinuation and Management of Specific Adverse Events 247

13.5.2 Safety Parameters and Definitions 249

13.5.2.1 Adverse Events of Special Interest (Immediately Reportable to the Sponsor) 249

13.5.2.2 Adverse Event Reporting Period 250

13.6 Statistical Considerations: bTMB+ Cohort 250

13.6.1 Determination of Sample Size 251

13.6.2 Efficacy Analyses 254

13.6.2.1 Primary Efficacy Endpoint and Hypothesis Testing 254

13.6.2.2 Secondary Efficacy Endpoints 255

13.6.2.3 Patient-Reported Outcome Analyses 257

13.6.2.4 Exploratory Analysis 258

13.6.3 Interim Analysis 259

13.6.3.1 Planned Interim Analysis 259

13.6.3.2 Optional Interim Analysis 260

13.7 References: bTMB+ Cohort 261

13.8 Schedule of Activities: bTMB+ Cohort 264

"Use of this document is governed by the terms of use on the first page of this document."

13.1 BACKGROUND AND RATIONALE: bTMB+ COHORT

Encouraging clinical data emerging in the field of tumor immunotherapy have demonstrated that therapies focused on enhancing T-cell responses against cancer can result in a significant survival benefit in patients with advanced malignancies (Hodi et al. 2010; Kantoff et al. 2010; Chen et al. 2012).

The PD-L1 pathway serves as an immune checkpoint to temporarily dampen immune responses in states of chronic antigen stimulation, such as chronic infection or cancer. PD-L1 is an extracellular protein that downregulates immune responses through binding to its two receptors, PD-1 and B7-1. PD-1 is an inhibitory receptor expressed on T cells following T-cell activation, and expression is sustained in states of chronic stimulation (Blank et al. 2005; Keir et al. 2008). B7-1 is a molecule expressed on antigen-presenting cells and activated T cells. Binding of PD-L1 to PD-1 and B7-1 inhibits T-cell proliferation and activation, cytokine production, and cytolytic activity, leading to the functional inactivation or exhaustion of T cells (Butte et al. 2007; Yang et al. 2011). Overexpression of PD-L1 on tumor cells has been reported to impede anti-tumor immunity, resulting in immune evasion (Blank and Mackensen 2007). Therefore, interruption of the PD-L1 pathway represents an attractive strategy for restoring tumor-specific T-cell immunity.

Atezolizumab is a humanized Ig G1 monoclonal antibody that targets PD-L1 and inhibits the interaction between PD-L1 and its receptors, PD-1 and B7-1 (also known as CD80), both of which function as inhibitory receptors expressed on T cells. Therapeutic blockade of PD-L1 binding by atezolizumab has been shown to enhance the magnitude and quality of tumor-specific T-cell responses, resulting in improved antitumor activity (Fehrenbacher et al. 2016; Rosenberg et al. 2016). Atezolizumab has minimal binding to Fc receptors, thus eliminating detectable Fc-effector function and associated antibody-mediated clearance of activated effector T cells.

Targeting the PD-L1 pathway with atezolizumab has demonstrated activity in patients with advanced malignancies who have failed standard-of-care therapies. Objective responses have been observed across a broad range of malignancies, including non–NSCLC, urothelial carcinoma, renal cell carcinoma, melanoma, colorectal cancer, head and neck cancer, gastric cancer, breast cancer, and sarcoma (see Atezolizumab Investigator's Brochure for detailed efficacy results).

Atezolizumab has been generally well tolerated. Adverse events with potentially immune-mediated causes consistent with an immunotherapeutic agent, including rash, influenza-like illness endocrinopathies, hepatitis or transaminitis, pneumonitis colitis, and

"Use of this document is governed by the terms of use on the first page of this document."

myasthenia gravis, have been observed (see Atezolizumab Investigator's Brochure for detailed safety results). To date, these events have been manageable with treatment.

Atezolizumab shows anti-tumor activity in both nonclinical models and cancer patients and is being investigated as a potential therapy in a wide variety of malignancies.

Atezolizumab is approved for the treatment of urothelial carcinoma, NSCLC, small-cell lung cancer, and triple-negative breast cancer.

Refer to the Atezolizumab Investigator's Brochure for details on nonclinical and clinical studies.

This cohort of the Blood First Assay Screening Trial (BFAST) umbrella trial will aim to enroll patients with untreated metastatic NSCLC who are otherwise not eligible for another cohort (i.e., driver mutation identified via blood-based assay and targeted therapy cohort open on the study) and who are selected using a predictive biomarker to anti-PD-L1 therapy (discussed in detail below). Given the emerging evidence of activity of atezolizumab and other PD-L1 inhibiting agents in relapsed NSCLC, it is rational to explore whether it can improve the outcome in the treatment-naive setting when compared with cytotoxic chemotherapy. This is particularly relevant to the subgroup of first-line patients who are appropriately selected by a biomarker predictive of response. The benefit–risk ratio for atezolizumab is expected to be acceptable in this setting.

This randomized study design is based on the assumption that in patients with Stage IV NSCLC who are chemotherapy-naive and whose tumors are screened and selected for high TMB, treatment with atezolizumab may prolong progression-free survival (PFS) or overall survival (OS) compared with treatment with platinum-based chemotherapy.

Despite the demonstrated clinical efficacy of PD-L1/PD-L1 inhibitors in NSCLC, PD-L1 expression as determined by immunohistochemistry does not appear to clearly identify the patients who derive substantial clinical benefit from these drugs. Emerging evidence suggests that patients with a high burden of somatic mutations derive significant clinical benefit from anti−PD-1/PD-L1 therapy and has been observed in a variety of cancer types including NSCLC (Alexandrov et al. 2013; Lawrence et al. 2013; Vogelstein et al. 2013; Kowanetz et al. 2016). Multiple clinical studies have reported that high tumor mutational burden (TMB) measured by whole-exome sequencing is associated with response to several immunotherapeutic agents, including anti-CTLA-4 in melanoma (Snyder et al. 2014; Johnson et al. 2016), anti-PD-L1 therapy in bladder cancer (Rosenberg et al. 2016), and anti-PD-1 therapy in lung and colorectal cancers (Le et al. 2015; Fehrenbacher et al. 2016). The accumulation of mutations is a central part of the oncogenic process and highly mutated tumors are associated with an increased

"Use of this document is governed by the terms of use on the first page of this document."

likelihood of generating mutated neoantigens that can be recognized by immune cells as foreign (Rizvi et al. 2015). Tumors with high TMB may therefore represent highly immunogenic tumors.

Foundation Medicine has developed (and CLIA has validated) a method to quantify TMB from formalin-fixed, paraffin-embedded tissue on FoundationOne®, an NGS assay that interrogates more than 300 genes, by measuring the somatic mutations occurring in the tested genes and extrapolating to the genome as a whole (Johnson et al. 2016; Rosenberg et al. 2016). In an independent study, the TMB assessed by FoundationOne® has been shown to correlate strongly with genome-wide measures of TMB (Campesato et al. 2015).

Foundation Medicine and Genentech have co-developed a blood-based tumor mutational burden (bTMB) assay that reliably calls TMB count in the blood using a version of the FoundationOne test adapted for blood. In order to validate the utility of the test, more than 250 plasma samples from a Phase II trial of atezolizumab in second-line NSCLC were retrospectively tested and showed significant clinical benefit across a tight range of cutoffs for positivity that produced PFS hazard ratios (HRs) of 0.49–0.68 and OS HRs of 0.5–0.6. These cutoffs from the Phase II study were then prospectively validated on approximately 800 plasma samples from a large Phase III trial of atezolizumab in NSCLC to produce similar PFS and OS HRs.

Two cutoffs for bTMB from within the aforementioned range will be clinically validated prospectively in Cohort C of BFAST. Patients will be enrolled at a lower cutoff with a validated PFS benefit, and the primary analysis will include patients at a higher cutoff to test for PFS benefit. In a planned hierarchical analysis, the PFS of the lower cutoff would be tested if the primary analysis was positive (see Figure C-3). The design of enrolling at a moderate cutoff and stratifying at a higher cutoff will enable clinical evaluation of multiple cutoffs within Cohort C.

The bTMB assay will be analytically validated and implemented in a CAP-CLIA laboratory before the start of the trial.

Despite recent improvements in treatment, the prognosis for patients with advanced NSCLC remains dismal, with a median OS of approximately 12.5 months (Sandler et al. 2006). Patients who receive second-line treatment for their disease have an even more limited prognosis, with median survival duration of approximately 8 to 9 months (Stinchcombe et al. 2008). Approved therapies are associated with significant toxicities (e.g., neuropathy, febrile neutropenia, myelosuppression, and alopecia) that negatively impact quality of life. Therefore, there is a continuing need for more efficacious, better tolerated treatments in the first-line setting.

"Use of this document is governed by the terms of use on the first page of this document."

13.2 OBJECTIVES AND CORRESPONDING ENDPOINTS: bTMB+ COHORT

EFFICACY OBJECTIVES	
**Primary Efficacy Objective**	**Corresponding Endpoint**
• To evaluate the efficacy of atezolizumab compared with platinum-based chemotherapy consisting of a platinum agent (cisplatin or carboplatin) in combination with either pemetrexed (nonsquamous disease) or gemcitabine (squamous disease) in chemotherapy-naive patients with inoperable Stage IIIB or Stage IV NSCLC in patients who are biomarker positive by the bTMB assay	• Investigator-assessed PFS according to RECIST v1.1 in bTMB PP1
**Secondary Efficacy Objective:**	**Corresponding Endpoints**
• To evaluate the efficacy of atezolizumab compared with platinum-based chemotherapy	• OS in bTMB PP1<br>• Investigator-assessed PFS according to RECIST v1.1 in bTMB PP2<br>• OS in bTMB PP2<br>• IRF-assessed PFS, ORR, and DOR according to RECIST v1.1<br>• Investigator-assessed ORR, and DOR according to RECIST v1.1<br>• Investigator-assessed PFS rates at 6-month and 1-year landmark timepoints
SAFETY OBJECTIVE	
**Safety Objective**	**Corresponding Endpoint**
• To evaluate the safety and tolerability of atezolizumab compared with platinum-based chemotherapy	• Incidence, type, and severity of adverse events (based on NCI CTCAE v4.0), including SAEs and adverse events of special interest (as identified for individual cohorts)
EXPLORATORY OBJECTIVE	
**Exploratory Objective**	**Corresponding Endpoints**
• To evaluate the efficacy of atezolizumab compared with platinum-based chemotherapy	• Investigator-assessed TTR, [redacted], and DCR according to RECIST v1.1<br>• OS rate at 1 and 2 years in each treatment arm
BIOMARKER OBJECTIVE	
**Biomarker Objective**	**Corresponding Endpoints**
• To assess predictive, prognostic, and pharmacodynamic exploratory biomarkers in blood, and their association with disease status, mechanisms of resistance, and/or response to atezolizumab	• Relationship between circulating biomarkers related to atezolizumab efficacy

"Use of this document is governed by the terms of use on the first page of this document."

PATIENT-REPORTED OUTCOME OBJECTIVES	
**PRO Objective**	**Corresponding Endpoints**
• To determine the impact of atezolizumab on PROs compared with platinum-based chemotherapy	• TTD and change from baseline in patient-reported lung cancer symptom (cough, dyspnea, chest pain) score as assessed by the SILC<br>• Change from baseline in PROs of HRQoL, symptoms, and functioning as assessed by the EORTC QLQ-C30
• To evaluate and compare patients' health status to generate utility scores for use in economic models for reimbursement	• Health status as assessed by the EQ-5D-5L questionnaire

bTMB = blood tumor mutational burden; bTMB PP1 = the primary population of patients with a bTMB level equal to or greater than the higher validated cutoff; bTMB PP2 = the secondary population of all patients who are bTMB-positive, which is the intent-to-treat (ITT) population in this cohort; DCR = disease control rate; DOR = duration of response; EORTC = European Organisation for Research and Treatment of Cancer; EQ-5D-5L = EuroQol 5-Dimension, 5-Level questionnaire; HRQoL = health-related quality of life; IRF = independent review facility; NCI CTCAE v4.0 = National Cancer Institute Common Terminology Criteria for Adverse Events, Version 4.0; NSCLC = non–small cell lung cancer; ORR = objective response rate; OS = overall survival; PFS = progression-free survival; PRO = patient-reported outcome; QLQ-C30 = Quality of Life Questionnaire Core 30; RECIST v1.1 = Response Evaluation Criteria in Solid Tumors, Version 1.1; SAE = serious adverse event; SILC = Symptoms in Lung Cancer; [redacted]; TTD = time to deterioration; TTR = time to response.

13.3 COHORT DESIGN: bTMB+ COHORT

This is a randomized, open-label cohort designed to evaluate the safety and efficacy of atezolizumab compared with chemotherapy consisting of a platinum agent (cisplatin or carboplatin per investigator discretion) combined with either pemetrexed (non-squamous disease) or gemcitabine (squamous disease) in patients who are biomarker positive by the bTMB assay.

To assure balance during randomization, a number of stratification factors will be used. Histology and Eastern Cooperative Oncology Group (ECOG) Performance Status will be used, as they are accepted to have potential prognostic implications. Because two distinct cutoffs will be evaluated for bTMB, patients will be stratified according to whether they are considered "bTMB moderate" or "bTMB high." Finally, as the trial has the potential to enroll all patients with NSCLC, including patients without tissue, the availability of tissue will be used as an additional stratification factor to prevent an imbalance between those patients with and without tissue, in the event this is prognostic.

The study schema is shown in Figure C-1. A schedule of activities is provided in Section 13.8.

"Use of this document is governed by the terms of use on the first page of this document."

Figure C-1 Schema: bTMB+ Cohort

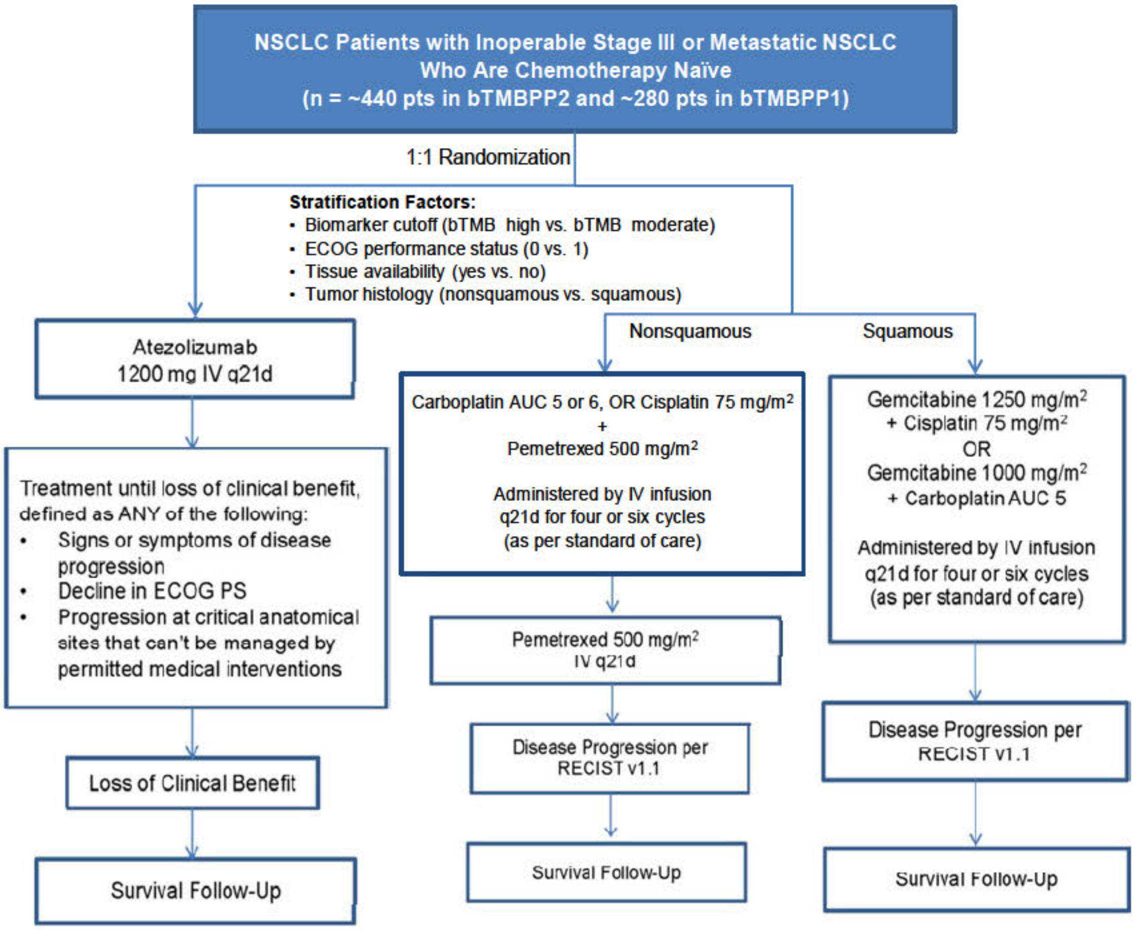

AUC = area under the concentration-time curve; bTMB = blood tumor mutational burden; bTMB PP1 = primary population of patients with a bTMB level equal to or greater than the higher validated cutoff; bTMB PP2 = secondary population of all patients who are bTMB+ (ITT population); bTMB high = bTMB PP1; bTMB moderate = bTMB level between bTMB higher and lower cutoffs for PP1 and PP2, respectively; ECOG PS = Eastern Cooperative Oncology Group Performance Status; ITT = intent-to-treat IV = intravenous; NSCLC = non–small cell lung cancer; PD-L1 = programmed death–ligand 1; q21d = every 21 days; RECIST v1.1 = Response Evaluation Criteria in Solid Tumors, Version 1.1.

Note: Gemcitabine is given on Days 1 and 8.

Given the toxicities associated with platinum-based chemotherapies (e.g., neutropenia, anemia) and the requirement for pre-medications, this will be an open-label study. No crossover will be allowed from the control arm (platinum-based chemotherapy) to the experimental arm (atezolizumab).

"Use of this document is governed by the terms of use on the first page of this document."

Atezolizumab (fixed dose of 1200 mg) will be administered intravenously on Day 1 of each 21-day cycle. Atezolizumab treatment may continue as long as patients are experiencing clinical benefit as assessed by the investigator (i.e., in the absence of unacceptable toxicity or symptomatic deterioration attributed to disease progression as determined by the investigator after an integrated assessment of radiographic data, biopsy results [if available], and clinical status) or until unacceptable toxicity or death.

During treatment, patients who are treated with atezolizumab and who show evidence of clinical benefit may be permitted to continue atezolizumab treatment after Response Evaluation Criteria in Solid Tumors, Version 1.1 (RECIST v1.1) for progressive disease are met if they meet all of the following criteria:

- Evidence of clinical benefit as assessed by the investigator
- Absence of symptoms and signs (including worsening of laboratory values [e.g., new or worsening hypercalcemia]) indicating unequivocal progression of disease
- No decline in ECOG Performance Status that can be attributed to disease progression
- Absence of tumor progression at critical anatomical sites (e.g., leptomeningeal disease) that cannot be managed by protocol-allowed medical interventions
- Patients must provide written consent to acknowledge deferring other treatment options in favor of continuing study treatment at the time of initial radiographic progression per RECIST v1.1

Patients randomized to receive pemetrexed in combination with either cisplatin or carboplatin (non-squamous disease) will receive chemotherapy intravenously on Day 1 of each 21-day cycle for four or six cycles as per local standard of care, followed by maintenance therapy with pemetrexed as per local standard of care. Patients randomized to receive gemcitabine in combination with either cisplatin or carboplatin (squamous disease) will receive cisplatin or carboplatin intravenously on Day 1 and gemcitabine intravenously on Days 1 and 8 of each 21-day cycle for four or six cycles, as per local standard of care, followed by best supportive care. The intended number of cycles planned for the platinum-based induction chemotherapy (i.e., four or six cycles) will be specified by the investigator prior to study randomization. Treatment will continue until disease progression, unacceptable toxicity, or death. Refer to Section 13.4.3.2 for treatment administration details.

All patients will undergo tumor assessment at baseline and every 6 weeks (± 7 days) for 48 weeks following Cycle 1, Day 1 regardless of treatment delays. After the completion of the Week 48 tumor assessment, tumor assessment will be required every 9 weeks (± 7 days) regardless of treatment delays, until radiographic disease progression per

"Use of this document is governed by the terms of use on the first page of this document."

RECIST v1.1 (or loss of clinical benefit for atezolizumab-treated patients who continue treatment beyond disease progression according to RECIST v1.1), withdrawal of consent, death, or study termination by the Sponsor, whichever occurs first. Patients who discontinue treatment for reasons other than disease progression (e.g., toxicity) will continue scheduled tumor assessments until disease progression per RECIST v1.1, withdrawal of consent, death, or study termination by Sponsor, whichever occurs first. In the absence of disease progression, tumor assessments should continue regardless of whether patients start a new anti-cancer therapy.

A secondary endpoint of this study is independent review facility (IRF)-assessed PFS according to RECIST v1.1. Therefore, an IRF will conduct an independent review of the responses of all patients, including a review of blinded computed tomography (CT) scans. All primary imaging data used for tumor assessment will be collected by the Sponsor to enable centralized, independent review of response endpoints. These reviews will be performed prior to the final efficacy analyses.

13.4 MATERIALS AND METHODS: bTMB+ COHORT

13.4.1 Patients

To be enrolled in this cohort, patients must have met and continue to meet all eligibility criteria specified for the blood-based NGS ctDNA assay screening (see Section 4.1), in addition to the cohort-specific criteria below.

If, and as long as, your study site is participating in Study GO29431 (the IMpower110 trial), the Sponsor requests that patients being considered for BFAST are patients who have failed screening for IMpower110 or do not have tissue available/accessible to screen for IMpower110.

To be enrolled into this cohort, patients must have met and continue to meet all eligibility criteria specified for the blood-based next-generation sequencing ctDNA assay screening (see Section 4.1), in addition to the cohort-specific criteria below.

13.4.1.1 Additional Inclusion Criteria

Patients must meet the following additional criteria for entry in Cohort C:

- bTMB+, as detected via the blood-based assay
- Signed cohort-specific informed consent for treatment
- ECOG Performance Status of 0 or 1
- For women of childbearing potential (see Appendix 2), a negative serum pregnancy test ≤7 days prior to initial cohort treatment

"Use of this document is governed by the terms of use on the first page of this document."

- No prior treatment for inoperable Stage IIIB not amenable to treatment with combined modality chemoradiation (advanced) or metastatic non-squamous or squamous NSCLC

  Patients who have received prior neo-adjuvant, adjuvant chemotherapy, radiotherapy, or chemoradiotherapy with curative intent for non-metastatic disease must have experienced a treatment-free interval of at least 6 months from randomization since the last chemotherapy, radiotherapy, or chemoradiotherapy cycle.

- Patients with a history of treated, asymptomatic CNS metastases are eligible, provided they meet all of the following criteria:

  Only supratentorial and cerebellar metastases allowed (i.e., no metastases to midbrain, pons, medulla, or spinal cord)

  No ongoing requirement for corticosteroids as therapy for CNS disease

  No stereotactic radiation within 7 days or whole-brain radiation within 14 days prior to randomization

  No evidence of interim progression between the completion of CNS-directed therapy and the screening radiographic study

    Patients with new asymptomatic CNS metastases detected at the screening scan must receive radiation therapy and/or surgery for CNS metastases. Following treatment, these patients may then be eligible without the need for an additional brain scan prior to randomization, if all other criteria are met.

- Negative HIV test at screening for cohort
- Negative hepatitis B surface antigen (HBsAg) test at screening for cohort
- Negative total hepatitis B core antibody (HBcAb) test at screening for cohort, or positive total HBcAb test followed by a negative hepatitis B virus (HBV) DNA test at screening

  The HBV DNA test will be performed only for patients who have a positive total HBcAb test.

- Negative hepatitis C virus (HCV) antibody test at screening, or positive HCV antibody test followed by a negative HCV RNA test at screening

  The HCV RNA test will be performed only for patients who have a positive HCV antibody test.

- Adequate hematologic and end-organ function, defined by the following laboratory test results obtained within 14 days prior to randomization:

  ANC $\geq$ 1500 cells/μL without granulocyte colony-stimulating factor support

  Lymphocyte count $\geq$ 500 cells/μL

"Use of this document is governed by the terms of use on the first page of this document."

Platelet count ≥ 100,000 cells/μL without transfusion

INR or aPTT ≤ 1.5 × upper limit of normal (ULN)

This applies only to patients who are not receiving therapeutic anticoagulation; patients receiving therapeutic anticoagulation must have an INR or aPTT within therapeutic limits for at least 1 week prior to randomization.

Alkaline phosphatase ≤ 2.5 × ULN with the following exceptions:

Patients with documented liver metastases: Alkaline phosphatase ≤ 5 × ULN

Serum bilirubin ≤ 1.5 × ULN

Patients with known Gilbert disease who have serum bilirubin level ≤ 3 × ULN may be enrolled.

13.4.1.2 Additional Exclusion Criteria

Patients who meet any of the following additional criteria will be excluded from entry in Cohort C:

<u>Cancer-Specific Exclusions</u>

- Presence of activating EGFR or ALK genetic alteration considered exclusionary by the blood-based assay (F1LCDx)

  The F1LCDx assay reports both known and novel alterations. Only patients with known oncogenic alterations in EGFR or ALK will be excluded from BFAST. Alterations in EGFR or ALK where oncogenic potential is unknown (i.e., variants of unknown significance) are not considered exclusionary.

- Spinal cord compression not definitively treated with surgery and/or radiation, or previously diagnosed and treated spinal cord compression without evidence that disease has been clinically stable for ≥ 2 weeks prior to randomization
- Leptomeningeal disease
- Uncontrolled tumor-related pain

  Patients requiring pain medication must be on a stable regimen at study entry.

  Symptomatic lesions amenable to palliative radiotherapy (e.g., bone metastases or metastases causing nerve impingement) should be treated prior to randomization. Patients should be recovered from the effects of radiation. There is no required minimum recovery period.

  Asymptomatic metastatic lesions whose further growth would likely cause functional deficits or intractable pain (e.g., epidural metastasis that is not currently associated with spinal cord compression) should be considered for loco-regional therapy if appropriate prior to enrollment.

"Use of this document is governed by the terms of use on the first page of this document."

- Uncontrolled pleural effusion, pericardial effusion, or ascites requiring recurrent drainage procedures (once monthly or more frequently)

    Patients with indwelling catheters (e.g., PleurX®) are allowed.

- Uncontrolled or symptomatic hypercalcemia ($>$1.5 mmol/L ionized calcium or calcium $>$12 mg/dL or corrected serum calcium $>$ULN)

General Medical Exclusions

- History of severe allergic, anaphylactic, or other hypersensitivity reactions to chimeric or humanized antibodies or fusion proteins
- Known hypersensitivity to biopharmaceuticals produced in Chinese hamster ovary cells or any component of the atezolizumab formulation
- History of autoimmune disease, including, but not limited to, myasthenia gravis, myositis, autoimmune hepatitis, systemic lupus erythematosus, rheumatoid arthritis, inflammatory bowel disease, vascular thrombosis associated with antiphospholipid syndrome, Wegener granulomatosis, Sjögren syndrome, Guillain-Barré syndrome, multiple sclerosis, vasculitis, or glomerulonephritis (see Appendix 13 for a more comprehensive list of autoimmune diseases)

    Patients with a history of autoimmune-related hypothyroidism on thyroid replacement therapy are eligible for this study.

    Patients with controlled Type I diabetes mellitus on an insulin regimen are eligible for this study.

    Patients with eczema, psoriasis, lichen simplex chronicus, or vitiligo with dermatologic manifestations only (e.g., patients with psoriatic arthritis would be excluded) are permitted provided that they meet the following conditions:

        Rash must cover less than 10% of body surface area.

        Disease is well controlled at baseline and only requiring low-potency topical steroids.

        No acute exacerbations of underlying condition within the last 12 months requiring treatment with PUVA [psoralen plus ultraviolet A radiation], methotrexate, retinoids, biologic agents, oral calcineurin inhibitors, or high-potency or oral steroids.

- History of idiopathic pulmonary fibrosis, organizing pneumonia (e.g., bronchiolitis obliterans), drug-induced pneumonitis, idiopathic pneumonitis, or evidence of active pneumonitis on screening chest CT scan

    History of radiation pneumonitis in the radiation field (fibrosis) is permitted.

- Current treatment with anti-viral therapy for HBV
- Active tuberculosis

"Use of this document is governed by the terms of use on the first page of this document."

- Severe infections within 4 weeks prior to randomization, including, but not limited to, hospitalization for complications of infection, bacteremia, or severe pneumonia
- Major surgical procedure other than for diagnosis within 28 days prior to randomization or anticipation of need for a major surgical procedure during the course of the study
- Prior allogeneic bone marrow transplantation or solid organ transplantation
- Any other diseases, metabolic dysfunction, physical examination finding, or clinical laboratory finding giving reasonable suspicion of a disease or condition that contraindicates the use of an investigational drug or that may affect the interpretation of the results or render the patient at high risk from treatment complications
- Patients with illnesses or conditions that interfere with their capacity to understand, follow, and/or comply with study procedures

<u>Exclusion Criteria Related to Medications</u>

- Any approved anti-cancer therapy, including hormonal therapy, within 3 weeks prior to initiation of study treatment
- Treatment with any other investigational agent with therapeutic intent within 28 days prior to randomization
- Receipt of therapeutic oral or IV antibiotics within 2 weeks prior to randomization

  Patients receiving prophylactic antibiotics (e.g., for prevention of a urinary tract infection or to prevent chronic obstructive pulmonary disease exacerbation) are eligible.
- Administration of a live, attenuated vaccine within 4 weeks before randomization or anticipation of need for such a vaccine during atezolizumab treatment or within 5 months after the last dose of atezolizumab
- Prior treatment with CD137 agonists or immune checkpoint blockade therapies, anti–PD-1, and anti–PD-L1 therapeutic antibodies

  Patients who have had prior anti–cytotoxic T lymphocyte–associated antigen 4 (CTLA-4) treatment may be enrolled, provided the following requirements are met:

    Last dose of anti–CTLA-4 at least 6 weeks prior to randomization

    No history of severe immune related adverse effects from anti–CTLA-4 (National Cancer Institute Common Terminology Criteria for Adverse Events [NCI CTCAE] Grade 3 and 4)
- Treatment with systemic immunostimulatory agents (including, but not limited to, interferons or interleukin-2) within 4 weeks or 5 half-lives of the drug, whichever is longer, prior to randomization

"Use of this document is governed by the terms of use on the first page of this document."

Prior treatment with cancer vaccines is allowed.

- Treatment with systemic corticosteroids or other systemic immunosuppressive medications (including, but not limited to, corticosteroids, cyclophosphamide, azathioprine, methotrexate, thalidomide, and anti–tumor necrosis factor [anti-TNF] agents) within 2 weeks prior to randomization

  Patients who received acute, low-dose, systemic immunosuppressant medication or a one-time pulse dose of systemic immunosuppressant medication (e.g., 48 hours of corticosteroids for a contrast allergy) are eligible for the study after Medical Monitor approval has been obtained.

  The use of corticosteroids ($\leq$10 mg oral prednisone or equivalent) for chronic obstructive pulmonary disease, mineralocorticoids (e.g., fludrocortisone) for patients with orthostatic hypotension, and low-dose supplemental corticosteroids for adrenocortical insufficiency are allowed.

Exclusion Criteria Related to Chemotherapy

- Hypersensitivity to the active substance or any of the excipients in pemetrexed, gemcitabine, carboplatin, cisplatin, or other platinum-containing compounds
- Patients with hearing impairment (cisplatin)
- Grade $\geq$2 peripheral neuropathy as defined by NCI CTCAE v4.0 criteria (cisplatin)
- CrCl $<$60 mL/min (cisplatin)
- History of radiation therapy within 7 days prior to initiating gemcitabine

13.4.2 Method of Treatment Assignment

This is an open-label study in regard to drug administration, though the Sponsor will remain blinded to treatment assignment and the associated data until primary analysis.

After written informed consent has been obtained and eligibility has been established (including determination of bTMB status by central testing), for patients who are eligible for enrollment, the study site will obtain the patient's randomization and treatment assignment from the interactive voice or Web-based response system (IxRS). Randomization to one of two treatment cohorts will occur in a 1:1 ratio.

Permuted-block randomization will be applied to ensure a balanced assignment to each treatment cohort. Randomization will be stratified by the following criteria:

- Biomarker cutoff (bTMB high vs. moderate)
- ECOG Performance Status (0 vs. 1)
- Tissue availability (yes vs. no)
- Histology (non-squamous vs. squamous)

"Use of this document is governed by the terms of use on the first page of this document."

Patients should receive their first dose of study treatment on the day of randomization. If this is not possible, the first dose should occur as soon as possible.

13.4.3 Study Treatment

Patients with non-squamous disease will receive either atezolizumab alone or pemetrexed in combination with cisplatin or carboplatin. Patients with squamous disease will receive either atezolizumab alone or gemcitabine in combination with cisplatin or carboplatin.

13.4.3.1 Study Treatment Formulation, Packaging, and Handling

Atezolizumab

The atezolizumab drug product is provided by the Sponsor as a sterile liquid in a single-use 20-mL glass vial. The vial is designed to deliver 20 mL (1200 mg) of atezolizumab-solution but may contain more than the stated volume to enable delivery of the entire 20-mL volume.

For further details on the formulation and handling of atezolizumab, see the Atezolizumab Investigator’s Brochure.

Cisplatin, Carboplatin, Pemetrexed, and Gemcitabine

Cisplatin, carboplatin, pemetrexed, and gemcitabine will be provided in commercially available formulations. For information on the formulation, packaging, and handling of cisplatin, carboplatin, pemetrexed, and gemcitabine, see the local prescribing information for each drug.

13.4.3.2 Study Treatment Dosage, Administration, and Compliance

Atezolizumab

Patients who are randomized to be treated with atezolizumab will receive 1200 mg atezolizumab administered by IV infusion every 21 days in a monitored setting where there is immediate access to trained personnel and adequate equipment/medicine to manage potentially serious reactions.

Atezolizumab infusions will be administered per the instructions outlined in Table C-1.

"Use of this document is governed by the terms of use on the first page of this document."

Table C-1 Administration of First and Subsequent Infusions of Atezolizumab

First Infusion	Subsequent Infusions
• No pre-medication is allowed.<br>• Record patient's vital signs (pulse rate, respiratory rate, blood pressure, and temperature) within 60 minutes before starting infusion.<br>• Infuse atezolizumab (1200 mg in a 250 mL 0.9% NaCl intravenous infusion bag) over 60 (± 15) minutes.<br>• If clinically indicated, record patient's vital signs (pulse rate, respiratory rate, blood pressure, and temperature) during the infusion at 15, 30, 45, and 60 minutes (± 5-minute windows are allowed for all timepoints).<br>• If clinically indicated, record patient's vital signs (pulse rate, respiratory rate, blood pressure, and temperature) at 30 (± 10) minutes after the infusion.<br>• Patients will be informed about the possibility of delayed post-infusion symptoms and instructed to contact their study physician if they develop such symptoms.	• If patient experienced infusion-related reaction during any previous infusion, pre-medication with antihistamines may be administered for Cycles ≥ 2 at the discretion of the treating physician.<br>• Record patient's vital signs (pulse rate, respiratory rate, blood pressure, and temperature) within 60 minutes before starting infusion.<br>• If the patient tolerated the first infusion well without infusion-associated adverse events, the second infusion may be delivered over 30 (± 10) minutes.<br>• If no reaction occurs, continue subsequent infusions over 30 (± 10) minutes.<br>Continue to record vital signs within 60 minutes before starting infusion. Record vital signs during and after the infusion if clinically indicated.<br>• If the patient had an infusion-related reaction during the previous infusion, the subsequent infusion must be delivered over 60 (± 15) minutes.<br>Record patient's vital signs (pulse rate, respiratory rate, blood pressure, and temperature) during the infusion if clinically indicated or if patient experienced symptoms during the previous infusion.<br>Record patient's vital signs (pulse rate, respiratory rate, blood pressure, and temperature) 30 (± 10) minutes after the infusion, if clinically indicated or if patient experienced symptoms during previous infusion.

NaCl = sodium chloride.

Dose modifications to atezolizumab are not permitted. Guidelines for treatment interruption or discontinuation and the management of specific adverse events are provided in Sections 13.5.1.6 and 13.5.1.7.

Refer to the Pharmacy Manual for detailed instructions on drug preparation, storage, and administration.

"Use of this document is governed by the terms of use on the first page of this document."

Pemetrexed in Combination with Cisplatin or Carboplatin (Patients with Non-Squamous NSCLC Only)

Each study site will administer pemetrexed (non-squamous NSCLC) in combination with platinum-based chemotherapy (cisplatin or carboplatin) for four or six cycles as per local standard of care. The intended number of chemotherapy induction cycles (four or six cycles) will be specified by the investigator prior to randomization. The selected platinum chemotherapy agent should remain the same for all cycles (e.g., patients who start on pemetrexed plus cisplatin should remain on this combination and not switch to pemetrexed plus carboplatin or vice versa). However, for patients who experience unacceptable toxicity with the selected platinum chemotherapy, a switch may be considered after discussion with and approval by the Medical Monitor.

Patients should receive steroid, folic acid, and vitamin B12 premedication for pemetrexed. The choice of steroid and timing of premedication can be administered according to the local standard of care and prescribing information (see Table C-2 and Section 13.5.1.8). Folic acid supplementation may be started before randomization in all patients at the discretion of the investigator to meet the local standard of care in anticipation for pemetrexed-based treatment and then discontinued in patients assigned to the atezolizumab cohort after randomization. In addition, patients should receive anti-emetic and IV hydration for platinum-based treatments according to the local standard of care and prescribing information.

Table C-2 lists the suggested premedication for the pemetrexed plus platinum-based chemotherapy and Table C-3 lists the doses and suggested infusion times for pemetrexed plus platinum-based chemotherapy. Chemotherapy infusion times may be adapted in accordance with local standard of care.

Table C-2 Premedication for Pemetrexed plus Platinum-Based Chemotherapy

Premedication	Dose/Route	Timing
Folic acid	350–1000 μg PO	Once daily beginning 5–7 days before Cycle 1, Day 1, and continuing until 3 weeks after discontinuation of pemetrexed or as per local standard of care.
Vitamin B12	1000 μg IM	Q9W beginning Cycle 1, Day 1, and continuing until 3 weeks after discontinuation of pemetrexed or as per local standard of care.
Dexamethasone (suggested)	4 mg PO	Twice daily the day prior to, the day of, and the day after each infusion of pemetrexed or as per local standard of care.

IM = intramuscular; PO = by mouth; Q9W = every 9 weeks.

Note: Prophylactic anti-emetics per local practice.

"Use of this document is governed by the terms of use on the first page of this document."

Table C-3 Treatment Regimen for Pemetrexed plus Platinum-Based Chemotherapy

Study Drug	Dose/Route	Induction Period (Four or Six Cycles)	Maintenance Period (Until PD)
Pemetrexed	500 mg/m^2 IV	Over ~10 minutes on Day 1 Q21D	Over approximately 10 minutes on Day 1 Q21D
Carboplatin	AUC 5 or 6 [a] IV	Over ~30–60 minutes on Day 1 Q21D	Not applicable
		OR	
Cisplatin	75 mg/m²	Over 1–2 hours on Day 1 Q21D	Not applicable

AUC = area under the concentration-time curve; PD = progressive disease; Q21D = every 21 days.
[a] See Section 13.4.3.2 for details on dose calculation of carboplatin.

Pemetrexed will be administered by IV infusion at a dose of 500 mg/m^2 on Day 1 of each 21-day cycle, followed by carboplatin or cisplatin at approximately 30 minutes after the completion of pemetrexed. Patients who do not experience disease progression per RECIST v1.1 after completing four or six cycles of induction treatment, will continue maintenance treatment with pemetrexed, given on Day 1 of each 21-day cycle until disease progression per RECIST v1.1. All patients eligible for pemetrexed therapy should avoid taking non-steroidal anti-inflammatory drugs (NSAIDs) for at least 2 days prior to pemetrexed administration if the NSAID has a short elimination half-life, for at least 5 days prior to pemetrexed administration if the NSAID has a long elimination half-life, on the day of pemetrexed administration, and at least 2 days following pemetrexed administration.

Guidelines for dose modification and treatment interruption or discontinuation are provided in Section 13.5.1.8 for pemetrexed, Section 13.5.1.11 for cisplatin, and Section 13.5.1.12 for carboplatin.

Gemcitabine in Combination with Cisplatin or Carboplatin (Patients with Squamous NSCLC Only)

Each study site will administer gemcitabine (squamous NSCLC) in combination with platinum-based chemotherapy (cisplatin or carboplatin) for four or six cycles as per local standard of care. The intended number of chemotherapy induction cycles (four or six cycles) will be specified by the investigator prior to randomization. The selected platinum chemotherapy agent should remain the same for all cycles (e.g., patients who start on gemcitabine plus cisplatin should remain on this combination and not switch to gemcitabine plus carboplatin or vice versa). However, for patients who experience unacceptable toxicity with the selected platinum chemotherapy, a switch may be considered after discussion with and approval by the Medical Monitor.

"Use of this document is governed by the terms of use on the first page of this document."

Patients should receive anti-emetic therapy and IV hydration for platinum-based treatments according to the local standard of care and prescribing information. Table C-4 lists the doses and the suggested infusion times for gemcitabine plus platinum-based treatments. Chemotherapy infusion times may be adapted in accordance with local standard of care.

Table C-4 Treatment Regimens for Gemcitabine plus Platinum-Based Chemotherapy

Chemotherapy	Dose/Route	Treatment (Four or Six Cycles)
Gemcitabine	1250 mg/m^2 IV	Over 30 minutes on Days 1 and 8 Q21D
Cisplatin	75 mg/m^2 IV	Over 1–2 hours on Day 1 Q21D
Gemcitabine	1000 mg/m^2 IV	Over 30 minutes on Days 1 and 8 Q21D
Carboplatin	AUC 5 IV	Over approximately 30–60 minutes on Day 1 Q21D

AUC = area under the concentration–time curve; IV = intravenous; Q21D = every 21 days.

Gemcitabine will be administered by IV infusion at a dose of 1250 mg/m^2 (in combination with cisplatin) or 1000 mg/m^2 (in combination with carboplatin) over 30 minutes on Days 1 and 8 of each 21-day cycle followed by cisplatin or carboplatin at approximately 30 minutes after the completion of gemcitabine infusion on Day 1 only.

Gemcitabine injection must be diluted prior to infusion. The recommended diluent for reconstitution of gemcitabine is 0.9% sodium chloride injection without preservatives.

The administration of gemcitabine should be done in accordance with local practice and the prescribing information; sites should follow their institutional standard of care for determining the gemcitabine dose for obese patients and for dose adjustment in the event of patient weight changes.

Guidelines for dose modification and treatment interruption or discontinuation are provided in Section 13.5.1.9 for gemcitabine, Section 13.5.1.11 for cisplatin, and Section 13.5.1.12 for carboplatin.

Cisplatin or Carboplatin Administration

Each site will choose to treat a given patient with either cisplatin or carboplatin according to local practice.

<u>Cisplatin</u>

Cisplatin should be administered by IV infusion approximately 30 minutes after completion of the pemetrexed or gemcitabine infusion at a dose of 75 mg/m^2 over

"Use of this document is governed by the terms of use on the first page of this document."

1–2 hours as per Table C-3 or Table C-4, respectively. Patients must receive adequate anti-emetic treatment and appropriate hydration prior to and/or after receiving cisplatin.

Refer to local clinical practice guidelines for further details.

Carboplatin

Carboplatin should be administered by IV infusion at a dose of area under the concentration–time curve (AUC) 5 or 6 when given in combination with pemetrexed (see Table C-3) or at a dose of AUC 5 when given in combination with gemcitabine (see Table C-4), after completion of the pemetrexed or gemcitabine infusion, with standard anti-emetics per local practice guidelines.

The carboplatin dose will be calculated using the Calvert formula (Calvert et al. 1989):

Calvert Formula

Total dose (mg) = (target AUC) × (glomerular filtration rate [GFR] + 25)

NOTE: The GFR used in the Calvert formula to calculate AUC-based dosing should not exceed 125 mL/min.

For the purposes of this protocol, the GFR is considered to be equivalent to the creatinine clearance (CrCl). The CrCl is calculated by institutional guidelines or by the method of Cockcroft and Gault (1976) using the following formula:

$$\text{CrCl} = \frac{(140 - \text{age})\ (\text{wt})}{72 \times \text{Scr}} \quad (\times\ 0.85 \text{ if female})$$

Where: CrCl = creatinine clearance in mL/min
age = patient's age in years
wt = patient's weight in kg
Scr = serum creatinine in mg/dL

NOTE: For patients with an abnormally low serum creatinine level, estimate GFR using a minimum creatinine level of 0.8 mg/dL or cap the estimated GFR at 125 mL/min.

If a patient's GFR is estimated on the basis of serum creatinine measurements by the isotope dilution mass spectroscopy method, the U.S. Food and Drug Administration (FDA) recommends that physicians consider capping the dose of carboplatin for desired exposure (AUC) to avoid potential toxicity caused by overdosing. On the basis of the Calvert formula described in the carboplatin label, the maximum doses can be calculated as follows:

Maximum carboplatin dose (mg) = target AUC (mg • min/mL) × (GFR + 25 mL/min)

"Use of this document is governed by the terms of use on the first page of this document."

The maximum dose is based on a GFR estimate that is capped at 150 mL/min for patients with normal renal function. No higher estimated GFR values should be used.

For a target AUC = 6, the maximum dose is $6 \times 150 = 900$ mg.
For a target AUC = 5, the maximum dose is $5 \times 150 = 750$ mg.
For a target AUC = 4, the maximum dose is $4 \times 150 = 600$ mg.

Refer to the FDA's communication regarding carboplatin dosing for more details at https://www.fda.gov/aboutfda/centersoffices/officeofmedicalproductsandtobacco/cder/ucm228974.htm.

13.4.4 Concomitant Therapy and Additional Restrictions

Concomitant therapy consists of any medication (e.g., prescription drugs, over-the-counter drugs, vaccines, herbal or homeopathic remedies, nutritional supplements) used by a patient in addition to protocol–mandated study treatment from 7 days prior to initiation of study treatment until the treatment discontinuation visit. All such medications should be reported to the investigator and recorded on the Concomitant Medications eCRF.

13.4.4.1 Permitted Therapy

Patients are permitted to use the following therapies during the study:

- Oral contraceptives
- Hormone-replacement therapy
- Prophylactic or therapeutic anticoagulation therapy (such as warfarin at a stable dose-level or low–molecular-weight heparin)
- Palliative radiotherapy (e.g., treatment of known bony metastases or symptomatic relief of pain) as outlined below:

  After the induction phase with chemotherapy (four or six cycles of pemetrexed or gemcitabine combined with a platinum agent) is complete, palliative radiotherapy is permitted, provided it does not interfere with the assessment of tumor target lesions (e.g., the lesion to be irradiated must not be the only site of measurable disease). Treatment with atezolizumab may be continued during palliative radiotherapy.
- Inactivated influenza vaccinations
- Megestrol administered as an appetite stimulant
- Corticosteroids administered for chronic obstructive pulmonary disease or asthma
- Mineralocorticoids (e.g., fludrocortisone)
- Low-dose corticosteroids administered for orthostatic hypotension or adrenocortical insufficiency

"Use of this document is governed by the terms of use on the first page of this document."

Premedication with antihistamines, antipyretics, and/or analgesics may be administered for the second and subsequent atezolizumab infusions only, at the discretion of the investigator.

In general, investigators should manage a patient's care with supportive therapies as clinically indicated, per local standard practice. Patients who experience infusion-associated symptoms may be treated symptomatically with acetaminophen, ibuprofen, diphenhydramine, and/or H_2-receptor antagonists (e.g., famotidine, cimetidine), or equivalent medications per standard practice (for sites outside the United States, equivalent medications may be substituted per local practice). Serious infusion-associated events manifested by dyspnea, hypotension, wheezing, bronchospasm, tachycardia, reduced oxygen saturation, or respiratory distress should be managed with supportive therapies as clinically indicated (e.g., supplemental oxygen and β_2-adrenergic agonists; see Appendix 14).

13.4.4.2 Cautionary Therapy for Atezolizumab-Treated Patients

Corticosteroids and Tumor Necrosis Factor–α Inhibitors

Systemic corticosteroids and TNF-α inhibitors may attenuate potential beneficial immunologic effects of treatment with atezolizumab. Therefore, in situations in which systemic corticosteroids or TNF-α inhibitors would be routinely administered, alternatives, including antihistamines, should be considered. If the alternatives are not feasible, systemic corticosteroids and TNF-α inhibitors may be administered at the discretion of the investigator.

Systemic corticosteroids are recommended, at the discretion of the investigator, for the treatment of specific adverse events when associated with atezolizumab therapy (refer to the Atezolizumab Investigator's Brochure for details).

Herbal Therapies

Concomitant use of herbal therapies is not recommended because their pharmacokinetics, safety profiles, and potential drug-drug interactions are generally unknown. However, herbal therapies not intended for the treatment of cancer (see Section 13.4.4.3) may be used during the study at the discretion of the investigator.

13.4.4.3 Prohibited Therapy

Use of the following medications is prohibited as described below:

- Concomitant therapy intended for the treatment of cancer (including, but not limited to, chemotherapy, hormonal therapy, immunotherapy, radiotherapy, and herbal therapy), whether health authority–approved or experimental, is prohibited for various time periods prior to starting study treatment, depending on the anti-cancer

"Use of this document is governed by the terms of use on the first page of this document."

agent (see Section 13.4.1.2), and during study treatment until disease progression is documented and the patient has discontinued study treatment.

- Investigational therapy (other than protocol-mandated study treatment) is prohibited within 28 days prior to initiation of study treatment and during study treatment.
- All patients eligible for pemetrexed therapy should avoid taking NSAIDs for at least 2 days prior to pemetrexed administration if the NSAID has a short elimination half-life; for at least 5 days prior to pemetrexed administration if the NSAID has a long elimination half-life; on the day of pemetrexed administration; and at least 2 days following pemetrexed administration.
- Live, attenuated vaccines (e.g., FluMist®) are prohibited within 4 weeks prior to initiation of study treatment with atezolizumab, during treatment with atezolizumab, and for 5 months after the final dose of atezolizumab.

13.4.5 Cohort Assessments

Vital Signs

For Cohort C, vital signs will be measured at Cycle 1 and at all subsequent cycles as follows:

**Cycle 1**	
Treatment Arm	Timepoints
Atezolizumab	• Within 60 minutes prior to atezolizumab infusion<br>• During the infusion (every 15 [±5] minutes) and within 30 (±10) minutes after atezolizumab infusion, if clinically indicated
Chemotherapy	• Within 60 minutes prior to pemetrexed or gemcitabine infusion<br>• As clinically indicated prior to, during, or after cisplatin or carboplatin infusion
**Subsequent Cycles**	
Treatment Arm	Timepoints
Atezolizumab or chemotherapy	• Within 60 minutes prior to infusion<br>• During the infusion if clinically indicated or if symptoms occurred during the prior infusion<br>• Within 30 (±10) minutes after infusion if clinically indicated or if symptoms occurred during the prior infusion

"Use of this document is governed by the terms of use on the first page of this document."

13.4.6 Study Treatment Discontinuation

Patients must discontinue study treatment if they fulfill any of the criteria in Section 4.6.1. However, there is an exception **for atezolizumab-treated patients only**: Patients will be permitted to continue study treatment after RECIST v1.1 criteria for disease progression are met if they meet all of the following criteria (see Figure C-2 below for schematic representation):

Evidence of clinical benefit as assessed by the investigator

- Absence of symptoms and signs (including worsening of laboratory values [e.g., new or worsening hypercalcemia]) indicating unequivocal progression of disease
- No decline in ECOG Performance Status that can be attributed to disease progression
- Absence of tumor progression at critical anatomical sites (e.g., leptomeningeal disease) that cannot be readily managed and stabilized by protocol-allowed medical interventions prior to repeat dosing
- Patients must provide written consent to acknowledge deferring other treatment options in favor of continuing atezolizumab treatment at the time of initial progression

"Use of this document is governed by the terms of use on the first page of this document."

Figure C-2 Criteria for Continuing Atezolizumab in the Presence of Increased Radiographic Tumor Size (Atezolizumab Arm Only)

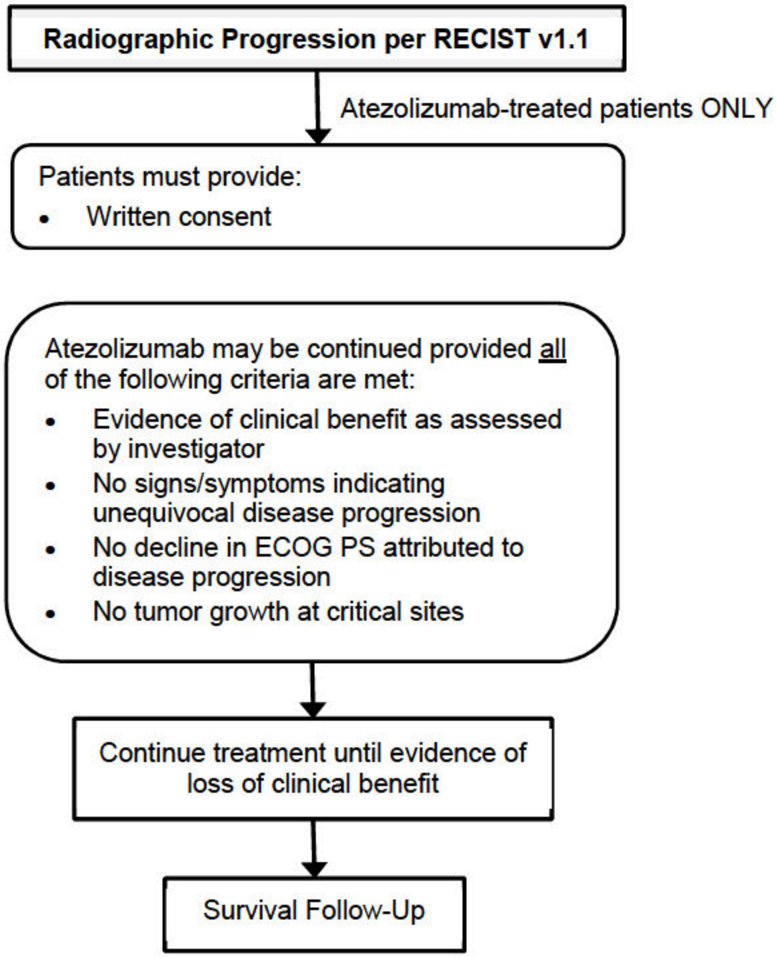

ECOG PS = Eastern Cooperative Oncology Group Performance Status; RECIST = Response Evaluation Criteria in Solid Tumors.

13.5 ASSESSMENT OF SAFETY: bTMB+ COHORT

Please refer to Section 5 for general instructions with regard to the assessment of safety in this study. Following are further instructions specific to the bTMB+ cohort.

Atezolizumab is approved in the United States for the treatment of metastatic NSCLC that has progressed on platinum-based chemotherapy. It is also approved for the treatment of locally advanced or metastatic urothelial carcinoma. Human experience is currently limited and the entire safety profile is not known at this time. The following

"Use of this document is governed by the terms of use on the first page of this document."

information is based on results from nonclinical and clinical studies and published data on similar molecules.

13.5.1 Safety Plan

Measures will be taken to ensure the safety of patients participating in this cohort, including the use of stringent inclusion and exclusion criteria (see Sections 13.4.1.1 and 13.4.1.2) and close monitoring (as indicated below and in Section 4.5).
See Sections 5.3 and 13.5.2 for complete details regarding safety reporting for this study.

Administration of atezolizumab will be performed in a monitored setting where there is immediate access to trained personnel and adequate equipment/medicine to manage potentially serious reactions. All serious adverse events and adverse events of special interest will be recorded during the study and for up to 90 days after the last dose of study treatment or initiation of new systemic anti-cancer therapy after the last dose of study treatment, whichever occurs first. All other adverse events will be recorded during the study and for up to 30 days after the last dose of study treatment or until the initiation of new systemic anti-cancer therapy after the last dose of study treatment, whichever occurs first.

Investigators are instructed to report all serious adverse events and adverse events of special interest considered related to study treatment regardless of time after study. The potential safety issues anticipated in this trial, as well as measures intended to avoid or minimize such toxicities, are outlined in the following sections.

13.5.1.1 Risks Associated with Atezolizumab

Atezolizumab has been associated with risks such as the following: IRRs and immune-mediated hepatitis, pneumonitis, colitis, pancreatitis, diabetes mellitus, hypothyroidism, hyperthyroidism, adrenal insufficiency, hypophysitis, Guillain-Barré syndrome, myasthenic syndrome or myasthenia gravis, meningoencephalitis, myocarditis, nephritis, myositis*, and severe cutaneous adverse reactions.* Immune-mediated reactions may involve any organ system and may lead to hemophagocytic lymphohistiocytosis *(HLH)* and macrophage activation syndrome *(MAS), which are considered to be potential risks for atezolizumab.* Refer to Appendix 15 of the protocol and Section 6 of the Atezolizumab Investigator's Brochure for a detailed description of anticipated safety risks for atezolizumab.

13.5.1.2 Risks Associated with Pemetrexed

The most common side-effects of pemetrexed include gastrointestinal symptoms (nausea, vomiting, diarrhea, or constipation), myelosuppression, infection, fatigue, stomatitis, loss of appetite, and rash.

"Use of this document is governed by the terms of use on the first page of this document."

For more details regarding the safety profile of pemetrexed, refer to the pemetrexed prescribing information.

13.5.1.3 Risks Associated with Gemcitabine

Gemcitabine infusion times that are longer than 60 minutes or gemcitabine administration that occurs more frequently than once a week have been shown to increase toxicity. Pulmonary toxicity has been reported with the use of gemcitabine. Myelosuppression manifested by neutropenia, thrombocytopenia, and anemia has been reported with gemcitabine as a single agent or in combination with other cytotoxic drugs. Hemolytic-uremic syndrome and/or renal failure have been reported following one or more doses of gemcitabine. Renal failure leading to death or requiring dialysis, despite discontinuation of therapy, has been rarely reported. Serious hepatotoxicity, including liver failure and death, has been reported very rarely in patients receiving gemcitabine alone or in combination with other potentially hepatotoxic drugs.

Patients will be monitored for gemcitabine-related adverse events. For more details regarding the safety profile of gemcitabine, refer to the gemcitabine prescribing information.

13.5.1.4 Risks Associated with Cisplatin

Cisplatin is known to cause myelosuppression, neurotoxicity, ototoxicity, and nephrotoxicity. Cisplatin-based chemotherapy is considered to be moderately emetogenic. Patients will be monitored for cisplatin-related adverse events.

For more details regarding the safety profile of cisplatin, refer to the cisplatin prescribing information.

13.5.1.5 Risks Associated with Carboplatin

Carboplatin is known to cause bone marrow suppression including myelosuppression, anemia, and thrombocytopenia. Carboplatin-based chemotherapy is considered to be moderately emetogenic. Patients will be monitored for carboplatin-related adverse events.

For more details regarding the safety profile of carboplatin, refer to the carboplatin prescribing information.

13.5.1.6 Dose Modifications

General Considerations

Reasons for dose modifications or delays, the supportive measures taken, and the outcomes will be documented in the patient's chart and recorded on the eCRF.

"Use of this document is governed by the terms of use on the first page of this document."

The severity of adverse events will be graded according to the NCI CTCAE v4.0 grading system.

- For any concomitant conditions already apparent at baseline, the dose modifications will apply according to the corresponding shift in toxicity grade, if the investigator considers it is appropriate. For example, if a patient has Grade 1 asthenia at baseline that increases to Grade 2 during study treatment, this will be considered a shift of one grade and treated as Grade 1 toxicity for dose-modification purposes.
- When several toxicities with different grades of severity occur at the same time, the dose modifications should be according to the highest grade observed.
- If, in the opinion of the investigator, a toxicity is considered to be due solely to one component of chemotherapy, the dose of the other chemotherapy component does not require modification and the other chemotherapy component(s) may be administered if there is no contraindication.
- The investigator may use discretion in modifying or accelerating the dose modification guidelines described below depending on the severity of toxicity and an assessment of the risk versus benefit for the patient, with the goal of maximizing patient compliance and access to supportive care.

Atezolizumab Dose Modifications, Treatment Delays, or Treatment Discontinuation and Management of Specific Adverse Events

Atezolizumab treatment may be temporarily suspended in patients experiencing toxicity considered to be related to study treatment. If corticosteroids are initiated for treatment of the toxicity, they must be tapered over ≥1 month to ≤10 mg/day oral prednisone or equivalent before atezolizumab can be resumed. If atezolizumab is withheld for >105 days after event onset, the patient will be discontinued from atezolizumab. However, atezolizumab may be withheld for >105 days to allow for patients to taper off corticosteroids prior to resuming treatment. Atezolizumab can be resumed after being withheld for >105 days if the Medical Monitor agrees that the patient is likely to derive clinical benefit. Atezolizumab treatment may be suspended for reasons other than toxicity (e.g. surgical procedures) with Medical Monitor approval. The investigator and the Medical Monitor will determine the acceptable length of treatment interruption.

Refer to the Atezolizumab Investigator's Brochure for more detailed information regarding dose modification.

13.5.1.7 Management of Atezolizumab-Specific Adverse Events

Refer to Appendix 15 for details on management of atezolizumab-specific adverse events. Refer to Appendix 14 for precautions for anaphylaxis.

"Use of this document is governed by the terms of use on the first page of this document."

13.5.1.8 Pemetrexed Dose Modifications, Treatment Delays, or Treatment Discontinuation and Management of Specific Adverse Events

The dose modification guidelines are applicable for pemetrexed used as a single agent or in combination with cisplatin or carboplatin.

Treatment with pemetrexed should be discontinued if a patient experiences any hematologic or non-hematologic Grade 3 or 4 toxicity after two dose reductions, or if treatment is delayed for more than 63 days due to toxicities.

Hematologic Toxicity

At the start of each cycle, the ANC must be ≥ 1500/μL and the platelet count must be ≥ 100,000/μL. Treatment should be delayed for up to 63 days to allow sufficient time for recovery. Growth factors may be used in accordance with American Society of Clinical Oncology (ASCO) and National Comprehensive Cancer Network (NCCN) guidelines (Smith et al. 2015; NCCN 2016). Upon recovery, dose adjustments at the start of a subsequent cycle will be made on the basis of the lowest (nadir) platelet and neutrophil values from the previous cycle (see Table C-5).

In the event that dose adjustments are needed for both ANC and platelets, patients are to receive the lower dose.

Table C-5 Chemotherapy Dose Modifications for Hematologic Toxicities

Toxicity [a]	Chemotherapy Dose
ANC < 500/μL and platelets ≥ 50,000/μL	75% of previous dose
Platelets < 50,000/μL, regardless of ANC	75% of previous dose
Platelets < 50,000/μL with Grade ≥ 2 bleeding, regardless of ANC	50% of previous dose

[a] Nadir of prior cycle.

Investigators should be vigilant and alert to early and overt signs of myelosuppression, infection, or febrile neutropenia so that these complications can be promptly and appropriately managed. Patients should be made aware of these signs and encouraged to seek medical attention at the earliest opportunity.

If chemotherapy must be withheld because of hematologic toxicity, full blood counts (including differential WBC) should be obtained weekly until the counts reach the lower limits for treatment as outlined. The treatment can then be resumed.

No dose reductions are recommended for anemia. Patients should be supported per institutional guidelines.

"Use of this document is governed by the terms of use on the first page of this document."

Non-Hematologic Toxicity

At the start of each cycle, the CrCl must be ≥45 mL/min. For enrollment and dosing decisions, CrCl will be estimated using the original, weight-based Cockcroft and Gault formula (1976) or measured using the appropriate radiolabeled method (51-CrEDTA or Tc99m-DTPA) to determine the GFR. The method of CrCl assessment used at baseline should be used throughout the study.

If a patient develops a non-hematologic toxicity (Table C-6), pemetrexed should be withheld for up to 63 days until resolution to equal or less than the patient's baseline (or Grade 1 or better if patient did not have that toxicity at baseline). Treatment should be resumed according to the guidelines in Table C-6. For Grade 3 or 4 neurotoxicity, pemetrexed should be resumed at 50% of the previous dose upon improvement, or discontinued immediately (based on investigator's clinical judgment).

Table C-6 Pemetrexed Dose Modifications for Non-Hematologic Toxicities

Toxicity	Pemetrexed Dose
Any diarrhea requiring hospitalization (irrespective of grade) or Grade 3 or 4 diarrhea that occurs on adequate anti-diarrhea medication.	75% of previous dose
Neurotoxicity	
Grade 2	75% of previous dose
Grade 3 or 4	50% of previous dose or permanent discontinuation
Any other Grade 3 or 4 toxicities	75% of previous dose

Treatment Delays Caused by Insufficient Folic Acid or Vitamin B12 Supplementation

Cycle 1 should not be started until both of the following requirements are met:

- The patient has taken folic acid for 5 to 7 days preceding the first dose of pemetrexed or as per local standard of care, but not later than Cycle 1, Day 1.
- The patient has received a Vitamin B12 injection (which can be given on Cycle 1, Day 1).

Delay subsequent cycles until the patient has taken folic acid for at least 14 of the 21 days before Day 1 of the subsequent cycle.

For more details regarding pemetrexed dose modification, refer to the pemetrexed prescribing information.

"Use of this document is governed by the terms of use on the first page of this document."

13.5.1.9 Gemcitabine Dose Modifications, Treatment Delays, or Treatment Discontinuation and Management of Specific Adverse Events

The dose modification guidelines for gemcitabine are provided below.

Treatment with gemcitabine should be discontinued if a patient experiences any hematologic or non-hematologic Grade 3 or 4 toxicity after two dose reductions, or if treatment is delayed for more than 63 days due to toxicities.

Hematologic Toxicities

Gemcitabine dose modifications for hematologic toxicity should be based on the granulocyte and platelet counts taken on Days 1 and 8 of treatment (Table C-7 and Table C-8). Patients receiving gemcitabine should be monitored prior to each dose with a full blood count, including differential and platelet counts. Treatment should be delayed for up to 63 days to allow sufficient time for recovery. Growth factors may be used in accordance with ASCO and NCCN guidelines (Smith et al. 2015; NCCN 2016). Upon recovery, dose adjustments at the start of a subsequent cycle will be made on the basis of the lowest (nadir) platelet and neutrophil values from the previous cycle (see Table C-5).

In the event that dose adjustments are needed for both ANC and platelets, patients are to receive the lower dose.

Table C-7 Gemcitabine Dose Modifications or Treatment Delays for Hematologic Toxicities on Day 1

Absolute Granulocyte Count ($\times 10^6$/L)		Platelet Count ($\times 10^6$/L)	Gemcitabine % of Full Dose
$\geq$1500	and	$\geq$100,000	100%
$<$1500	or	$<$100,000	Withhold

Table C-8 Gemcitabine Dose Modifications or Treatment Delays for Hematologic Toxicities on Day 8

Absolute Granulocyte Count		Platelet Count	Gemcitabine % of Full Dose
$\geq$1000/μL	And	$\geq$100,000/μL	100%
500–999/μL	Or	50,000–99,999/μL	75%
$<$500/μL	Or	$<$50,000/μL	Withhold

"Use of this document is governed by the terms of use on the first page of this document."

Investigators should be vigilant and alert to early and overt signs of myelosuppression, infection, or febrile neutropenia so that these complications can be promptly and appropriately managed. Patients should be made aware of these signs and encouraged to seek medical attention at the earliest opportunity.

If chemotherapy must be withheld because of hematologic toxicity, full blood counts (including differential WBC) should be obtained weekly until the counts reach the lower limits for treatment as outlined. The treatment can then be resumed.

No dose reductions are recommended for anemia. Patients should be supported per the treating physician's institution's guidelines.

13.5.1.10 Non-Hematologic Toxicities

In general, for Grade 3 or 4 non-hematologic toxicities, gemcitabine should be withheld or dose reduced by 50%, according to investigator's clinical judgment.

Permanent discontinuation should be considered for any of the following events:

- Unexplained dyspnea or other evidence of severe pulmonary toxicity
- Severe hepatic toxicity
- Hemolytic-uremic syndrome
- Capillary-leak syndrome
- Posterior reversible encephalopathy syndrome

Table C-9 provides dose modification guidelines for non-hematologic toxicities.

"Use of this document is governed by the terms of use on the first page of this document."

Table C-9 Gemcitabine Dose Modifications, Treatment Delays, or Treatment Discontinuation and Patient Management for Grade 2, 3, or 4 Non-Hematologic Toxicities

	Grade 2	Grade 3	Grade 4
First appearance	Interrupt treatment until resolved to Grade 0–1, then continue at same dose with prophylaxis where possible.	Interrupt treatment until resolved to Grade 0–1, then continue at 75% of original dose with prophylaxis where possible.	Discontinue treatment unless considered it to be in the best interest of the patient to continue at 50% of original dose, once toxicity has resolved to Grade 0–1.
Second appearance of same toxicity	Interrupt treatment until resolved to Grade 0–1, then continue at 75% of original dose.	Interrupt treatment until resolved to Grade 0–1, then continue at 50% of original dose.	
Third appearance of same toxicity	Interrupt treatment until resolved to Grade 0–1, then continue at 50% of original dose.	Discontinue treatment permanently.	
Fourth appearance of same toxicity	Discontinue treatment permanently.		

13.5.1.11 Cisplatin Dose Modifications, Treatment Delays, or Treatment Discontinuation and Management of Specific Adverse Events

The dose modification guidelines for cisplatin are provided below.

Treatment with cisplatin should be discontinued if a patient experiences any hematologic or non-hematologic Grade 3 or 4 toxicity after two dose reductions or treatment is delayed for more than 63 days due to toxicities.

Hematologic Toxicities

At the start of each cycle, the ANC must be ≥ 1500/μL and the platelet count must be ≥ 100,000/μL. Treatment should be delayed for up to 63 days to allow sufficient time for recovery. Growth factors may be used in accordance with ASCO and NCCN guidelines (Smith et al. 2015; NCCN 2016). Upon recovery, dose adjustments at the start of a subsequent cycle will be made on the basis of the lowest platelet and neutrophil values from the previous cycle (see Table C-10).

In the event that dose adjustments are needed for both ANC and platelets, patients are to receive the lower dose.

"Use of this document is governed by the terms of use on the first page of this document."

Table C-10 Cisplatin Dose Modifications for Hematologic Toxicities

Toxicity [a]	Cisplatin Dose
ANC $<$ 500/µL and platelets $\geq$ 50,000/µL	75% of previous dose
Platelets $<$ 50,000/µL, regardless of ANC	75% of previous dose
Platelets $<$ 50,000/µL with Grade $\geq$ 2 bleeding, regardless of ANC	50% of previous dose
ANC $<$ 1000/µL plus fever of $\geq$ 38.5°C	75% of previous dose

[a] Nadir of prior cycle.

Investigators should be vigilant and alert to early and overt signs of myelosuppression, infection, or febrile neutropenia so that these complications can be promptly and appropriately managed. Patients should be made aware of these signs and encouraged to seek medical attention at the earliest opportunity.

If chemotherapy must be withheld because of hematologic toxicity, full blood counts (including differential WBC) should be obtained weekly until the counts reach the lower limits for treatment as outlined. The treatment can then be resumed.

No dose reductions are recommended for anemia. Patients should be supported per institutional guidelines.

Non-Hematologic Toxicities

If a patient develops a non-hematologic toxicity (see Table C-11), cisplatin should be withheld for up to 63 days until resolution to less than or equal to the patient's baseline (or Grade $\leq$ 1 if patient did not have that toxicity at baseline). Treatment should be resumed according to the guidelines in Table C-11.

Diarrhea should be controlled with adequate anti-diarrhea medication. Nausea and/or vomiting should be controlled with adequate anti-emetics.

"Use of this document is governed by the terms of use on the first page of this document."

Table C-11 Cisplatin Dose Modifications for Non-Hematologic Toxicities (Excluding Neurotoxicity)

Toxicity	Cisplatin Dose
Any diarrhea requiring hospitalization (irrespective of grade) or Grade 3 or 4 diarrhea that occurs on adequate anti-diarrhea medication	75% of previous dose
Grade 3 or 4 nausea/vomiting [a]	75% of previous dose
Any other Grade 3 or 4 toxicity	75% of previous dose

[a] Despite the use of anti-emetics.

Nephrotoxicity

CrCl must be ≥60 mL/min prior to the start of any cycle of cisplatin. If there is a decrease in CrCl between cycles, but the CrCl is still ≥60 mL/min at the time of the next cycle, the investigator should use clinical judgment regarding continuing cisplatin, dose reduction, or delaying the cycle. If a patient's CrCl value has not returned to ≥60 mL/min within 63days following last cisplatin administration, the patient should be discontinued from cisplatin.

Neurotoxicity

In the event of neurotoxicity, the recommended dose adjustment for cisplatin is documented in Table C-12. For Grade 3 or 4 neurotoxicity, cisplatin should be resumed at 50% of the previous dose upon improvement, or discontinued immediately (based on investigator's clinical judgment).

Table C-12 Cisplatin Dose Modifications or Treatment Discontinuation for Associated Neurotoxicity

Toxicity	Cisplatin Dose
Grade 0–1 neurotoxicity	100% of previous dose
Grade 2 neurotoxicity	75% of previous dose
Grade 3 or 4 neurotoxicity	50% of previous dose or permanent discontinuation

If the patient develops ototoxicity, subsequent doses of cisplatin should not be given until an audiometric analysis indicates that auditory acuity is within normal limits (http://www.drugs.com/pro/platinol.html). See Table C-12 for dose modifications.

13.5.1.12 Carboplatin Dose Modifications, Treatment Delays, or Treatment Discontinuation and Management of Specific Adverse Events

The dose modification guidelines for carboplatin are provided below.

"Use of this document is governed by the terms of use on the first page of this document."

Treatment with carboplatin should be discontinued if a patient experiences any hematologic or non-hematologic Grade 3 or Grade 4 toxicity after two dose reductions or treatment is delayed for more than 63 days due to toxicities.

Hematologic Toxicities

At the start of each cycle, the ANC must be ≥ 1500/μL and the platelet count must be ≥ 100,000/μL. Treatment should be delayed for up to 63 days to allow sufficient time for recovery. Growth factors may be used in accordance with ASCO and NCCN guidelines (Smith et al. 2015; NCCN 2016). Upon recovery, dose adjustments at the start of a subsequent cycle will be made on the basis of the lowest platelet and neutrophil values from the previous cycle (see Table C-13).

In the event that dose adjustments are needed for both ANC and platelets, patients are to receive the lower dose.

Table C-13 Carboplatin Dose Modifications for Hematologic Toxicities

Toxicity [a]	Carboplatin Dose
ANC < 500/μL and platelets ≥ 50,000/μL	75% of previous dose
Platelets < 50,000/μL, regardless of ANC	75% of previous dose
Platelets < 50,000/μL with Grade ≥ 2 bleeding, regardless of ANC	50% of previous dose
ANC < 1000/μL plus fever of ≥ 38.5°C	75% of previous dose

[a] Nadir of prior cycle.

Investigators should be vigilant and alert to early and overt signs of myelosuppression, infection, or febrile neutropenia so that these complications can be promptly and appropriately managed. Patients should be made aware of these signs and encouraged to seek medical attention at the earliest opportunity.

If chemotherapy must be withheld because of hematologic toxicity, full blood counts (including differential WBC) should be obtained weekly until the counts reach the lower limits for treatment as outlined. The treatment can then be resumed.

No dose reductions are recommended for anemia. Patients should be supported per the treating physician's institution's guidelines.

Non-Hematologic Toxicities

For a non-hematologic toxicity (see Table C-14), treatment should be delayed for up to 63 days until resolution to less than or equal to the patient's baseline value (or Grade ≤ 1 if patient did not have that toxicity at baseline). Dose reductions at the start of the subsequent cycle will be made on the basis of non-hematologic toxicities from the dose

"Use of this document is governed by the terms of use on the first page of this document."

administered in the preceding cycle. Table C-14 provides the dose modifications for non-hematologic toxicities.

Table C-14 Carboplatin Dose Modifications or Treatment Discontinuation for Non-Hematologic Toxicities

Toxicity		Adjusted Carboplatin Dose as % of Previous Dose [a]
Diarrhea	Grade 3 or 4 [b]	75%
Nausea/vomiting	Grade 3 or 4 [c]	75%
Neurotoxicity	Grade 2	75%
	Grade 3 or 4	50% or permanent discontinuation
Transaminase elevation	Grade 3	75%
	Grade 4	Discontinue
Other	Grade 3 or 4	75%

AUC = area under the concentration-time curve.

[a] If deemed appropriate by the investigator, adjust carboplatin dose to the specified percentage of the previous AUC.

[b] Grade 3 or 4 diarrhea that occurs on adequate anti-diarrhea medication or any grade of diarrhea requiring hospitalization.

[c] Despite the use of anti-emetics.

Diarrhea should be controlled with adequate anti-diarrhea medication. Nausea and/or vomiting should be controlled with adequate anti-emetics. For Grade 3 or 4 neurotoxicity, carboplatin should be resumed at 50% of the previous dose upon improvement or discontinued immediately (based on investigator's clinical judgment).

13.5.2 Safety Parameters and Definitions

13.5.2.1 Adverse Events of Special Interest (Immediately Reportable to the Sponsor)

Section 5.2.3 describes adverse events of special interest that are required to be reported by the investigator to the Sponsor immediately in this study, and Section 5.4.2 provides reporting instructions. Additional adverse events of special interest that are required to be reported by the investigator to the Sponsor immediately for this cohort include the following confirmed treatment-emergent autoimmune conditions:

- Pneumonitis
- Colitis
- Endocrinopathies: diabetes mellitus, pancreatitis, adrenal insufficiency, hyperthyroidism, and hypophysitis

"Use of this document is governed by the terms of use on the first page of this document."

- Hepatitis, including AST or ALT $> 10 \times$ ULN
- Systemic lupus erythematosus
- Neurological disorders: Guillain-Barré syndrome, myasthenic syndrome or myasthenia gravis, and meningoencephalitis
- Events suggestive of hypersensitivity, infusion-related reactions, cytokine-release syndrome, influenza-like illness, and systemic inflammatory response syndrome
- Nephritis
- Ocular toxicities (e.g., uveitis, retinitis)
- Myositis
- Myopathies, including rhabdomyolysis
- Grade $\geq$2 cardiac disorders (e.g., atrial fibrillation, myocarditis, pericarditis)
- Vasculitis

13.5.2.2 Adverse Event Reporting Period

See Section 5.3.1 for general guidance regarding the adverse event reporting period.

With regard to event reporting **after initiation of study drug** in this treatment cohort, all serious adverse events and adverse events of special interest, regardless of relationship to study drug, will be reported until 90 days after the last dose of study drug or initiation of new anti-cancer therapy, whichever occurs first.

13.6 STATISTICAL CONSIDERATIONS: bTMB+ COHORT

This is a randomized, Phase III, open-label cohort designed to evaluate the safety and efficacy of atezolizumab compared with chemotherapy consisting of a platinum agent (cisplatin or carboplatin per investigator discretion) combined with either pemetrexed (non-squamous NSCLC) or gemcitabine (squamous NSCLC).

There are two populations defined in this cohort:

- bTMB PP1: the primary population of patients with a bTMB level equal to or greater than the higher validated cutoff
- bTMB PP2: the secondary population of all patients who are bTMB+, which is the intent-to-treat (ITT) population in this cohort

Approximately 440 patients will be randomized into bTMB PP2 in the global enrollment phase of this cohort, with approximately 280 patients randomized into bTMB PP1.

The analysis of PFS and OS will be performed for bTMB PP1 and bTMB PP2, with patients grouped according to the treatment assigned at randomization, regardless of whether they received any assigned study treatment. Objective response rate (ORR)

"Use of this document is governed by the terms of use on the first page of this document."

will be analyzed using all randomized patients who have measurable disease at baseline in bTMB PP1 and/or bTMB PP2 where statistical significance of PFS will be declared. Duration of response (DOR) will be assessed in patients who have an objective response. Time to deterioration (TTD) analyses for PROs will be conducted on all randomized patients. Change-from-baseline analyses for PROs will be performed using patients who have both a non-missing baseline assessment and at least one post-baseline assessment. Safety analyses will be performed on all randomized patients who received any amount of any components of protocol treatment, with patients grouped according to whether any full or partial dose of atezolizumab was received.

The overall type I error rate for this cohort is strictly controlled at 2.5% (one-sided), which allows the trial to declare positivity in PFS and/or OS in bTMB PP1 and/or bTMB PP2 (see Figure C-3). Details of the analyses will be provided in the Statistical Analysis Plan (SAP).

13.6.1 Determination of Sample Size

This study will randomize approximately 280 patients in bTMB PP1, which will result in the randomization of approximately 440 patients in bTMB PP2 (including the 280 patients in bTMB PP1).

The comparison of PFS and OS analysis hierarchy is described below and shown in Figure C-3:

1. PFS in bTMB PP1 will be tested at $\alpha = 0.025$ (one-sided).
2. If PFS results for bTMB PP1 are statistically significant, then OS in bTMB PP1 will be tested at $\alpha = 0.025$ (one-sided).
3. If the OS results are statistically significant in bTMB PP1, then PFS in bTMB PP2 will be tested at $\alpha = 0.025$ (one-sided).
4. If the PFS results in bTMB PP2 are statistically significant, then OS in bTMB PP2 will be tested at $\alpha = 0.025$ (one-sided).

"Use of this document is governed by the terms of use on the first page of this document."

Figure C-3 Progression-Free Survival and Overall Survival Analysis Hierarchy

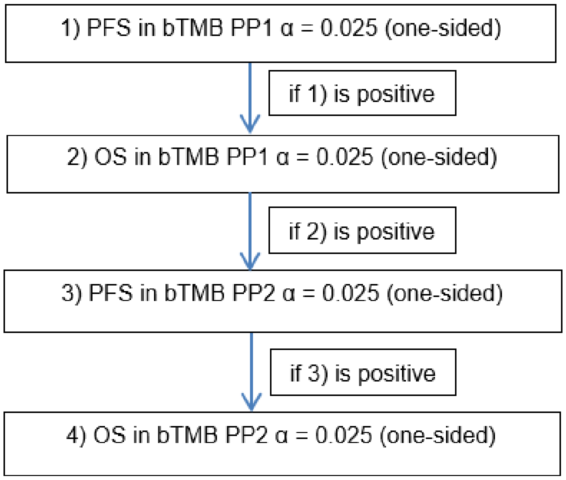

The sample size determination for bTMB PP1 and bTMB PP2 is based on the number of events required to demonstrate efficacy with regard to PFS. The estimate of the number of PFS events required is based on the following assumptions:

- One-sided significance level of 0.025 for the comparison in bTMB PP1
- One-sided significance level of 0.025 for the comparison in bTMB PP2
- Approximately 95% power to detect an HR of 0.6, corresponding to an improvement in median PFS from 6 months to 10 months in bTMB PP1
- Approximately 98% power to detect an HR of 0.65, corresponding to an improvement in median PFS from 6 months to 9.2 months in bTMB PP2
- No interim analysis of PFS
- Event times exponentially distributed
- Accrual duration is approximately 25 months
- Dropout rate is 5% per 12 months for each treatment arm

With these assumptions, approximately 440 patients in total will be randomized into the bTMB+ cohort, with approximately 280 patients in bTMB PP1. A total of approximately 198 PFS events are required for the PFS primary comparison for bTMB PP1. This number of events corresponds to a minimum detectable difference in HR of approximately 0.756. The primary analysis of PFS (as defined in Section 13.6.2.1) will occur when approximately 198 investigator-assessed PFS events have occurred in patients in bTMB PP1 or at least 6 months after the last patient in bTMB PP1 has been

"Use of this document is governed by the terms of use on the first page of this document."

enrolled, whichever occurs later. This number of events is expected to be reached approximately 32 months after the first patient is randomized in bTMB PP1. At the time of the PFS primary analysis in bTMB PP1, approximately 338 PFS events will have occurred in bTMB PP2, with a corresponding minimal detectable HR of approximately 0.808.

OS will be tested using the group sequential method at the interim and final OS analyses. Table C-15 provides the summary of the OS interim and final analysis timing in bTMB PP1 and bTMB PP2 populations.

Table C-15 Summary of PFS and OS Analysis Timing in the bTMB PP1 and bTMB PP2 Populations

Analysis	Endpoints	Expected Timing of Analysis	Events Expected at Time of Analysis	
			bTMB PP1	bTMB PP2
Final PFS analysis	PFS	~32 months from FPI	~198 total PFS (~128 OS) events in both atezolizumab and chemo arms	~338 total PFS (~229 OS) events in both atezolizumab and chemo arms
Interim OS analysis	OS			
Final OS analysis	OS	~46 months from FPI	~190 total OS events in both the atezolizumab and chemo arms	~318 total OS events in both the atezolizumab and chemo arms

bTMB PP1 = the primary population of patients with a bTMB level equal to or greater than the higher validated cutoff; bTMB PP2 = the secondary population of all patients who are bTMB+, which is the intent-to-treat population in this cohort; FPI = first patient in; OS = overall survival.

With the target sample size in bTMB PP1 and bTMB PP2 and the expected timing of PFS final analysis, the following assumptions would lead to the estimate of the number of OS events with associated power of approximately 80% in bTMB PP1 and 85% in bTMB PP2, respectively:

- One-sided significance level of 0.025 for the comparison in bTMB PP1
- One-sided significance level of 0.025 for the comparison in bTMB PP2
- Approximately 80% power to detect an HR of 0.65, corresponding to an improvement in median OS from 14 months to 21.5 months in bTMB PP1
- Approximately 85% power to detect an HR of 0.7, corresponding to an improvement in median OS from 14 months to 20 months in bTMB PP2
- One OS interim analysis performed when approximately 67% of the total OS events required for the final analysis in bTMB PP1 have occurred using the Lan-DeMets approximation to the Pocock boundary

"Use of this document is governed by the terms of use on the first page of this document."

- One OS interim analysis performed when approximately 72% of the total OS events required for the final analysis in bTMB PP2 have occurred using the Lan-DeMets approximation to the Pocock boundary
- Event times exponentially distributed
- Accrual duration is approximately 25 months
- Dropout rate is 5% per 24 months for each treatment arm

The stopping boundaries at the interim and final analyses of OS are described in Section 13.6.3.

13.6.2 Efficacy Analyses

13.6.2.1 Primary Efficacy Endpoint and Hypothesis Testing

The primary efficacy endpoint is PFS assessed by the investigator using RECIST v1.1 in the bTMB PP1 population.

PFS is defined as the time from the date of randomization to the date of first documented disease progression or death, whichever occurs earlier. Disease progression will be determined on the basis of investigator assessment with use of RECIST v1.1. Data for patients who have not experienced disease progression or death at the time of analysis will be censored at the time of the last tumor assessment. Patients with no post-baseline tumor assessment will be censored at the date of randomization plus 1 day. Type I error control will be applied to this analysis of PFS.

For U.S. registrational purposes, a sensitivity analysis of PFS will be defined as described above with an additional censoring rule for missed visits. Data for patients with a PFS event who missed two or more scheduled assessments immediately prior to the PFS event will be censored at the last tumor assessment prior to the missed visits.

The following analyses will be performed for both PFS endpoints described above and secondary efficacy endpoints to be hierarchically tested (OS in bTMB PP1, PFS assessed by the investigator in bTMB PP2, OS in bTMB PP2; see Section 13.6.2.2). PFS and OS will be compared between treatment arms with the use of the stratified log-rank test. The HR for PFS and OS will be estimated using a stratified Cox regression model. The 95% CIs for the HRs will be provided.

The hypothesis testing will be performed in the order described below:

1. First, PFS will be tested at a one-sided significance level of 0.025 for bTMB PP1. If the estimate of the HR is <1 and the one-sided p-value corresponding to the stratified log-rank test is <0.025, the null hypothesis will be rejected, and it will be concluded that atezolizumab prolongs the duration of PFS relative to the control arm in bTMB PP1.

"Use of this document is governed by the terms of use on the first page of this document."

2. If the hypothesis in (1) is rejected, then OS will be tested at a one-sided significance level of 0.025 in bTMB PP2. If the estimate of the HR is < 1 and the one-sided p-value corresponding to the stratified log-rank test is < 0.025, the null hypothesis will be rejected, and it will be concluded that atezolizumab prolongs the duration of OS relative to the control arm in bTMB PP1.
3. If the hypotheses in (2) is rejected, then PFS will be tested at a one-sided significance level of 0.025 in bTMB PP2. If the estimate of the HR is < 1 and the one-sided p-value corresponding to the stratified log-rank test is < 0.025, the null hypothesis will be rejected, and it will be concluded that atezolizumab prolongs the duration of PFS relative to the control arm in bTMB PP2.
4. If the hypothesis in (3) is rejected, then OS will be tested at a one-sided significance level of 0.025 in bTMB PP2. If the estimate of the HR is < 1 and the one-sided p-value corresponding to the stratified log-rank test is less than the corresponding type I error rate, the null hypothesis will be rejected, and it will be concluded that atezolizumab prolongs the duration of OS relative to the control arm in bTMB PP2.

In stratified analyses, the stratification factors will be those used during randomization: specifically, for PP1 population, ECOG Performance Status (0 vs. 1), histology (non-squamous vs. squamous), and tissue availability (yes vs. no) as recorded in IxRS; for PP2 population, bTMB cutoff (high vs. moderate), ECOG Performance Status (0-vs.-1), histology (non-squamous vs. squamous), and tissue availability (yes vs. no) as recorded in IxRS. Both stratified and unstratified analysis will be performed. In case the number of patients is too small in some strata, patients in these strata may be pooled for analysis according to a pre-specified method to be presented in the Statistical Analysis Plan (SAP). Kaplan-Meier methodology will be used to estimate the median PFS for each treatment arm and to construct survival curves for the visual description of the difference between the treatment arms. The Brookmeyer-Crowley methodology (Brookmeyer and Crowley 1982) will be used to construct the 95% CI for the median PFS for each treatment arm.

13.6.2.2 Secondary Efficacy Endpoints

Overall Survival in bTMB PP1 Population

OS is defined as the time from the date of randomization to the date of death due to any cause. Data for patients who are not reported as having died at the time of analysis will be censored at the date when they were last known to be alive. Data for patients who do not have post-baseline information will be censored at the date of randomization plus 1 day.

See Section 13.6.2.1 for details on hypothesis testing for OS in bTMB PP1 population. OS will be analyzed through use of the same methods described for the PFS analysis in the bTMB PP1 population (see Section 13.6.2.1).

"Use of this document is governed by the terms of use on the first page of this document."

Progression-Free Survival in bTMB PP2 Population

See Section 13.6.2.1 for details on the definition and hypothesis testing for PFS in bTMB PP2 population.

Overall Survival in bTMB PP2 Population

See Section 13.6.2.1 for details on hypothesis testing for OS in bTMB PP2 population. OS will be analyzed through use of the same methods described for the PFS analysis in the bTMB PP1 population (see Section 13.6.2.1).

Progression-Free Survival as Assessed by the Independent Review Facility

To support the primary analysis of investigator-assessed PFS, the analysis of PFS as assessed by the IRF will be performed. The methodologies outlined for the primary analysis of PFS per the investigator will be used for the analyses of PFS based on IRF assessment.

Objective Response Rate as Assessed by the Investigator

An objective response is defined as either a CR or PR, as determined by the investigator with use of RECIST v1.1. Confirmation of objective response is required (confirmed ≥4 weeks apart in two separate tumor assessments). Patients not meeting these criteria, including patients without any postbaseline tumor assessments, will be considered non-responders.

ORR is defined as the proportion of patients who have a confirmed objective response. The analysis population for ORR will be all randomized patients with measurable disease at baseline. An estimate of ORR and its 95% CI will be calculated using the Clopper-Pearson method for each treatment cohort. CIs for the difference in ORRs between the two treatment arms will be determined using the normal approximation to the binomial distribution. The ORR will be compared between the two treatment arms using the stratified Cochran-Mantel-Haenszel test, stratified by the same factors used in the primary PFS analysis.

Objective Response Rate as Assessed by the Independent Review Facility

The methodologies outlined for the analysis of ORR per the investigator will be used for the analyses of ORR based on IRF assessment.

Duration of Response as Assessed by the Investigator

DOR is defined as the period measured from the date of the first occurrence of a confirmed CR or PR (whichever status is recorded first) until the first date that progressive disease or death is documented, whichever occurs first. Disease progression will be determined on the basis of investigator assessment with use of RECIST v1.1. DOR will be assessed in patients who had a confirmed objective response during the study as determined by the investigator with use of RECIST v1.1.

"Use of this document is governed by the terms of use on the first page of this document."

Patients who have not progressed and who have not died by the date of data cutoff for analysis will be censored at the date of the last tumor assessment. If no tumor assessments were performed after the date of the first occurrence of a CR or PR, DOR will be censored at the date of the first occurrence of a CR or PR plus 1 day.

The DOR analysis will be performed on the basis of a non-randomized subset of patients (specifically, patients who achieve an objective response); therefore, formal hypothesis testing will not be performed for this endpoint. DOR will be estimated using Kaplan-Meier methodology. Comparisons between treatment arms will be made for descriptive purposes only.

Duration of Response as Assessed by the Independent Review Facility

The methodologies outlined for the analysis of DOR per the investigator will be used for the analyses of DOR based on IRF assessment.

Progression-Free Survival at Landmark Timepoints

The PFS rates at 6 months and 1 year will be estimated using Kaplan-Meier methodology for each treatment arm, along with 95% CIs calculated using the standard error derived from Greenwood's formula. The 95% CI for the difference in PFS rates between the two treatment arms will be estimated using the normal approximation method.

13.6.2.3 Patient-Reported Outcome Analyses

Time to deterioration (TTD) analyses will be performed on lung cancer symptom scores in the ITT population and will include all data collected through disease progression and survival follow-up.

TTD with the SILC questionnaire is defined as the time from randomization until the first confirmed clinically meaningful deterioration in the SILC symptom score (cough, dyspnea, or chest pain). Confirmed clinically meaningful deterioration in symptoms is defined as a score change that must be maintained for at least two consecutive assessments or an initial increase above baseline followed by 1) death within 3 weeks from the last assessment through Cycle 4; or 2) death within 6 weeks from the last assessment from Week 12 through Week 48; or 3) death within 9 weeks from the last assessment from Week 48.

TTD will be documented for the symptom score (cough, dyspnea, and chest pain per the SILC questionnaire).

Additional analyses below will utilize ITT patients with a baseline assessment and at least one post-baseline assessment (PRO-evaluable subset).

"Use of this document is governed by the terms of use on the first page of this document."

Compliance rates will be summarized by listing the number and proportion of patients in the PRO-evaluable subset who completed the PRO assessments at each timepoint. Responses for non-completion will be summarized if available. SILC and EORTC scores and change from baseline scores will be analyzed descriptively using means, standard deviations, medians, and range at baseline and at each post-baseline timepoint. Graphs of the mean changes and standard errors over time from the baseline assessment will be provided for all items and scales. The number and proportion of patients who improved, worsened, or remained stable compared with their baseline score will be summarized for the SILC score as well as all items and scales of the EORTC QLQ-C30 at baseline and each post-baseline timepoint for the PRO-evaluable population.

Further details regarding all PRO analyses will be provided in the SAP.

13.6.2.4 Exploratory Analysis

Time to Response as Assessed by the Investigator

Time to response (TTR) will be assessed in patients who had an objective response as determined by the investigator using RECIST v1.1. TTR is defined as the time from the date of randomization to the date of first occurrence of a CR or PR (whichever status is recorded first). No censoring observation will occur by definition. TTR is based on a non-randomized subset of patients (specifically, patients who achieved an objective response); therefore, formal hypothesis testing will not be performed for this endpoint. Comparisons between treatment arms will be made for descriptive purposes. The methodologies outlined for the analysis of PFS will be those used for the analyses of TTR.

[redacted]

Disease Control Rate as Assessed by the Investigator

DCR is defined as the rate of patients with complete or partial response as best response or stable disease maintained for ≥6 weeks per RECIST v1.1.

Overall Survival Analysis at Landmark Timepoints

The OS rates at 1 and 2 years will be estimated using Kaplan-Meier methodology for each treatment arm, along with 95% CIs calculated using the standard error derived from Greenwood's formula. The 95% CI for the difference in OS rates between the two treatment arms will be estimated using the normal approximation method.

"Use of this document is governed by the terms of use on the first page of this document."

13.6.3 Interim Analysis

13.6.3.1 Planned Interim Analysis

There is no interim analysis planned for PFS in this cohort. No iDMC or IMC will be set up for this cohort.

If PFS in bTMB PP1 shows superiority of atezolizumab over chemotherapy, OS in bTMB PP1 will be tested at $\alpha = 0.025$ (one-sided). One interim analysis of OS in bTMB PP1 for comparison of atezolizumab versus chemotherapy will be conducted at the time of the final PFS analysis (with approximately 128 OS events). The final OS analysis will be conducted after approximately 190 total deaths have occurred in both the atezolizumab and chemotherapy arms in bTMB PP1. This is expected to occur approximately 46 months after the first patient is randomized.

If OS in bTMB PP1 and PFS in bTMB PP2 also show superiority of atezolizumab over chemotherapy, OS in bTMB PP2 will be tested at $\alpha = 0.025$ (one-sided). The OS analyses in bTMB PP2 will be performed at the same time as the OS test in bTMB PP1. The stopping boundaries for OS interim and final analyses in bTMB PP1 and bTMB PP2 population are computed using the Lan-DeMets approximation to the Pocock boundary as shown in Table C-16. The actual stopping boundaries will depend on the actual number of OS events in bTMB PP1 and bTMB PP2 populations.

Table C-16 Analysis Timing and Stopping Boundary for Overall Survival in bTMB PP1 and bTMB PP2 Population

		bTMB PP1		bTMB PP2	
Analysis	Expected Timing of Analysis	Information fraction (number of events)	Stopping Boundary in HR (p-value)	Information fraction (number of events)	Stopping Boundary in HR (p-value)
Interim OS Analysis	~32 months from FPI	67% (128)	HR $\leq$ 0.693 ($\leq$ 0.0192)	72% (229)	HR $\leq$ 0.762 ($\leq$ 0.0201)
Final OS Analysis	~46 months from FPI	100% (190)	HR $\leq$ 0.722 ($\leq$ 0.0123)	100% (318)	HR $\leq$ 0.776 ($\leq$ 0.0121)

bTMB PP1 = the primary population of patients with a bTMB level equal to or greater than the higher validated cutoff; bTMB PP2 = the secondary population of all patients who are bTMB+, which is the intent-to-treat population in this cohort; FPI = first patient in; HR = hazard ratio; OS = overall survival.

"Use of this document is governed by the terms of use on the first page of this document."

13.6.3.2 Optional Interim Analysis

The Sponsor may choose to conduct one interim efficacy analysis for OS beyond what is specified in Section 13.6.3.1. The decision to conduct the optional interim analysis, along with the rationale, timing, and statistical details for the analysis, will be documented in the SAP, and the SAP will be submitted to relevant health authorities at least 2 months prior to the conduct of the interim analysis.

"Use of this document is governed by the terms of use on the first page of this document."

13.7 REFERENCES: bTMB+ COHORT

Alexandrov LB, Nik-Zainal S, Wedge DC, et al. Signatures of mutational processes in human cancer. Nature 2013;500:415–21.

Blank C, Gajewski TF, Mackensen A. Interaction of PD-L1 on tumor cells with PD 1 on tumor-specific T cells as a mechanism of immune evasion: implications for tumor immunotherapy. Cancer Immunol Immunother 2005;54:307–14.

Blank C, Mackensen A. Contribution of the PD-L1/PD-1 pathway to T-cell exhaustion: an update on implications for chronic infections and tumor evasion. Cancer Immunol Immunother 2007;56:739–45.

Brookmeyer R, Crowley J. A confidence interval for the median survival time. Biometrics 1982;38: 29–41.

Burman CF, Sonesson C, Guilbaud O, et al. A recycling framework for the construction of Bonferroni-based multiple tests. Stat Med 2009;28:739–61.

Butte MJ, Keir ME, Phamduy TB. Programmed death-1 ligand 1 interacts specifically with the B7-1 costimulatory molecule to inhibit T cell responses. Immunity 2007;27:111–22.

Calvert AH, Newell DR, Gumbrell LA, et al. Carboplatin dosage: prospective evaluation of a simple formula based on renal function. J Clin Oncol 1989;7:1748–56.

Campesato LF, Barroso-Sousa R, Jimenez L, et al. Comprehensive cancer-gene panels can be used to estimate mutational load and predict clinical benefit to PD-1 blockade in clinical practice. Oncotarget 2015;6:34221.

Chen DS, Irving BA, Hodi FS. Molecular pathways: next-generation immunotherapy–inhibiting programmed death-ligand 1 and programmed death-1. Clin Cancer Res 2012;18:6580–7.

Cockcroft DW, Gault MH. Prediction of creatinine clearance from serum creatinine. Nephron 1976;16:31–41.

Di Giacomo AM, Biagioli M, Maio M. The emerging toxicity profiles of anti–CTLA-4 antibodies across clinical indications. Semin Oncol 2010;37:499–507.

Fehrenbacher L, Spira A, Ballinger M, et al. Atezolizumab versus docetaxel for patients with previously treated non-small-cell lung cancer (POPLAR): a multicentre, open label, phase 2 randomised controlled trial. Lancet 2016;387:1837–46.

Hodi FS, O'Day SJ, McDermott DF, et al. Improved survival with ipilimumab in patients with metastatic melanoma. N Engl J Med 2010;363:711–23.

Hwang IK, Shih WJ, De Cani JS. Group sequential designs using a family of type I error probability spending functions. Stat Med 1990;9:1439–45.

"Use of this document is governed by the terms of use on the first page of this document."

Johnson DB, Frampton GM, Rioth MJ, et al. Targeted next generation sequencing identifies markers of response to PD-1 blockade. Cancer Immunol Res 2016;4:959–67.

Kantoff PW, Higano CS, Shore ND, et al. Sipuleucel-T immunotherapy for castration resistant prostate cancer. N Engl J Med 2010;363:411–22.

Keir ME, Butte MJ, Freeman GJ, et al. PD-1 and its ligands in tolerance and immunity. Annual Rev Immunol 2008;26:677–704.

Kowanetz M, Zou W, Shames DS, et al. Tumor mutation load assessed by FoundationOne (FM1) is associated with improved efficacy of atezolizumab (atezo) in patients with advanced NSCLC. Ann Oncol 2016;27(suppl 6):77P.

Lawrence MS, Stojanov P, Polak P, et al. Mutational heterogeneity in cancer and the search for new cancer-associated genes. Nature 2013;499:214–8.

Le DT, Uram JN, Wang H, et al. PD-1 blockade in tumors with mismatch-repair deficiency. N Eng J Med 2015;372:2509–20.

NCCN Clinical Practice Guidelines in Oncology (NCCN Guidelines®). Myeloid Growth Factors. Version 2.2016

Rizvi NA, Hellmann MD, Snyder A, et al. Mutational landscape determines sensitivity to PD-1 blockade in non–small cell lung cancer. Science 2015;348:124–8.

Rosenberg JE, Hoffman-Censits J, Powles T, et al. Atezolizumab in patients with locally advanced and metastatic urothelial carcinoma who have progressed following treatment with platinum-based chemotherapy: a single-arm, multicentre, phase 2 trial. Lancet 2016;387:1909–20.

Sandler A, Gray R, Perry MC, et al. Paclitaxel–carboplatin alone or with bevacizumab for non–small-cell lung cancer. N Engl J Med 2006;355:2542–50.

Smith TJ, Bohlke K, Lyman GH, et al. Recommendations for the use of WBC growth factors: American Society of Clinical Oncology clinical practice guideline update. J Clin Oncol 2015;33:3199–212.

Snyder A, Makarov V, Merghoub T, et al. Genetic basis for clinical response to CTLA-4 blockade in melanoma. N Eng J Med 2014;371:2189–99.

Socinski M, Velcheti V, Mekhail T, et al. Final Efficacy Results From B-F1RST, a Prospective Phase II Trial Evaluating Blood-Based Tumor Mutational Burden (bTMB) as a Predictive Biomarker for Atezolizumab in First-Line Non-Small Cell Lung Cancer (NSCLC). Ann Oncol (2019) 30 (suppl_5): v851–v934. 10.1093/annonc/mdz394

"Use of this document is governed by the terms of use on the first page of this document."

Stinchcombe TE, Socinski MA, Lee CB, et al. Considerations for second-line therapy of non-small cell lung cancer. Oncologist 2008;13:128–36.

Tecentriq® (atezolizumab) U.S. Prescribing Information, Genentech.

Vogelstein B, Papadopoulos N, Velculescu VE, et al. Cancer genome landscapes. Science 2013;339:1546–58.

Yang J, Riella LV, Chock S. The novel costimulatory programmed death ligand 1/B7.1 pathway is functional in inhibiting alloimmune responses in vivo. J Immunol 2011;187:1113–9.

Yang J, Riella LV, Chock S, et al. The novel costimulatory programmed death ligand 1/B7.1 pathway is functional in inhibiting alloimmune responses in vivo. J Immunol 2011;187:1113–9.

"Use of this document is governed by the terms of use on the first page of this document."

13.8 SCHEDULE OF ACTIVITIES: bTMB+ COHORT

	Screening [a]	Treatment Cycles (21-day cycles)		Treatment Discontinuation [b]	Follow-Up
		Cycle 1	Cycles 2 and Beyond		
	Days –28 to –1	Day 1 (±3 days)	Day 1 (±3 days)	≤30 Days after Last Dose	
Cohort C informed consent	x				
Patient-reported outcomes [c]		x	x [c]		x [d]
Vital signs [e]	x	x	x	x	
Weight	x	x	x	x	
Height	x				
Complete physical examination [f]	x			x	
Limited physical examination [g]		x	x		
ECOG Performance Status	x	x	x	x	
12-lead ECG [h]	x	x [i]		x [i]	
Hematology [j]	x	x [k]	x	x	
Chemistry [l]	x	x [k]	x	x	
Pregnancy test [m]	x	x [m]	x [m]	x [m]	
Coagulation (INR, aPTT)	x			x	
TSH, free T3 (or total T3), free T4	x	x [n]		x	
Viral serology [o]	x				
Urinalysis [p]	x	As clinically indicated			
Blood ctDNA sample		x	Cycle 3 and every odd cycle thereafter	x	
Optional tumor sample [q]		Any time (at investigator's discretion)			

"Use of this document is governed by the terms of use on the first page of this document."

	Screening [a]	Treatment Cycles (21-day cycles)		Treatment Discontinuation [b]	
		Cycle 1	Cycles 2 and Beyond		
	Days –28 to –1	Day 1 (±3 days)	Day 1 (±3 days)	≤30 Days after Last Dose	Follow-Up
Tumor assessments	x [r]	x [s]			
Concomitant medications [t]	x [t]	x	x	x	
Adverse events [u]	x [u]	x [u]	x	x	x [u]
Study treatment administration [v]		x	x		
Survival follow-up and anti-cancer treatment					x [w]

CT = computed tomography; ctDNA = circulating tumor DNA; ECG = electrocardiogram; ECOG = Eastern Cooperative Oncology Group; eCRF = electronic Case Report Form; EORTC = European Organisation for Research and Treatment of Cancer; EQ-5D-5L = EuroQol 5 Dimension, 5-Level questionnaire; HBcAb = hepatitis B core antibody; HBsAb = hepatitis B surface antibody; HBsAg = hepatitis B surface antigen; MRI = magnetic resonance imaging; PRO = patient-reported outcome; QLQ-C30 =Quality of Life Questionnaire Core 30; RECIST v1.1 = Response Evaluation Criteria in Solid Tumors, Version 1.1; SILC = Symptoms in Lung Cancer T3 = triiodothyronine; T4 = thyroxine; TSH = thyroid-stimulating hormone.

Notes: On treatment days, all assessments should be performed prior to dosing, unless otherwise specified.

Assessments shaded in gray should be performed as scheduled, but the associated data do not need to be recorded on the eCRF (except in the case of an adverse event).

[a] Results of standard-of-care tests or examinations performed prior to obtaining informed consent and within 3 days prior to Day 1 may be used; such tests do not need to be repeated for screening.

[b] Patients who discontinue study treatment will return to the clinic for a treatment discontinuation visit not more than 30 days after the last dose of study treatment. The visit at which response assessment shows progressive disease may be used as the treatment discontinuation visit.

[c] The questionnaires will be completed in-clinic every cycle (every 3 weeks) from Cycle 1 (baseline) through Cycle 4 (Week 9), then completed according to the tumor assessment schedule thereafter (i.e., every 6 weeks [±7 days] for 48 weeks [Week 12, Week 18, etc.] then every 9 weeks [±7 days] following the completion of the Week 48 assessment [Week 57, Week 66, etc.]) until radiographic disease progression per RECIST v1.1 (or loss of clinical benefit for atezolizumab-treated patients who continue treatment after disease progression according to RECIST v1.1) *or 111 weeks after Cycle 1,* death, withdrawal of consent or study termination by the Sponsor, whichever occurs first. Further guidelines on PRO questionnaire administration can be found in Section 4.5.9 and will be provided in the study manual.

"Use of this document is governed by the terms of use on the first page of this document."

[d] During survival follow-up, the SILC, EORTC QLQ-C30, and EQ-5D-5L will be completed at 3 *months (± 30 days)* and 6 months *(± 30 days)* following disease progression (or loss of clinical benefit for atezolizumab-treated patients who continue treatment after disease progression) according to RECIST v1.1, death, withdrawal of consent or study termination by the Sponsor, whichever occurs first. Patients who discontinue treatment for reasons other than disease progression will continue to complete the SILC, EORTC QLQ-C30, and EQ-5D-5L as per the tumor assessment schedule until radiographic disease progression per RECIST v1.1 *or 111 weeks after Cycle 1*, unless the patient withdraws consent or the Sponsor terminates the study.

[e] Includes respiratory rate, pulse rate, and systolic and diastolic blood pressure while the patient is in a seated position, and temperature. Record abnormalities observed at baseline on the General Medical History and Baseline Conditions eCRF. At subsequent visits, record new or worsened clinically significant abnormalities on the Adverse Event eCRF.

[f] Includes evaluation of the head, eyes, ears, nose, and throat, and the cardiovascular, dermatological, musculoskeletal, respiratory, gastrointestinal, genitourinary, and neurological systems. Record abnormalities observed at baseline on the General Medical History and Baseline Conditions eCRF. At subsequent visits, record new or worsened clinically significant abnormalities on the Adverse Event eCRF.

[g] Perform a limited, symptom-directed examination at specified timepoints and as clinically indicated at other timepoints. Record new or worsened clinically significant abnormalities on the Adverse Event eCRF.

[h] ECG recordings will be obtained during screening. Patients should be resting in a supine position for at least 10 minutes prior to ECG recording.

[i] ECG to be completed as clinically indicated throughout the study.

[j] Hematology includes WBC count, RBC count, hemoglobin, hematocrit, platelet count, differential count (neutrophils, eosinophils, basophils, monocytes, lymphocytes, other cells). Hematology tests must be performed prior to Day 1 infusions, and for gemcitabine administration, also prior to Day 8 infusions.

[k] If screening laboratory assessments were performed within 14 days prior to Day 1 of Cycle 1, they do not have to be repeated.

[l] Chemistry panel (serum or plasma) includes sodium, potassium, magnesium, chloride, bicarbonate, glucose, BUN or urea, creatinine, total protein, albumin, phosphorus, calcium, total bilirubin, alkaline phosphatase, ALT, AST, LDH.

[m] All women of childbearing potential will have a serum pregnancy test at screening (must be within 7 days of treatment initiation). Urine pregnancy tests will be performed prior to each subsequent cycle and at treatment discontinuation. If a urine pregnancy test is positive, it must be confirmed by a serum pregnancy test.

[n] TSH, free T3 (or total T3 for sites where free T3 is not performed), and free T4 will be assessed on Day 1 of Cycle 1 and every fourth cycle thereafter.

[o] At screening, patients will be tested for HIV, HBsAg, total HBcAb, and HCV antibody. If a patient has a negative HBsAg test and a positive total HBcAb test at screening, an HBV DNA test must also be performed to determine if the patient has an HBV infection. If a patient has a positive HCV antibody test at screening, an HCV RNA test must also be performed to determine if the patient has an HCV infection.

[p] Includes pH, specific gravity, glucose, protein, ketones, and blood); dipstick permitted.

"Use of this document is governed by the terms of use on the first page of this document."

[q] Can be taken from tumor blocks if available.

[r] All measurable and evaluable lesions should be assessed and documented at screening. Tumor assessments performed as standard of care prior to obtaining informed consent and within 28 days prior to initiation of study treatment do not have to be repeated at screening. Screening assessments must include CT scans (with oral or IV contrast) or MRI scans of the chest, abdomen, pelvis, and head. A spiral CT scan of the chest may be obtained but is not a requirement. If a CT scan with contrast is contraindicated (i.e., in patients with contrast allergy or impaired renal clearance), a non-contrast CT scan of the chest may be performed and MRI scans of the abdomen, pelvis, and head should be performed. An MRI scan of the head is required to confirm or refute the diagnosis of CNS metastases at baseline in the event of an equivocal CT scan. Bone scans and CT scans of the neck should also be performed if clinically indicated. Pelvic imaging beyond screening is required only as clinically indicated or as per local standard of care. Patients with a history of irradiated brain metastases at screening are not required to undergo brain scans at subsequent tumor evaluations unless scans are clinically indicated (see cohort-specific appendix for more details). See Section 4.5.5 for more details.

[s] Patients will undergo tumor assessments at baseline, every 6 weeks (± 1 week) for the first 48 weeks following treatment initiation, and every 9 weeks (± 1 week) thereafter, regardless of dose delays, until radiographic disease progression per RECIST v1.1 or loss of clinical benefit (for atezolizumab-treated patients who continue treatment after radiographic disease progression per RECIST v1.1) as determined by the investigator. Thus, tumor assessments are to continue according to schedule in patients who discontinue treatment for reasons other than disease progression or loss of clinical benefit, even if they start new anti-cancer therapy.

All measurable and evaluable lesions should be re-assessed at each subsequent tumor evaluation. The same radiographic procedures used to assess disease sites at screening should be used for subsequent tumor assessments (e.g., the same contrast protocol for CT scans).

[t] Includes any medication (e.g., prescription drugs, over-the-counter drugs, vaccines, herbal or homeopathic remedies, nutritional supplements) used by a patient in addition to protocol-mandated study treatment from 7 days prior to cohort-specific treatment consent until the treatment discontinuation visit.

[u] All serious adverse events and adverse events of special interest, regardless of relationship to study drug, will be reported until 90 days after the last dose of study drug or initiation of new systemic anti-cancer therapy after last dose of study treatment.

[v] The initial dose of atezolizumab will be delivered over 60 (± 15) minutes. Subsequent infusions will be delivered over 30 (± 10) minutes if the previous infusion was tolerated without infusion-associated adverse events, or 60 (± 15) minutes if the patient experienced an infusion-associated adverse event with the previous infusion.

[w] After treatment discontinuation, information on survival follow-up and new anti-cancer therapy (including targeted therapy and immunotherapy) will be collected via telephone calls, patient medical records, and/or clinic visits approximately every 3 months (unless the patient withdraws consent or the Sponsor terminates the study). If a patient requests to be withdrawn from follow-up, this request must be documented in the source documents and signed by the investigator. If the patient withdraws from study, the study staff may use a public information source (e.g., county records) to obtain information about survival status only.

"Use of this document is governed by the terms of use on the first page of this document."
